# HIV Reverse Transcriptase and Protease Genes Variability Can Be a Biomarker Associated with HIV and Hepatitis B or C Coinfection

**DOI:** 10.1038/s41598-018-26675-z

**Published:** 2018-05-29

**Authors:** Natália Mirele Cantão, Lauana Fogaça de Almeida, Ivan Rodrigo Wolf, Rodrigo Oliveira Almeida, Andressa Alves de Almeida Cruz, Caroline Nunes, Alexandre Naime Barbosa, Guilherme Targino Valente, Maria Inês de Moura Campos Pardini, Rejane Maria Tommasini Grotto

**Affiliations:** 1 São Paulo State University (Unesp), Medical School, Botucatu, Sao Paulo State Brazil; 2 São Paulo State University (Unesp), School of Agriculture (FCA), Department of Bioprocess and Biotechnology, Botucatu, Sao Paulo State Brazil

## Abstract

Variability of the HIV reverse transcriptase (RT) and protease (PR) genes has been used as indicators of drug resistance and as a mean to evaluate phylogenetic relationships among circulating virus. However, these studies have been carried in HIV mono-infected populations. The goal of this study was to evaluate, for the first time, the HIV PR and RT sequences from HIV/HBV and HIV/HCV co-infected patients. HIV PR and RT genes were amplificated and sequenced to resistance analysis. The bioinformatics analysis was performed to infer about sequences clustering and molecular evolution. The results showed that the most frequent amino acid substitutions in RT were L214F (67.6%), I135T (55.9%), and in PR was V15I (41.2%). The molecular clock analysis showed that the HIV circulating in co-infected patients were separated in two clusters in the years 1999–2000. Some patients included as HIV mono-infected according patients’ medical records and inside the co-infected cluster were, in fact, co-infected by PCR analysis. Analysis of the decision trees showed susceptibility to lamivudine and emtricitabine were important attribute to characterize co-infected patients. In conclusion, the results obtained in this study suggest, for the first time, that HIV RT and PR genes variability could be a genetic biomarker to coinfection.

## Introduction

Although several advances in HIV diagnosis, prognosis and therapies have been achieved in recent years, HIV/AIDS epidemic remains a public health problem^1^. It is estimated that 36.7 million people worldwide are currently HIV carriers, and 882,810 individuals have AIDS in Brazil^2,3^.

Distinct HIV subtypes, circulating recombinant forms (CRF) and *quasispecies* have been isolated from infected patients^4–7^ due to the high degree of HIV genetic variability^8^, a consequence of the selective pressure of the host immune system and/or antiretroviral therapy^9^.

When genetic variability is present in HIV, protease (PR) and/or reverse transcriptase (RT) genes are particularly important. Drug classes against these targets are still used as first-line antiretroviral treatment^10^. The PR and RT enzyme variability can decrease HIV fitness^11^ (QUINONES-MATEU, 2000) and leads to further variability as a compensatory mechanism for virus propagation^12^.

Several studies have been reported in the literature about HIV genetic variability in PR and RT genes as indicators of drug resistance^13–15^, viral fitness^16^, and transmitted resistance^17,18^ and as a mean to evaluate phylogenetic relationships among circulating virus^19^. However, these studies have been carried in HIV mono-infected (mi) populations.

Until now, there has been no information about HIV PR and RT genetic variability in patients who are co-infected (co) with HIV and either the Hepatitis C (HCV) or B (HBV) Virus.

The presence of HCV or HBV in HIV infected patients has previously been associated with patient deaths^20^. The hepatic virus leads to a more rapid progression of HIV infection^21^. On the other hand, studies have demonstrated that HIV infected patients using HAART had mortality when presenting with HCV or HBV^22^.

In addition, it has been demonstrated that parasitosis in HIV infected patients can alter the HIV dynamic^23^.

In this context, the goal of this study was to evaluate, for the first time, the HIV PR and RT sequences from co-infected patients who accessed public health services in Brazil.

## Results

Table 1 presents the patient characteristics included in this study. The co-infected patients were diagnosed with HIV infection between 1995 and 2007 and the second viral infection (HBV or HCV) was detected at the same time. The most of co-infected patients were male (70.59%), with age median 43.5 (IQR: 39.0–48.7). From co-infected patients, 76.5% had AIDS and, the most of them (82.35%) were under antiretroviral therapy by the first time. Other virologic and immunological characteristics is in the Table 1.

**Table 1:** HIV monoinfected (n = 75) and HIV/HBV or HIV/HCV coinfected (n = 34) patients’ characteristics included in the analysis.

Characteristics	G1 (N = 75)	G2 (N = 34)
Age, years [median (IQR)]*	43.0 (38.0–50.5)	43.5 (39.0–48.7)
Sex, Male [N (%)]*	44.0 (58.67)	24.0 (70.59)
HIV Viral Load^*^ [N (%)]		
Lower 10,000 RNA copies/mL Higher 10,000 RNA copies/mL	24.0 (32.00) 51.0 (68.00)	19.0 (29.4) 15.0 (73.5)
TCD4 cells count^*^		
Lower 250 cells/mm^3^ Higher 250 cells/mm^3^	51.0 (68.00) 24.0 (32.00)	16.0 (47.1) 18.0 (52.9)
Aids Presence**		
Yes No	44.0 (58.67) 31.0 (41.33)	26.0 (76.5) 08.0 (23.5)
Subtipo HIV***		
B pure Non B	54.0 (72.0) 21.0 (28.0)	29.0 (85.3) 05.0 (14.7)

HIV: Human Immunodeficiency Virus; HBV: Hepatitis B Virus; HCV: Hepatitis C Virus; IQR: interquartile range. *Information according patients’ medical records. **According CDC classification (SELIK, 2014). ***Subtype according protease (PR) and reverse transcriptase (RT) genes. B pure is subtype B in PR and RT genes; non-B is subtype F or B recombinant with other subtypes.

Table 2 presents the number of Protease and Reverse Transcriptase resistance mutations in HIV mono-infected patients (G1) and HIV/HBV or HIV/HCV co-infected patients (G2) included in this study. G2 had a higher number of mutations to nucleoside analog reverse-transcriptase inhibitors (NRTI), and this group presented more wild type variation than G1 to non-nucleoside analog reverse-transcriptase inhibitors (NNRTI) (P < 0.05).

**Table 2:** Number of Protease and Reverse Transcriptase resistance mutations in HIV monoinfected (G1) and HIV/HBV or HIV/HCV coinfected (G2) patients included in this study.

HIV Enzyme	G1 (n = 75) N (%)	G2 (n = 34) N (%)
Protease		
0	18	09
1	08	05
2	05	03
3 or more	33	17
Reverse Transcriptase (according NRTI)		
0	25	14
1	12	0
2	08	1
3 or more	19*	19*
Reverse Transcriptase (according NNRTI)		
0	27*	20*
1	15	7
2	06	3
3	12	4

NRTI: Nucleoside analog reverse-transcriptase inhibitors; NNRTI: Non-nucleoside analog reverse-transcriptase inhibitors. *Statistical difference (P < 0,05), Fisher’s Exact Test.

**Figure 1:**
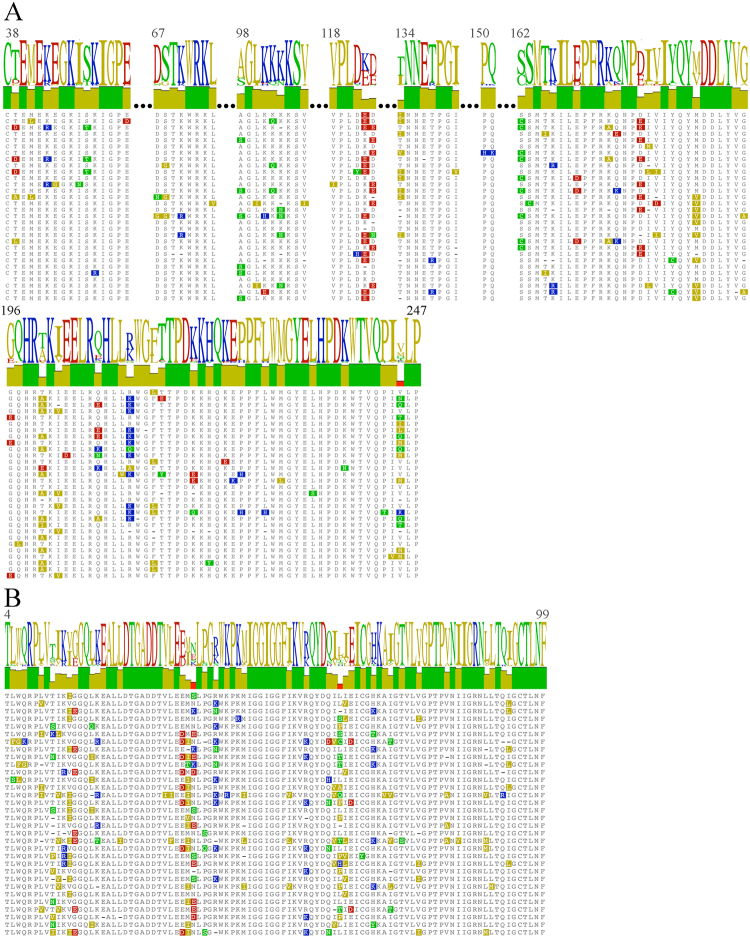
Mutational points of partial protease and reverse transcriptase of HIV in co-infected patients. It is highlighted the mutational points in the alignment. The bars represent the identity of alignment and the logo is also shown. The first sequence in each alignment is the reference genes of HIV. The ambiguous amino-acid (due to ambiguities in gene sequence) were substituted by “−”. 100% conserved blocks were cutoff the figure (“…” symbols). (**A**) Reverse transcriptase (64.3% of identical sites and 93% of pairwise identity); (**B**) protease (56.3% of identical sites and 87.5% of pairwise identity).

Figure 1 shows resistance mutations and genetic polymorphisms for the 34 samples from HIV/HBV and HIV/HCV co-infected patients. Global sequence analysis showed that the most frequent amino acid substitutions in RT were S162C (29.4%), T200A (35.3%), M184V (32.3%), L214F (67.6%), I135T (55.9%), D177E (26.5%) and E122K (44.1%), and the most in PR were V15I (41.2%), M36I (38.2%) and E35D (23.5%).

Thymidine Analog Mutations (TAMs) were found in nine (26.5%) samples, with one or more mutations among M41L, D67N, K70R, L210W, T215Y/F e K219Q/E. The TAM mutation way more present was TAM 2 (D67N, K70R, T215F and K219Q/E), and was present in seven (77.8%) patients. Only one (11.1%) patient showed the TAM 1 (M41L, L210W and T215Y) mutation pathway, and one patient (11.1%) had both pathways.

K103N was the NNRTI mutation most frequently found in co-infected patients (14.7%). The major mutations to PI were I54V (2.9%), V82A (11.7%), M46I (8.8%) and L90M (40.4%). On the other hand, the minor mutations to PI found were S37N (32.3%), I15V (26.5%) and M36I (23.5%).

A significant difference (P < 0.05) was found in the number of NRTI mutations (when the mutation number is three or more) between mono-infected and co-infected patients. In the same way, the wild type was found to be significantly more present in co-infected patients than in HIV mono-infected when the presence of NNRTI mutations are considered (Table 2).

**Figure 2:**
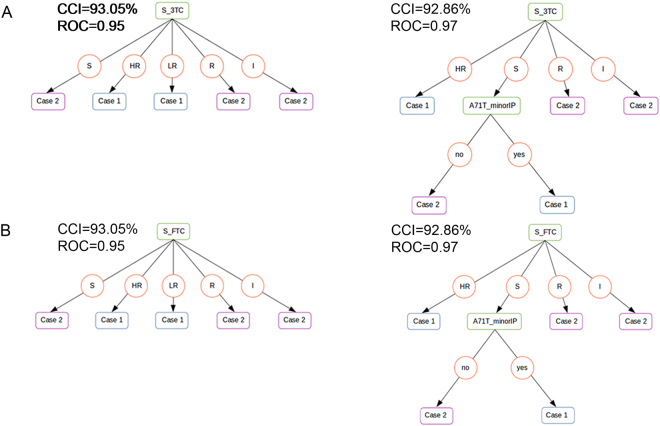
Decision trees generated by supervised learning. Left trees, tress generated with the dataset 1; Right trees, tress generated with the dataset 2; (**A**) training with all attributes; (**B**) training excluding S_3TC; Case 1, mono-infected; Case 2, co-infected. CCI, corrected classified instances; ROC, receiver operating characteristic.

The analysis of decision trees (Fig. 2; Table 3; Supplementary Figure 1) revealed that the most important attributes for which classify both groups of patients (co- and mono-infected) are susceptibility to Lamivudine (S_3TC) and presence of minor protease mutation in codon 71 (A71T_minorIP). The first iterative pruning (S_3TC exclusion) showed that susceptibility to Emtricitabine (S_FTC) is another important attribute, and its exclusion revealed that susceptibility to Nelfinavir (S_NFV), T215F_NRTI, F225H_NNRTI, A71_minorIP and S_NVP are also important attributes. Iterative pruning indicated other important attribute such as susceptibility to Abacavir (S_ABC), plasma viral load, age, susceptibility to Didanosine (S_DDI), P255H_NNRTI and susceptibility to Etravirine (S_ETR).

**Table 3:** Metrics of supervised learning.

Model	Decision trees	%C	*%TP	*%FP	*%prec	*%rec	*%roc
1	All attributes	93.05	93.1	6.5	93.4	93.1	95.3
	Less S_3TC	93.05	93.1	6.5	93.4	93.1	95.3
	Less S_3TC and S_FTC	88.89	88.9	10.3	90.1	88.9	91.1
	Less S_3TC, S_FTC and S_NFV	86.11	86.1	14	86.1	86.1	90.4
	Less S_3TC, S_FTC, S_NFV and S_ABC	79.16	79.2	20.5	79.5	79.2	81.4
2	All attributes	92.86	92.9	6.9	93.2	92.9	96.9
	Less S_3TC	92.86	92.9	6.9	93.2	92.9	96.9
	Less S_3TC and S_FTC	94.28	94.3	5.6	94.4	94.3	96.0
	Less S_3TC, S_FTC and S_NFV	90.0	90.0	10.1	90.0	90.0	91.7
	Less S_3TC, S_FTC, S_NFV and S_ABC	87.14	87.1	12.8	87.2	87.1	87.3

*Weighted average; C, corrected classified instances; TP, true positive rate; FP, false positive rate; prec, precission; rec, recall; roc, receiver operating characteristic.

S_3TC: Susceptibility to Lamivudine; S_FTC: Susceptibility to Emtricitabine; S_NFV: Susceptibility to Nelfinavir; S_ABC: Susceptibility to Abacavir.

**Figure 3:**
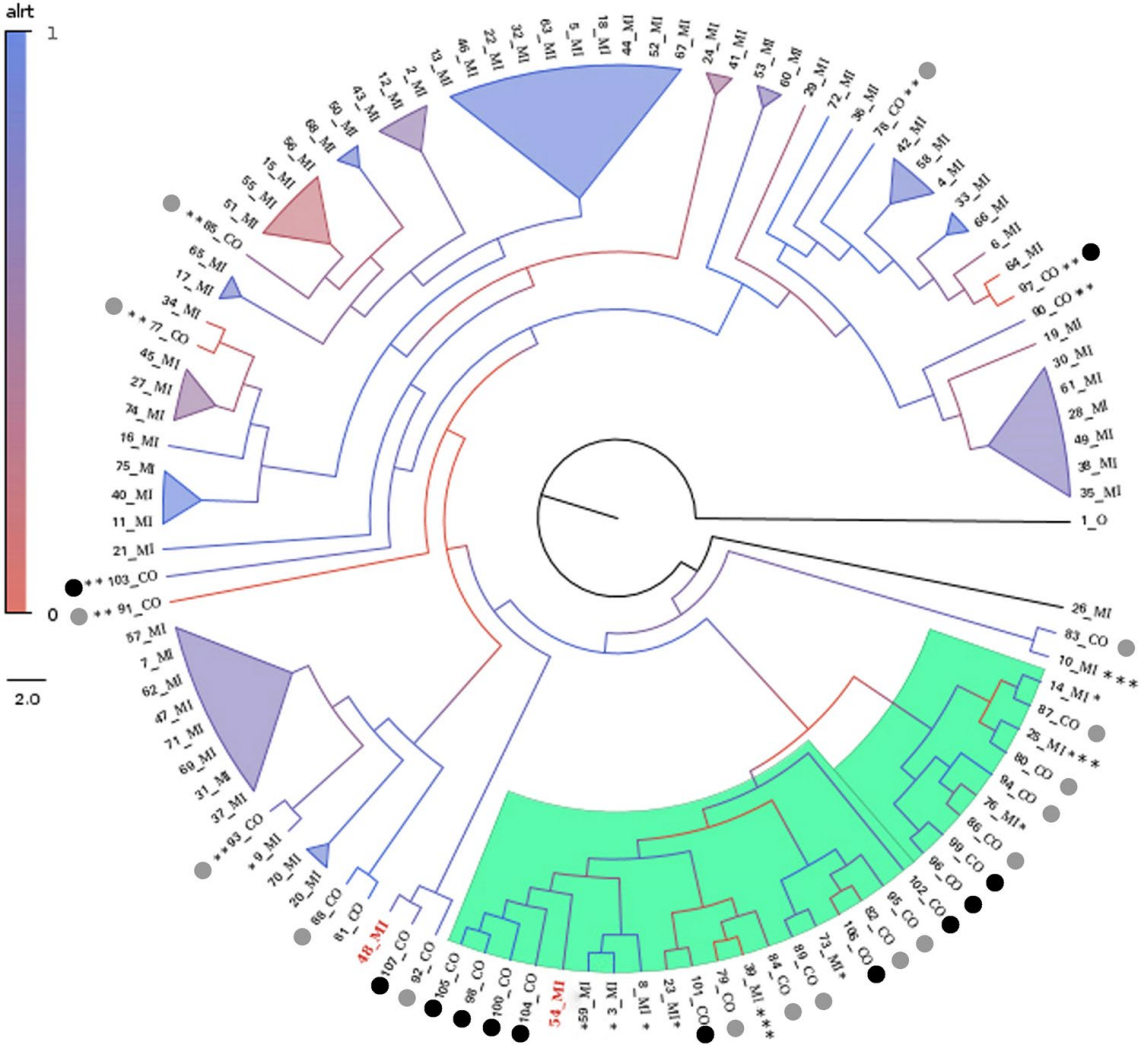
Cladogram showing the phylogenetic relationships among the HIV virus from the patients here analyzed. Green clades, co-infected clade; Black circle, HIV/HBV coinfected; Grey circle, HIV/HCV coinfected *mono-infected patients closely related to the co-infected; **co-infected patients out of the two co-infected clades; ***mono-infected with high probability to be co-infected; red names, patients previously determined as mono-infected but positive co-infected using PCR analysis.

The phylogenetic tree reported at least two clades with an enrichment of co-infected patients (18 individuals), despite the presence of some mono-infected inside these clades. Moreover, some sequences of co-infected patients (10 individuals) are outside of these clades and occasionally are closely related to HIV of mono-infected patients with good branch supports (Fig. 3). The application of models generated from supervised learning from mono-infected patients was closely related to the results of co-infected patients, and the results indicated that four of patients had a high probability to be co-infected. Subsequent PCR analysis confirmed that at least one patient was, in fact, co-infected; while additional PCR confirmed that another patient was also co-infected (Table 4).

**Table 4:** Results of classification over mono-infected patients using the models generated by supervised learning and PCR results.

ID	Prediction model 1	Probability	Prediction model 2	Probability	PCR
14_MI	mono-infected	1	mono-infected	1	—
9_MI	mono-infected	1	mono-infected	1	—
8_MI	mono-infected	1	mono-infected	1	—
10_MI*	co-infected	0.917	co-infected	0.917	—
3_MI	mono-infected	1	mono-infected	1	—
25_MI*	co-infected	0.917	mono-infected	1	—
23_MI	mono-infected	1	mono-infected	1	—
59_MI	mono-infected	1	mono-infected	1	Negative**
76_MI	mono-infected	1	—	—	—
73_MI	mono-infected	1	—	—	—
64_MI	mono-infected	1	mono-infected	1	—
39_MI*	co-infected	0.917	co-infected	0.917	Negative
48_MI	mono-infected	1	mono-infected	1	Positive
54_MI*	co-infected	0.917	co-infected	0.917	Positive**
26_MI	—	—	—	—	Negative

*Mono-infected patients classified as co-infected; **concordant results.

**Figure 4:**
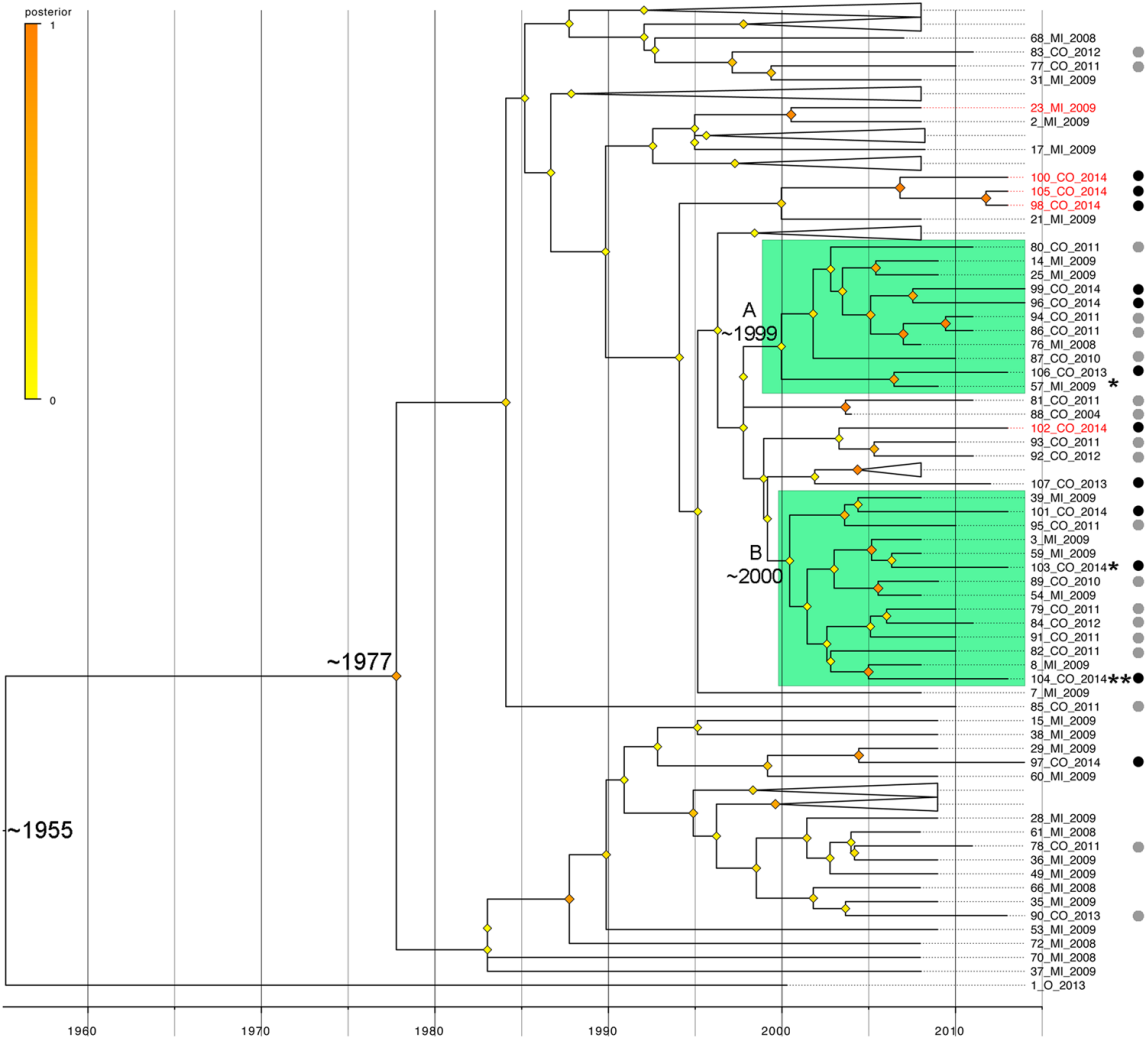
Cladogram with branches adjusted by molecular clock. Green clades, two main co-infected clades according Fig. 3; *patients inside the main two co-infected clades and not in agreement with Fig. 3; **co-infected patient under positive selection; Red names, patients outside the main two co-infected clades and not in agreement with Fig. 3. Black circle, HIV/HBV coinfected; Grey circle, HIV/HCV coinfected.

Molecular clock analysis allows us to date the origin of human HIV at ~1955, which agrees with previous study^24^. We also dated the origin of HIV of the patients analyzed here at ~1977 and the origin of the HIV present in both co-infected clusters (according the phylogenetic tree generated by maximum likelihood (Fig. 3)) at ~1999–2000. Interestingly, one co-infected patient [103_co (HIV/HBV)] who was outside of the two main clades in the maximum likelihood tree was placed in the co-infected clades, supporting the real existence of two main co-infected clades (Fig. 4).

Moreover, the patient 104_co (HIV/HBV), a long-branch individual in clade B of Fig. 4, is under positive selection (P-value < 0.0085) (Fig. 4). Two other patients are also under positive selection with P-values < 0.05 (51_mi and 6_mi).

## Discussion

The results showed there more NRTI resistance mutations than those for other drug classes (NNRTI and PI) in both groups (mono-infected and co-infected) (Table 2). This result could be explained using NRTI in the beginning of the antiretroviral therapy, and often, they were used in the past (until 1996) as a monotherapy and with lower efficacy^25,26^. The presence of M184V is related to early virologic failure during therapy with 3TC. In HIV mono-infected patients, this mutation is present with a frequency ranging from 60.4–68.3%^27–29^. In this study, this mutation was observed 32.3% of the time, and all patients with mutations were using 3TC, supporting the premise that the presence of M184V is due to the selective pressure of 3TC. This is useful because M184V reduces viral fitness^30^. This mutation leads to resistance to 3TC and FTC^31^, and all these patients presented resistance to these two drugs. These results found in co-infected patients included in this study corroborated the data obtained in the analysis of the decision trees (Fig. 2). Despite all decision trees being statistically supported, sometimes they are quite branched or do not have good CCI values or false positive rates. Thus, we suggest that S_3TC, A71T_minorIP and S_FTC are the most important attributes since they are reported in the first two iterative pruning’s, which show the best trees and statistics. Moreover, we suggest that A71T_minorIP is always dependent on a previous condition (S feature for both S_3TC and S_FTC). Interestingly, the trees in all tests for each dataset are relatively different from each other, which we presume reflects the low number of instances analyzed. On the other hand, the most important attributes of each dataset along the iterative pruning are the same when comparing both balanced datasets, supporting the interpretation that the aforementioned instances are the most important classifiers. Then, these results suggest that resistance to 3TC and FTC could be a molecular marker of co-infected patients.

The L214F mutation was the most frequent (Fig. 1) in co-infected patients (67.6%). This mutation has been related to Azvudine (FNC) resistance, a novel nucleoside reverse transcriptase inhibitor^32^. This finding is very important considering that FNC has not been available for clinical use; in this context, many co-infected patients could be presumed to have had transmitted resistance to FNC. I135T is a polymorphism also frequently found in co-infected patients in this study (55.9%) (Fig. 1). Some studies have related this polymorphism to increased resistance to efavirenz mediated by K103N in HIV mono-infected patients^33^, but this association was not found for co-infected patients in this study.

The other substitutions found in co-infected patients, E122K (44.1%), T200A (35.3%), S162C (29.4%) and D177E (26.5%) have been previously reported in studies with HIV mono-infected patients^34,35^.

It can be noted that the frequency of NNRTI mutations in co-infected patients is different than this frequency in HIV mono-infected patients, suggesting that the presence of HBV or HCV could be interfering with the selective pressure of HIV mutation perpetuation.

The frequency of TAMs found for NNRTI and PI resistance mutations in the co-infected patients was lower than the frequency reported in the literature for mono-infected patients (Table 2)^27,28^. It has been shown that co-infection with parasites can alter the HIV dynamic^23^. In the same way, these results suggest that the hepatotropic virus presence could affect the HIV mutation perpetuation due to alteration of the HIV dynamic.

The molecular clock analysis (Fig. 4) showed that the HIV circulating in two co-infected patients’ clusters was separated in the years 1999–2000. The most recent common ancestor for the co-infected variants are younger than the ones in monoinfected (>1997). Several events have marked the evolution of HIV infection between 1996 and 2001. In 1995, the protease inhibitors (PI) were introduced as an antiretroviral therapy^36^, and since the introduction of saquinavir, the first PI approved for clinical use, the combined HIV therapy termed highly active antiretroviral therapy (HAART) has been implemented^37^, leading to improved HIV cycle control^36^. In 2000, resistance testing was introduced by protease and reverse transcriptase gene genotyping in order to infer emergence of resistance in variants and to assist in the choice of therapy. The Genotyping Laboratory National Network was implemented in Brazil in 2001^38^. The co-infected patients included in the two clusters in green in Fig. 4 were infected by HIV between 1990 and 2000, dates that coincide with the introduction of PI and the clinical use of drug resistance tests. The majority of HIV mono-infected patients were infected before 1990. These facts could explain why the wild type variant is present mainly in co-infected patients (Table 2). Previous studies have demonstrated that HAART use is associated with lower hepatic disease progression^39^ and lower mortality in co-infected patients^22^.

In this analysis, patient 104_co (HIV/HBV) is a patient with symptomatic HIV infection, under positive selection pressure (Fig. 4), who had an HIV viral load of 5.4 log and a CD4 cell count of 23 cells/mm^3^ at the time of diagnosis. After HAART introduction, there was rapid immune restoration with low plasma viremia. Additionally, the patient had the L10V, M41L and T215E resistance codons. All other co-infected patients presented in this cluster had higher HIV viral loads and lower CD4 counts. Although the mutation L10V is an accessory mutation to PI, the mutations M41L and T215E are associated with resistance to NNRTI, and are therefore associated with cross resistance. It is probable that the HIV variant circulating in this patient is under selection from the efficient therapy and immunological pressure. These results suggest that the patient may have organ failure in the future and should be carefully monitored to observe the evolution of viral dynamics.

Although in Fig. 4, the patient 57_mi is in the co-infected cluster, when analysis was carried out, the patient was found also to be infected with HBV. This analysis shows that the two clusters in green (Fig. 4) are representative of co-infection.

Of the co-infected patients in the green cluster in the cladogram (Fig. 3), 16 (84.21%) were diagnosed with HIV after the advent of HAART. On the other hand, from the 13 co-infected patients outside this clade, 10 (76.92%) were diagnosed after HAART was in use. These findings suggest that the cluster is due to the presence of co-infection and is not influenced by the therapeutic schedule used, because all patients used HAART. This finding is supported by the fact that sample 54_mi was positive for HCV when PCR was performed (Table 3).

When making the phylogenetic analysis (Fig. 3), some samples were grouped into the green cluster, but they were not observed in the green cluster in Fig. 4. On the other hand, some samples were present in the green cluster in Fig. 4 but not in Fig. 3.

Patients that were present in the green cluster in Fig. 3 but not in Fig. 4 included patient 98_co (HIV/HBV) with resistance codon E138A; patient 100_co (HIV/HBV) with codons L10A and L10V; and patient 105_co (HIV/HBV) with codon K103E. They had similar characteristics such as subtype C, co-infection with HBV, AIDS patient, male gender, resistance the ETR drug, and use of ARV therapy. Patient 102_co (HIV/HBV) is also present in the green cluster in Fig. 3 but not in Fig. 4. This patient does not have resistance codons, is co-infected with HBV, is subtype B and is an AIDS patient. This patient was grouped (Fig. 4) with patients 93_co (HIV/HCV) and 92_co (HIV/HCV), which are subtype B, co-infected with HCV and AIDS patients.

Patient 57_mi was included in the green cluster in Fig. 4 but not in Fig. 3. This patient has the following resistance codons: M41L, D67H, T69N, K70R, L74I, V118I, T215F, K219E, K219Q and K103Y. Codon mutations K219Q, T69N, and K70R generate conditions where the drug AZT has decreased effectiveness^31^. The M41L mutation causes high cross resistance to the drug ABC^40,41^. T215F causes resistance to both the AZT and ABC drugs^31^. The codon L74I is selected for by ABC^42,43^. The codon V118I reduces the action of nucleoside reverse transcriptase inhibitor drugs (NRTIs)^44,45^. Although this patient has a lower HIV viral load and higher CD4 cell count (700 cells/mm^3^), the presence of these mutations may lead to a progression of infection.

Patient 91_co (HIV/HCV) has the codons T12S, L19I, L74V, K103R, E122K, D123E, I135T, S163C, M184V, L214F, K219E, E224K, V245M, L100I, V106I, V179D and M230L and used the drugs 3TC, EFV and TDF for treatment. The mutations K103R and V179D together reduce the virus susceptibility by more than 10 times compared to the drugs NVP and EFV^46^. The mutations V106I and V179D together may cause resistance the drugs NVP, EFV, ETR and RPV^47^.

The other patient present in the cluster is 103_co (HIV/HBV); however, this patient is different because he has an undetectable HIV viral load and a CD4 count higher than 700 cells/mm^3^, characterizing a slow HIV infection progression. In addition, this patient maintained an undetectable HBV viral load. The resistance codons that are present include K238N and L10I, which are indicative of infection progression because these mutations are associated with high viral replication and a decreased effectiveness of PI and NNRTI class drugs^48–50^. In the same way, an efficient immune response could be difficult to maintain under both the HIV and HCV conditions. Similar characteristics with others in this group are present; both are subtype B and AIDS patients, and they are grouped close together in Fig. 4.

People infected with the virus either have not been treated (naïve) or have been treated. When infection occurs, resistant strains can be transmitted. Transmitted resistance is the transfer of viral mutations that cause decreased effectiveness of drugs used in treatment for controlling the disease. However, the viral fitness of resistant strains is sometimes less than that of the wildtype virus, and the use of drugs interrupts the growth and the replication of the wildtype virus. Transmitted resistance is not always negative because as in patient 104_co (HIV/HBV), over time, there was an increase in the number of CD4 cells and a decreased HIV viral load.

When patients co-infected with HIV and HBV are considered, it is observed that four patients are outside of the cluster (103_co (HIV/HBV), 97_co (HIV/HBV), 107_co (HIV/HBV), 57_mi) in Fig. 3. Patients 103_co and 107_co have asymptomatic infection by HIV and no detectable HIV viral load, CD4 count cell or transmission path. However, when these patients were evaluated by cladogram (Fig. 4), only patients 97_Co (the only co-infected patient with HBV with subtype KF) and 107_co were outside the clusters. This result shows that the non-presence of AIDS in a patient co-infected with HIV and HBV could be a factor to phylogenetically differentiate patients. Patient 103_co had selective pressure from drug therapy and presented resistance to EFV and NVP. Patient 97_co was infected in 2014; this proves that the analysis made from the cladogram is correct (Fig. 4). All patients co-infected with HIV and HCV that are outside of the green cluster in Fig. 3 are also outside of the cluster in Fig. 4, except patient 91_co (HIV/HCV).

The phylogenetic tree results indicate that the patients inside the two main groups of co-infected patients may be infected by up to two different HIV lineages since the branch that supports both clusters is not well supported. Moreover, we have also shown that the HIV infections were quite recent, ranging from the end of the 90’s to the beginning of the 2000’s.

The high probability of four mono-infected to be HCV/HBV infected (at least 1 was positive by PCR) and the low distribution of co/mono-infected patients outside their expected clades, conduct us to consider that HCV/HBV infections may be influencing the HIV evolution. The molecular clock also allowed to date the origin of variants that infected the patients in two main clades as being ~1999 and ~2000, which is a recent origin.

Although the HIV infection progression is dependent of viral^51^ and host factors^52^ the presence of the co-infection could lead to overload the host immune system^53^. The HIV variant circulating in plasma from co-infected patients represents the most adapted strain under these conditions (co-infection). The results presented here showed that the presence of a hepatotropic virus (HBV or HCV) can be lead to emergence of the specific HIV variants.

In conclusion, the results obtained in this study suggest, for the first time, that HIV RT and PR genes variability could be a genetic biomarker to coinfection with HBV or HCV. Knowledge of the HIV RT and PR sequences could provide information for clinical investigation about possible HBV or HCV coinfection.

## Methods

### Patients’ Selection

Aliquots of EDTA-anticoagulated peripheral venous blood were collected from 34 HIV/Hepatitis (B or C) Virus coinfected patients from the Infectious Diseases Specialized Assistance Service, Botucatu Medical School, Sao Paulo State University, UNESP, Botucatu, SP, Brazil. Inclusion criteria were the presence of HIV and HCV or HBV confirmed in patients’ medical registers. Exclusion criteria were: patients with age below 18 years old, pregnant women and the presence of other hepatic diseases. Then, the patients included in this study represent all coinfected population in studied region.

Sample collection from HIV/Hepatitis (B or C) Virus coinfected patients was performed between 2010 and 2014 according to the attendance of these patients in the healthy service for their follow-up. The included patients resided in the region of Botucatu city, Sao Paulo State, Brazil.

This study was approved by the Research Ethics Committee of Botucatu Medical School, UNESP and performed according national ethical procedures. All methods used in this study are in accordance with the ethical principles for human experimental procedures. The informed consent was obtained from all participants included in this study.

To comparative analysis was used from a data bank with 289 HIV monoinfected patients assisted in the same healthy service, which represents the same geographic region. From this bank were selected 75 patients according bioinformatic analysis results (see Bioinformatic Analysis).

**Figure 5:**
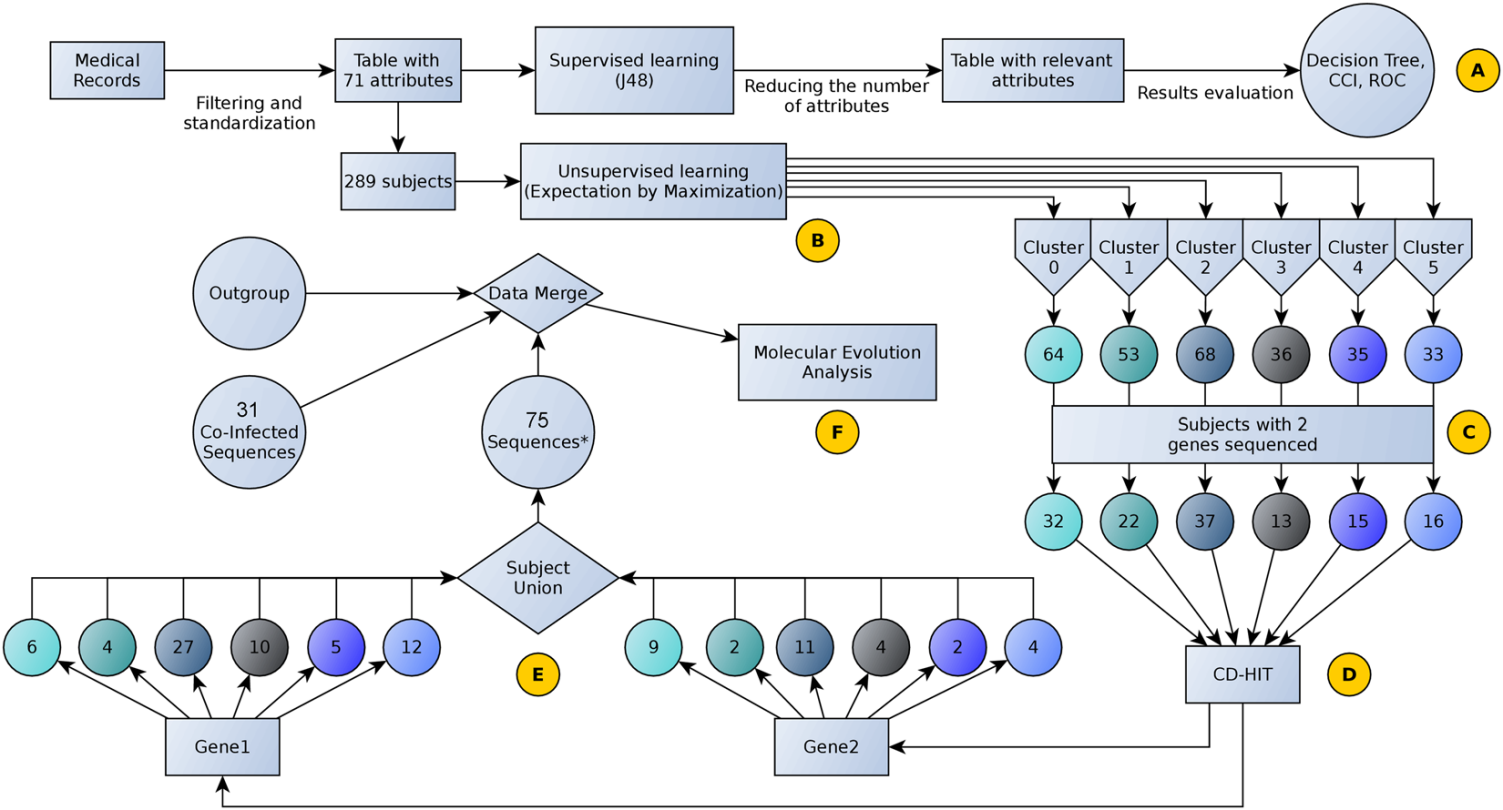
Bioinformatics pipeline. Small yellow spheres, steps.

This data bank from HIV monoinfected patients was constituted with sequences obtained from HIV patients seen between 2008 and 2013 according their attendance in the healthy service for their follow-up. All these HIV monoinfected patients were from Botucatu city region, Sao Paulo State, Brazil (Fig. 5).

### Laboratory Analysis

RNA isolated from plasma using QIAamp RNA Viral Mini Kit (Qiagen, Valencia, CA, USA) was used as source to amplification and sequencing of the HIV PR and RT genes using Trugene HIV-1 Genotyping Kit (Siemens Healthcare Diagnostics, Inc. Tarrytown, NY, USA). The Trugene HIV-1 Genotyping kit is a method used to detect the major HIV strain from plasma in HIV infected patient. This strain represents the major quasispecies circulating in infected patient^54^ but other variants can be present in others biological reservoirs. All conclusions of this study are about the selective pressure to HIV variant circulating in plasma which represents the active (in replication) strain.

All procedures were performed according manufacture’s recommendations. These laboratory procedures were performed to 289 HIV monoinfected patients and 34 HIV/HCV and HIV/HBV coinfected patients (the condition of the HIV monoinfected, HIV/HBV or HIV/HCV coinfected patients was obtained from patient’s medical records).

The RT and PR obtained sequences were analyzed using HIVdb: Genotypic Resistance Interpretation Algorithm, version 7.0, based in the *Panel of resistance of the International AIDS Society-USA* available on line in http://hivdb.stanford.edu. The report obtained from this analysis shows mutations in RT and PR genes and resistance data. The data about resistance are divided in High Level Resistance, Intermediate Resistance, Low level Resistance and Susceptible.

HIV subtype was determined by sequences (positions 2253–3290 of the HXB2) analysis using REGA HIV-1 Subtyping Tool 3.0, (http://www.bioafrica.net/subtypetool) and by RIP 3.0 - *Los Alamos Recombinant Identification Program* (http://www.hiv.lanl.gov/content/sequence/RIP/RIP.html).

All analyses were performed using PR sequences (codon 4–99) and TR (codon 38–247) due to used methodology.

### Patient’s Medical Records Review

Patients’ medical record review was performed by medical team of the Infectious Diseases Specialized Assistance Service, Botucatu Medical School, Sao Paulo State University, UNESP, Botucatu, SP, Brazil. From patients’ medical records was obtained information as gender, age, immunological condition (T CD4 count), HIV plasma viral load, HIV antiretroviral drugs exposition, HBV and HCV antiviral exposition, time of infection (HIV, HBV, HCV), AIDS presence (AIDS presence was defined according CDC parameters, AIDS was defined to CDC B and C stage). From medical records was also obtained the information about HIV monoinfection or HIV/HBV or HIV/HCV coinfections. This study was approved by the Research Ethics Committee of Botucatu Medical School, UNESP (Document number 430.610).

### Bioinformatic Analysis

The bioinformatics pipeline is reported in the Fig. 5 and, consists of supervised and unsupervised machine learning, patient and sequence clustering and molecular evolutionary analysis.

#### Supervised learning to create mono/co-infected model and medical records selection

The medical records from all patients (75 mono-infected and 34 coinfected, here called “instances”) were intersected in an attempt to extract features (here called “attributes”) presents in both classes of patients (mono- and co-infected). Thus, 71 attributes were selected, and two balanced datasets (both with 38 mono- and 34 co-infected instances) were obtained. Boolean attributes were assigned as binary for correct data interpretation (Supplementary Files 1 and 2). The C4.5 (J48) algorithm (C = 0.25, M = 2, pruned trees and 10-fold cross-validation parameters) was applied over both datasets to generate the decision trees, that were further analyzed by iterative pruning of relevant attributes until the statistics of classification were acceptable, giving us the most relevant set up attributes. Predictive models able to classify unlabeled instances (here called the “unknown dataset”) were obtained from the best machine learning training results (Supplementary Files 3 and 4). The most important statistics to evaluate the predictive performance of trainings were the Corrected Classified Instances (CCI) and Receiver Operating Characteristic (ROC) (Fig. 5a).

Some patients (15 individuals) were previously classified by clinical trials as mono-infected, but their positions in the phylogenetic tree (closely related to HIV of co-infected) indicated the likelihood of their being co-infected. Thus, classification of those 15 patients (Supplementary Files 5 and 6) using the two generated models (Supplementary Files 3 and 4) was performed. The second model was applied to only 13 patients since some instances were not available for two of the patients.The attribute transformation and supervised learning were performed using Weka v. 3.7.13 (The University of Waikato, Hamilton, New Zeland).

#### Filtering of gene sequences for molecular evolutionary analysis

Since we focused on correlating the medical records with molecular evolutionary analysis, we developed a pipeline for sequence selection (Fig. 5b–e) to ensure medical record and HIV sequence diversity in further analysis. The 289 mono-infected patients were clustered using an unsupervised learning maximization (EM) algorithm implemented in Weka v. 3.7.13; the dataset had the same 71 attributes previously mentioned. Next, the sequences of gag-pol (p6-p10, 288 bp), encoding the protease enzyme, and pol (p55-p66, 630 bp), encoding transcriptase, were selected for each cluster. This reduced the number of patients to be further evaluated since many did not have both sequences available (Fig. 5c). For each gene, the selected sequences were clustered by similarity using CD-HIT^55^, considering medical record clusters as well (Fig. 5d). The sequences of 75 patients (representing each CD-HIT cluster (Fig. 5e)), sequences from 31 co-infected patients and from one outgroup, were fused to compose the two datasets (one for each gene analyzed) for molecular evolutionary analysis (Fig. 5f). The sequences of the outgroup were selected from the Simian immunodeficiency virus isolate LB715 genome.

#### Molecular evolutionary analysis

The sequences for both genes were independently aligned using Muscle aligner and edited using SeaView 4.6.1^56^. The edited alignments were submitted to estimation of saturation using the saturation test by the Xia algorithm implemented in DAMBE 5.6.7 (Xia, 2013), with default parameters. The edited alignments were concatenated, and the best-fit evolutionary model was calculated using jModelTest2^57^ on XSEDE (11 substitution schemes, +f + i + g 4, −t ML, −S BEST, information criteria AICc). The phylogenetic reconstruction was determined by maximum likelihood using Phyml implemented in Seaview 4.6.1^56^. The parameters for phylogeny were GTR + I + G mode (4 rate categories, invariable sites at 0.4520 and fixed gamma shape at 1.1620), approximate likelihood ratio test (Shimodaira-Hasegawa-like), starting tree using BioNJ, and tree searching operation based on the best results of NNI and SPR. The tree was rooted and edited using the program FigTree v. 1.3.1.

The molecular clock rates were estimated only for protease by the software BEAST 1.8.3^58^, with the HKY+ uncorrelated relaxed clock model over 200 million generations, and the tree topology was fixed to the established by maximum likelihood.

The selective pressure analysis was performed using HYPHY^59^ with Branch-Site REL to look for episodic selection with an adaptive version of BS-REL and disallowing branch-site variation in synonymous rates.

### Data available

The data here presented are available to consult.

## Electronic supplementary material

Supplementary Information

Dataset 1

Dataset 2
