# Supplementary material for: HIV Reverse Transcriptase and Protease Genes Variability Can Be a Biomarker Associated with HIV and Hepatitis B or C Coinfection
Source: Sci Rep. 2018 May 29;8:8280. doi: 10.1038/s41598-018-26675-z (PMC5974300; doi:10.1038/s41598-018-26675-z)
Supplement: Supplementary file 1 — Supplementary Information [file 41598_2018_26675_MOESM1_ESM.pdf]

**Supplementary Information**

# HIV Reverse Transcriptase and Protease Genes Variability Can Be a Biomarker Associated with HIV and Hepatitis B or C Coinfection

Natália Mirele Cantão[1], Lauana Fogaça[2], Ivan Wolf[2], Rodrigo de Oliveira Almeida[2], Andressa Alves Cruz[1], Caroline Nunes[1], Alexandre Naime Barbosa[1], Guilherme Targino Valente[2], Maria Inês de Moura Campos Pardini[1], Rejane Maria Tommasini Grotto[1,2,*]
[1]São Paulo State University (Unesp), Medical School, Botucatu, Sao Paulo State, Brazil;
[2]São Paulo State University (Unesp), School of Agriculture (FCA), Department of Bioprocess and Biotechnology, Botucatu, Sao Paulo State, Brazil.
[*]regrotto@fca.unesp.br

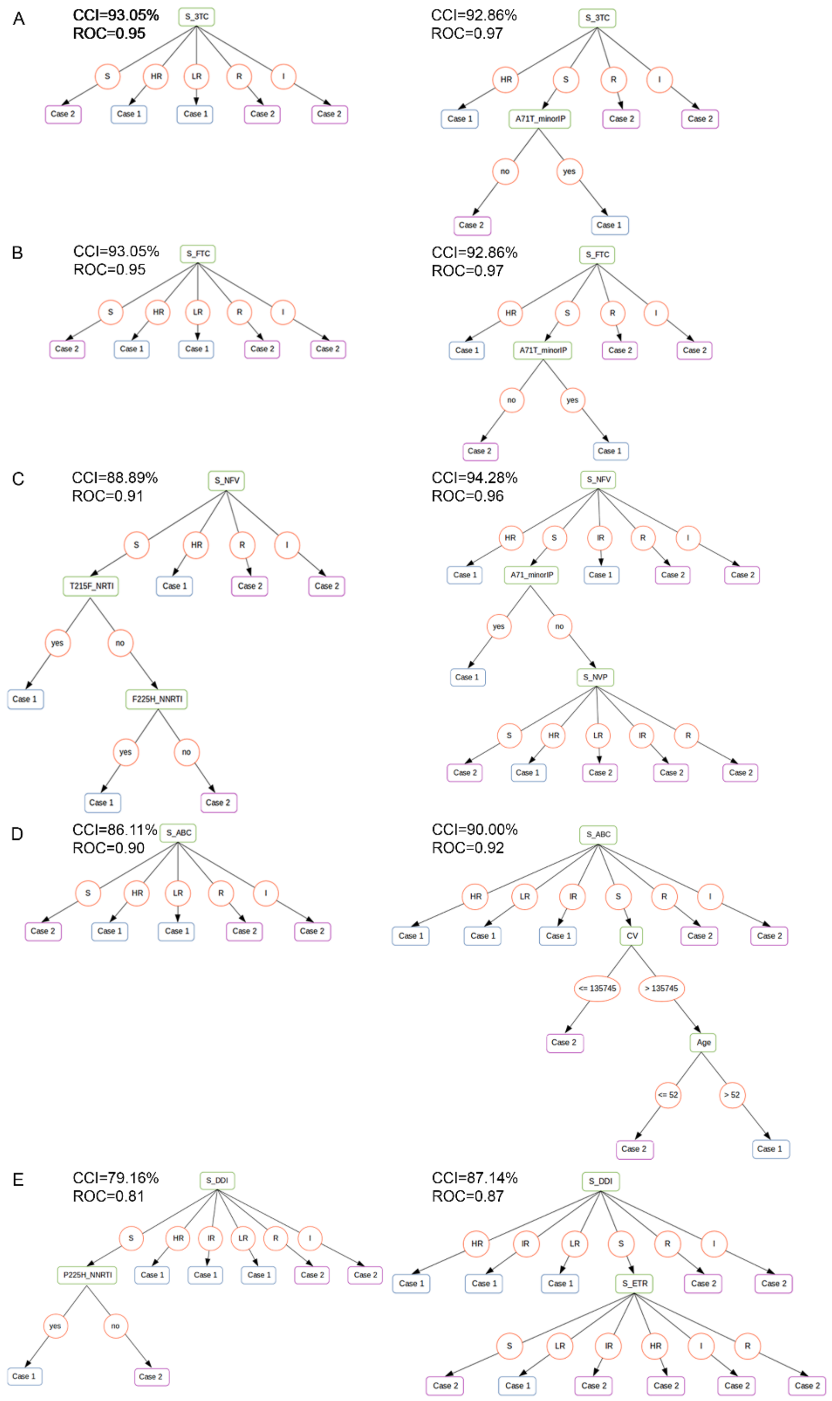


**Supplementary Figure 1 -** Decision trees generated by supervised learning. Left figures, tress generated using the dataset 1; Right figures, tress generated using the dataset 2; A, training with all attributes; B, training excluding S_3TC; C, training excluding S_3TC and S_FTC; D, training excluding S_3TC, S_FTC and S_NFV; E, training excluding S_3TC, S_FTC, S_NFV and S_ABC; Case 1, mono-infected; Case 2, co-infected. CCI, corrected classified instances; ROC, receiver operating characteristic.

**Supplementary File 1 –** Dataset 1 used to create mono/co-infected model 1. Weka file format (arff).

```
@relation Rej_R-1-
weka.filters.unsupervised.attribute.NumericToBinary-R60-70-
weka.filters.unsupervised.attribute.NumericToBinary-R7-42-
weka.filters.unsupervised.attribute.NumericToBinary-R5

@attribute AGE numeric
@attribute GENDER {F,M}
@attribute CV numeric
@attribute CD4 numeric
@attribute AIDS_cases_binarized {0,1}
@attribute PR_RT {BB,F1,BF,CC,KF,CB}
@attribute L24I_majorIP_binarized {0,1}
@attribute V32I_majorIP_binarized {0,1}
@attribute L33F_majorIP_binarized {0,1}
@attribute M46I_majorIP_binarized {0,1}
@attribute M46L_majorIP_binarized {0,1}
@attribute I54L_majorIP_binarized {0,1}
@attribute I54V_majorIP_binarized {0,1}
@attribute V82A_majorIP_binarized {0,1}
@attribute I84V_majorIP_binarized {0,1}
@attribute L90M_majorIP_binarized {0,1}
@attribute L10I_minorIP_binarized {0,1}
@attribute L10V_minorIP_binarized {0,1}
@attribute A71T_minorIP_binarized {0,1}
@attribute A71V_minorIP_binarized {0,1}
@attribute T74S_minorIP_binarized {0,1}
@attribute M41L_NRTI_binarized {0,1}
@attribute A62V_NRTI_binarized {0,1}
@attribute D67G_NRTI_binarized {0,1}
@attribute D67N_NRTI_binarized {0,1}
@attribute T69N_NRTI_binarized {0,1}
@attribute K70R_NRTI_binarized {0,1}
@attribute L74V_NRTI_binarized {0,1}
@attribute V75I_NRTI_binarized {0,1}
@attribute F116Y_NRTI_binarized {0,1}
@attribute V118I_NRTI_binarized {0,1}
@attribute Q151M_NRTI_binarized {0,1}
@attribute M184I_NRTI_binarized {0,1}
@attribute M184V_NRTI_binarized {0,1}
@attribute T215F_NRTI_binarized {0,1}
@attribute T215I_NRTI_binarized {0,1}
@attribute T215S_NRTI_binarized {0,1}
@attribute T215Y_NRTI_binarized {0,1}
@attribute K219E_NRTI_binarized {0,1}
@attribute K219Q_NRTI_binarized {0,1}
@attribute P225H_NNRTI_binarized {0,1}
@attribute M230L_NNRTI_binarized {0,1}
@attribute S_ATV_r {S,HR,IR,LR,R}
@attribute S_DRV_r {S,LR,IR,I}
@attribute S_FPV_r {S,IR,HR,LR,R}
@attribute S_IDV_r {S,HR,IR,LR,R,I}
```

```
@attribute S_LPV_r {S,HR,IR,LR,R,I}
@attribute S_NFV {S,HR,R,I}
@attribute S_SQV_r {S,HR,IR,LR,R,I}
@attribute S_TPV_r {S,LR,IR,HR,R,I}
@attribute S_3TC {S,HR,LR,R,I}
@attribute S_ABC {S,HR,LR,IR,R,I}
@attribute S_AZT {S,HR,IR,LR,I,R}
@attribute S_D4T {S,HR,IR,LR,I,R}
@attribute S_DDI {S,HR,IR,LR,R,I}
@attribute S_FTC {S,HR,LR,R,I}
@attribute S_TDF {S,LR,IR,HR,R,I}
@attribute S_EFV {HR,LR,S,IR,R,I}
@attribute S_ETR {LR,HR,S,IR,I,R}
@attribute X_3TC_use_binarized {0,1}
@attribute ATV_use_binarized {0,1}
@attribute ATV_r_use_binarized {0,1}
@attribute AZT_use_binarized {0,1}
@attribute D4T_use_binarized {0,1}
@attribute DDI_use_binarized {0,1}
@attribute DRV_r_use_binarized {0,1}
@attribute LPV_r_use_binarized {0,1}
@attribute NVP_use_binarized {0,1}
@attribute RTV_use_binarized {0,1}
@attribute TDF_use_binarized {0,1}
@attribute S_NVP {HR,LR,S,R}
@attribute Class {Mono,Co}

@data
40,F,167281,271,0,BB,0,0,0,0,0,0,0,0,0,0,0,0,0,0,0,0,0,0,0,0,0
,0,0,0,0,0,0,0,0,0,0,0,0,1,0,S,S,S,S,S,S,S,S,S,S,S,S,S,HR,LR
,1,0,0,1,0,0,0,0,0,0,0,HR,Mono
37,M,14370,334,0,BB,0,0,0,1,0,0,1,1,0,1,1,0,0,1,0,0,0,0,1,1,1,0,
0,0,0,0,0,1,1,0,0,0,0,1,0,0,HR,S,IR,HR,HR,HR,HR,LR,HR,HR,HR,HR,H
R,HR,LR,HR,HR,1,0,0,0,0,0,0,1,0,0,1,HR,Mono
34,F,33369,355,0,BB,0,0,0,0,0,1,0,0,0,1,0,1,0,1,0,0,0,1,0,0,1,0,
0,0,1,0,0,1,0,0,0,0,0,1,0,0,HR,LR,IR,HR,IR,HR,HR,S,HR,LR,IR,IR,I
R,HR,LR,HR,LR,1,0,1,0,0,0,0,0,0,0,1,HR,Mono
57,M,142639,8,1,F1,0,0,0,0,0,0,1,1,0,1,0,1,0,1,0,0,0,0,0,0,0,1,0
,0,0,0,0,1,0,0,0,0,0,0,0,0,HR,S,IR,HR,HR,HR,HR,IR,HR,HR,S,S,HR,H
R,S,HR,HR,1,0,0,0,0,1,0,1,0,0,0,HR,Mono
52,M,50865,321,0,BB,0,0,1,0,0,0,1,1,0,1,1,0,0,0,0,1,0,0,1,1,1,0,
0,0,1,0,0,1,1,0,0,0,1,0,0,0,HR,S,IR,HR,HR,HR,HR,IR,HR,IR,HR,HR,I
R,HR,IR,LR,S,1,0,0,0,0,0,0,1,0,0,1,LR,Mono
42,M,8080,121,1,BB,1,0,1,0,1,0,0,1,0,0,1,0,0,1,0,1,0,0,0,0,1,0,0
,0,0,0,0,1,0,0,0,1,1,0,0,0,IR,LR,HR,HR,HR,HR,IR,IR,HR,HR,HR,HR,H
R,HR,HR,HR,LR,1,0,0,1,0,0,0,0,0,0,1,HR,Mono
34,M,7667,139,1,BF,0,0,1,0,0,0,1,1,0,1,1,0,0,0,0,1,0,0,1,0,1,0,0
,0,1,0,0,1,1,0,0,0,0,1,0,0,HR,LR,HR,HR,HR,HR,HR,IR,HR,HR,HR,HR,H
R,HR,IR,S,S,1,0,0,1,0,0,0,1,0,0,1,LR,Mono
38,F,34075,583,1,BB,0,1,1,0,0,1,0,0,1,1,0,1,0,0,0,0,0,0,1,0,1,0,
0,0,0,0,0,1,1,0,0,0,0,1,0,0,HR,IR,HR,HR,IR,HR,HR,IR,HR,IR,HR,HR,
IR,HR,LR,HR,IR,1,0,0,0,0,0,0,0,0,0,1,HR,Mono
48,F,35448,328,0,BB,0,1,1,1,0,0,0,0,0,1,0,1,0,0,0,1,1,0,0,0,0,0,
0,0,0,0,0,1,0,0,0,1,0,0,0,0,HR,LR,HR,HR,IR,HR,IR,LR,HR,IR,IR,IR,
IR,HR,IR,S,S,1,0,1,0,0,0,0,0,0,0,1,S,Mono
```

32,M,11198,66,0,BB,0,0,0,0,0,0,0,0,0,0,0,0,0,0,0,0,0,1,1,1,0,0
,0,1,0,0,1,1,0,0,0,1,0,0,0,S,S,S,S,S,S,S,S,HR,IR,HR,HR,IR,HR,LR,
HR,IR,1,0,0,1,0,0,0,0,0,0,0,HR,Mono
30,M,53919,189,1,BF,0,0,0,0,0,0,0,0,0,0,0,0,0,0,1,0,0,0,0,0,0,
0,0,0,0,0,1,0,0,0,1,0,0,1,0,S,S,S,S,S,S,S,S,HR,HR,IR,IR,HR,HR,IR
,HR,LR,1,0,0,1,0,0,0,0,0,0,0,HR,Mono
40,F,258611,201,1,BB,0,0,0,1,0,0,1,0,0,1,0,0,0,1,0,1,0,0,1,0,0,0
,0,0,1,0,0,1,0,0,0,1,0,0,0,0,HR,S,IR,HR,HR,HR,HR,IR,HR,HR,HR,HR,
HR,HR,IR,HR,IR,1,0,0,0,0,0,0,1,0,0,1,HR,Mono
53,M,93995,34,1,BB,0,0,0,1,0,0,1,0,0,1,0,0,1,0,0,1,0,0,0,0,0,0,0
,0,1,0,0,1,0,0,0,1,0,0,1,0,HR,S,IR,HR,IR,HR,HR,LR,HR,HR,HR,HR,HR
,HR,IR,IR,LR,1,0,0,1,0,0,0,1,0,0,0,LR,Mono
46,M,15076,290,1,BB,0,0,0,1,1,0,1,0,0,0,0,1,0,1,1,1,0,0,1,0,0,0,
0,0,1,0,0,0,0,0,0,1,0,0,0,0,IR,S,IR,HR,IR,HR,IR,IR,LR,IR,HR,HR,H
R,LR,IR,HR,LR,0,0,0,0,1,1,0,1,0,0,0,HR,Mono
55,M,11983,124,1,BF,0,0,1,0,0,0,0,0,0,1,0,0,0,0,0,0,0,0,0,0,0,0,
0,0,0,0,0,1,0,0,0,0,0,0,0,0,HR,S,LR,IR,LR,HR,IR,LR,HR,LR,S,S,S,H
R,S,S,S,1,0,0,1,0,0,0,0,0,0,0,S,Mono
45,M,1704,478,1,BB,0,0,0,0,0,0,0,0,0,0,0,0,0,0,0,0,0,0,0,0,0,0,0
,0,0,0,0,0,0,0,0,0,0,0,0,0,S,S,S,S,S,S,S,S,S,S,S,S,S,S,S,S,S,1,0
,1,0,0,0,0,0,0,0,0,1,S,Mono
59,M,28993,194,1,BB,0,0,0,1,0,0,0,1,0,0,0,0,0,0,1,1,0,0,1,0,0,0,
0,0,0,0,0,1,0,0,0,1,0,0,0,0,IR,S,IR,HR,IR,HR,IR,IR,HR,HR,HR,HR,H
R,HR,IR,IR,IR,1,0,0,0,0,0,0,0,1,0,1,HR,Mono
49,F,140685,15,1,BB,0,0,0,1,0,0,1,1,0,1,1,0,0,1,0,1,0,0,1,0,0,0,
0,0,1,0,0,1,0,0,0,1,0,0,0,0,HR,LR,HR,HR,HR,HR,HR,IR,HR,HR,HR,HR,
HR,HR,IR,HR,HR,1,0,1,0,0,0,0,0,0,0,1,HR,Mono
43,M,4037,878,1,BB,0,0,0,0,0,0,0,0,0,0,0,0,0,0,0,1,0,0,0,1,1,0,0
,0,1,0,0,0,1,0,0,0,1,1,0,0,S,S,S,S,S,S,S,S,LR,HR,HR,HR,HR,LR,IR,
S,S,1,0,0,1,0,0,0,0,0,0,0,S,Mono
45,F,23725,57,0,F1,0,0,0,0,0,0,0,1,1,0,1,1,0,0,1,1,1,0,0,1,0,1,1,0
,0,1,0,0,1,1,0,0,0,0,1,0,0,IR,S,IR,HR,IR,HR,HR,LR,HR,HR,HR,HR,HR
,HR,IR,HR,IR,1,0,0,1,0,1,0,0,0,0,0,HR,Mono
49,F,8016,1056,0,BF,0,0,0,0,0,0,0,0,0,0,0,0,0,0,0,1,0,0,1,0,0,0,
0,0,1,0,0,1,0,0,0,1,0,0,0,0,S,S,S,S,S,S,S,S,HR,HR,HR,HR,HR,HR,IR
,IR,LR,1,0,0,1,0,0,0,0,0,0,0,HR,Mono
39,F,7695,26,1,BB,0,0,0,0,0,0,0,0,0,0,0,0,0,0,0,0,0,0,0,0,0,0,0,
0,0,0,0,1,1,0,0,0,0,0,0,0,S,S,S,S,S,S,S,S,HR,IR,LR,LR,LR,HR,S,S,
S,1,0,0,0,0,0,0,1,0,0,1,S,Mono
39,F,7886,129,0,BF,0,0,0,0,0,0,0,0,0,0,0,1,0,0,0,0,0,0,0,0,0,0,0
,0,0,0,0,1,0,0,0,0,0,0,1,0,S,S,S,S,S,S,S,S,HR,LR,S,S,S,HR,S,HR,L
R,1,1,0,1,0,0,0,0,0,0,0,HR,Mono
48,M,22987,214,1,BB,0,1,1,0,0,0,0,0,1,0,0,0,1,0,0,1,0,0,1,0,0,0,
0,0,1,0,0,0,0,0,0,1,0,0,0,0,HR,IR,HR,HR,IR,HR,IR,HR,LR,IR,HR,HR,
IR,LR,IR,S,S,0,0,0,1,0,1,0,1,0,0,0,S,Mono
44,F,305459,227,1,BB,0,0,1,1,0,0,1,0,0,1,0,1,1,0,0,1,0,0,1,1,1,0
,0,0,1,0,0,1,1,0,0,0,1,0,0,0,HR,LR,HR,HR,IR,HR,HR,IR,HR,HR,HR,HR
,HR,HR,IR,HR,LR,1,0,0,0,0,1,0,1,0,0,1,HR,Mono
65,M,10744,335,0,F1,0,0,0,0,1,0,1,0,0,0,1,0,0,0,0,0,0,0,1,0,1,0,
0,0,0,0,0,1,0,1,0,0,1,0,0,0,HR,S,IR,HR,IR,HR,HR,IR,HR,IR,HR,IR,L
R,HR,LR,S,S,1,0,0,1,0,0,0,1,0,0,1,S,Mono
51,M,51038,168,1,BB,1,0,1,0,1,1,1,1,1,0,1,0,0,1,0,1,0,0,1,0,0,0,
0,0,0,0,0,1,0,0,0,1,0,0,0,0,HR,IR,HR,HR,HR,HR,HR,IR,HR,IR,IR,IR,
IR,HR,IR,HR,LR,1,0,0,1,0,0,0,0,0,0,0,HR,Mono

37,F,201888,30,1,F1,0,0,0,0,0,0,1,0,1,0,1,0,0,0,0,0,0,0,0,0,0,0,
0,0,0,0,0,1,0,0,0,1,0,0,0,0,IR,LR,IR,IR,LR,HR,IR,IR,HR,IR,LR,LR,
LR,HR,LR,HR,LR,1,0,1,1,0,0,0,0,0,0,0,HR,Mono
32,F,41867,707,1,BB,0,0,0,0,1,0,0,0,0,0,0,1,0,0,0,1,0,0,1,1,1,0,
0,0,0,0,0,1,0,0,0,1,1,0,0,0,IR,LR,IR,LR,HR,HR,LR,LR,HR,HR,HR,HR,
HR,HR,IR,S,S,1,0,0,1,0,0,0,1,0,0,0,S,Mono
19,M,75506,10,1,BB,0,0,0,1,0,0,1,0,1,0,1,0,0,1,0,1,0,0,1,0,1,1,0
,0,1,0,0,1,0,0,0,1,1,0,0,0,HR,LR,HR,HR,HR,HR,HR,IR,HR,HR,HR,HR,H
R,HR,IR,HR,IR,1,0,0,0,0,0,0,1,0,0,1,HR,Mono
41,F,4883,321,1,BB,0,0,0,0,1,0,0,0,0,1,1,0,0,0,0,1,0,0,0,0,0,0,0
,0,0,0,0,1,0,0,0,1,0,0,0,0,IR,S,IR,IR,LR,HR,IR,LR,HR,HR,IR,IR,HR
,HR,IR,HR,HR,1,0,0,1,0,0,0,0,0,0,0,HR,Mono
36,M,21398,368,1,BB,0,0,1,1,0,0,0,1,0,1,0,0,1,1,0,0,0,0,0,0,0,0,
0,0,0,0,0,1,1,0,0,0,0,0,0,0,HR,S,IR,HR,IR,HR,HR,LR,HR,IR,LR,LR,L
R,HR,S,IR,IR,1,0,0,0,0,0,0,1,0,0,1,HR,Mono
43,M,40638,414,0,BB,0,1,1,1,0,0,0,1,1,0,0,0,0,1,0,1,0,0,0,0,0,0,
0,0,0,0,0,1,0,0,0,1,0,0,0,0,HR,IR,HR,HR,HR,HR,HR,IR,HR,IR,IR,IR,
IR,HR,IR,HR,IR,1,0,0,0,1,0,0,0,0,0,0,HR,Mono
42,M,28819,437,0,BF,0,0,0,0,0,0,0,0,0,0,0,0,0,0,0,0,0,0,0,1,1,0,
0,0,0,0,0,1,0,0,0,0,0,1,0,0,LR,S,S,S,S,HR,LR,S,HR,LR,LR,LR,LR,HR
,S,HR,IR,1,0,0,0,1,0,0,0,0,0,0,HR,Mono
31,F,190164,73,1,BB,0,1,1,0,1,0,0,0,0,0,0,1,1,0,0,0,1,0,0,0,0,0,
1,1,0,1,0,1,0,0,0,0,1,0,0,0,HR,LR,HR,IR,IR,HR,LR,LR,HR,HR,HR,HR,
HR,HR,HR,HR,HR,1,1,0,0,0,0,0,0,0,1,1,HR,Mono
46,M,15046,233,1,BB,1,0,0,1,0,0,1,1,0,0,0,0,1,0,0,0,0,0,1,0,1,0,
0,0,0,0,0,1,1,0,0,0,0,1,0,0,IR,S,IR,HR,IR,HR,IR,LR,HR,IR,HR,IR,I
R,HR,LR,S,S,1,0,0,1,0,0,0,1,0,0,0,S,Mono
38,M,31100,36,1,BB,0,1,1,1,0,0,0,1,0,1,0,1,1,0,0,1,0,0,1,0,1,0,0
,0,1,0,0,1,1,0,0,0,0,1,0,0,HR,IR,HR,HR,HR,HR,HR,HR,HR,HR,HR,HR,H
R,HR,IR,HR,HR,0,0,0,0,1,1,0,1,0,0,1,HR,Mono
58,F,39481,43,0,BB,0,0,0,0,0,0,0,0,0,1,0,0,0,0,0,0,1,0,0,0,0,0,1
,1,0,1,0,0,0,0,0,0,0,0,0,0,LR,S,LR,IR,LR,HR,IR,S,LR,IR,HR,HR,HR,
LR,LR,HR,LR,0,0,0,0,0,0,0,1,0,0,0,HR,Mono
57,M,45307,486,0,BB,0,0,0,0,0,0,0,0,0,0,0,0,0,0,0,0,0,0,0,1,1,0,
0,0,0,0,0,1,0,0,0,0,0,1,0,0,S,S,S,S,S,S,S,S,R,S,I,I,S,R,S,R,S,1,
0,1,0,1,0,0,0,0,0,0,R,Co
68,M,58115,170,1,BB,0,0,0,1,0,0,0,0,0,0,0,0,0,0,0,0,0,0,0,0,0,0,
0,0,0,0,0,0,0,0,0,0,0,0,0,0,S,S,S,S,S,S,S,S,S,S,S,S,S,S,S,S,S,0,
0,0,0,0,0,0,0,0,0,0,S,Co
45,F,130026,126,1,BB,0,0,0,0,0,0,0,0,0,0,0,0,0,0,0,0,0,0,0,0,0,0
,0,0,0,0,0,0,0,0,0,0,1,0,0,S,S,S,S,S,S,S,S,S,S,S,S,S,S,S,S,S,1
,0,1,1,0,0,0,0,0,0,0,S,Co
43,M,22761,117,1,BB,0,1,1,1,0,0,1,1,0,0,1,0,0,1,0,1,0,0,1,0,0,0,
0,0,0,0,0,1,0,0,0,1,1,0,1,0,R,S,R,R,R,R,R,R,R,R,R,R,R,R,R,R,S,1,
1,0,1,0,0,0,0,0,0,0,S,Co
34,F,5475,126,1,BB,0,0,0,0,0,0,0,0,0,0,0,0,0,0,0,0,0,0,0,0,0,0,0
,0,0,0,0,0,0,0,0,0,0,0,0,0,S,S,S,S,S,S,S,S,S,S,S,S,S,S,S,S,S,1,0
,0,1,0,0,0,1,0,0,0,S,Co
53,M,2865,146,1,BB,0,0,0,0,0,0,0,0,0,0,0,0,0,0,0,0,0,0,0,0,0,0,0
,0,0,0,0,0,0,0,0,0,0,0,0,0,S,S,S,S,S,S,S,S,S,S,S,S,S,S,S,S,S,1,0
,0,1,0,0,0,1,0,0,0,S,Co
44,M,75022,97,1,BB,0,0,0,0,0,0,0,0,0,0,0,0,0,0,0,0,0,0,0,0,0,0,0
,0,0,0,0,1,0,0,0,0,0,0,0,0,S,S,S,S,S,S,S,S,R,I,S,S,I,R,S,R,I,1,0
,0,1,0,1,0,0,0,1,0,R,Co

42,M,15233,156,1,BB,0,0,0,0,0,0,0,0,0,0,0,0,0,0,0,0,0,0,0,0,0,0,
0,0,0,0,0,0,0,0,0,0,0,0,0,0,S,S,S,S,S,S,S,S,S,S,S,S,S,S,S,1,
0,0,0,0,0,1,0,0,0,1,S,Co
44,F,1951,197,1,BB,0,0,0,0,0,0,1,1,0,1,1,0,1,0,0,0,0,1,0,0,1,0,0
,0,0,0,1,1,1,1,1,0,1,0,0,0,R,S,R,R,R,I,R,S,R,S,I,I,S,R,S,R,S,1,0
,0,0,0,0,0,1,0,0,1,R,Co
48,M,206459,80,1,BB,0,0,0,0,0,0,0,0,0,0,0,0,0,0,0,0,0,0,0,0,1,0,
0,0,0,0,0,1,0,0,0,0,0,1,0,0,S,S,S,S,S,S,S,S,R,S,S,S,S,R,S,I,S,1,
0,0,1,0,0,0,0,1,0,0,R,Co
63,M,10831,438,1,BB,1,0,0,0,1,1,0,1,1,0,1,1,0,0,1,0,0,0,0,0,1,0,
0,0,0,0,0,1,0,0,0,0,0,1,1,0,R,I,R,R,R,R,R,S,R,S,I,I,S,R,S,R,S,1,
0,0,0,0,0,0,0,0,0,1,R,Co
43,M,11791,102,1,BB,0,0,0,0,0,0,0,0,0,0,0,0,0,0,0,0,0,0,0,0,0,0,
0,0,0,0,0,1,0,0,0,0,0,0,0,0,S,S,S,S,S,S,S,S,R,S,S,S,S,R,S,R,R,1,
0,0,1,0,0,0,0,0,0,0,R,Co
39,M,3380,420,1,BB,0,0,0,0,0,0,0,0,0,0,0,0,0,0,0,0,0,0,0,0,0,0,0
,0,0,0,0,1,0,0,0,0,0,0,0,0,S,S,S,S,S,S,S,R,S,S,S,S,R,S,S,S,1,0
,1,1,0,0,0,0,0,0,0,S,Co
39,F,720,837,1,BB,0,0,0,0,0,0,0,0,0,0,0,0,0,0,0,0,0,0,0,0,0,1,0,
0,0,0,0,1,0,0,0,0,1,0,0,1,S,S,S,S,S,S,S,S,R,R,S,S,R,R,S,R,R,1,0,
0,0,0,0,0,0,0,0,1,R,Co
46,M,205287,366,1,BB,0,0,0,1,0,0,1,0,0,1,0,0,0,0,0,0,1,0,0,1,0,0
,1,1,1,1,0,0,0,0,0,0,1,0,0,0,R,I,S,R,R,S,I,I,I,R,R,R,R,I,I,R,I,1
,0,0,0,0,0,1,0,0,0,1,R,Co
39,F,2966,148,1,BB,0,0,0,0,0,0,0,0,0,0,0,0,0,0,0,0,0,0,0,0,0,0,0
,0,0,0,0,1,0,0,0,0,0,0,0,0,S,S,S,S,S,S,S,R,S,S,S,S,R,S,R,S,1,0
,0,0,0,0,0,0,0,0,1,R,Co
36,F,50218,89,1,BB,0,0,0,0,0,0,0,0,0,0,0,0,0,0,0,0,0,0,0,0,0,0,0
,0,0,0,0,0,0,0,0,0,0,0,0,0,S,S,S,S,S,S,S,S,S,S,S,S,S,S,S,1,0
,0,1,0,0,0,0,0,0,0,S,Co
34,F,1016,76,1,F1,0,0,0,0,0,0,0,0,0,0,0,0,0,0,0,0,0,0,0,0,0,0,0,
0,0,0,0,0,0,0,0,0,0,0,0,0,S,S,S,S,S,S,S,S,S,S,S,S,S,S,S,1,0,
0,1,0,0,0,1,0,0,0,S,Co
35,M,2861,154,1,BB,0,0,0,0,0,0,0,0,0,0,0,1,0,0,0,0,0,0,0,0,0,0,0
,0,0,0,0,1,0,0,0,0,0,0,0,0,S,S,S,S,S,S,S,R,S,S,S,S,R,S,R,I,1,0
,0,0,1,0,0,0,0,0,0,R,Co
50,M,49,956,1,BB,0,0,0,0,0,0,0,0,0,0,0,1,0,0,0,1,0,0,0,0,0,0,0,0
,0,0,0,0,0,0,0,0,0,0,0,0,S,S,S,S,S,S,S,S,S,LR,IR,IR,LR,S,LR,S,S,
1,0,0,0,0,0,0,1,0,0,1,S,Co
50,M,49,427,1,CC,0,0,0,0,0,0,0,0,0,0,0,0,0,0,0,0,0,0,0,0,0,0,0,0,0
,0,0,0,0,0,0,0,0,0,0,0,0,S,S,S,S,S,S,S,S,S,S,S,S,S,S,S,S,1,0,0
,0,0,0,0,0,0,0,0,1,S,Co
40,M,49,666,0,BB,0,0,0,0,0,0,0,0,0,0,0,0,0,0,0,0,0,0,0,0,0,0,0,0
,0,0,0,0,0,0,0,0,0,0,0,0,S,S,S,S,S,S,S,S,S,S,S,S,S,S,S,S,1,0,0
,0,0,0,0,0,0,0,0,1,S,Co
47,M,49,477,1,BB,0,0,0,0,0,0,0,0,0,0,0,0,0,0,0,0,0,0,0,0,0,0,0,0
,0,0,0,0,0,0,0,0,0,0,0,0,S,S,S,S,S,S,S,S,S,S,S,S,S,S,S,S,1,0,0
,1,0,0,0,1,0,0,0,S,Co
41,F,49,1058,0,BB,0,0,0,0,0,0,0,0,0,0,1,0,0,0,0,0,0,0,0,0,0,0,0,
0,0,0,0,0,0,0,0,0,0,0,0,0,S,S,S,S,S,S,S,S,S,S,S,S,S,S,S,S,1,0,
0,0,0,0,0,0,1,0,1,S,Co
38,M,72273,290,0,KF,0,0,0,0,0,0,0,0,0,0,0,0,0,0,0,0,0,0,0,0,0,0,
0,0,0,0,0,0,0,0,0,0,0,0,0,0,S,S,S,S,S,S,S,S,S,S,S,S,S,S,S,S,0,
0,0,0,0,0,0,0,0,0,0,S,Co

```
54,M,116,300,1,BB,0,0,0,0,0,0,0,0,0,0,1,0,0,0,0,0,0,0,0,0,0,0,0,
0,0,0,0,0,0,0,0,0,0,0,0,0,S,S,S,S,S,S,S,S,S,S,S,S,S,S,S,LR,S,1,0
,0,0,0,0,0,1,0,0,1,LR,Co
36,M,49,460,0,CB,0,0,0,0,0,0,0,0,0,0,0,1,0,0,0,0,0,0,0,0,0,0,0,0
,0,0,0,0,0,0,0,0,0,0,0,0,S,S,S,S,S,S,S,S,S,S,S,S,S,S,S,S,S,1,0,0
,0,0,0,0,0,0,0,1,S,Co
40,M,16069,149,1,BB,0,0,0,0,0,0,0,0,0,0,0,0,0,0,0,0,0,0,0,0,0,0,
0,0,0,0,0,0,0,0,0,0,0,0,0,0,S,S,S,S,S,S,S,S,S,S,S,S,S,S,S,S,S,0,
0,0,0,0,0,0,0,0,0,0,S,Co
38,M,7950,382,0,BB,0,0,0,0,0,0,0,0,0,0,0,0,0,0,0,0,0,0,0,0,0,0,0
,0,0,0,0,0,0,0,0,0,0,0,0,0,S,S,S,S,S,S,S,S,S,S,S,S,S,S,S,S,S,1,0
,0,0,0,0,0,0,0,0,1,S,Co
36,M,266198,148,1,BB,0,0,0,0,0,0,0,0,0,0,0,0,0,0,0,0,0,0,0,0,0,0
,0,0,0,0,0,0,0,0,0,0,0,0,0,0,S,S,S,S,S,S,S,S,S,S,S,S,S,S,S,S,S,0
,0,0,0,0,0,0,0,0,0,0,S,Co
44,F,49,854,0,BB,0,0,0,0,0,0,0,0,0,0,1,0,0,0,0,0,0,0,0,0,0,0,0,0
,0,0,0,0,0,0,0,0,0,0,0,0,S,S,S,S,S,S,S,S,S,S,S,S,S,S,S,LR,S,1,0,
0,0,0,0,0,0,0,0,1,LR,Co
54,M,12726,361,1,BB,0,0,0,0,0,0,0,0,0,0,0,0,0,0,0,0,0,0,0,0,0,0,
0,0,0,0,0,0,0,0,0,0,0,0,0,0,S,S,S,S,S,S,S,S,S,S,S,S,S,S,S,S,S,0,
0,0,0,0,0,0,0,0,0,0,S,Co
46,M,49,490,1,CC,0,0,0,0,0,0,0,0,0,0,0,0,0,0,0,0,0,0,0,0,0,0,0,0
,0,0,0,0,0,0,0,0,0,0,0,0,S,S,S,S,S,S,S,S,S,S,S,S,S,S,S,S,LR,1,0,
0,0,0,0,0,0,0,0,1,S,Co
49,F,49,864,0,BB,0,0,0,0,0,0,0,1,0,0,0,0,0,0,0,0,0,0,0,0,0,0,0,0
,0,0,0,0,0,0,0,0,0,0,0,0,LR,S,LR,I,I,I,LR,S,S,S,S,S,S,S,S,S,S,1,
0,0,1,0,0,0,1,0,0,0,S,Co
```

**Supplementary File 2** – Dataset 2 used to create mono/co-infected model 1. Weka file format (arff).

```
relation Rej_R-2-
weka.filters.unsupervised.attribute.NumericToBinary-R60-70-
weka.filters.unsupervised.attribute.NumericToBinary-R5-
weka.filters.unsupervised.attribute.NumericToBinary-R7-42-
weka.filters.unsupervised.attribute.NumericToBinary-R60-70

@attribute AGE numeric
@attribute GENDER {M,F}
@attribute CV numeric
@attribute CD4 numeric
@attribute AIDS_case_binarized {0,1}
@attribute PR_RT {BB,F1,BF,CC,KF,CB}
@attribute L24I_majorIP_binarized {0,1}
@attribute V32I_majorIP_binarized {0,1}
@attribute L33F_majorIP_binarized {0,1}
@attribute M46I_majorIP_binarized {0,1}
@attribute M46L_majorIP_binarized {0,1}
@attribute I54L_majorIP_binarized {0,1}
@attribute I54V_majorIP_binarized {0,1}
@attribute V82A_majorIP_binarized {0,1}
@attribute I84V_majorIP_binarized {0,1}
@attribute L90M_majorIP_binarized {0,1}
```

```
@attribute L10I_minorIP_binarized {0,1}
@attribute L10V_minorIP_binarized {0,1}
@attribute A71T_minorIP_binarized {0,1}
@attribute A71V_minorIP_binarized {0,1}
@attribute T74S_minorIP_binarized {0,1}
@attribute M41L_NRTI_binarized {0,1}
@attribute A62V_NRTI_binarized {0,1}
@attribute D67G_NRTI_binarized {0,1}
@attribute D67N_NRTI_binarized {0,1}
@attribute T69N_NRTI_binarized {0,1}
@attribute K70R_NRTI_binarized {0,1}
@attribute L74V_NRTI_binarized {0,1}
@attribute V75I_NRTI_binarized {0,1}
@attribute F116Y_NRTI_binarized {0,1}
@attribute V118I_NRTI_binarized {0,1}
@attribute Q151M_NRTI_binarized {0,1}
@attribute M184I_NRTI_binarized {0,1}
@attribute M184V_NRTI_binarized {0,1}
@attribute T215F_NRTI_binarized {0,1}
@attribute T215I_NRTI_binarized {0,1}
@attribute T215S_NRTI_binarized {0,1}
@attribute T215Y_NRTI_binarized {0,1}
@attribute K219E_NRTI_binarized {0,1}
@attribute K219Q_NRTI_binarized {0,1}
@attribute P225H_NNRTI_binarized {0,1}
@attribute M230L_NNRTI_binarized {0,1}
@attribute S_ATV_r {HR,S,IR,R,LR}
@attribute S_DRV_r {S,IR,LR,I}
@attribute S_FPV_r {IR,S,LR,HR,R}
@attribute S_IDV_r {HR,S,IR,LR,R,I}
@attribute S_LPV_r {IR,S,LR,HR,R,I}
@attribute S_NFV {HR,S,IR,R,I}
@attribute S_SQV_r {IR,S,HR,LR,R,I}
@attribute S_TPV_r {IR,S,LR,R,I}
@attribute S_3TC {HR,S,R,I}
@attribute S_ABC {HR,LR,IR,S,R,I}
@attribute S_AZT {HR,IR,S,LR,I,R}
@attribute S_D4T {HR,LR,S,IR,I,R}
@attribute S_DDI {HR,IR,LR,S,R,I}
@attribute S_FTC {HR,S,R,I}
@attribute S_TDF {IR,LR,S,R,I}
@attribute S_EFV {S,HR,IR,LR,R,I}
@attribute S_ETR {S,LR,IR,HR,I,R}
@attribute X_3TC_use_binarized {0,1}
@attribute ATV_use_binarized {0,1}
@attribute ATV_r_use_binarized {0,1}
@attribute AZT_use_binarized {0,1}
@attribute D4T_use_binarized {0,1}
@attribute DDI_use_binarized {0,1}
@attribute DRV_r_use_binarized {0,1}
@attribute LPV_r_use_binarized {0,1}
@attribute NVP_use_binarized {0,1}
@attribute RTV_use_binarized {0,1}
@attribute TDF_use_binarized {0,1}
@attribute S_NVP {S,HR,LR,IR,R}
@attribute Class {Mono,Co}
```

@data
45,M,10920,242,1,BB,1,0,1,1,0,0,1,1,0,0,1,0,0,1,0,1,0,0,1,1,1,0,
0,0,0,0,0,1,1,0,0,0,1,0,0,0,HR,S,IR,HR,IR,HR,IR,IR,HR,HR,HR,HR,H
R,HR,IR,S,S,1,0,1,1,0,0,0,0,0,1,S,Mono
42,M,3048,439,1,BB,0,0,0,0,0,0,0,0,0,0,0,0,0,0,1,0,0,0,1,0,0,0
,0,0,0,0,1,0,0,0,1,0,0,0,0,S,S,S,S,S,S,S,S,HR,HR,HR,HR,HR,HR,IR,
HR,LR,1,0,0,0,0,0,0,0,0,0,1,HR,Mono
40,F,11276,576,1,F1,0,0,0,0,0,0,0,0,0,1,0,1,0,1,0,1,0,1,0,0,1,0,
0,0,0,0,0,1,1,0,0,0,1,0,0,0,HR,S,LR,IR,LR,HR,IR,S,HR,HR,HR,HR,HR
,HR,LR,IR,LR,1,1,0,0,0,0,0,0,0,0,1,HR,Mono
38,F,135745,239,1,BB,0,1,1,0,0,1,0,1,0,0,0,0,1,0,0,1,0,0,1,0,0,0
,0,0,0,0,0,1,0,0,0,1,0,0,0,0,HR,IR,HR,HR,HR,HR,IR,IR,HR,HR,HR,HR
,IR,HR,IR,HR,IR,1,0,0,0,0,0,0,1,0,0,1,HR,Mono
43,F,5004,437,0,F1,0,0,0,0,0,0,0,0,0,0,0,0,0,0,0,0,0,1,0,1,0,0
,0,0,0,0,1,0,0,0,0,0,1,0,0,HR,S,S,S,S,IR,S,S,HR,LR,IR,LR,LR,HR,S
,HR,IR,1,0,0,1,0,0,0,0,0,0,0,HR,Mono
43,M,7792,246,0,BB,0,0,0,0,0,0,0,0,0,0,0,1,0,0,1,0,1,0,0,1,0,0
,0,0,0,0,1,0,0,0,1,0,1,0,0,S,S,S,S,S,S,S,S,HR,HR,HR,HR,HR,HR,IR,
HR,LR,1,0,0,1,0,0,0,0,0,0,0,HR,Mono
37,M,5300,819,0,BB,0,0,0,0,0,0,0,0,0,0,1,1,0,0,0,0,0,0,0,0,0,0
,0,0,0,0,1,0,0,0,0,0,0,1,0,S,S,S,S,S,S,S,S,HR,LR,S,S,S,HR,S,HR,L
R,1,0,0,1,0,0,0,0,0,0,0,HR,Mono
38,F,2864,432,0,BB,0,0,0,0,0,0,1,1,0,1,1,0,0,1,0,1,0,0,0,0,0,0
,0,0,0,0,1,0,0,0,1,0,0,0,0,HR,S,IR,HR,IR,HR,HR,LR,HR,IR,IR,IR,IR
,HR,LR,HR,LR,1,0,0,1,0,0,0,0,1,0,0,HR,Mono
57,M,197720,46,1,BB,0,0,0,0,0,0,0,0,0,0,0,0,0,0,0,0,0,0,0,0,0,0,
0,0,0,0,0,0,0,0,0,0,0,0,0,0,S,S,S,S,S,S,S,S,S,S,S,S,S,S,S,S,S,1,
0,0,1,0,0,0,1,0,0,1,S,Mono
56,F,3167,88,1,BB,0,0,0,1,0,1,0,1,0,1,1,0,0,1,0,0,0,0,1,1,1,0,0,
0,0,0,0,1,1,0,0,0,1,0,0,0,HR,LR,HR,HR,HR,HR,HR,S,HR,IR,HR,HR,IR,
HR,LR,HR,HR,1,0,0,0,0,0,0,0,0,0,1,HR,Mono
65,M,3090,130,0,BB,0,0,1,0,0,0,0,1,0,0,0,1,0,1,0,1,0,0,1,0,1,0,0
,0,0,0,0,1,1,0,0,0,1,0,0,0,HR,S,IR,HR,IR,HR,HR,IR,HR,IR,HR,HR,IR
,HR,LR,S,S,1,0,0,1,0,0,0,1,0,0,1,LR,Mono
59,M,12707,498,1,BB,0,0,0,0,1,0,1,1,0,1,0,1,0,1,0,0,0,0,1,0,1,0,
0,0,1,0,0,1,1,0,0,0,1,0,0,0,HR,S,IR,HR,HR,HR,HR,IR,HR,IR,HR,IR,I
R,HR,LR,HR,IR,1,0,0,1,0,1,0,1,0,0,0,HR,Mono
41,M,3802,205,0,BB,0,0,0,0,0,0,0,0,0,0,0,0,0,0,0,0,0,0,0,0,0,0
,0,0,0,0,1,0,0,0,0,0,0,0,0,S,S,S,S,S,S,S,S,HR,LR,S,S,S,HR,S,HR,L
R,1,0,0,1,0,0,0,0,1,0,0,HR,Mono
38,M,19376,63,1,BF,1,0,0,0,1,0,1,1,0,0,1,0,0,1,0,1,0,0,1,0,0,0,0
,0,1,0,0,1,0,0,0,1,0,0,0,0,HR,LR,IR,HR,IR,HR,IR,LR,HR,HR,HR,HR,H
R,HR,IR,IR,LR,1,0,1,0,0,0,0,0,0,0,1,HR,Mono
62,M,6553,426,0,BB,1,0,0,1,0,0,1,0,0,0,1,1,0,1,1,0,0,0,1,0,1,0,0
,0,0,0,0,1,1,0,0,0,0,1,0,0,IR,S,IR,HR,IR,HR,IR,IR,HR,IR,HR,IR,IR
,HR,LR,S,S,1,0,0,0,0,0,0,1,0,0,1,S,Mono
56,M,23442,266,1,BB,0,0,1,0,0,0,1,1,1,1,1,0,0,1,0,1,0,0,1,0,0,0,
0,0,1,0,0,1,0,0,0,1,1,0,1,0,HR,LR,HR,HR,HR,HR,HR,IR,HR,HR,HR,HR,
HR,HR,IR,HR,LR,1,0,0,0,0,0,0,1,0,0,1,HR,Mono
48,F,97393,99,1,BB,0,0,1,1,0,0,0,1,0,0,0,0,1,0,0,1,0,0,0,1,0,0,0
,0,0,0,0,1,1,0,0,0,0,0,0,0,HR,S,IR,IR,IR,HR,LR,IR,HR,HR,HR,HR,HR
,HR,IR,S,S,1,0,0,0,0,0,0,1,0,0,1,LR,Mono
47,F,18474,736,0,BF,0,0,1,0,0,0,1,1,0,1,1,0,0,0,0,0,0,0,1,0,1,0,
0,0,0,0,0,1,0,0,0,0,1,0,0,0,IR,S,IR,HR,IR,HR,HR,IR,HR,LR,IR,LR,L
R,HR,S,S,S,1,0,0,0,0,0,0,0,0,0,1,S,Mono

48,M,231791,510,1,BB,0,0,1,0,1,0,0,1,0,0,0,0,0,0,0,0,0,0,1,0,1,0
,0,0,0,0,0,1,1,0,0,0,0,0,1,1,HR,S,LR,IR,IR,HR,LR,LR,HR,IR,HR,IR,
IR,HR,LR,HR,IR,1,0,1,0,0,0,0,0,0,0,1,HR,Mono
63,M,8188,353,0,BB,0,0,0,0,0,0,0,0,0,0,0,0,0,1,0,0,0,0,1,0,1,0,0
,0,0,0,0,1,0,0,0,0,0,1,0,0,S,S,S,S,S,S,S,S,HR,IR,IR,LR,IR,HR,S,I
R,LR,1,0,0,0,0,0,0,0,1,0,1,HR,Mono
38,M,11962,695,0,BB,0,0,0,0,0,0,1,1,0,0,0,1,0,1,1,1,0,0,0,0,0,0,
0,0,0,0,0,1,0,0,0,1,0,0,0,0,IR,S,LR,IR,IR,HR,LR,LR,HR,IR,IR,IR,I
R,HR,LR,S,S,1,0,0,0,1,0,0,0,0,0,0,S,Mono
50,F,71632,340,1,BB,1,0,0,1,0,0,0,1,0,0,1,0,0,1,0,0,1,0,0,0,0,0,
0,0,0,0,0,1,0,0,0,0,0,1,0,0,HR,S,IR,IR,IR,HR,IR,S,HR,HR,S,LR,IR,
HR,IR,S,S,1,0,1,0,0,0,0,0,0,0,1,S,Mono
38,F,9924,53,1,BB,0,0,0,0,0,0,0,0,0,1,1,0,0,0,0,0,0,0,1,0,1,0,0,
0,0,0,0,1,0,0,0,1,0,1,0,0,IR,S,LR,IR,LR,HR,HR,S,HR,IR,HR,IR,IR,H
R,LR,IR,IR,1,0,1,0,0,0,0,0,0,0,1,HR,Mono
34,M,3087,591,0,BB,0,0,0,0,0,0,0,0,0,0,0,0,1,0,0,1,0,0,1,0,0,0,0
,0,0,0,0,0,1,0,0,0,0,1,0,0,S,S,S,S,S,S,S,S,IR,HR,HR,IR,S,LR,S,
S,1,0,0,0,0,0,0,1,0,0,1,S,Mono
37,F,19658,239,0,BB,0,0,0,0,0,0,0,0,0,0,0,1,0,0,0,0,0,0,0,0,0,
0,0,0,0,0,0,0,0,0,0,0,0,0,0,S,S,S,S,S,S,S,S,S,S,S,S,S,S,S,S,1,
1,0,1,0,0,0,0,0,0,0,0,S,Mono
43,M,112936,452,0,BF,0,1,0,1,0,1,1,0,0,0,0,1,0,1,1,0,0,0,1,1,1,0
,0,0,1,0,0,1,1,0,0,0,1,0,0,0,HR,IR,HR,IR,IR,HR,IR,IR,HR,IR,HR,HR
,IR,HR,LR,HR,LR,1,0,0,1,0,0,0,1,0,0,0,HR,Mono
39,M,6216,125,1,BF,0,0,0,0,0,0,0,0,0,0,0,0,0,0,0,1,0,0,0,0,0,0,0
,0,0,0,0,1,1,0,0,1,0,0,1,0,S,S,S,S,S,S,S,HR,IR,IR,IR,IR,HR,LR,
HR,LR,1,0,0,1,0,0,0,0,0,0,0,0,HR,Mono
42,F,12404,220,0,BB,0,0,0,0,0,0,0,0,0,0,0,0,0,0,0,0,0,0,0,0,0,0,
0,0,0,0,0,1,0,0,0,0,0,0,0,0,S,S,S,S,S,S,S,S,HR,LR,S,S,S,HR,S,HR,
LR,1,0,0,1,0,0,0,0,1,0,0,HR,Mono
47,M,5250,412,0,BB,1,0,0,1,0,1,0,0,0,0,0,0,0,0,0,1,0,0,0,0,0,0,0
,0,0,0,0,1,0,0,0,1,0,0,0,0,IR,IR,HR,HR,HR,HR,LR,S,HR,HR,IR,IR,HR
,HR,IR,HR,IR,1,0,0,0,0,0,0,0,0,0,1,HR,Mono
58,M,316620,15,1,BB,0,0,0,0,0,1,0,0,0,1,1,0,0,1,0,1,0,0,1,1,1,0,
0,0,0,0,0,1,0,0,0,1,1,0,0,0,IR,LR,IR,IR,LR,HR,IR,S,HR,HR,HR,HR,H
R,HR,IR,S,S,1,1,0,1,0,0,0,0,0,0,0,S,Mono
36,F,2068,314,0,F1,0,0,0,0,0,0,1,0,0,1,0,1,0,1,0,0,0,1,0,0,0,0,0
,0,0,0,0,1,0,0,0,0,0,0,0,0,IR,S,IR,IR,LR,HR,HR,LR,HR,IR,LR,LR,IR
,HR,LR,S,S,1,0,1,0,0,0,0,0,0,0,1,S,Mono
36,M,7331,258,1,BB,1,0,0,1,0,0,1,1,0,0,1,0,0,0,1,0,0,0,1,1,1,0,0
,0,1,0,0,1,1,0,0,0,0,1,0,0,IR,S,IR,HR,IR,HR,IR,IR,HR,IR,HR,HR,IR
,HR,LR,S,S,1,0,0,1,0,0,0,1,0,0,0,LR,Mono
42,M,51455,320,1,BF,0,0,0,0,0,0,0,0,0,1,0,0,1,0,1,1,0,0,0,0,0,0,
0,0,0,0,0,1,0,0,0,1,0,1,0,0,IR,S,LR,IR,LR,HR,IR,S,HR,HR,HR,HR,HR
,HR,IR,S,S,1,0,1,0,0,0,0,0,0,0,1,S,Mono
65,F,195313,107,1,F1,0,0,0,0,0,0,0,0,0,0,0,0,0,0,0,0,0,0,0,0,0,0,0
,0,0,0,0,0,0,0,0,0,0,0,0,0,S,S,S,S,S,S,S,S,S,S,S,S,S,S,S,HR,LR
,1,0,1,0,0,0,0,0,0,0,1,HR,Mono
52,M,15739,739,0,BB,0,0,0,0,0,0,1,0,0,0,0,0,0,0,0,1,0,1,0,0,1,0,
0,0,0,0,0,1,0,0,0,1,1,0,0,0,IR,S,LR,LR,LR,HR,LR,LR,HR,IR,HR,HR,I
R,HR,IR,LR,S,1,0,1,0,0,0,0,0,0,0,1,IR,Mono
55,F,20848,224,0,BF,0,1,0,1,0,0,1,0,0,1,1,0,0,1,0,1,0,0,1,0,0,0,
0,0,0,0,0,1,0,0,0,1,0,0,0,0,HR,LR,HR,HR,IR,HR,HR,IR,HR,HR,HR,HR,
IR,HR,IR,S,S,1,0,1,0,0,0,0,0,0,0,1,S,Mono

57,M,45307,486,0,BB,0,0,0,0,0,0,0,0,0,0,0,0,0,0,0,0,0,0,0,1,1,0,
0,0,0,0,0,1,0,0,0,0,0,1,0,0,S,S,S,S,S,S,S,S,R,S,I,I,S,R,S,R,S,1,
0,1,0,1,0,0,0,0,0,0,R,Co
68,M,58115,170,1,BB,0,0,0,1,0,0,0,0,0,0,0,0,0,0,0,0,0,0,0,0,0,0,
0,0,0,0,0,0,0,0,0,0,0,0,0,0,S,S,S,S,S,S,S,S,S,S,S,S,S,S,S,S,S,0,
0,0,0,0,0,0,0,0,0,0,S,Co
45,F,130026,126,1,BB,0,0,0,0,0,0,0,0,0,0,0,0,0,0,0,0,0,0,0,0,0,0
,0,0,0,0,0,0,0,0,0,0,0,1,0,0,S,S,S,S,S,S,S,S,S,S,S,S,S,S,S,S,S,1
,0,1,1,0,0,0,0,0,0,0,S,Co
43,M,22761,117,1,BB,0,1,1,1,0,0,1,1,0,0,1,0,0,1,0,1,0,0,1,0,0,0,
0,0,0,0,0,1,0,0,0,1,1,0,1,0,R,S,R,R,R,R,R,R,R,R,R,R,R,R,R,R,S,1,
1,0,1,0,0,0,0,0,0,0,S,Co
34,F,5475,126,1,BB,0,0,0,0,0,0,0,0,0,0,0,0,0,0,0,0,0,0,0,0,0,0,0
,0,0,0,0,0,0,0,0,0,0,0,0,0,S,S,S,S,S,S,S,S,S,S,S,S,S,S,S,S,S,1,0
,0,1,0,0,0,1,0,0,0,S,Co
53,M,2865,146,1,BB,0,0,0,0,0,0,0,0,0,0,0,0,0,0,0,0,0,0,0,0,0,0,0
,0,0,0,0,0,0,0,0,0,0,0,0,0,S,S,S,S,S,S,S,S,S,S,S,S,S,S,S,S,S,1,0
,0,1,0,0,0,1,0,0,0,S,Co
44,M,75022,97,1,BB,0,0,0,0,0,0,0,0,0,0,0,0,0,0,0,0,0,0,0,0,0,0,0
,0,0,0,0,1,0,0,0,0,0,0,0,0,S,S,S,S,S,S,S,S,R,I,S,S,I,R,S,R,I,1,0
,0,1,0,1,0,0,0,1,0,R,Co
42,M,15233,156,1,BB,0,0,0,0,0,0,0,0,0,0,0,0,0,0,0,0,0,0,0,0,0,0,
0,0,0,0,0,0,0,0,0,0,0,0,0,0,S,S,S,S,S,S,S,S,S,S,S,S,S,S,S,S,S,1,
0,0,0,0,0,1,0,0,0,1,S,Co
44,F,1951,197,1,BB,0,0,0,0,0,0,1,1,0,1,1,0,1,0,0,0,0,1,0,0,1,0,0
,0,0,0,1,1,1,1,1,0,1,0,0,0,R,S,R,R,R,I,R,S,R,S,I,I,S,R,S,R,S,1,0
,0,0,0,0,0,1,0,0,1,R,Co
48,M,206459,80,1,BB,0,0,0,0,0,0,0,0,0,0,0,0,0,0,0,0,0,0,0,0,1,0,
0,0,0,0,0,1,0,0,0,0,0,1,0,0,S,S,S,S,S,S,S,S,R,S,S,S,S,R,S,I,S,1,
0,0,1,0,0,0,0,1,0,0,R,Co
63,M,10831,438,1,BB,1,0,0,0,1,1,0,1,1,0,1,1,0,0,1,0,0,0,0,0,1,0,
0,0,0,0,0,1,0,0,0,0,0,1,1,0,R,I,R,R,R,R,R,S,R,S,I,I,S,R,S,R,S,1,
0,0,0,0,0,0,0,0,0,1,R,Co
43,M,11791,102,1,BB,0,0,0,0,0,0,0,0,0,0,0,0,0,0,0,0,0,0,0,0,0,0,
0,0,0,0,0,1,0,0,0,0,0,0,0,0,S,S,S,S,S,S,S,S,R,S,S,S,S,R,S,R,R,1,
0,0,1,0,0,0,0,0,0,0,R,Co
39,M,3380,420,1,BB,0,0,0,0,0,0,0,0,0,0,0,0,0,0,0,0,0,0,0,0,0,0,0
,0,0,0,0,1,0,0,0,0,0,0,0,0,S,S,S,S,S,S,S,S,R,S,S,S,S,R,S,S,S,1,0
,1,1,0,0,0,0,0,0,0,S,Co
39,F,720,837,1,BB,0,0,0,0,0,0,0,0,0,0,0,0,0,0,0,0,0,0,0,0,0,1,0,
0,0,0,0,1,0,0,0,0,1,0,0,1,S,S,S,S,S,S,S,S,R,R,S,S,R,R,S,R,R,1,0,
0,0,0,0,0,0,0,0,1,R,Co
46,M,205287,366,1,BB,0,0,0,1,0,0,1,0,0,1,0,0,0,0,0,0,1,0,0,1,0,0
,1,1,1,1,0,0,0,0,0,0,1,0,0,0,R,I,S,R,R,S,I,I,I,R,R,R,R,I,I,R,I,1
,0,0,0,0,0,1,0,0,0,1,R,Co
39,F,2966,148,1,BB,0,0,0,0,0,0,0,0,0,0,0,0,0,0,0,0,0,0,0,0,0,0,0
,0,0,0,0,1,0,0,0,0,0,0,0,0,S,S,S,S,S,S,S,S,R,S,S,S,S,R,S,R,S,1,0
,0,0,0,0,0,0,0,0,1,R,Co
36,F,50218,89,1,BB,0,0,0,0,0,0,0,0,0,0,0,0,0,0,0,0,0,0,0,0,0,0,0
,0,0,0,0,0,0,0,0,0,0,0,0,0,S,S,S,S,S,S,S,S,S,S,S,S,S,S,S,S,S,1,0
,0,1,0,0,0,0,0,0,0,S,Co
34,F,1016,76,1,F1,0,0,0,0,0,0,0,0,0,0,0,0,0,0,0,0,0,0,0,0,0,0,0,
0,0,0,0,0,0,0,0,0,0,0,0,0,S,S,S,S,S,S,S,S,S,S,S,S,S,S,S,S,S,1,0,
0,1,0,0,0,1,0,0,0,S,Co

35,M,2861,154,1,BB,0,0,0,0,0,0,0,0,0,0,0,1,0,0,0,0,0,0,0,0,0,0,0
,0,0,0,0,1,0,0,0,0,0,0,0,0,S,S,S,S,S,S,S,S,R,S,S,S,S,R,S,R,I,1,0
,0,0,1,0,0,0,0,0,0,R,Co
50,M,49,956,1,BB,0,0,0,0,0,0,0,0,0,0,0,1,0,0,0,1,0,0,0,0,0,0,0,0
,0,0,0,0,0,0,0,0,0,0,0,0,S,S,S,S,S,S,S,S,S,LR,IR,IR,LR,S,LR,S,S,
1,0,0,0,0,0,0,1,0,0,1,S,Co
50,M,49,427,1,CC,0,0,0,0,0,0,0,0,0,0,0,0,0,0,0,0,0,0,0,0,0,0,0,0
,0,0,0,0,0,0,0,0,0,0,0,0,S,S,S,S,S,S,S,S,S,S,S,S,S,S,S,S,S,1,0,0
,0,0,0,0,0,0,0,1,S,Co
40,M,49,666,0,BB,0,0,0,0,0,0,0,0,0,0,0,0,0,0,0,0,0,0,0,0,0,0,0,0
,0,0,0,0,0,0,0,0,0,0,0,0,S,S,S,S,S,S,S,S,S,S,S,S,S,S,S,S,S,1,0,0
,0,0,0,0,0,0,0,1,S,Co
47,M,49,477,1,BB,0,0,0,0,0,0,0,0,0,0,0,0,0,0,0,0,0,0,0,0,0,0,0,0
,0,0,0,0,0,0,0,0,0,0,0,0,S,S,S,S,S,S,S,S,S,S,S,S,S,S,S,S,S,1,0,0
,1,0,0,0,1,0,0,0,S,Co
41,F,49,1058,0,BB,0,0,0,0,0,0,0,0,0,0,1,0,0,0,0,0,0,0,0,0,0,0,0,
0,0,0,0,0,0,0,0,0,0,0,0,0,S,S,S,S,S,S,S,S,S,S,S,S,S,S,S,S,S,1,0,
0,0,0,0,0,0,1,0,1,S,Co
38,M,72273,290,0,KF,0,0,0,0,0,0,0,0,0,0,0,0,0,0,0,0,0,0,0,0,0,0,
0,0,0,0,0,0,0,0,0,0,0,0,0,0,S,S,S,S,S,S,S,S,S,S,S,S,S,S,S,S,S,0,
0,0,0,0,0,0,0,0,0,0,S,Co
54,M,116,300,1,BB,0,0,0,0,0,0,0,0,0,0,1,0,0,0,0,0,0,0,0,0,0,0,0,
0,0,0,0,0,0,0,0,0,0,0,0,0,S,S,S,S,S,S,S,S,S,S,S,S,S,S,LR,S,1,0
,0,0,0,0,0,1,0,0,1,LR,Co
36,M,49,460,0,CB,0,0,0,0,0,0,0,0,0,0,0,1,0,0,0,0,0,0,0,0,0,0,0,0
,0,0,0,0,0,0,0,0,0,0,0,0,S,S,S,S,S,S,S,S,S,S,S,S,S,S,S,S,S,1,0,0
,0,0,0,0,0,0,0,1,S,Co
40,M,16069,149,1,BB,0,0,0,0,0,0,0,0,0,0,0,0,0,0,0,0,0,0,0,0,0,0,
0,0,0,0,0,0,0,0,0,0,0,0,0,0,S,S,S,S,S,S,S,S,S,S,S,S,S,S,S,S,S,0,
0,0,0,0,0,0,0,0,0,0,S,Co
38,M,7950,382,0,BB,0,0,0,0,0,0,0,0,0,0,0,0,0,0,0,0,0,0,0,0,0,0,0
,0,0,0,0,0,0,0,0,0,0,0,0,0,0,S,S,S,S,S,S,S,S,S,S,S,S,S,S,S,S,S,1,0
,0,0,0,0,0,0,0,0,1,S,Co
36,M,266198,148,1,BB,0,0,0,0,0,0,0,0,0,0,0,0,0,0,0,0,0,0,0,0,0,0
,0,0,0,0,0,0,0,0,0,0,0,0,0,0,S,S,S,S,S,S,S,S,S,S,S,S,S,S,S,S,S,0
,0,0,0,0,0,0,0,0,0,0,S,Co
44,F,49,854,0,BB,0,0,0,0,0,0,0,0,0,0,1,0,0,0,0,0,0,0,0,0,0,0,0,0
,0,0,0,0,0,0,0,0,0,0,0,0,S,S,S,S,S,S,S,S,S,S,S,S,S,S,LR,S,1,0,
0,0,0,0,0,0,0,0,1,LR,Co
54,M,12726,361,1,BB,0,0,0,0,0,0,0,0,0,0,0,0,0,0,0,0,0,0,0,0,0,0,
0,0,0,0,0,0,0,0,0,0,0,0,0,0,0,S,S,S,S,S,S,S,S,S,S,S,S,S,S,S,S,S,0,
0,0,0,0,0,0,0,0,0,0,S,Co
46,M,49,490,1,CC,0,0,0,0,0,0,0,0,0,0,0,0,0,0,0,0,0,0,0,0,0,0,0,0
,0,0,0,0,0,0,0,0,0,0,0,0,S,S,S,S,S,S,S,S,S,S,S,S,S,S,S,S,LR,1,0,
0,0,0,0,0,0,0,0,1,S,Co
49,F,49,864,0,BB,0,0,0,0,0,0,0,0,1,0,0,0,0,0,0,0,0,0,0,0,0,0,0,0
,0,0,0,0,0,0,0,0,0,0,0,0,LR,S,LR,I,I,I,LR,S,S,S,S,S,S,S,S,S,S,1,
0,0,1,0,0,0,1,0,0,0,S,Co

**Supplementary File 5 –** Unlabeled instances and attributes of 15 patients recognized as mon-infected ones. This file was classified using the model 1. Weka file format (arff).

```
@relation new_ids_unknow_class-
weka.filters.unsupervised.attribute.NumericToBinary-R5-
weka.filters.unsupervised.attribute.NumericToBinary-R7-42-
weka.filters.unsupervised.attribute.NumericToBinary-R60-70

@attribute AGE numeric
@attribute GENDER {F,M}
@attribute CV numeric
@attribute CD4 numeric
@attribute AIDS_case_binarized {0,1}
@attribute PR_RT {BB,F1,BF,CC,KF,CB}
@attribute L24I_majorIP_binarized {0,1}
@attribute V32I_majorIP_binarized {0,1}
@attribute L33F_majorIP_binarized {0,1}
@attribute M46I_majorIP_binarized {0,1}
@attribute M46L_majorIP_binarized {0,1}
@attribute I54L_majorIP_binarized {0,1}
@attribute I54V_majorIP_binarized {0,1}
@attribute V82A_majorIP_binarized {0,1}
@attribute I84V_majorIP_binarized {0,1}
@attribute L90M_majorIP_binarized {0,1}
@attribute L10I_minorIP_binarized {0,1}
@attribute L10V_minorIP_binarized {0,1}
@attribute A71T_minorIP_binarized {0,1}
@attribute A71V_minorIP_binarized {0,1}
@attribute T74S_minorIP_binarized {0,1}
@attribute M41L_NRTI_binarized {0,1}
@attribute A62V_NRTI_binarized {0,1}
@attribute D67G_NRTI_binarized {0,1}
@attribute D67N_NRTI_binarized {0,1}
@attribute T69N_NRTI_binarized {0,1}
@attribute K70R_NRTI_binarized {0,1}
@attribute L74V_NRTI_binarized {0,1}
@attribute V75I_NRTI_binarized {0,1}
@attribute F116Y_NRTI_binarized {0,1}
@attribute V118I_NRTI_binarized {0,1}
@attribute Q151M_NRTI_binarized {0,1}
@attribute M184I_NRTI_binarized {0,1}
@attribute M184V_NRTI_binarized {0,1}
@attribute T215F_NRTI_binarized {0,1}
@attribute T215I_NRTI_binarized {0,1}
@attribute T215S_NRTI_binarized {0,1}
@attribute T215Y_NRTI_binarized {0,1}
@attribute K219E_NRTI_binarized {0,1}
@attribute K219Q_NRTI_binarized {0,1}
@attribute P225H_NNRTI_binarized {0,1}
@attribute M230L_NNRTI_binarized {0,1}
@attribute S_ATV_r {S,HR,IR,LR,R}
@attribute S_DRV_r {S,LR,IR,I}
@attribute S_FPV_r {S,IR,HR,LR,R}
```

@attribute S_IDV_r {S,HR,IR,LR,R,I}
@attribute S_LPV_r {S,HR,IR,LR,R,I}
@attribute S_NFV {S,HR,R,I}
@attribute S_SQV_r {S,HR,IR,LR,R,I}
@attribute S_TPV_r {S,LR,IR,HR,R,I}
@attribute S_3TC {S,HR,LR,R,I}
@attribute S_ABC {S,HR,LR,IR,R,I}
@attribute S_AZT {S,HR,IR,LR,I,R}
@attribute S_D4T {S,HR,IR,LR,I,R}
@attribute S_DDI {S,HR,IR,LR,R,I}
@attribute S_FTC {S,HR,LR,R,I}
@attribute S_TDF {S,LR,IR,HR,R,I}
@attribute S_EFV {HR,LR,S,IR,R,I}
@attribute S_ETR {LR,HR,S,IR,I,R}
@attribute X_3TC_use_binarized {0,1}
@attribute ATV_use_binarized {0,1}
@attribute ATV_r_use_binarized {0,1}
@attribute AZT_use_binarized {0,1}
@attribute D4T_use_binarized {0,1}
@attribute DDI_use_binarized {0,1}
@attribute DRV_r_use_binarized {0,1}
@attribute LPV_r_use_binarized {0,1}
@attribute NVP_use_binarized {0,1}
@attribute RTV_use_binarized {0,1}
@attribute TDF_use_binarized {0,1}
@attribute S_NVP {HR,LR,S,R}
@attribute Class {Mono,Co}

@data
41,M,3802,205,0,BB,0,0,0,0,0,0,0,0,0,0,0,0,0,0,0,0,0,0,0,0
,0,0,0,0,1,0,0,0,0,0,0,0,0,S,S,S,S,S,S,S,S,HR,LR,S,S,S,HR,S,HR,L
R,1,0,0,1,0,0,0,0,1,0,0,HR,?
38,F,2864,432,0,BB,0,0,0,0,0,0,1,1,0,1,1,0,0,1,0,1,0,0,0,0,0,0
,0,0,0,0,1,0,0,0,1,0,0,0,0,HR,S,IR,HR,IR,HR,HR,LR,HR,IR,IR,IR,IR
,HR,LR,HR,LR,1,0,0,1,0,0,0,0,1,0,0,HR,?
37,M,5300,819,0,BB,0,0,0,0,0,0,0,0,0,0,1,1,0,0,0,0,0,0,0,0,0,0
,0,0,0,0,1,0,0,0,0,0,0,1,0,S,S,S,S,S,S,S,S,HR,LR,S,S,S,HR,S,HR,L
R,1,0,0,1,0,0,0,0,0,0,0,HR,?
57,M,197720,46,1,BB,0,0,0,0,0,0,0,0,0,0,0,0,0,0,0,0,0,0,0,0,
0,0,0,0,0,0,0,0,0,0,0,0,0,0,S,S,S,S,S,S,S,S,S,S,S,S,S,S,S,S,S,1,
0,0,1,0,0,0,1,0,0,1,S,?
42,M,3048,439,1,BB,0,0,0,0,0,0,0,0,0,0,0,0,0,0,0,1,0,0,0,1,0,0,0
,0,0,0,0,1,0,0,0,1,0,0,0,0,S,S,S,S,S,S,S,S,HR,HR,HR,HR,HR,HR,IR,
HR,LR,1,0,0,0,0,0,0,0,0,0,1,HR,?
34,M,3087,591,0,BB,0,0,0,0,0,0,0,0,0,0,0,0,1,0,0,1,0,0,0,0,0,0,0
,0,0,0,0,0,1,0,0,0,0,1,0,0,S,S,S,S,S,S,S,S,S,IR,HR,HR,IR,S,LR,S,
S,1,0,0,0,0,0,0,1,0,0,1,S,?
50,F,71632,340,1,BB,1,0,0,1,0,0,0,1,0,0,1,0,0,1,0,0,1,0,0,0,0,0,
0,0,0,0,0,1,0,0,0,0,0,1,0,0,HR,S,IR,IR,IR,HR,IR,S,HR,HR,S,LR,IR,
HR,IR,S,S,1,0,1,0,0,0,0,0,0,0,1,S,?
49,F,8016,1056,0,BF,0,0,0,0,0,0,0,0,0,0,0,0,0,0,0,1,0,0,0,0,0,0,
0,0,1,0,0,1,0,0,0,1,0,0,0,0,S,S,S,S,S,S,S,S,HR,HR,HR,HR,HR,HR,IR
,IR,LR,1,0,0,1,0,0,0,0,0,0,0,HR,?
58,F,39481,43,0,BB,0,0,0,0,0,0,0,0,0,1,0,0,0,0,0,0,1,0,0,0,0,0,1
,1,0,1,0,0,0,0,0,0,0,0,0,0,LR,S,LR,IR,LR,HR,IR,S,LR,IR,HR,HR,HR,
LR,LR,HR,LR,0,0,0,0,0,0,0,1,0,0,0,HR,?

```
31,F,190164,73,1,BB,0,1,1,0,1,0,0,0,0,0,0,1,1,0,0,0,1,0,0,0,0,0,
1,1,0,1,0,1,0,0,0,0,1,0,0,0,HR,LR,HR,IR,IR,HR,LR,LR,HR,HR,HR,HR,
HR,HR,HR,HR,HR,1,1,0,0,0,0,0,0,0,1,1,HR,?
65,M,10744,335,0,F1,0,0,0,0,1,0,1,0,0,0,1,0,0,0,0,0,0,0,0,0,1,0,
0,0,0,0,0,1,0,1,0,0,1,0,0,0,HR,S,IR,HR,IR,HR,HR,IR,HR,IR,HR,IR,L
R,HR,LR,S,S,1,0,0,1,0,0,0,1,0,0,1,S,?
40,F,167281,271,0,BB,0,0,0,0,0,0,0,0,0,0,0,0,0,0,0,0,0,0,0,0,0,0
,0,0,0,0,0,0,0,0,0,0,0,0,1,0,S,S,S,S,S,S,S,S,S,S,S,S,S,S,S,HR,LR
,1,0,0,1,0,0,0,0,0,0,0,HR,?
32,M,11198,66,0,BB,0,0,0,0,0,0,0,0,0,0,0,0,0,0,0,0,0,0,1,1,0,0
,0,1,0,0,1,1,0,0,0,1,0,0,0,S,S,S,S,S,S,S,S,HR,IR,HR,HR,IR,HR,LR,
HR,IR,1,0,0,1,0,0,0,0,0,0,0,HR,?
32,M,11198,66,0,BB,0,0,0,0,0,0,0,0,0,0,0,0,0,0,0,0,0,0,1,1,0,0
,0,1,0,0,1,1,0,0,0,1,0,0,0,S,S,S,S,S,S,S,S,HR,IR,HR,HR,IR,HR,LR,
HR,IR,1,0,0,1,0,0,0,0,0,0,0,HR,?
45,M,1704,478,1,BB,0,0,0,0,0,0,0,0,0,0,0,0,0,0,0,0,0,0,0,0,0,0
,0,0,0,0,0,0,0,0,0,0,0,0,0,0,S,S,S,S,S,S,S,S,S,S,S,S,S,S,S,S,1,0
,1,0,0,0,0,0,0,0,1,S,?
```

**Supplementary File 6 –** Unlabeled instances and attributes of 15 patients recognized as mon-infected ones. This file was classified using the model 2. Weka file format (arff).

```
@relation new_ids_unknown_class-
weka.filters.unsupervised.attribute.NumericToBinary-R5-
weka.filters.unsupervised.attribute.NumericToBinary-R7-42-
weka.filters.unsupervised.attribute.NumericToBinary-R60-70

@attribute AGE numeric
@attribute GENDER {M,F}
@attribute CV numeric
@attribute CD4 numeric
@attribute AIDS_case_binarized {0,1}
@attribute PR_RT {BB,F1,BF,CC,KF,CB}
@attribute L24I_majorIP_binarized {0,1}
@attribute V32I_majorIP_binarized {0,1}
@attribute L33F_majorIP_binarized {0,1}
@attribute M46I_majorIP_binarized {0,1}
@attribute M46L_majorIP_binarized {0,1}
@attribute I54L_majorIP_binarized {0,1}
@attribute I54V_majorIP_binarized {0,1}
@attribute V82A_majorIP_binarized {0,1}
@attribute I84V_majorIP_binarized {0,1}
@attribute L90M_majorIP_binarized {0,1}
@attribute L10I_minorIP_binarized {0,1}
@attribute L10V_minorIP_binarized {0,1}
@attribute A71T_minorIP_binarized {0,1}
@attribute A71V_minorIP_binarized {0,1}
@attribute T74S_minorIP_binarized {0,1}
@attribute M41L_NRTI_binarized {0,1}
@attribute A62V_NRTI_binarized {0,1}
@attribute D67G_NRTI_binarized {0,1}
@attribute D67N_NRTI_binarized {0,1}
@attribute T69N_NRTI_binarized {0,1}
```

```
@attribute K70R_NRTI_binarized {0,1}
@attribute L74V_NRTI_binarized {0,1}
@attribute V75I_NRTI_binarized {0,1}
@attribute F116Y_NRTI_binarized {0,1}
@attribute V118I_NRTI_binarized {0,1}
@attribute Q151M_NRTI_binarized {0,1}
@attribute M184I_NRTI_binarized {0,1}
@attribute M184V_NRTI_binarized {0,1}
@attribute T215F_NRTI_binarized {0,1}
@attribute T215I_NRTI_binarized {0,1}
@attribute T215S_NRTI_binarized {0,1}
@attribute T215Y_NRTI_binarized {0,1}
@attribute K219E_NRTI_binarized {0,1}
@attribute K219Q_NRTI_binarized {0,1}
@attribute P225H_NNRTI_binarized {0,1}
@attribute M230L_NNRTI_binarized {0,1}
@attribute S_ATV_r {HR,S,IR,R,LR}
@attribute S_DRV_r {S,IR,LR,I}
@attribute S_FPV_r {IR,S,LR,HR,R}
@attribute S_IDV_r {HR,S,IR,LR,R,I}
@attribute S_LPV_r {IR,S,LR,HR,R,I}
@attribute S_NFV {HR,S,IR,R,I}
@attribute S_SQV_r {IR,S,HR,LR,R,I}
@attribute S_TPV_r {IR,S,LR,R,I}
@attribute S_3TC {HR,S,R,I}
@attribute S_ABC {HR,LR,IR,S,R,I}
@attribute S_AZT {HR,IR,S,LR,I,R}
@attribute S_D4T {HR,LR,S,IR,I,R}
@attribute S_DDI {HR,IR,LR,S,R,I}
@attribute S_FTC {HR,S,R,I}
@attribute S_TDF {IR,LR,S,R,I}
@attribute S_EFV {S,HR,IR,LR,R,I}
@attribute S_ETR {S,LR,IR,HR,I,R}
@attribute X_3TC_use_binarized {0,1}
@attribute ATV_use_binarized {0,1}
@attribute ATV_r_use_binarized {0,1}
@attribute AZT_use_binarized {0,1}
@attribute D4T_use_binarized {0,1}
@attribute 'DDI_use_binarized' {0,1}
@attribute DRV_r_use_binarized {0,1}
@attribute LPV_r_use_binarized {0,1}
@attribute NVP_use_binarized {0,1}
@attribute RTV_use_binarized {0,1}
@attribute TDF_use_binarized {0,1}
@attribute S_NVP {S,HR,LR,IR,R}
@attribute Class {Mono,Co}

@data
41,M,3802,205,0,BB,0,0,0,0,0,0,0,0,0,0,0,0,0,0,0,0,0,0,0,0,0
,0,0,0,0,1,0,0,0,0,0,0,0,0,S,S,S,S,S,S,S,S,HR,LR,S,S,S,HR,S,HR,L
R,1,0,0,1,0,0,0,0,1,0,0,HR,?
38,F,2864,432,0,BB,0,0,0,0,0,0,1,1,0,1,1,0,0,1,0,1,0,0,0,0,0,0,0
,0,0,0,0,1,0,0,0,1,0,0,0,0,HR,S,IR,HR,IR,HR,HR,LR,HR,IR,IR,IR,IR
,HR,LR,HR,LR,1,0,0,1,0,0,0,0,1,0,0,HR,?
```

```
37,M,5300,819,0,BB,0,0,0,0,0,0,0,0,0,0,1,1,0,0,0,0,0,0,0,0,0,0,0
,0,0,0,0,1,0,0,0,0,0,1,0,S,S,S,S,S,S,S,S,HR,LR,S,S,S,HR,S,HR,L
R,1,0,0,1,0,0,0,0,0,0,0,HR,?
57,M,197720,46,1,BB,0,0,0,0,0,0,0,0,0,0,0,0,0,0,0,0,0,0,0,0,0,0,
0,0,0,0,0,0,0,0,0,0,0,0,0,0,S,S,S,S,S,S,S,S,S,S,S,S,S,S,S,S,S,1,
0,0,1,0,0,0,1,0,0,1,S,?
42,M,3048,439,1,BB,0,0,0,0,0,0,0,0,0,0,0,0,0,0,0,1,0,0,0,1,0,0,0
,0,0,0,0,1,0,0,0,1,0,0,0,0,S,S,S,S,S,S,S,S,HR,HR,HR,HR,HR,HR,IR,
HR,LR,1,0,0,0,0,0,0,0,0,0,1,HR,?
34,M,3087,591,0,BB,0,0,0,0,0,0,0,0,0,0,0,0,1,0,0,1,0,0,1,0,0,0,0
,0,0,0,0,0,1,0,0,0,0,1,0,0,S,S,S,S,S,S,S,S,S,IR,HR,HR,IR,S,LR,S,
S,1,0,0,0,0,0,0,1,0,0,1,S,?
50,F,71632,340,1,BB,1,0,0,1,0,0,0,1,0,0,1,0,0,1,0,0,1,0,0,0,0,0,
0,0,0,0,0,1,0,0,0,0,0,1,0,0,HR,S,IR,IR,IR,HR,IR,S,HR,HR,S,LR,IR,
HR,IR,S,S,1,0,1,0,0,0,0,0,0,0,1,S,?
49,F,8016,1056,0,BF,0,0,0,0,0,0,0,0,0,0,0,0,0,0,0,1,0,0,1,0,0,0,
0,0,1,0,0,1,0,0,0,1,0,0,0,0,S,S,S,S,S,S,S,S,HR,HR,HR,HR,HR,HR,IR
,IR,LR,1,0,0,1,0,0,0,0,0,0,0,HR,?
65,M,10744,335,0,F1,0,0,0,0,1,0,1,0,0,0,1,0,0,0,0,0,0,0,1,0,1,0,
0,0,0,0,0,1,0,1,0,0,1,0,0,0,HR,S,IR,HR,IR,HR,HR,IR,HR,IR,HR,IR,L
R,HR,LR,S,S,1,0,0,1,0,0,0,1,0,0,1,S,?
40,F,167281,271,0,BB,0,0,0,0,0,0,0,0,0,0,0,0,0,0,0,0,0,0,0,0,0,0
,0,0,0,0,0,0,0,0,0,0,0,0,1,0,S,S,S,S,S,S,S,S,S,S,S,S,S,S,S,HR,LR
,1,0,0,1,0,0,0,0,0,0,0,HR,?
32,M,11198,66,0,BB,0,0,0,0,0,0,0,0,0,0,0,0,0,0,0,0,0,0,1,1,1,0,0
,0,1,0,0,1,1,0,0,0,1,0,0,0,S,S,S,S,S,S,S,S,HR,IR,HR,HR,IR,HR,LR,
HR,IR,1,0,0,1,0,0,0,0,0,0,0,HR,?
32,M,11198,66,0,BB,0,0,0,0,0,0,0,0,0,0,0,0,0,0,0,0,0,0,1,1,1,0,0
,0,1,0,0,1,1,0,0,0,1,0,0,0,S,S,S,S,S,S,S,S,HR,IR,HR,HR,IR,HR,LR,
HR,IR,1,0,0,1,0,0,0,0,0,0,0,HR,?
45,M,1704,478,1,BB,0,0,0,0,0,0,0,0,0,0,0,0,0,0,0,0,0,0,0,0,0,0,0
,0,0,0,0,0,0,0,0,0,0,0,0,0,S,S,S,S,S,S,S,S,S,S,S,S,S,S,S,S,S,1,0
,1,0,0,0,0,0,0,0,1,S,?
```

**Supplementary File 7 -** Instances and attributes of all mono-infected patients used for unsupervised learning. The clusters are also assigned. Weka file format (arff).

```
@relation 'Rej.no_child-
weka.filters.unsupervised.attribute.NumericToBinary-R6,8-43,61-
71_clustered-weka.filters.unsupervised.attribute.Remove-R1-2'

@attribute AGE numeric
@attribute GENDER {F,M}
@attribute CV numeric
@attribute CD4 numeric
@attribute AIDS_case_binarized {0,1}
@attribute PR_RT {BB,F1,BF,BD,CC,BC}
@attribute L24I_majorIP_binarized {0,1}
@attribute V32I_majorIP_binarized {0,1}
@attribute L33F_majorIP_binarized {0,1}
@attribute M46I_majorIP_binarized {0,1}
@attribute M46L_majorIP_binarized {0,1}
```

```
@attribute I54L_majorIP_binarized {0,1}
@attribute I54V_majorIP_binarized {0,1}
@attribute V82A_majorIP_binarized {0,1}
@attribute I84V_majorIP_binarized {0,1}
@attribute L90M_majorIP_binarized {0,1}
@attribute L10I_minorIP_binarized {0,1}
@attribute L10V_minorIP_binarized {0,1}
@attribute A71T_minorIP_binarized {0,1}
@attribute A71V_minorIP_binarized {0,1}
@attribute T74S_minorIP_binarized {0,1}
@attribute M41L_NRTI_binarized {0,1}
@attribute A62V_NRTI_binarized {0,1}
@attribute D67G_NRTI_binarized {0,1}
@attribute D67N_NRTI_binarized {0,1}
@attribute T69N_NRTI_binarized {0,1}
@attribute K70R_NRTI_binarized {0,1}
@attribute L74V_NRTI_binarized {0,1}
@attribute V75I_NRTI_binarized {0,1}
@attribute F116Y_NRTI_binarized {0,1}
@attribute V118I_NRTI_binarized {0,1}
@attribute Q151M_NRTI_binarized {0,1}
@attribute M184I_NRTI_binarized {0,1}
@attribute M184V_NRTI_binarized {0,1}
@attribute T215F_NRTI_binarized {0,1}
@attribute T215I_NRTI_binarized {0,1}
@attribute T215S_NRTI_binarized {0,1}
@attribute T215Y_NRTI_binarized {0,1}
@attribute K219E_NRTI_binarized {0,1}
@attribute K219Q_NRTI_binarized {0,1}
@attribute P225H_NNRTI_binarized {0,1}
@attribute M230L_NNRTI_binarized {0,1}
@attribute S_ATV_r {S,HR,LR,IR}
@attribute S_DRV_r {S,LR,IR}
@attribute S_FPV_r {S,IR,HR,LR}
@attribute S_IDV_r {S,HR,IR,LR}
@attribute S_LPV_r {S,IR,LR,HR}
@attribute S_NFV {S,HR,LR,IR}
@attribute S_SQV_r {S,HR,LR,IR}
@attribute S_TPV_r {S,LR,IR,HR}
@attribute S_3TC {HR,S,LR,IR}
@attribute S_ABC {LR,HR,IR,S}
@attribute S_AZT {S,HR,IR,LR}
@attribute S_D4T {LR,HR,IR,S}
@attribute S_DDI {LR,HR,IR,S}
@attribute S_FTC {HR,S,LR,IR}
@attribute S_TDF {S,LR,IR,HR}
@attribute S_EFV {HR,S,IR,LR}
@attribute S_ETR {LR,IR,S,HR}
@attribute _3TC_use_binarized {0,1}
@attribute ATV_use_binarized {0,1}
@attribute ATV_r_use_binarized {0,1}
@attribute AZT_use_binarized {0,1}
@attribute D4T_use_binarized {0,1}
@attribute DDI_use_binarized {0,1}
@attribute DRV_r_use_binarized {0,1}
@attribute LPV_r_use_binarized {0,1}
```

```
@attribute NVP_use_binarized {0,1}
@attribute RTV_use_binarized {0,1}
@attribute TDF_use_binarized {0,1}
@attribute S_NVP {HR,S,LR,IR}
@attribute Cluster
{cluster0,cluster1,cluster2,cluster3,cluster4,cluster5}

@data
30,F,13455,273,1,BB,0,0,0,0,0,0,0,0,0,0,0,1,0,0,0,0,1,0,0,0,0,0,
0,0,0,0,0,1,0,0,0,0,0,0,0,0,S,S,S,S,S,S,S,S,HR,LR,S,LR,LR,HR,S,H
R,LR,1,0,0,1,0,0,0,0,0,0,0,HR,cluster1
42,F,182845,10,1,BB,0,0,1,1,0,0,0,1,0,1,0,0,0,0,0,0,0,0,1,1,1,0,
0,0,0,0,0,1,1,0,0,0,0,1,0,0,HR,LR,IR,HR,IR,HR,HR,LR,HR,HR,HR,HR,
HR,HR,LR,HR,IR,1,0,0,0,0,0,0,0,0,0,1,HR,cluster2
41,F,2318,683,1,BB,0,0,0,0,0,0,0,0,0,0,0,0,0,0,0,1,0,0,0,0,0,0,1
,0,0,0,0,1,0,0,0,1,0,0,0,0,S,S,S,S,S,S,S,S,HR,IR,IR,IR,IR,HR,LR,
HR,LR,1,0,0,1,0,0,0,0,0,0,0,HR,cluster0
59,F,145510,64,1,BB,0,0,1,1,0,0,0,1,0,0,0,0,0,0,0,1,0,0,0,0,0,0,
0,0,0,0,0,1,0,0,0,1,0,0,0,0,HR,S,IR,IR,IR,HR,LR,LR,HR,IR,IR,IR,I
R,HR,LR,S,S,1,0,1,1,0,0,0,0,0,0,0,S,cluster3
53,F,8448,343,0,BB,0,0,1,0,0,1,0,0,0,1,1,0,1,0,1,0,1,0,0,0,0,0,0
,0,0,0,0,1,0,0,0,0,0,0,0,0,HR,LR,HR,HR,IR,HR,HR,S,HR,LR,S,S,S,HR
,S,HR,LR,1,0,1,1,0,0,0,0,0,0,0,HR,cluster5
35,M,6405,355,0,BB,0,0,0,0,0,0,0,0,0,0,0,0,0,0,0,0,1,0,0,0,0,0,0
,0,0,0,0,1,0,0,0,0,0,0,0,0,S,S,S,S,S,S,S,S,HR,LR,S,S,S,HR,S,HR,L
R,1,0,0,1,0,0,0,0,1,0,0,HR,cluster1
37,F,31562,14,1,BB,0,0,0,0,0,0,0,0,0,1,0,0,0,0,0,0,0,0,1,0,0,0,0
,0,0,0,0,0,0,1,1,0,0,0,0,LR,S,LR,IR,LR,HR,IR,S,S,LR,HR,IR,LR,S
,LR,HR,HR,1,0,0,0,0,0,0,0,0,0,1,HR,cluster3
33,M,42975,195,0,BB,0,0,0,0,0,0,0,0,0,1,0,1,0,1,1,1,0,0,1,0,0,0,
0,0,1,0,0,1,0,0,0,1,0,0,0,0,LR,S,LR,IR,LR,HR,IR,S,HR,HR,HR,HR,HR
,HR,IR,HR,IR,1,0,0,0,0,0,0,0,0,0,1,HR,cluster3
41,M,17972,173,0,BB,0,0,0,0,0,0,0,0,0,0,0,0,0,0,0,0,0,0,0,0,0,0,
0,0,0,0,0,1,0,0,0,0,0,0,1,0,S,S,S,S,S,S,S,S,HR,LR,S,S,S,HR,S,HR,
LR,1,0,0,1,0,0,0,0,0,0,0,HR,cluster1
63,M,18253,300,1,BB,0,1,0,0,0,0,0,0,0,0,0,1,1,0,0,0,0,0,1,1,1,0,
0,0,0,0,0,0,0,0,0,0,0,1,0,0,LR,LR,IR,LR,LR,LR,S,S,S,IR,HR,HR,HR,
S,IR,HR,IR,1,0,0,1,0,0,0,0,0,0,0,HR,cluster3
47,M,17303,207,0,F1,0,0,0,0,0,0,1,1,0,0,1,0,0,1,0,1,0,0,0,0,0,0,
0,0,0,0,0,1,0,0,0,1,0,0,0,0,IR,S,LR,IR,IR,IR,IR,LR,HR,IR,IR,IR,I
R,HR,LR,HR,IR,1,0,0,1,0,0,0,0,0,0,0,HR,cluster5
46,M,18283,377,0,BB,0,0,0,0,0,0,0,0,0,0,0,1,0,0,0,0,0,0,0,0,0,0,
0,0,0,0,0,1,0,0,0,0,0,0,0,0,S,S,S,S,S,S,S,S,HR,LR,S,S,S,HR,S,HR,
LR,1,0,0,1,0,0,0,0,0,0,0,HR,cluster1
35,F,78443,216,1,BB,0,0,0,0,0,0,0,0,0,0,0,0,0,0,0,0,0,0,0,0,0,0,
0,0,0,0,0,1,0,0,0,0,0,0,0,0,S,S,S,S,S,S,S,S,HR,LR,S,S,S,HR,S,HR,
LR,1,0,0,1,0,0,0,0,1,0,0,HR,cluster1
40,M,9403,254,1,BB,0,1,0,1,0,0,1,1,0,0,1,0,0,1,0,1,0,0,1,0,0,0,0
,0,0,0,0,1,0,0,0,1,1,0,1,0,IR,LR,HR,HR,HR,HR,IR,LR,HR,HR,HR,HR,I
R,HR,IR,IR,LR,1,1,0,1,0,0,0,0,0,0,0,LR,cluster2
58,F,39481,43,0,BB,0,0,0,0,0,0,0,0,0,1,0,0,0,0,0,0,1,0,0,0,0,0,1
,1,0,1,0,0,0,0,0,0,0,0,0,0,LR,S,LR,IR,LR,HR,IR,S,LR,IR,HR,HR,HR,
LR,LR,HR,LR,0,0,0,0,0,0,0,1,0,0,0,HR,cluster3
33,F,9832,252,?,BB,0,0,0,0,0,0,0,0,0,0,0,0,0,0,0,0,1,0,0,0,0,0,0
,0,0,0,0,0,1,0,0,0,0,0,0,0,S,S,S,S,S,S,S,S,S,IR,IR,IR,IR,S,LR,HR
,HR,0,0,0,1,0,1,0,0,1,0,0,HR,cluster0
```

58,F,3750,349,1,BB,1,0,1,1,0,0,1,1,0,0,1,0,0,0,0,1,0,1,0,0,1,0,0
,0,0,0,0,1,0,0,0,1,1,0,0,0,IR,S,IR,HR,IR,HR,IR,IR,HR,IR,HR,HR,IR
,HR,IR,S,S,1,0,0,0,0,0,0,1,0,0,1,S,cluster5
38,M,31100,36,1,BB,0,1,1,1,0,0,0,1,0,1,0,1,1,0,0,1,0,0,1,0,1,0,0
,0,1,0,0,1,1,0,0,0,0,1,0,0,HR,IR,HR,HR,HR,HR,HR,HR,HR,HR,HR,HR,H
R,HR,IR,HR,HR,0,0,0,0,1,1,0,1,0,0,1,HR,cluster2
46,F,5816,717,0,BB,0,0,0,0,0,0,0,0,0,0,0,0,0,0,0,0,0,1,0,0,0,0,0
,0,0,0,0,1,0,0,0,0,0,0,0,0,S,S,S,S,S,S,S,S,HR,LR,LR,IR,IR,HR,S,H
R,IR,1,0,0,1,0,0,0,0,1,0,0,HR,cluster0
46,M,15046,233,1,BB,1,0,0,1,0,0,1,1,0,0,0,0,1,0,0,0,0,0,1,0,1,0,
0,0,0,0,0,1,1,0,0,0,0,1,0,0,IR,S,IR,HR,IR,HR,IR,LR,HR,IR,HR,IR,I
R,HR,LR,S,S,1,0,0,1,0,0,0,1,0,0,0,S,cluster5
40,M,3343,546,1,BB,0,0,0,0,0,0,0,0,0,0,0,0,0,0,0,0,0,0,0,0,0,0,0
,0,0,0,0,1,0,0,0,0,0,0,1,0,S,S,S,S,S,S,S,S,HR,LR,S,S,S,HR,S,HR,I
R,1,0,0,1,0,0,0,0,0,0,0,HR,cluster1
48,M,8766,279,1,BB,0,0,0,0,0,0,0,0,0,0,0,0,0,0,0,0,0,0,0,0,0,0,0
,0,0,0,0,1,0,0,0,0,0,0,1,0,S,S,S,S,S,S,S,S,HR,LR,S,S,S,HR,S,HR,L
R,1,0,0,1,0,0,0,0,0,0,0,HR,cluster1
41,F,10949,72,1,BF,0,0,0,0,1,0,1,1,0,1,0,1,1,0,0,1,0,0,1,1,1,0,0
,0,1,0,0,1,1,0,0,0,0,1,0,0,HR,S,IR,HR,HR,HR,HR,LR,HR,HR,HR,HR,HR
,HR,IR,IR,LR,1,0,0,1,0,0,0,1,0,0,1,HR,cluster2
39,F,7695,26,1,BB,0,0,0,0,0,0,0,0,0,0,0,0,0,0,0,0,0,0,0,0,0,0,0,
0,0,0,0,1,1,0,0,0,0,0,0,0,S,S,S,S,S,S,S,S,HR,IR,LR,LR,LR,HR,S,S,
S,1,0,0,0,0,0,0,1,0,0,1,S,cluster0
49,F,8016,1056,0,BF,0,0,0,0,0,0,0,0,0,0,0,0,0,0,0,1,0,0,1,0,0,0,
0,0,1,0,0,1,0,0,0,1,0,0,0,0,S,S,S,S,S,S,S,S,HR,HR,HR,HR,HR,HR,IR
,IR,LR,1,0,0,1,0,0,0,0,0,0,0,HR,cluster0
45,F,23725,57,0,F1,0,0,0,0,0,0,1,1,0,1,1,0,0,1,1,1,0,0,1,0,1,1,0
,0,1,0,0,1,1,0,0,0,0,1,0,0,IR,S,IR,HR,IR,HR,HR,LR,HR,HR,HR,HR,HR
,HR,IR,HR,IR,1,0,0,1,0,1,0,0,0,0,0,HR,cluster2
50,M,9615,292,1,BB,0,0,0,0,0,0,0,0,0,0,0,0,0,0,0,0,0,0,0,0,0,0,0
,0,0,0,0,1,0,0,0,0,0,0,0,0,S,S,S,S,S,S,S,S,HR,LR,S,S,S,HR,S,HR,L
R,1,0,0,1,0,0,0,0,1,0,0,HR,cluster1
40,F,6437,108,1,BB,0,0,0,0,0,0,0,0,0,0,0,0,0,0,1,0,1,0,0,0,1,0,0
,0,0,0,0,1,0,0,0,0,0,0,0,0,S,S,S,S,S,LR,S,S,HR,LR,LR,S,S,HR,S,HR
,LR,1,0,0,1,0,0,0,0,1,0,0,HR,cluster1
43,M,4037,878,1,BB,0,0,0,0,0,0,0,0,0,0,0,0,0,0,0,1,0,0,0,1,1,0,0
,0,1,0,0,0,1,0,0,0,1,1,0,0,S,S,S,S,S,S,S,S,LR,HR,HR,HR,HR,LR,IR,
S,S,1,0,0,1,0,0,0,0,0,0,0,S,cluster0
49,F,140685,15,1,BB,0,0,0,1,0,0,1,1,0,1,1,0,0,1,0,1,0,0,1,0,0,0,
0,0,1,0,0,1,0,0,0,1,0,0,0,0,HR,LR,HR,HR,HR,HR,HR,IR,HR,HR,HR,HR,
HR,HR,IR,HR,HR,1,0,1,0,0,0,0,0,0,0,1,HR,cluster2
45,M,119193,219,1,BF,0,0,0,0,1,0,1,0,0,1,0,1,0,0,1,1,0,0,0,0,0,0
,0,0,0,0,0,1,0,0,0,1,0,0,0,0,HR,S,IR,HR,IR,HR,HR,LR,HR,IR,IR,IR,
IR,HR,LR,S,S,1,0,0,1,0,0,0,1,0,0,0,S,cluster2
33,F,4795,704,1,F1,0,1,0,1,0,0,0,0,0,0,0,1,0,1,0,0,0,0,1,0,1,0,0
,0,0,0,0,1,0,0,0,0,1,0,0,1,IR,LR,IR,IR,LR,IR,LR,S,HR,LR,IR,LR,LR
,HR,S,HR,IR,1,0,0,1,0,0,0,0,0,0,1,HR,cluster3
35,M,7193,646,1,F1,0,0,0,1,0,0,1,1,0,0,1,0,0,0,0,0,0,0,0,0,0,0,0
,0,1,0,0,1,1,0,0,0,0,0,0,0,IR,LR,HR,HR,HR,HR,LR,IR,HR,IR,LR,LR,L
R,HR,S,S,S,1,0,0,0,1,0,0,1,0,0,0,S,cluster5
34,M,25923,157,1,BB,0,0,0,0,0,1,0,0,0,0,0,0,0,1,0,0,0,0,0,1,1,0,
0,0,0,0,0,1,0,0,0,0,0,1,0,0,IR,LR,IR,LR,LR,HR,LR,S,HR,LR,IR,LR,L
R,HR,S,S,S,1,0,1,1,0,0,0,0,0,0,0,S,cluster5

40,M,8327,241,0,BB,0,1,0,1,0,0,0,0,0,1,1,0,0,1,0,0,0,0,1,0,1,0,0
,0,0,0,0,1,0,0,0,0,0,1,0,0,IR,IR,HR,HR,HR,HR,IR,LR,HR,LR,IR,LR,L
R,HR,S,S,S,1,0,0,0,0,0,0,1,0,0,1,S,cluster5
40,M,199707,164,1,F1,1,0,1,0,1,0,0,1,0,0,1,1,0,1,0,1,0,0,1,0,0,0
,0,0,1,0,0,1,0,0,0,1,0,0,0,0,IR,S,IR,HR,HR,HR,IR,IR,HR,HR,HR,HR,
HR,HR,IR,LR,S,1,0,0,0,1,0,0,0,0,0,0,LR,cluster2
43,M,7663,300,0,BB,0,0,0,0,0,0,0,0,0,0,0,0,0,0,0,1,0,0,1,0,0,0,0
,0,1,0,0,0,0,0,0,1,0,0,0,0,0,S,S,S,S,S,S,S,S,LR,IR,HR,HR,IR,LR,IR,
HR,IR,0,0,0,1,0,1,0,0,1,0,0,HR,cluster0
32,F,8106,260,1,F1,0,0,0,0,0,0,0,0,0,0,0,1,0,0,1,0,0,0,0,0,0,0,0
,0,0,0,0,0,0,0,0,0,0,0,0,0,S,S,S,S,S,LR,S,S,S,S,S,S,S,S,S,S,1,
1,0,0,1,0,0,0,0,0,0,S,cluster4
33,F,109829,42,0,BF,0,0,0,0,0,0,0,0,0,0,0,0,0,0,0,0,0,0,0,0,0,0,
0,0,0,0,0,1,0,0,0,0,0,0,0,0,S,S,S,S,S,S,S,S,HR,LR,S,S,LR,HR,S,HR
,LR,1,0,0,1,0,0,0,0,0,0,0,HR,cluster1
48,M,6952,248,1,BF,0,1,0,0,0,0,0,0,0,0,0,0,0,1,0,1,0,0,1,1,1,0,0
,0,1,0,0,1,1,0,0,0,1,0,0,0,0,LR,IR,HR,LR,HR,IR,S,LR,HR,IR,HR,HR,IR
,HR,IR,S,S,1,0,0,0,0,0,0,1,0,0,1,S,cluster3
45,F,56287,120,0,BB,0,0,0,1,0,0,1,0,1,1,0,0,0,1,0,1,0,0,1,0,0,0,
0,0,1,0,0,1,0,0,0,1,0,0,0,0,HR,LR,HR,HR,IR,HR,HR,IR,HR,HR,HR,HR,
HR,HR,IR,IR,IR,1,0,0,1,0,0,0,1,0,0,0,HR,cluster2
43,M,148104,11,1,BF,1,0,1,0,1,0,1,1,1,0,1,0,0,0,0,1,0,0,1,0,0,0,
0,0,1,0,0,1,0,0,0,1,0,0,0,0,HR,LR,HR,HR,HR,HR,HR,IR,HR,HR,HR,HR,
HR,HR,IR,S,S,1,0,0,1,0,0,0,1,0,0,0,S,cluster2
37,M,14611,67,1,F1,1,0,1,0,1,0,1,1,0,0,1,0,0,0,0,1,0,0,0,0,0,0,0
,0,1,0,0,1,0,0,0,1,0,0,0,0,IR,S,IR,HR,IR,HR,IR,IR,HR,HR,IR,IR,HR
,HR,IR,HR,HR,1,0,0,0,1,0,0,0,0,0,0,HR,cluster2
57,M,81337,589,1,BB,0,0,1,0,0,0,0,1,0,0,0,0,0,1,0,1,0,0,0,0,0,0,
0,0,1,0,0,0,0,0,0,1,0,0,0,0,HR,S,LR,IR,LR,HR,LR,LR,LR,IR,HR,IR,I
R,LR,IR,HR,IR,0,0,1,1,0,1,0,0,0,0,0,HR,cluster3
55,F,8197,161,1,BB,0,0,0,0,0,0,0,0,0,0,1,0,0,0,1,0,1,0,0,0,0,0,0
,0,0,0,0,0,0,0,0,1,0,0,0,0,HR,S,S,IR,LR,HR,IR,S,S,IR,IR,IR,IR,S,
IR,S,S,1,0,0,0,1,0,0,0,0,1,0,S,cluster3
70,F,18155,282,0,BB,0,1,0,1,0,0,1,1,0,1,1,0,0,1,0,1,0,0,1,0,0,0,
0,0,1,0,0,1,0,0,0,1,0,0,0,0,HR,IR,HR,HR,HR,HR,HR,IR,HR,IR,HR,HR,
IR,HR,LR,HR,HR,1,0,0,0,0,0,0,1,0,0,1,HR,cluster2
37,M,7776,416,0,BB,0,0,0,0,0,0,1,0,0,0,0,0,0,0,0,1,0,0,0,0,0,0,0
,0,1,0,0,1,0,0,1,1,0,0,0,0,LR,S,LR,LR,LR,LR,LR,LR,HR,HR,HR,IR,HR
,HR,IR,HR,HR,1,0,0,1,0,0,0,0,0,0,0,HR,cluster3
36,M,180630,80,0,F1,0,0,1,1,0,0,0,0,0,1,0,1,0,0,1,1,0,0,0,0,0,1,
0,0,0,0,0,1,0,0,0,1,0,0,0,0,HR,IR,HR,HR,IR,HR,HR,IR,HR,HR,IR,IR,
HR,HR,LR,HR,LR,1,0,0,0,0,0,0,0,0,0,1,HR,cluster2
52,M,292098,42,1,BB,0,0,1,1,0,0,1,0,0,1,0,1,1,0,0,1,0,0,1,0,0,0,
0,0,1,0,0,1,0,0,0,1,0,0,0,0,HR,LR,HR,HR,HR,HR,HR,IR,HR,HR,HR,HR,
HR,HR,IR,HR,LR,1,0,0,1,0,0,0,1,0,0,0,HR,cluster2
44,F,6496,442,0,BF,0,0,0,0,0,0,0,0,0,0,0,0,0,0,0,0,0,0,0,0,0,0,0
,0,0,0,0,1,0,0,0,1,0,0,1,0,S,S,S,S,S,S,S,S,HR,IR,LR,LR,LR,HR,LR,
HR,LR,1,0,0,1,0,0,0,0,0,0,1,HR,cluster0
48,M,14609,290,0,BB,1,0,1,0,1,0,1,1,0,0,0,1,0,0,0,1,0,0,0,0,0,0,
0,0,1,0,0,1,0,0,0,1,0,0,0,0,IR,S,IR,HR,IR,HR,IR,IR,HR,HR,HR,HR,H
R,HR,IR,S,S,1,0,0,0,0,0,0,1,0,0,0,S,cluster2
43,M,96583,40,0,BB,0,0,0,1,0,0,1,0,0,1,1,0,0,1,0,1,0,0,1,0,0,0,0
,0,1,0,0,1,0,0,0,1,0,0,0,0,HR,IR,HR,HR,HR,HR,HR,LR,HR,HR,HR,HR,H
R,HR,IR,HR,LR,1,0,0,0,0,0,0,1,0,0,1,HR,cluster2

46,M,105551,154,0,BB,0,0,0,0,0,0,0,0,0,0,0,0,0,0,0,0,0,0,0,0,0
,0,0,0,0,0,1,0,0,0,0,0,0,0,0,S,S,S,S,S,S,S,S,HR,LR,S,S,S,HR,S,HR
,IR,1,0,0,1,0,0,0,0,0,0,0,HR,cluster1
39,M,10075,127,1,BB,0,0,0,0,0,0,0,0,0,0,0,0,0,0,0,0,0,0,0,0,0,
0,0,0,0,0,1,0,0,0,0,0,0,0,0,S,S,S,S,S,S,S,S,HR,LR,S,S,S,HR,S,HR,
LR,1,0,0,0,1,0,0,0,0,0,0,HR,cluster1
27,M,26988,156,0,BB,0,0,0,0,0,0,0,0,0,0,0,0,0,0,0,0,0,0,0,0,0,
0,0,0,0,0,1,0,0,0,0,0,0,0,0,S,S,S,S,S,S,S,S,HR,LR,S,S,S,HR,S,HR,
LR,1,0,0,1,0,0,0,0,1,0,0,HR,cluster1
38,M,151761,231,1,F1,1,0,1,0,1,0,1,1,1,0,0,1,0,1,0,1,0,0,0,0,0,0
,0,0,1,0,0,1,0,0,0,1,0,0,0,0,HR,LR,HR,HR,HR,HR,HR,IR,HR,HR,HR,HR
,HR,HR,IR,HR,HR,1,0,0,0,1,0,0,0,0,0,1,HR,cluster2
45,M,24502,76,1,BF,0,0,0,0,0,0,0,0,0,0,1,0,1,0,0,0,1,0,0,0,0,0,0
,0,1,0,0,0,0,0,0,0,0,0,0,0,S,S,S,S,S,S,S,S,IR,IR,S,LR,IR,IR,IR,H
R,IR,0,0,0,0,0,0,0,1,0,0,1,HR,cluster0
34,F,5466,667,0,BB,0,0,0,0,0,0,0,0,0,0,0,0,0,0,0,0,0,0,0,0,0,0,0
,0,0,0,0,1,0,0,0,0,0,0,0,0,S,S,S,S,S,S,S,S,HR,LR,S,S,S,HR,S,HR,I
R,1,0,0,1,0,0,0,0,1,0,0,HR,cluster1
32,F,10213,478,0,BF,0,0,0,0,0,0,0,0,0,0,0,0,0,0,0,0,0,0,0,0,0,
0,0,0,0,0,0,0,0,0,0,0,0,0,0,S,S,S,S,S,S,S,S,S,S,S,S,S,S,S,S,S,1,
0,0,1,0,0,0,0,1,0,0,S,cluster4
43,M,32108,184,0,F1,0,0,0,0,0,0,0,0,0,0,0,0,0,0,0,0,0,0,0,0,0,
0,0,0,0,0,0,0,0,0,0,0,0,0,0,S,S,S,S,S,S,S,S,S,S,S,S,S,S,S,S,S,1,
0,0,0,0,0,0,1,0,0,1,S,cluster4
33,F,16062,368,0,F1,0,0,0,0,0,0,0,0,0,0,0,0,0,0,0,0,0,1,0,1,0,
0,0,0,0,0,1,0,0,0,0,0,0,0,0,S,S,S,S,S,S,S,S,HR,LR,IR,LR,IR,HR,LR
,HR,LR,1,0,0,0,1,0,0,0,1,0,0,HR,cluster0
40,M,128000,60,0,BF,0,0,0,0,0,0,0,1,0,0,0,1,0,0,0,0,0,0,0,0,0,
0,0,0,0,0,0,0,0,0,0,0,0,0,0,LR,S,LR,IR,LR,IR,LR,S,S,S,S,S,S,S,S,
S,S,1,0,1,1,0,0,0,0,0,0,0,S,cluster4
32,F,6812,376,0,BB,0,1,0,0,0,0,0,0,0,1,0,0,0,0,0,0,0,0,0,0,0,0,0
,0,0,0,0,1,0,0,0,0,0,0,0,0,IR,LR,IR,IR,LR,HR,IR,S,HR,LR,S,S,S,HR
,S,IR,IR,1,1,0,1,0,0,0,0,0,0,0,HR,cluster1
55,M,3254,389,?,BB,0,0,0,1,0,0,0,1,0,0,1,0,1,0,0,1,0,0,1,0,0,0,0
,0,1,0,0,1,0,0,0,1,0,0,0,0,HR,S,LR,HR,IR,HR,LR,LR,HR,HR,HR,HR,HR
,HR,IR,S,S,1,0,1,1,0,0,0,0,0,0,0,S,cluster3
35,F,6687,401,0,BB,0,0,0,0,0,0,0,0,0,0,0,1,0,0,0,0,0,0,0,0,1,0,0
,0,0,0,0,1,0,0,0,0,0,0,1,0,S,S,S,S,S,S,S,S,HR,LR,LR,S,S,HR,S,HR,
LR,1,0,0,1,0,0,0,0,0,0,0,HR,cluster1
31,F,190164,73,1,BB,0,1,1,0,1,0,0,0,0,0,0,1,1,0,0,0,1,0,0,0,0,0,
1,1,0,1,0,1,0,0,0,0,1,0,0,0,HR,LR,HR,IR,IR,HR,LR,LR,HR,HR,HR,HR,
HR,HR,HR,HR,HR,1,1,0,0,0,0,0,0,0,1,1,HR,cluster3
42,M,28819,437,0,BF,0,0,0,0,0,0,0,0,0,0,0,0,0,0,0,0,0,0,1,1,0,
0,0,0,0,0,1,0,0,0,0,0,1,0,0,LR,S,S,S,S,HR,LR,S,HR,LR,LR,LR,LR,HR
,S,HR,IR,1,0,0,0,1,0,0,0,0,0,0,HR,cluster1
43,M,40638,414,0,BB,0,1,1,1,0,0,0,1,1,0,0,0,0,1,0,1,0,0,0,0,0,0,
0,0,0,0,0,1,0,0,0,1,0,0,0,0,HR,IR,HR,HR,HR,HR,HR,IR,HR,IR,IR,IR,
IR,HR,IR,HR,IR,1,0,0,0,1,0,0,0,0,0,0,HR,cluster2
31,F,249788,59,1,BB,0,0,1,0,0,0,0,1,0,0,0,1,0,0,0,1,0,0,1,0,1,0,
0,0,0,0,0,1,1,0,0,0,1,0,0,0,HR,S,LR,IR,IR,IR,LR,LR,HR,IR,HR,HR,H
R,HR,IR,S,S,1,1,0,0,0,0,0,0,0,0,1,S,cluster3
34,M,104546,52,1,BF,0,1,1,1,0,1,0,1,0,0,1,0,0,1,1,1,0,0,1,0,0,0,
0,0,1,0,0,1,0,0,0,1,0,0,0,0,HR,IR,HR,HR,HR,HR,IR,IR,HR,HR,HR,HR,
HR,HR,IR,S,S,1,0,0,0,0,0,0,1,0,0,1,S,cluster2

22,F,45232,162,1,BF,0,0,0,0,0,0,0,0,0,0,1,0,0,0,0,0,0,0,0,0,0,0,
0,0,0,0,0,0,0,0,0,0,0,0,0,0,S,S,S,S,S,S,S,S,S,S,S,S,S,S,S,S,1,
1,0,1,0,0,0,0,0,0,0,S,cluster4
32,M,12447,289,1,BF,0,0,0,0,0,0,0,0,0,0,1,0,0,0,1,0,0,1,1,1,0,
0,0,0,0,0,1,1,1,1,0,0,1,0,0,S,S,S,S,S,S,S,S,HR,HR,HR,HR,HR,HR,IR
,HR,LR,1,0,0,1,0,0,0,0,0,0,0,HR,cluster0
42,F,12249,268,1,BB,0,0,0,0,0,0,0,0,0,0,0,0,0,0,0,1,0,0,0,0,0,0,
0,0,0,0,0,0,0,0,0,0,0,0,0,0,S,S,S,S,S,S,S,S,S,LR,LR,LR,LR,S,LR,S
,S,1,1,0,0,0,0,0,0,0,1,1,S,cluster4
49,M,8517,639,1,BB,0,0,0,0,0,0,0,0,0,0,1,0,0,0,0,1,0,0,0,0,0,0,0
,0,0,0,0,1,0,0,0,1,0,0,0,0,S,S,S,S,S,S,S,S,HR,IR,IR,IR,IR,HR,LR,
IR,LR,1,0,0,1,0,0,0,0,0,0,0,HR,cluster0
36,F,11252,400,1,BB,0,0,1,1,0,0,0,0,0,0,0,0,1,0,0,1,0,0,0,0,0,0,
0,0,0,0,0,1,0,0,0,1,0,0,0,0,HR,S,LR,S,LR,IR,S,LR,HR,IR,IR,IR,IR,
HR,LR,S,S,1,1,0,1,0,0,0,0,0,0,0,S,cluster3
45,M,5857,842,1,BB,0,0,0,0,0,0,0,0,0,0,0,0,1,0,0,0,0,0,1,0,1,0,0
,0,1,0,0,1,1,0,0,0,1,1,0,0,S,S,S,S,S,S,S,S,HR,HR,HR,HR,HR,HR,IR,
HR,IR,1,0,0,1,0,0,0,0,1,0,0,HR,cluster0
57,F,3551,982,1,BB,0,0,0,0,0,0,0,0,0,0,1,0,0,0,0,1,0,0,1,1,1,0,0
,0,0,0,0,1,0,0,0,1,1,0,0,0,S,S,S,S,S,S,S,S,HR,IR,HR,HR,IR,HR,IR,
HR,HR,1,0,0,1,0,0,0,0,1,0,0,HR,cluster0
37,F,38420,945,1,BB,0,0,0,0,0,0,0,0,0,0,0,0,0,0,0,0,0,0,0,0,0,0,
0,0,0,0,0,1,0,0,0,0,0,0,0,0,HR,S,S,S,S,S,S,LR,HR,LR,S,S,S,HR,S,S
,S,1,1,0,1,0,0,0,0,0,0,0,S,cluster1
46,F,4108,711,1,BB,0,0,0,0,1,0,0,0,0,0,0,0,0,0,0,0,0,0,0,0,0,0,0
,0,1,0,0,0,0,0,0,0,0,0,0,0,LR,S,LR,LR,LR,IR,S,S,S,S,S,S,S,S,S,IR
,LR,1,0,0,1,0,0,0,0,0,0,0,HR,cluster4
40,M,45837,656,?,BD,1,0,0,1,0,0,0,1,0,0,1,0,0,1,0,1,0,0,1,0,0,0,
0,0,1,0,0,1,0,0,0,1,0,0,0,0,IR,S,IR,HR,IR,HR,IR,LR,HR,HR,HR,HR,I
R,HR,IR,HR,IR,1,1,0,0,1,0,0,0,0,0,0,HR,cluster2
50,F,3938,286,1,BB,0,0,0,1,0,0,1,1,0,0,0,0,0,0,0,0,0,0,0,0,0,0,0
,0,0,0,0,1,0,0,0,0,0,0,0,0,IR,S,IR,HR,IR,HR,IR,LR,HR,LR,S,S,S,HR
,S,S,S,1,0,0,1,0,0,0,1,0,0,0,S,cluster5
36,F,1161,964,1,BB,0,0,0,0,0,0,0,0,0,0,0,0,0,0,0,1,0,0,0,0,0,0,0
,0,0,0,0,1,1,0,0,0,0,0,0,0,S,S,S,S,S,S,S,S,HR,IR,IR,IR,IR,HR,LR,
HR,LR,1,0,0,1,0,0,0,0,0,0,0,HR,cluster0
59,M,33377,137,1,BB,0,0,0,0,0,1,0,1,0,1,0,0,0,1,0,0,0,0,1,1,1,0,
0,0,0,0,0,1,1,0,0,0,0,1,0,0,HR,LR,HR,HR,IR,HR,HR,S,HR,IR,HR,HR,I
R,HR,LR,HR,LR,1,0,0,0,0,0,0,0,0,0,1,HR,cluster2
34,F,14048,90,1,BB,0,0,0,0,0,0,0,0,0,0,1,0,0,0,0,0,0,0,0,0,0,0,0
,0,0,0,0,0,0,0,0,0,0,0,0,0,S,S,S,S,S,S,S,S,S,S,S,S,S,S,S,S,1,0
,0,1,0,0,0,1,0,0,0,S,cluster4
50,M,19707,413,1,BB,0,0,0,1,0,0,1,1,0,1,1,0,0,1,0,1,0,0,0,0,0,0,
0,0,0,0,0,1,1,0,0,1,0,0,0,0,HR,S,IR,HR,HR,HR,HR,IR,HR,IR,IR,IR,I
R,HR,LR,S,S,1,0,1,1,0,0,0,0,0,0,0,LR,cluster5
51,M,36317,15,1,BB,1,0,1,1,0,0,1,1,0,0,1,0,0,0,0,0,1,0,1,1,1,0,0
,0,1,1,0,1,1,0,0,0,0,1,0,0,IR,LR,HR,HR,HR,HR,IR,LR,HR,HR,HR,HR,H
R,HR,IR,S,S,1,0,0,0,0,0,0,1,0,0,1,S,cluster2
49,F,60898,423,1,BF,0,0,0,0,0,0,0,0,0,0,0,0,0,0,0,0,0,0,0,0,0,0,
0,0,1,0,0,0,0,0,0,0,0,0,0,0,S,S,S,S,S,S,S,S,S,S,S,S,S,S,S,S,1,
0,1,0,0,0,0,0,0,0,1,S,cluster4
52,F,231250,443,1,BB,0,0,0,0,0,0,0,0,0,0,0,0,0,0,0,1,0,0,0,0,0,0
,0,0,0,0,0,1,0,0,0,1,0,0,0,0,S,S,S,S,S,S,S,S,HR,IR,IR,IR,IR,HR,I
R,HR,IR,1,0,0,1,0,0,0,0,0,0,0,HR,cluster0

39,F,141891,154,1,BB,0,0,0,0,0,0,0,0,0,0,0,0,0,1,0,0,0,0,0,0,0,0
,0,0,0,0,0,1,0,0,0,0,0,0,1,0,S,S,S,S,S,S,S,S,HR,LR,S,S,S,HR,S,HR
,LR,1,0,0,1,0,0,0,0,0,0,0,HR,cluster1
51,M,12000,348,1,BB,0,0,0,0,0,0,0,0,0,0,0,0,0,0,1,0,0,1,0,0,0,
0,0,1,0,0,1,0,0,0,1,1,0,0,0,S,S,S,S,S,S,S,S,HR,HR,HR,HR,IR,HR,IR
,HR,LR,1,0,0,0,1,0,0,0,0,0,0,HR,cluster0
49,M,18877,199,1,BB,0,0,0,0,0,0,0,0,0,0,0,0,0,1,0,0,0,0,0,0,0,0,
0,0,0,0,0,1,0,0,0,0,0,0,0,0,S,S,S,S,S,S,S,S,HR,LR,S,S,S,HR,S,LR,
LR,1,0,0,1,0,0,0,1,0,0,0,LR,cluster1
44,F,1584,259,0,BB,0,0,0,0,0,0,0,0,0,0,0,0,0,0,0,0,0,0,0,0,0,0,0
,0,0,0,0,1,0,0,0,0,0,0,0,0,S,S,S,S,S,S,S,S,HR,LR,S,S,S,HR,S,S,S,
1,0,0,1,0,0,0,1,0,0,0,S,cluster1
39,M,10741,252,1,BB,0,0,0,0,0,0,0,0,1,1,0,0,1,0,0,1,1,0,0,0,0,0,
0,0,0,0,0,1,0,0,0,1,0,0,0,0,HR,LR,IR,HR,IR,HR,HR,LR,HR,IR,IR,IR,
IR,HR,LR,S,S,1,0,1,0,0,0,0,0,0,0,1,S,cluster5
37,F,68904,123,1,BB,0,0,0,1,0,0,1,0,0,0,1,0,0,0,0,1,0,0,0,0,0,0,
0,0,1,0,0,1,0,0,0,1,0,0,0,0,IR,LR,HR,HR,HR,HR,LR,S,HR,HR,HR,HR,H
R,HR,IR,S,S,1,0,0,0,0,0,0,1,0,0,1,S,cluster2
38,M,9027,302,0,BB,0,0,0,1,0,0,0,0,0,0,0,0,0,0,0,0,0,0,0,0,0,0,0
,0,0,0,0,0,0,0,0,0,0,0,0,0,LR,S,LR,LR,LR,IR,S,S,S,S,S,S,S,S,S,S,
S,1,0,1,0,0,0,0,0,0,0,1,S,cluster4
47,M,43677,492,1,BB,0,0,0,0,0,0,0,0,0,0,1,0,0,0,0,0,0,0,0,0,0,0,
0,0,0,0,0,1,0,0,0,0,0,0,0,0,S,S,S,S,S,S,S,S,HR,LR,S,S,S,HR,S,S,S
,1,0,1,0,1,0,0,0,0,0,0,S,cluster1
44,M,8862,131,1,BB,0,1,0,0,1,0,0,1,0,1,0,1,0,1,0,0,0,0,0,0,0,0,0
,0,0,0,0,1,1,0,0,1,0,0,0,0,HR,LR,HR,HR,HR,HR,IR,LR,HR,IR,LR,LR,L
R,HR,LR,HR,LR,1,0,1,0,0,0,0,0,0,0,1,HR,cluster5
39,M,14148,5,1,BB,0,0,0,0,0,0,0,0,0,0,0,0,0,0,0,0,0,0,0,0,0,0,
0,0,0,0,1,0,0,0,0,0,0,0,0,S,S,S,S,S,S,S,S,HR,LR,S,S,S,HR,S,S,S,1
,1,0,1,0,0,0,0,0,0,0,S,cluster1
36,M,21398,368,1,BB,0,0,1,1,0,0,0,1,0,1,0,0,1,1,0,0,0,0,0,0,0,0,
0,0,0,0,0,1,1,0,0,0,0,0,0,0,HR,S,IR,HR,IR,HR,HR,LR,HR,IR,LR,LR,L
R,HR,S,IR,IR,1,0,0,0,0,0,0,1,0,0,1,HR,cluster5
41,F,4883,321,1,BB,0,0,0,0,1,0,0,0,0,1,1,0,0,0,0,1,0,0,0,0,0,0,0
,0,0,0,0,1,0,0,0,1,0,0,0,0,IR,S,IR,IR,LR,HR,IR,LR,HR,HR,IR,IR,HR
,HR,IR,HR,HR,1,0,0,1,0,0,0,0,0,0,0,HR,cluster3
19,M,75506,10,1,BB,0,0,0,1,0,0,1,0,1,0,1,0,0,1,0,1,0,0,1,0,1,1,0
,0,1,0,0,1,0,0,0,1,1,0,0,0,HR,LR,HR,HR,HR,HR,HR,IR,HR,HR,HR,HR,H
R,HR,IR,HR,IR,1,0,0,0,0,0,0,1,0,0,1,HR,cluster2
54,M,4257,327,1,BB,0,0,0,1,0,0,1,0,0,0,1,0,0,0,1,0,1,0,0,1,1,0,0
,0,0,1,0,1,0,0,0,0,1,0,0,0,LR,LR,IR,IR,IR,HR,LR,S,HR,HR,HR,HR,HR
,HR,HR,HR,LR,1,0,0,0,0,0,0,1,0,0,1,HR,cluster3
34,F,3789,447,1,BB,0,0,0,0,0,0,0,0,0,0,0,0,0,0,0,0,0,0,0,0,0,0,0
,0,0,0,0,1,0,0,0,0,0,0,0,0,S,S,S,S,S,S,S,S,HR,LR,S,S,S,HR,S,HR,L
R,1,0,0,0,1,0,0,0,1,0,0,HR,cluster1
32,F,41867,707,1,BB,0,0,0,0,1,0,0,0,0,0,0,1,0,0,0,1,0,0,1,1,1,0,
0,0,0,0,0,1,0,0,0,1,1,0,0,0,IR,LR,IR,LR,HR,HR,LR,LR,HR,HR,HR,HR,
HR,HR,IR,S,S,1,0,0,1,0,0,0,1,0,0,0,S,cluster3
47,M,10598,103,1,BF,0,0,0,0,0,0,0,0,0,0,0,0,0,0,0,0,0,0,1,0,1,0,
0,0,0,0,0,1,0,0,0,1,0,1,1,0,S,S,S,S,S,S,S,S,HR,IR,HR,IR,IR,HR,LR
,HR,LR,1,0,0,1,0,0,0,0,0,0,0,HR,cluster0
59,M,28993,194,1,BB,0,0,0,1,0,0,0,1,0,0,0,0,0,0,1,1,0,0,1,0,0,0,
0,0,0,0,0,1,0,0,0,1,0,0,0,0,IR,S,IR,HR,IR,HR,IR,IR,HR,HR,HR,HR,H
R,HR,IR,IR,IR,1,0,0,0,0,0,0,0,1,0,1,HR,cluster2

32,F,52801,316,1,BB,0,0,0,0,0,0,0,0,0,0,1,0,0,0,0,0,0,0,0,1,0,0,
0,0,0,0,0,0,0,0,0,0,0,0,0,0,S,S,S,S,S,S,S,S,S,S,S,LR,LR,S,S,IR,I
R,1,1,0,1,0,0,0,0,0,0,0,HR,cluster4
39,F,3270,366,1,BB,0,0,0,1,0,0,0,0,0,0,0,0,0,0,1,0,0,0,0,0,0,0
,0,1,0,0,1,0,0,0,1,0,0,0,0,LR,S,LR,LR,LR,IR,S,S,HR,HR,HR,IR,IR,H
R,IR,HR,IR,1,0,0,0,0,0,0,0,0,0,1,HR,cluster3
36,F,134965,192,0,BB,0,0,0,0,0,0,0,0,0,0,1,0,0,0,0,0,0,0,0,0,0,0
,0,0,0,0,0,0,0,0,0,0,0,0,0,0,S,S,S,S,S,S,S,S,S,S,S,S,S,S,S,S,1
,0,0,1,0,0,0,1,0,0,0,S,cluster4
34,M,87270,135,0,BF,0,0,0,0,0,0,0,0,0,0,0,0,0,0,0,0,0,0,0,0,0,0,
0,0,0,0,0,1,0,0,0,1,0,0,0,0,HR,S,S,S,S,S,S,S,HR,IR,LR,LR,LR,HR,L
R,S,S,1,1,0,1,0,0,0,0,0,0,0,S,cluster0
45,M,1704,478,1,BB,0,0,0,0,0,0,0,0,0,0,0,0,0,0,0,0,0,0,0,0,0,0
,0,0,0,0,0,0,0,0,0,0,0,0,0,0,S,S,S,S,S,S,S,S,S,S,S,S,S,S,S,S,1,0
,1,0,0,0,0,0,0,0,1,S,cluster4
67,F,18974,482,0,BB,0,0,1,0,0,0,0,0,0,0,0,0,1,0,1,0,0,0,0,0,1,
0,0,0,0,0,1,0,0,0,1,0,0,0,0,HR,S,S,S,S,HR,S,S,HR,HR,IR,IR,HR,HR,
IR,HR,IR,0,0,0,0,0,0,0,1,0,0,0,HR,cluster0
55,M,11983,124,1,BF,0,0,1,0,0,0,0,0,0,1,0,0,0,0,0,0,0,0,0,0,0,0,
0,0,0,0,0,1,0,0,0,0,0,0,0,0,HR,S,LR,IR,LR,HR,IR,LR,HR,LR,S,S,S,H
R,S,S,S,1,0,0,1,0,0,0,0,0,0,0,S,cluster5
59,M,98706,12,1,BB,0,0,0,0,0,0,0,0,0,0,1,0,0,0,0,0,0,0,0,0,0,0
,0,0,0,0,0,0,0,0,0,0,0,0,0,0,S,S,S,S,S,S,S,S,S,S,S,S,S,S,HR,LR,1
,0,0,1,0,0,0,1,0,0,0,HR,cluster4
46,M,15076,290,1,BB,0,0,0,1,1,0,1,0,0,0,0,1,0,1,1,1,0,0,1,0,0,0,
0,0,1,0,0,0,0,0,0,1,0,0,0,0,IR,S,IR,HR,IR,HR,IR,IR,LR,IR,HR,HR,H
R,LR,IR,HR,LR,0,0,0,0,1,1,0,1,0,0,0,HR,cluster2
41,M,17987,459,1,BB,0,0,0,0,0,0,0,0,0,0,0,0,0,0,0,0,0,0,0,0,0,0,
0,0,0,0,0,0,0,0,0,0,0,0,1,0,S,S,S,S,S,S,S,S,S,S,S,S,S,S,HR,LR,
1,1,0,1,0,0,0,0,0,0,0,HR,cluster4
45,F,54939,149,1,BB,0,0,0,0,0,0,0,0,0,0,1,0,0,0,0,1,0,0,1,0,0,0,
0,0,0,0,0,1,0,0,0,1,0,0,0,0,S,S,S,S,S,S,S,S,HR,HR,HR,HR,HR,HR,IR
,HR,IR,1,0,0,1,0,0,0,0,0,0,0,HR,cluster0
50,F,11634,214,1,BB,0,0,0,0,0,0,0,0,0,0,0,0,0,0,0,0,0,0,0,0,1,0,
0,0,0,0,0,1,0,0,0,0,0,0,1,0,S,S,S,S,S,S,S,S,HR,LR,LR,S,S,HR,S,HR
,LR,1,0,0,1,0,0,0,0,0,0,0,HR,cluster1
53,M,93995,34,1,BB,0,0,0,1,0,0,1,0,0,1,0,0,1,0,0,1,0,0,0,0,0,0
,0,1,0,0,1,0,0,0,1,0,0,1,0,HR,S,IR,HR,IR,HR,HR,LR,HR,HR,HR,HR,HR
,HR,IR,IR,LR,1,0,0,1,0,0,0,1,0,0,0,LR,cluster2
40,F,258611,201,1,BB,0,0,0,1,0,0,1,0,0,1,0,0,0,1,0,1,0,0,1,0,0,0
,0,0,1,0,0,1,0,0,0,1,0,0,0,0,HR,S,IR,HR,HR,HR,HR,IR,HR,HR,HR,HR,
HR,HR,IR,HR,IR,1,0,0,0,0,0,0,1,0,0,1,HR,cluster2
50,F,3316,259,1,BB,0,0,0,0,0,0,0,0,0,0,0,0,0,0,0,0,0,0,1,0,1,0,0
,0,0,0,0,1,1,0,0,0,0,1,0,0,S,S,S,S,S,S,S,S,HR,IR,HR,IR,IR,HR,LR,
HR,LR,1,0,0,1,0,0,0,0,1,0,0,HR,cluster0
46,M,65867,178,0,BB,0,0,0,0,0,0,0,0,0,0,0,0,0,0,0,0,0,0,0,0,0,0,
0,0,0,0,0,0,0,0,0,0,0,0,0,0,S,S,S,S,S,S,S,S,S,S,S,S,S,S,HR,LR,
1,0,0,0,0,0,0,1,0,0,1,HR,cluster4
45,F,154872,65,1,BB,0,0,0,1,0,0,0,0,1,0,0,0,1,0,0,1,0,0,0,0,0,0,
0,0,0,0,0,1,0,0,0,1,0,0,0,0,HR,LR,HR,HR,IR,HR,HR,IR,HR,IR,HR,HR,
IR,HR,IR,LR,S,1,0,0,0,0,0,0,1,0,0,1,LR,cluster2
30,M,53919,189,1,BF,0,0,0,0,0,0,0,0,0,0,0,0,0,0,0,1,0,0,0,0,0,0,
0,0,0,0,0,1,0,0,0,1,0,0,1,0,S,S,S,S,S,S,S,S,HR,HR,IR,IR,HR,HR,IR
,HR,LR,1,0,0,1,0,0,0,0,0,0,0,HR,cluster0

39,F,78762,179,1,BB,0,0,0,0,0,0,0,0,0,0,0,0,0,0,0,0,0,0,0,0,0,0,
0,0,0,0,0,1,0,0,0,1,0,0,0,0,S,S,S,S,S,S,S,S,HR,IR,LR,LR,LR,HR,LR
,HR,IR,1,0,0,1,0,0,0,0,0,0,0,HR,cluster0
32,M,11198,66,0,BB,0,0,0,0,0,0,0,0,0,0,0,0,0,0,0,0,0,1,1,1,0,0
,0,1,0,0,1,1,0,0,0,1,0,0,0,S,S,S,S,S,S,S,S,HR,IR,HR,HR,IR,HR,LR,
HR,IR,1,0,0,1,0,0,0,0,0,0,0,HR,cluster0
47,M,90402,45,1,BF,0,0,0,0,0,0,0,0,0,0,0,0,0,0,1,0,0,1,0,1,0,0
,0,0,0,0,1,1,0,0,1,1,0,1,0,S,S,S,S,S,S,S,S,HR,IR,HR,HR,IR,HR,IR,
HR,LR,1,0,0,1,0,0,0,0,0,0,0,HR,cluster0
48,F,33413,194,0,BD,0,0,0,0,0,0,0,0,0,0,1,0,0,0,0,0,0,0,0,0,0,0,
0,0,0,0,0,0,0,0,0,0,0,0,0,0,S,S,S,S,S,S,S,S,S,S,S,S,S,S,S,1,
0,0,0,0,0,0,1,0,0,1,S,cluster4
46,M,117694,34,1,BB,1,0,0,1,0,0,1,1,0,0,1,0,0,1,0,1,0,0,1,0,0,0,
0,0,0,0,0,1,0,0,0,1,0,0,0,0,HR,LR,HR,HR,HR,HR,IR,IR,HR,HR,HR,HR,
HR,HR,IR,HR,IR,1,0,0,0,0,0,0,1,0,0,1,HR,cluster2
48,M,10796,326,0,BB,1,0,1,0,1,0,1,1,0,0,0,0,0,1,0,1,1,0,0,0,0,0,
0,0,1,0,0,1,0,0,0,1,0,0,0,0,HR,LR,IR,HR,HR,HR,IR,IR,HR,HR,IR,IR,
HR,HR,IR,S,S,1,0,0,0,0,0,0,1,0,0,1,S,cluster2
30,M,28588,175,1,BF,0,0,0,0,0,0,0,0,0,0,0,0,0,0,0,0,0,0,0,1,0,
1,1,0,1,0,0,0,1,0,0,0,1,1,0,S,S,S,S,S,S,S,S,IR,HR,HR,HR,HR,IR,HR
,HR,LR,1,0,0,0,0,0,0,0,0,0,1,HR,cluster0
44,M,12787,17,1,F1,0,0,0,0,0,0,0,0,0,0,0,0,0,0,0,0,0,1,0,1,0,0
,0,0,0,0,0,1,0,0,0,1,0,0,0,S,S,S,S,S,S,S,S,S,LR,HR,HR,LR,S,LR,S,
S,1,1,0,1,0,0,0,0,0,0,1,S,cluster0
37,F,11188,67,1,BF,0,0,0,0,0,0,0,0,0,0,0,0,0,0,0,0,0,1,0,0,0
,0,0,0,0,1,0,0,0,0,0,0,1,0,S,S,S,S,S,S,S,S,HR,LR,S,LR,LR,HR,S,HR
,LR,1,0,1,0,0,0,0,0,0,0,1,HR,cluster1
69,F,325848,232,0,BB,0,0,0,0,0,0,0,0,0,0,0,1,0,0,0,0,0,0,0,0,0
,0,0,0,0,0,0,0,0,0,0,0,0,0,S,S,S,S,S,S,S,S,S,S,S,S,S,S,S,1
,0,0,1,0,1,0,0,0,0,0,S,cluster4
42,M,18712,345,1,BF,0,0,0,0,0,1,0,0,0,0,0,1,0,0,0,1,0,0,1,0,0,0,
0,0,1,0,0,1,0,0,0,1,0,0,0,0,HR,LR,LR,LR,LR,HR,LR,S,HR,HR,HR,HR,I
R,HR,IR,S,S,1,0,1,0,0,0,0,0,0,0,1,S,cluster3
31,M,354796,168,0,BF,0,0,0,0,0,0,0,0,0,0,0,0,0,0,0,0,0,0,0,0,0
,0,0,0,0,0,0,0,0,0,0,0,0,0,S,S,S,S,S,S,S,S,S,S,S,S,S,HR,HR
,1,0,0,1,0,0,0,0,0,0,0,HR,cluster4
53,M,14499,200,1,BB,1,0,0,0,0,0,0,1,0,0,0,1,1,0,0,0,0,0,0,1,0,
0,0,1,0,0,1,0,1,0,0,1,0,0,0,IR,S,LR,IR,IR,HR,IR,IR,HR,IR,IR,IR,I
R,HR,LR,HR,HR,1,0,1,0,0,0,0,0,0,0,1,HR,cluster5
34,M,29457,210,1,BB,0,0,0,0,0,0,0,0,0,0,0,0,0,0,1,0,0,1,0,1,0,
0,0,0,0,0,1,0,0,0,1,0,0,0,0,S,S,S,S,S,S,S,S,HR,HR,HR,HR,HR,HR,IR
,HR,LR,1,0,0,1,0,0,0,0,0,0,0,HR,cluster0
46,M,5338,358,0,BB,0,0,0,0,0,1,0,0,0,0,1,0,1,0,1,1,0,1,0,0,0,0,0
,0,0,0,0,1,0,0,0,1,0,0,0,0,IR,LR,IR,LR,LR,HR,LR,S,HR,IR,IR,IR,IR
,HR,LR,HR,LR,1,0,1,0,0,0,0,0,0,0,1,HR,cluster3
46,M,28385,16,0,BB,0,0,0,1,0,0,0,0,0,1,0,0,0,0,0,0,0,0,0,0,0,0
,0,0,0,0,1,0,0,0,0,0,0,1,0,IR,S,LR,IR,LR,HR,IR,S,HR,LR,S,S,S,HR,
S,HR,LR,1,0,0,1,0,0,0,1,0,0,0,HR,cluster1
37,F,201888,30,1,F1,0,0,0,0,0,0,1,0,1,0,1,0,0,0,0,0,0,0,0,0,0,0,
0,0,0,0,0,1,0,0,0,1,0,0,0,0,IR,LR,IR,IR,LR,HR,IR,IR,HR,IR,LR,LR,
LR,HR,LR,HR,LR,1,0,1,1,0,0,0,0,0,0,0,HR,cluster3
36,F,58450,110,0,BB,0,0,0,0,0,0,0,0,0,0,0,0,0,0,1,0,0,0,0,0,0,
0,0,0,0,0,1,0,0,0,1,0,0,0,0,S,S,S,S,S,S,S,S,HR,IR,IR,IR,IR,HR,IR
,HR,LR,1,0,0,1,0,0,0,0,0,0,0,HR,cluster0

27,F,25129,112,0,BB,0,0,0,0,0,0,1,1,0,0,1,1,0,0,0,0,0,0,1,0,0,0,
0,0,1,0,0,1,0,0,0,1,1,0,0,0,LR,S,LR,IR,IR,IR,LR,LR,HR,IR,HR,HR,I
R,HR,LR,HR,IR,1,0,0,0,1,0,0,1,0,0,0,HR,cluster3
35,F,21887,133,1,BB,0,0,0,0,0,0,0,0,0,0,1,0,0,0,0,1,0,0,0,0,0,
0,0,0,0,0,1,0,0,0,0,0,0,1,0,S,S,S,S,S,S,S,S,HR,HR,S,LR,IR,HR,IR,
HR,LR,1,0,0,1,0,0,0,0,0,0,1,HR,cluster0
48,F,35448,328,0,BB,0,1,1,1,0,0,0,0,0,1,0,1,0,0,0,1,1,0,0,0,0,0,
0,0,0,0,0,1,0,0,0,1,0,0,0,0,HR,LR,HR,HR,IR,HR,IR,LR,HR,IR,IR,IR,
IR,HR,IR,S,S,1,0,1,0,0,0,0,0,0,0,1,S,cluster5
45,M,51148,318,1,CC,0,0,0,0,0,0,0,0,0,1,0,0,0,0,0,1,0,0,1,0,0,0,
0,0,0,0,0,0,0,0,0,0,0,0,0,0,LR,S,LR,IR,LR,HR,IR,S,LR,IR,HR,HR,HR
,LR,IR,S,S,1,0,0,1,0,0,0,0,0,0,1,S,cluster3
49,F,4073,102,1,F1,0,0,1,0,1,0,1,1,0,0,0,1,0,0,0,1,0,0,1,0,0,0,0
,0,1,0,0,1,1,0,0,0,0,0,0,0,IR,LR,HR,HR,HR,HR,LR,LR,HR,IR,HR,IR,I
R,HR,LR,IR,LR,1,0,0,0,0,1,0,1,0,0,1,HR,cluster2
57,F,50217,709,0,BF,0,0,1,0,1,0,1,1,0,1,0,1,0,1,1,1,0,0,0,0,0,0,
0,0,1,0,0,1,0,0,0,1,0,0,0,0,HR,LR,HR,HR,HR,HR,HR,IR,HR,IR,IR,IR,
IR,HR,LR,IR,IR,1,0,0,0,0,0,0,1,0,0,1,HR,cluster2
42,M,7597,498,1,BB,0,0,0,0,0,0,0,0,0,0,0,0,0,0,0,0,0,0,0,0,0,0,0
,0,0,0,0,1,0,0,0,0,0,0,0,0,S,S,S,S,S,S,S,S,HR,HR,S,LR,HR,HR,IR,H
R,IR,1,0,0,0,0,0,0,0,0,0,1,HR,cluster0
51,M,28810,377,1,BC,0,0,0,0,0,0,0,0,0,0,0,0,0,0,0,0,0,0,0,0,0,0,
1,0,0,0,0,1,0,0,0,0,0,0,0,0,S,S,S,S,S,S,S,S,HR,HR,S,IR,IR,HR,IR,
HR,HR,1,0,0,0,0,0,0,0,0,0,1,HR,cluster0
41,M,8593,281,0,BB,0,0,0,0,0,0,0,0,0,0,0,0,0,0,0,0,0,1,0,1,0,0
,0,0,0,0,1,1,0,0,0,0,1,1,0,S,S,S,S,S,S,S,S,HR,IR,HR,IR,IR,HR,LR,
HR,LR,1,0,0,1,0,0,0,0,0,0,0,HR,cluster0
54,F,58382,96,1,BB,0,0,0,0,0,0,1,0,1,1,1,0,0,1,0,0,0,0,0,1,1,0,0
,0,1,0,0,1,1,0,0,0,1,0,0,0,HR,LR,HR,HR,IR,HR,HR,IR,HR,IR,HR,HR,I
R,HR,LR,IR,IR,1,0,0,0,1,0,0,1,0,0,0,HR,cluster2
47,F,24164,590,0,BB,0,1,0,0,1,0,1,1,0,0,1,0,0,1,0,0,0,0,1,0,0,0,
0,0,1,0,0,1,0,0,0,1,0,0,0,0,HR,IR,HR,HR,HR,HR,IR,IR,HR,IR,IR,IR,
IR,HR,LR,HR,LR,1,0,0,0,0,0,0,1,0,0,1,HR,cluster2
38,F,23726,269,1,F1,0,0,0,1,0,0,1,1,0,0,0,0,0,0,0,0,0,0,0,0,0,0,
0,0,0,0,0,1,0,0,0,0,0,0,1,0,IR,S,IR,IR,IR,HR,IR,LR,HR,LR,S,S,S,H
R,S,HR,LR,1,0,0,1,0,0,0,1,0,0,0,HR,cluster5
51,M,51038,168,1,BB,1,0,1,0,1,1,1,1,1,0,1,0,0,1,0,1,0,0,1,0,0,0,
0,0,0,0,0,1,0,0,0,1,0,0,0,0,HR,IR,HR,HR,HR,HR,HR,IR,HR,IR,IR,IR,
IR,HR,IR,HR,LR,1,0,0,1,0,0,0,0,0,0,0,HR,cluster2
65,M,10744,335,0,F1,0,0,0,0,1,0,1,0,0,0,1,0,0,0,0,0,0,0,1,0,1,0,
0,0,0,0,0,1,0,1,0,0,1,0,0,0,HR,S,IR,HR,IR,HR,HR,IR,HR,IR,HR,IR,L
R,HR,LR,S,S,1,0,0,1,0,0,0,1,0,0,1,S,cluster5
38,F,32743,265,1,BB,0,0,1,0,1,0,1,1,0,1,0,1,0,1,0,0,0,0,1,0,1,0,
0,0,1,0,0,1,1,0,0,0,0,0,1,0,0,HR,LR,HR,HR,HR,HR,HR,IR,HR,IR,HR,HR,
HR,HR,LR,S,S,1,0,0,0,0,0,0,1,0,0,1,S,cluster2
58,M,187889,101,1,BB,0,0,0,1,0,0,1,0,0,0,1,0,0,0,0,1,0,0,0,0,0,0
,0,0,0,0,0,1,0,0,0,1,0,0,0,0,IR,S,IR,HR,IR,HR,IR,LR,HR,HR,IR,IR,
HR,HR,IR,HR,LR,1,0,0,0,0,0,0,1,0,0,1,HR,cluster2
34,M,2975,503,0,BB,0,0,0,0,0,0,0,0,0,0,1,0,1,0,0,0,0,0,1,0,1,0,0
,0,0,0,0,1,0,0,0,0,0,0,0,0,S,S,S,S,S,S,S,S,HR,LR,LR,LR,LR,HR,S,H
R,LR,1,0,0,0,0,0,0,0,0,0,1,HR,cluster0
38,F,34075,583,1,BB,0,1,1,0,0,1,0,0,1,1,0,1,0,0,0,0,0,0,1,0,1,0,
0,0,0,0,0,1,1,0,0,0,0,1,0,0,HR,IR,HR,HR,IR,HR,HR,IR,HR,IR,HR,HR,
IR,HR,LR,HR,IR,1,0,0,0,0,0,0,0,0,0,1,HR,cluster2

34,M,7667,139,1,BF,0,0,1,0,0,0,1,1,0,1,1,0,0,0,0,1,0,0,1,0,1,0,0
,0,1,0,0,1,1,0,0,0,0,1,0,0,HR,LR,HR,HR,HR,HR,HR,IR,HR,HR,HR,HR,H
R,HR,IR,S,S,1,0,0,1,0,0,0,1,0,0,1,LR,cluster2
42,M,8080,121,1,BB,1,0,1,0,1,0,0,1,0,0,1,0,0,1,0,1,0,0,0,0,1,0,0
,0,0,0,0,1,0,0,0,1,1,0,0,0,IR,LR,HR,HR,HR,HR,IR,IR,HR,HR,HR,HR,H
R,HR,HR,HR,LR,1,0,0,1,0,0,0,0,0,0,1,HR,cluster2
52,M,50865,321,0,BB,0,0,1,0,0,0,1,1,0,1,1,0,0,0,0,1,0,0,1,1,1,0,
0,0,1,0,0,1,1,0,0,0,1,0,0,0,HR,S,IR,HR,HR,HR,HR,IR,HR,IR,HR,HR,I
R,HR,IR,LR,S,1,0,0,0,0,0,0,1,0,0,1,LR,cluster2
50,M,32552,13,1,BB,0,0,0,1,0,0,1,0,1,1,1,0,0,1,1,1,0,0,1,0,1,0,0
,0,1,0,0,1,1,0,0,0,0,1,0,0,HR,LR,HR,HR,HR,HR,HR,IR,HR,HR,HR,HR,H
R,HR,IR,S,S,1,0,0,1,0,0,0,1,0,0,1,S,cluster2
57,M,142639,8,1,F1,0,0,0,0,0,0,1,1,0,1,0,1,0,1,0,0,0,0,0,0,0,1,0
,0,0,0,0,1,0,0,0,0,0,0,0,0,HR,S,IR,HR,HR,HR,HR,IR,HR,HR,S,S,HR,H
R,S,HR,HR,1,0,0,0,0,1,0,1,0,0,0,HR,cluster2
34,F,33369,355,0,BB,0,0,0,0,0,1,0,0,0,1,0,1,0,1,0,0,0,1,0,0,1,0,
0,0,1,0,0,1,0,0,0,0,0,1,0,0,HR,LR,IR,HR,IR,HR,HR,S,HR,LR,IR,IR,I
R,HR,LR,HR,LR,1,0,1,0,0,0,0,0,0,0,1,HR,cluster5
37,M,14370,334,0,BB,0,0,0,1,0,0,1,1,0,1,1,0,0,1,0,0,0,0,1,1,1,0,
0,0,0,0,0,1,1,0,0,0,0,1,0,0,HR,S,IR,HR,HR,HR,HR,LR,HR,HR,HR,HR,H
R,HR,LR,HR,HR,1,0,0,0,0,0,0,1,0,0,1,HR,cluster2
35,F,7954,554,0,BB,0,0,0,0,0,0,0,0,0,0,0,0,0,1,0,0,0,0,0,0,0,0,0
,0,0,0,0,0,0,0,0,0,0,0,0,0,S,S,S,S,S,S,S,S,IR,HR,LR,IR,IR,IR,HR,
IR,LR,1,0,0,0,0,0,0,0,1,0,1,IR,cluster0
41,M,20978,438,0,BB,0,0,0,0,0,0,0,0,0,0,0,0,0,0,0,0,0,0,0,0,0,0,
0,0,0,0,0,0,0,0,0,0,0,0,0,0,S,S,S,S,S,S,S,S,S,S,S,S,S,S,S,S,S,0,
0,0,0,0,0,0,1,0,0,1,S,cluster4
40,F,167281,271,0,BB,0,0,0,0,0,0,0,0,0,0,0,0,0,0,0,0,0,0,0,0,0,0
,0,0,0,0,0,0,0,0,0,0,0,1,0,S,S,S,S,S,S,S,S,S,S,S,S,S,S,S,HR,LR
,1,0,0,1,0,0,0,0,0,0,0,HR,cluster4
31,M,9862,153,1,BB,0,0,0,0,0,0,0,0,0,1,0,0,0,0,0,0,0,0,0,0,0,0,0
,0,0,0,0,1,0,0,0,0,0,0,0,0,LR,S,LR,IR,LR,HR,IR,S,HR,LR,S,S,S,HR,
S,S,S,1,0,0,0,0,0,0,1,0,0,1,S,cluster1
55,F,20848,224,0,BF,0,1,0,1,0,0,1,0,0,1,1,0,0,1,0,1,0,0,1,0,0,0,
0,0,0,0,0,1,0,0,0,1,0,0,0,0,HR,LR,HR,HR,IR,HR,HR,IR,HR,HR,HR,HR,
IR,HR,IR,S,S,1,0,1,0,0,0,0,0,0,0,1,S,cluster2
52,M,15739,739,0,BB,0,0,0,0,0,0,1,0,0,0,0,0,0,0,0,1,0,1,0,0,1,0,
0,0,0,0,0,1,0,0,0,1,1,0,0,0,IR,S,LR,LR,LR,HR,LR,LR,HR,IR,HR,HR,I
R,HR,IR,LR,S,1,0,1,0,0,0,0,0,0,0,1,IR,cluster3
28,M,7355,171,1,BB,0,0,0,0,0,0,0,0,0,0,0,0,0,0,0,0,0,1,0,0,0,0,0
,0,0,0,1,0,0,0,0,0,1,0,0,0,S,S,S,S,S,S,S,S,HR,LR,LR,LR,LR,HR,S,H
R,IR,1,0,0,0,0,0,0,0,0,0,1,HR,cluster0
39,M,2859,387,1,BB,1,0,0,1,0,0,1,1,0,0,0,1,0,1,0,0,0,0,0,1,1,0,0
,0,1,0,0,1,0,1,0,0,0,1,0,0,IR,LR,HR,HR,HR,HR,IR,LR,HR,LR,IR,IR,L
R,HR,LR,S,S,1,0,0,1,0,0,0,1,0,0,0,S,cluster5
65,F,195313,107,1,F1,0,0,0,0,0,0,0,0,0,0,0,0,0,0,0,0,0,0,0,0,0,0,0
,0,0,0,0,0,0,0,0,0,0,0,0,0,S,S,S,S,S,S,S,S,S,S,S,S,S,S,S,HR,LR
,1,0,1,0,0,0,0,0,0,0,1,HR,cluster4
42,F,11525,38,0,BF,0,0,1,0,0,0,0,0,0,0,0,1,0,0,0,1,0,0,0,0,0,0,0
,0,1,0,0,1,0,0,1,1,0,0,0,0,IR,S,LR,S,S,HR,LR,LR,HR,HR,HR,HR,HR,H
R,IR,S,S,1,0,0,0,0,0,0,1,0,0,1,S,cluster3
41,M,8949,559,1,BF,0,0,1,0,1,0,1,1,0,1,0,1,0,1,0,1,0,0,1,0,0,0,0
,0,1,0,0,1,0,0,0,1,0,0,0,0,HR,LR,HR,HR,HR,HR,HR,IR,HR,HR,HR,HR,H
R,HR,IR,S,S,1,0,0,0,0,0,0,1,0,0,1,S,cluster2

33,F,56527,38,0,BF,0,0,0,1,0,0,0,0,0,0,0,0,0,0,0,0,0,0,0,0,0,0
,0,0,0,0,1,0,0,0,0,0,0,0,0,LR,S,LR,LR,LR,IR,S,S,HR,LR,S,S,S,HR,S
,S,S,1,1,0,0,0,0,0,0,0,1,1,S,cluster1
30,M,48165,65,0,BF,0,0,0,0,0,0,0,0,0,0,0,0,0,0,0,0,0,1,0,1,0,0
,0,0,0,0,1,0,1,0,0,0,1,0,0,S,S,S,S,S,S,S,S,HR,IR,HR,IR,LR,HR,LR,
HR,LR,1,0,0,1,0,0,0,0,1,0,0,HR,cluster0
36,F,2494,236,0,BB,0,0,0,0,0,0,0,0,0,0,0,1,0,0,0,1,0,0,1,0,1,0,0
,0,0,0,0,1,1,0,0,1,0,1,0,0,S,S,S,S,S,S,S,S,HR,HR,HR,HR,HR,HR,IR,
HR,LR,1,0,0,1,0,0,0,0,1,0,0,HR,cluster0
41,F,172000,192,0,BB,1,0,1,0,1,0,1,1,1,0,0,1,0,1,0,1,0,0,1,0,1,0
,0,0,0,0,0,1,1,0,0,0,0,1,0,0,HR,LR,HR,HR,HR,HR,HR,IR,HR,IR,HR,HR
,HR,HR,IR,S,S,1,0,0,0,0,0,0,0,0,0,1,S,cluster2
40,M,6901,373,0,BB,0,0,0,0,0,0,0,0,0,0,0,0,0,0,0,0,0,0,0,0,0,0,0
,0,0,0,0,1,0,0,0,1,0,0,0,0,S,S,S,S,S,S,S,S,HR,IR,LR,LR,LR,HR,LR,
HR,LR,1,0,0,1,0,0,0,0,0,0,0,HR,cluster0
33,M,72894,211,1,BB,0,0,0,0,0,0,0,0,0,0,0,0,1,0,0,1,0,0,1,0,1,0,
0,0,0,0,0,1,0,0,0,1,1,0,0,0,HR,S,S,S,S,S,S,LR,HR,IR,HR,HR,IR,HR,
IR,HR,IR,1,0,0,1,0,0,0,0,0,0,0,HR,cluster0
38,F,131190,44,1,BB,0,0,0,0,0,0,0,0,0,0,1,0,0,0,0,1,0,0,0,0,0,0,
0,0,0,0,0,1,0,0,0,1,0,0,0,0,S,S,S,S,S,S,LR,S,HR,HR,IR,IR,HR,HR,I
R,HR,LR,1,0,1,0,0,0,0,0,0,0,1,HR,cluster0
37,F,26478,93,1,BB,0,0,0,0,0,0,0,0,0,0,0,1,0,0,0,0,0,0,0,0,0,0,0
,0,0,0,0,1,0,0,0,0,0,0,0,0,S,S,S,S,S,S,S,S,HR,LR,S,S,S,HR,S,S,S,
1,1,0,0,0,0,0,0,0,0,1,S,cluster1
53,F,100349,158,1,BB,0,0,0,0,0,0,0,0,0,0,0,0,0,0,0,0,0,0,1,0,1,0
,0,0,0,0,0,1,0,1,0,0,1,0,0,0,LR,S,LR,LR,LR,LR,LR,HR,HR,IR,HR,IR,
LR,HR,LR,S,S,1,0,0,0,1,0,0,0,0,0,1,S,cluster3
48,M,28005,128,1,BF,0,0,0,0,0,0,0,0,0,0,1,0,0,0,0,0,0,0,0,0,0,
0,0,0,0,0,0,0,0,0,0,0,0,0,0,S,S,S,S,S,S,S,S,S,S,S,S,S,S,S,S,1,
0,0,0,0,0,0,1,0,0,1,S,cluster4
44,F,305459,227,1,BB,0,0,1,1,0,0,1,0,0,1,0,1,1,0,0,1,0,0,1,1,1,0
,0,0,1,0,0,1,1,0,0,0,1,0,0,0,HR,LR,HR,HR,IR,HR,HR,IR,HR,HR,HR,HR
,HR,HR,IR,HR,LR,1,0,0,0,0,1,0,1,0,0,1,HR,cluster2
35,M,9919,571,0,BF,0,0,0,0,0,0,0,0,0,0,0,0,0,0,0,0,0,0,0,0,1,0
,0,0,0,0,1,0,0,0,0,0,0,0,0,S,S,S,S,S,S,S,HR,HR,S,S,HR,HR,S,HR,
HR,1,0,0,0,0,0,0,0,0,0,1,HR,cluster1
33,M,2250,413,1,BB,0,0,0,0,0,0,0,0,0,0,0,1,0,0,0,0,0,0,0,0,0,0,0
,0,0,0,0,1,0,0,0,0,0,0,0,0,S,S,S,S,S,S,S,S,HR,LR,S,S,S,HR,S,HR,L
R,1,0,0,1,0,0,0,0,0,0,0,HR,cluster1
40,F,6898,196,0,BB,0,0,0,0,0,0,0,0,0,0,0,0,0,0,0,0,0,0,0,0,0,0,0
,0,0,0,0,1,0,0,0,0,0,0,0,0,S,S,S,S,S,S,S,S,HR,LR,S,S,S,HR,S,HR,L
R,1,0,0,1,0,0,0,1,0,0,1,HR,cluster1
39,M,31966,296,1,BB,0,0,0,0,1,0,1,0,0,1,0,0,0,1,0,1,0,0,0,0,0,0,
0,0,0,0,0,1,0,0,0,1,0,0,0,0,IR,S,IR,HR,IR,HR,HR,LR,HR,IR,HR,HR,I
R,HR,IR,S,S,0,0,1,1,0,0,0,0,0,0,0,S,cluster5
48,M,22987,214,1,BB,0,1,1,0,0,0,0,0,1,0,0,0,1,0,0,1,0,0,1,0,0,0,
0,0,1,0,0,0,0,0,0,1,0,0,0,0,HR,IR,HR,HR,IR,HR,IR,HR,LR,IR,HR,HR,
IR,LR,IR,S,S,0,0,0,1,0,1,0,1,0,0,0,S,cluster2
39,F,7886,129,0,BF,0,0,0,0,0,0,0,0,0,0,0,1,0,0,0,0,0,0,0,0,0,0,0
,0,0,0,0,1,0,0,0,0,0,0,1,0,S,S,S,S,S,S,S,S,HR,LR,S,S,S,HR,S,HR,L
R,1,1,0,1,0,0,0,0,0,0,0,HR,cluster1
76,F,20546,85,1,BB,0,0,0,0,0,0,0,0,0,0,0,0,1,0,0,0,0,0,1,1,1,0,0
,0,0,0,0,1,1,1,1,0,1,0,0,0,S,S,S,S,S,S,S,S,HR,IR,HR,HR,IR,HR,LR,
S,S,1,1,0,1,0,0,0,0,0,0,0,S,cluster0

44,M,23835,95,0,BF,0,0,0,0,0,0,0,0,0,0,0,0,0,0,0,0,0,0,0,0,0
,0,0,0,0,0,0,0,0,0,0,0,0,0,S,S,S,S,S,S,S,S,S,S,S,S,S,S,S,1,0
,1,0,0,0,0,0,0,0,1,S,cluster4
42,M,51455,320,1,BF,0,0,0,0,0,0,0,0,0,1,0,0,1,0,1,1,0,0,0,0,0,0,
0,0,0,0,0,1,0,0,0,1,0,1,0,0,IR,S,LR,IR,LR,HR,IR,S,HR,HR,HR,HR,HR
,HR,IR,S,S,1,0,1,0,0,0,0,0,0,0,1,S,cluster3
36,M,7331,258,1,BB,1,0,0,1,0,0,1,1,0,0,1,0,0,0,1,0,0,0,1,1,1,0,0
,0,1,0,0,1,1,0,0,0,0,1,0,0,IR,S,IR,HR,IR,HR,IR,IR,HR,IR,HR,HR,IR
,HR,LR,S,S,1,0,0,1,0,0,0,1,0,0,0,LR,cluster5
37,F,3922,523,0,BF,0,0,0,0,0,0,0,0,0,0,0,0,0,0,0,0,0,0,0,0,0,0,0
,0,0,0,0,1,0,0,0,0,0,0,0,0,S,S,S,S,S,S,S,S,HR,LR,S,S,S,HR,S,HR,L
R,1,0,0,1,0,0,0,0,1,0,0,HR,cluster1
39,F,59414,88,0,BF,0,0,0,0,0,0,0,0,0,0,0,1,0,0,0,0,0,0,0,0,0,0,0
,0,0,0,0,0,0,0,0,0,0,0,0,0,S,S,S,S,S,S,S,S,S,S,S,S,S,S,S,HR,IR,1
,0,0,1,0,0,0,0,1,0,0,HR,cluster4
36,F,2068,314,0,F1,0,0,0,0,0,0,1,0,0,1,0,1,0,1,0,0,0,1,0,0,0,0,0
,0,0,0,0,1,0,0,0,0,0,0,0,0,IR,S,IR,IR,LR,HR,HR,LR,HR,IR,LR,LR,IR
,HR,LR,S,S,1,0,1,0,0,0,0,0,0,0,1,S,cluster5
58,M,316620,15,1,BB,0,0,0,0,0,1,0,0,0,1,1,0,0,1,0,1,0,0,1,1,1,0,
0,0,0,0,0,1,0,0,0,1,1,0,0,0,IR,LR,IR,IR,LR,HR,IR,S,HR,HR,HR,HR,H
R,HR,IR,S,S,1,1,0,1,0,0,0,0,0,0,0,S,cluster3
42,M,4308,106,1,BB,0,0,0,0,0,0,0,0,0,0,1,1,0,0,0,0,0,0,0,0,0,0,0
,0,0,0,0,0,0,0,0,0,0,0,0,0,S,S,S,S,S,S,S,S,S,S,S,S,S,S,S,S,1,0
,1,0,0,0,0,0,0,0,1,S,cluster4
47,M,5250,412,0,BB,1,0,0,1,0,1,0,0,0,0,0,0,0,0,0,1,0,0,0,0,0,0,0
,0,0,0,0,1,0,0,0,1,0,0,0,0,IR,IR,HR,HR,HR,HR,LR,S,HR,HR,IR,IR,HR
,HR,IR,HR,IR,1,0,0,0,0,0,0,0,0,0,1,HR,cluster2
33,F,7623,160,0,BB,0,0,0,0,0,0,0,0,0,0,0,0,0,1,0,0,0,0,0,0,0,0,0
,0,0,0,0,1,0,0,0,0,0,0,1,0,S,S,S,S,S,S,S,S,HR,LR,S,S,S,HR,S,HR,L
R,1,0,0,0,0,0,0,1,0,0,0,HR,cluster1
47,M,58423,463,1,BB,0,0,0,0,0,0,0,0,0,0,0,0,0,0,0,0,0,0,0,0,0,0,
0,0,0,0,0,0,0,0,0,0,0,0,0,0,S,S,S,S,S,S,S,S,S,S,S,S,S,S,S,S,1,
0,0,0,0,1,0,1,0,0,1,S,cluster4
39,F,26610,7,?,BF,0,0,0,1,0,0,1,0,1,0,1,0,0,1,0,0,0,0,1,0,1,0,0,
0,0,0,0,1,0,0,0,1,1,0,0,0,HR,IR,HR,HR,HR,HR,IR,IR,HR,IR,HR,IR,IR
,HR,LR,HR,IR,1,0,0,0,0,0,0,1,1,0,1,HR,cluster2
42,F,12404,220,0,BB,0,0,0,0,0,0,0,0,0,0,0,0,0,0,0,0,0,0,0,0,0,0,
0,0,0,0,0,1,0,0,0,0,0,0,0,0,S,S,S,S,S,S,S,S,HR,LR,S,S,S,HR,S,HR,
LR,1,0,0,1,0,0,0,0,1,0,0,HR,cluster1
39,M,6216,125,1,BF,0,0,0,0,0,0,0,0,0,0,0,0,0,0,0,1,0,0,0,0,0,0,0
,0,0,0,0,1,1,0,0,1,0,0,1,0,S,S,S,S,S,S,S,S,HR,IR,IR,IR,IR,HR,LR,
HR,LR,1,0,0,1,0,0,0,0,0,0,0,HR,cluster0
43,M,112936,452,0,BF,0,1,0,1,0,1,1,0,0,0,0,1,0,1,1,0,0,0,1,1,1,0
,0,0,1,0,0,1,1,0,0,0,1,0,0,0,HR,IR,HR,IR,IR,HR,IR,IR,HR,IR,HR,HR
,IR,HR,LR,HR,LR,1,0,0,1,0,0,0,1,0,0,0,HR,cluster2
41,M,98327,13,1,BB,0,0,0,0,0,0,0,0,0,0,0,0,0,0,0,0,0,0,0,0,0,0,0
,0,0,0,0,1,0,0,0,0,0,0,0,0,S,S,S,S,S,S,S,S,HR,IR,S,LR,IR,HR,LR,H
R,LR,1,0,0,0,0,0,0,0,0,0,1,HR,cluster0
37,F,19658,239,0,BB,0,0,0,0,0,0,0,0,0,0,0,0,1,0,0,0,0,0,0,0,0,0,
0,0,0,0,0,0,0,0,0,0,0,0,0,0,S,S,S,S,S,S,S,S,S,S,S,S,S,S,S,S,1,
1,0,1,0,0,0,0,0,0,0,S,cluster4
36,F,47754,99,1,BB,0,0,1,0,0,0,1,1,0,1,1,0,1,0,0,0,1,0,0,0,0,0,1
,0,0,0,0,1,0,0,0,0,0,0,0,0,HR,S,IR,HR,IR,HR,HR,IR,HR,LR,S,S,LR,H
R,S,S,S,1,0,0,1,0,0,0,1,0,0,1,S,cluster5

44,M,3359,278,0,BB,0,0,0,0,0,0,0,0,0,0,0,1,0,0,0,0,0,0,0,0,0,0,0
,0,0,0,0,1,0,0,0,1,0,0,0,0,S,S,S,S,S,S,S,S,HR,IR,LR,LR,LR,HR,LR,
HR,IR,1,0,0,1,0,0,0,0,0,0,0,HR,cluster0
41,F,34217,89,1,BB,0,0,0,0,0,0,0,0,0,0,0,0,0,0,0,0,0,0,0,0,0,0,0
,0,0,0,0,0,0,0,0,0,0,0,0,0,S,S,S,S,S,S,S,S,S,S,S,S,S,S,S,S,S,1,0
,0,1,0,0,0,1,0,0,0,S,cluster4
31,M,222200,69,0,BB,0,1,0,0,0,0,0,0,0,0,1,0,0,0,0,0,0,0,0,0,1,0,
0,0,1,0,0,1,0,0,0,0,0,1,1,0,LR,LR,HR,LR,HR,IR,LR,LR,HR,IR,IR,IR,
HR,HR,LR,HR,LR,1,0,0,0,0,0,0,1,0,0,1,HR,cluster3
32,F,3874,510,0,CC,0,0,0,0,0,0,0,0,0,0,0,0,0,0,0,0,0,0,0,0,0,0,0
,0,0,0,0,1,0,0,0,0,0,0,0,0,S,S,S,S,S,S,S,S,HR,LR,S,S,S,HR,S,HR,L
R,1,0,0,1,0,0,0,0,1,0,0,HR,cluster1
27,M,8844,138,1,BB,0,1,0,0,0,0,0,0,0,0,0,0,0,1,0,0,0,0,0,0,0,0,0
,0,0,0,0,1,0,0,0,0,0,0,0,0,HR,LR,IR,LR,LR,HR,LR,S,HR,LR,S,S,S,HR
,S,LR,LR,1,0,1,1,0,0,0,0,0,0,0,IR,cluster1
32,M,5337,649,0,BB,0,0,1,0,1,0,1,1,0,1,0,1,1,0,0,1,0,0,0,0,0,1,0
,0,0,0,0,1,0,0,0,1,0,0,0,0,HR,LR,HR,HR,HR,HR,HR,HR,HR,HR,IR,IR,H
R,HR,IR,HR,IR,1,0,0,1,0,0,0,1,0,0,0,HR,cluster2
30,F,237127,241,0,BB,0,0,0,0,0,0,0,0,0,0,0,0,0,0,0,0,0,0,0,1,0,0
,0,0,1,0,0,0,0,0,0,0,0,0,0,0,S,S,S,S,S,S,S,S,LR,S,S,LR,LR,LR,S,H
R,LR,1,0,1,1,0,0,0,0,0,0,1,HR,cluster4
35,F,27328,264,1,BB,0,0,0,0,0,1,0,0,0,0,0,1,0,0,0,1,0,0,1,0,0,0,
0,0,1,0,0,0,0,0,0,1,0,0,0,0,LR,LR,IR,LR,LR,LR,LR,S,LR,IR,HR,HR,I
R,LR,IR,HR,IR,1,0,0,0,0,0,0,0,0,0,1,HR,cluster3
47,F,22713,41,1,F1,0,0,0,0,0,0,0,0,0,0,0,1,0,0,0,0,1,0,0,0,0,0,0,0
,0,0,0,0,1,0,0,0,1,0,0,0,0,S,S,S,S,S,S,S,S,HR,IR,IR,IR,IR,HR,IR,
S,S,1,0,0,1,0,0,0,0,0,0,0,LR,cluster0
46,F,12827,178,0,BB,0,0,0,0,0,0,0,0,0,0,1,0,1,0,0,0,1,0,0,0,0,0,
0,0,0,0,0,1,0,0,0,0,0,0,0,0,S,S,S,S,S,S,S,S,HR,HR,S,LR,IR,HR,IR,
S,S,1,1,0,0,0,0,0,0,0,0,1,S,cluster0
43,M,49800,113,1,BB,0,0,0,0,0,0,0,0,0,0,0,0,0,0,0,0,0,0,0,0,0,1,
0,0,0,0,0,1,0,0,0,0,0,0,0,0,S,S,S,S,S,S,S,S,HR,HR,S,S,HR,HR,S,HR
,HR,1,0,0,0,0,0,0,0,0,0,1,HR,cluster1
42,M,2495,134,1,F1,0,0,0,1,0,0,1,1,0,0,0,1,0,0,1,1,0,0,1,0,1,0,0
,0,0,0,0,1,1,0,0,0,0,1,1,0,IR,LR,HR,HR,HR,HR,IR,IR,HR,IR,HR,HR,H
R,HR,IR,HR,LR,1,0,0,1,0,0,0,1,0,0,0,HR,cluster2
37,M,139249,204,1,BB,0,1,0,0,0,0,0,0,0,0,0,1,0,1,0,0,1,0,0,0,0,0
,0,0,0,0,0,1,0,0,0,0,0,0,0,0,LR,LR,IR,LR,LR,LR,S,S,HR,LR,S,S,S,H
R,S,S,S,1,1,0,1,0,0,0,0,0,0,0,LR,cluster1
46,F,20864,341,1,BB,0,0,0,1,0,0,1,1,0,0,1,0,1,0,0,0,1,0,0,0,0,0,
0,0,1,0,0,1,0,0,0,0,0,0,0,0,IR,LR,HR,HR,HR,HR,IR,IR,HR,LR,LR,LR,
LR,HR,S,S,S,1,0,0,0,0,0,0,0,0,0,0,S,cluster5
33,F,57876,416,1,BB,0,0,0,0,0,0,0,0,0,0,0,1,0,0,0,0,0,0,0,0,0,0,
0,0,0,0,0,0,0,0,0,0,0,0,0,0,S,S,S,S,S,S,S,S,S,S,S,S,S,S,S,S,S,1,
0,0,0,0,0,0,1,0,0,1,S,cluster4
32,M,57980,224,1,BB,0,0,0,1,0,0,0,0,0,1,1,0,0,0,0,1,0,0,1,0,0,0,
0,0,1,0,0,0,0,0,0,1,0,0,0,0,IR,S,IR,IR,LR,HR,IR,S,LR,HR,HR,HR,HR
,LR,IR,S,S,0,0,1,1,0,1,0,0,0,0,0,S,cluster3
65,M,1374,234,0,BB,0,0,0,0,1,0,1,1,0,0,0,1,0,0,0,0,0,0,0,0,0,0,0
,0,0,0,0,1,0,0,0,0,0,0,0,0,IR,S,IR,HR,IR,HR,IR,LR,HR,LR,S,S,S,HR
,S,S,S,1,0,0,1,0,0,0,0,0,1,0,S,cluster5
39,F,421898,188,1,BB,0,0,0,0,0,0,0,0,0,0,0,0,0,0,0,0,0,0,0,0,0,0,0
,0,0,0,0,0,1,1,0,0,0,0,0,0,0,S,S,S,S,S,S,S,S,HR,IR,LR,LR,LR,HR,S
,HR,LR,1,0,0,1,0,0,0,0,0,0,0,HR,cluster1

45,M,26115,18,1,BB,0,0,0,0,0,0,0,0,0,0,0,0,0,0,0,0,0,0,0,0,0,0
,0,0,0,0,0,0,0,0,0,0,0,0,0,0,S,S,S,S,S,S,S,S,S,S,S,S,S,S,S,S,1,0
,0,0,0,0,0,0,1,0,0,1,S,cluster4
41,M,14173,295,0,BB,0,0,0,0,0,0,0,0,0,0,1,0,0,0,0,1,0,0,1,0,1,0,
0,0,0,0,0,1,0,0,0,1,1,0,0,0,S,S,S,S,S,S,S,S,HR,IR,HR,HR,IR,HR,IR
,HR,LR,1,0,0,1,0,0,0,0,0,0,0,HR,cluster0
49,M,370416,122,1,BB,0,0,0,0,0,0,0,0,0,0,0,1,0,0,0,0,0,0,0,0,0,0
,0,0,0,0,0,0,0,0,0,0,0,0,1,0,S,S,S,S,S,S,S,S,S,S,S,S,S,S,S,HR,LR
,1,0,0,1,0,0,0,0,0,0,0,HR,cluster4
43,M,3393,142,1,BB,0,0,0,0,0,0,0,0,0,0,0,0,0,0,0,0,0,0,0,0,0,0,0
,0,0,0,0,1,0,0,0,0,0,0,0,1,S,S,S,S,S,S,S,S,HR,LR,S,S,S,HR,S,HR,I
R,1,0,0,1,0,0,0,0,0,0,0,HR,cluster1
39,M,4668,253,0,BB,0,0,1,1,0,0,1,1,0,0,0,0,0,0,0,0,0,0,0,0,0,0,0
,0,0,0,0,1,0,0,0,0,0,0,0,0,IR,LR,HR,HR,HR,HR,LR,LR,HR,LR,S,S,S,H
R,S,LR,LR,1,0,0,0,0,0,0,1,0,0,1,LR,cluster5
57,F,3330,361,1,BB,0,0,0,0,0,0,0,0,0,0,0,0,0,0,0,0,0,0,0,0,0,0,0
,0,0,0,0,1,0,0,1,1,0,0,0,0,S,S,S,S,S,S,S,S,HR,IR,LR,LR,LR,HR,S,H
R,IR,1,0,0,0,1,0,0,0,0,0,0,HR,cluster0
49,M,6516,176,1,BB,0,0,0,0,0,0,0,0,0,1,0,0,0,1,0,0,0,1,0,0,1,1,0
,0,0,0,0,1,1,0,0,0,1,0,0,0,IR,S,LR,IR,LR,HR,IR,S,HR,HR,HR,IR,HR,
HR,LR,HR,HR,1,0,0,0,0,0,0,0,0,0,1,HR,cluster3
41,M,21392,419,1,BB,0,0,0,0,0,0,0,0,1,0,1,0,0,1,0,1,0,0,1,0,0,0,
0,0,1,0,0,1,0,0,0,1,0,0,0,0,IR,LR,IR,IR,LR,IR,IR,IR,HR,HR,HR,HR,
HR,HR,IR,HR,IR,1,0,1,0,0,0,0,0,0,0,1,HR,cluster3
44,F,95520,4,1,BB,0,1,1,0,0,1,0,0,1,1,0,0,0,1,0,0,1,0,0,1,0,0,0,
1,1,1,0,1,0,0,0,0,0,0,1,0,HR,IR,HR,HR,HR,HR,HR,IR,HR,HR,HR,HR,HR
,HR,IR,HR,IR,1,0,0,0,0,0,0,0,0,0,1,HR,cluster2
58,F,9772,208,0,BB,0,0,0,0,0,0,1,0,0,0,0,0,0,1,0,1,0,0,0,0,0,0,0
,0,1,0,0,1,1,0,0,0,0,0,0,0,IR,LR,IR,IR,IR,HR,IR,IR,HR,HR,HR,HR,H
R,HR,IR,HR,IR,1,0,0,0,0,0,0,1,0,0,1,HR,cluster2
47,M,2641,716,0,BD,0,0,0,1,1,0,1,0,0,0,0,1,0,1,1,0,0,0,1,0,1,0,0
,0,0,0,0,1,1,0,0,0,0,1,0,0,IR,S,IR,HR,IR,HR,IR,LR,HR,IR,HR,HR,IR
,HR,LR,S,S,1,0,0,1,0,0,0,1,0,0,0,S,cluster5
36,M,61257,275,1,BB,0,0,0,0,0,0,0,0,0,0,0,0,0,0,0,0,0,0,0,0,0,0,
0,0,0,0,0,1,0,0,1,1,0,0,0,0,S,S,S,S,S,S,S,S,HR,IR,LR,LR,LR,HR,LR
,S,S,1,0,1,1,0,0,0,0,0,0,0,S,cluster0
33,F,12208,362,1,BB,0,0,0,0,0,0,0,0,0,0,0,0,0,0,0,0,0,0,0,0,0,0,
0,0,0,0,0,1,0,0,0,0,0,0,0,0,S,S,S,S,S,S,S,S,HR,LR,S,S,S,HR,S,S,S
,1,1,0,1,0,0,0,0,0,0,0,S,cluster1
44,M,90340,198,1,BB,0,0,0,0,0,0,0,0,0,0,0,0,0,0,0,0,0,0,0,0,0,0,
0,0,0,0,0,1,0,0,0,0,0,0,1,0,S,S,S,S,S,S,S,S,HR,LR,S,LR,LR,HR,S,H
R,LR,1,0,0,1,0,0,0,0,0,0,0,HR,cluster1
46,M,10111,157,0,BB,0,0,0,0,0,0,0,0,0,0,0,1,0,0,0,0,0,0,1,0,1,0,
0,0,0,0,0,1,0,1,0,0,1,1,0,0,S,S,S,S,S,S,S,S,HR,IR,HR,IR,LR,HR,LR
,HR,LR,1,0,0,1,0,0,0,0,0,0,0,HR,cluster0
45,M,2854,67,1,F1,0,0,1,0,0,0,0,1,0,1,0,0,0,1,0,1,0,0,0,0,0,1,0,
0,0,0,0,1,0,0,0,1,0,0,0,1,HR,S,IR,HR,IR,HR,IR,LR,HR,HR,IR,IR,HR,
HR,LR,HR,IR,1,0,0,0,0,0,0,1,0,0,1,HR,cluster2
52,F,9781,162,0,BB,0,1,0,1,0,0,0,1,0,0,1,0,1,0,0,1,1,0,0,0,0,0,0
,0,1,0,0,1,0,0,0,1,0,0,0,0,IR,LR,IR,HR,IR,HR,LR,LR,HR,HR,HR,HR,H
R,HR,IR,HR,LR,1,0,0,0,1,0,0,0,0,0,0,HR,cluster2
29,F,5482,174,1,BB,0,0,0,0,0,0,0,0,0,0,0,0,0,0,0,0,0,0,0,0,0,0,0
,0,0,0,0,1,0,0,0,0,0,0,0,0,S,S,S,S,S,S,S,S,HR,LR,S,S,S,HR,S,HR,L
R,1,0,1,0,0,0,0,0,0,0,1,HR,cluster1

34,M,3087,591,0,BB,0,0,0,0,0,0,0,0,0,0,0,0,1,0,0,1,0,0,1,0,0,0,0
,0,0,0,0,0,1,0,0,0,0,1,0,0,S,S,S,S,S,S,S,S,S,IR,HR,HR,IR,S,LR,S,
S,1,0,0,0,0,0,0,1,0,0,1,S,cluster0
38,F,9924,53,1,BB,0,0,0,0,0,0,0,0,0,1,1,0,0,0,0,0,0,0,1,0,1,0,0,
0,0,0,0,1,0,0,0,1,0,1,0,0,IR,S,LR,IR,LR,HR,HR,S,HR,IR,HR,IR,IR,H
R,LR,IR,IR,1,0,1,0,0,0,0,0,0,0,1,HR,cluster3
50,F,71632,340,1,BB,1,0,0,1,0,0,0,1,0,0,1,0,0,1,0,0,1,0,0,0,0,0,
0,0,0,0,0,1,0,0,0,0,0,1,0,0,HR,S,IR,IR,IR,HR,IR,S,HR,HR,S,LR,IR,
HR,IR,S,S,1,0,1,0,0,0,0,0,0,0,1,S,cluster5
38,M,11962,695,0,BB,0,0,0,0,0,0,1,1,0,0,0,1,0,1,1,1,0,0,0,0,0,0,
0,0,0,0,0,1,0,0,0,1,0,0,0,0,IR,S,LR,IR,IR,HR,LR,LR,HR,IR,IR,IR,I
R,HR,LR,S,S,1,0,0,0,1,0,0,0,0,0,0,S,cluster5
53,M,82737,453,0,F1,0,0,0,0,0,0,0,0,0,0,0,0,0,0,0,1,0,1,0,0,1,0,
0,0,1,0,0,1,0,0,0,0,1,0,0,0,S,S,S,S,S,S,S,S,HR,IR,HR,HR,IR,HR,LR
,HR,LR,1,0,0,1,0,0,0,0,0,0,0,HR,cluster0
63,M,8188,353,0,BB,0,0,0,0,0,0,0,0,0,0,0,0,0,1,0,0,0,0,1,0,1,0,0
,0,0,0,0,1,0,0,0,0,0,1,0,0,S,S,S,S,S,S,S,S,HR,IR,IR,LR,IR,HR,S,I
R,LR,1,0,0,0,0,0,0,0,1,0,1,HR,cluster0
45,M,7962,170,0,BB,0,0,0,0,0,0,0,0,0,0,0,1,0,0,0,1,0,0,0,0,0,0,0
,0,0,0,0,1,1,0,0,0,0,0,0,0,S,S,S,S,S,S,S,S,HR,HR,HR,HR,HR,HR,HR,
HR,LR,1,0,0,1,0,0,0,0,0,0,0,HR,cluster0
55,M,3401,135,1,BB,0,0,0,0,0,0,0,0,0,0,0,0,0,0,0,0,0,0,0,0,0,0,0
,0,0,0,0,0,0,0,0,0,0,0,0,0,S,S,S,S,S,S,S,S,S,S,S,S,S,S,S,S,S,0,0
,0,0,1,1,0,1,0,0,0,S,cluster4
48,M,231791,510,1,BB,0,0,1,0,1,0,0,1,0,0,0,0,0,0,0,0,0,0,1,0,1,0
,0,0,0,0,0,1,1,0,0,0,0,0,1,1,HR,S,LR,IR,IR,HR,LR,LR,HR,IR,HR,IR,
IR,HR,LR,HR,IR,1,0,1,0,0,0,0,0,0,0,1,HR,cluster3
48,M,3524,393,0,BF,0,0,0,0,0,0,0,0,0,0,0,1,0,0,0,0,0,0,0,0,0,0,0
,0,0,0,0,1,1,0,0,0,0,0,0,1,S,S,S,S,S,S,S,S,HR,IR,LR,LR,LR,HR,S,H
R,IR,1,0,0,1,0,0,0,0,0,0,0,HR,cluster0
47,F,18474,736,0,BF,0,0,1,0,0,0,1,1,0,1,1,0,0,0,0,0,0,0,1,0,1,0,
0,0,0,0,0,1,0,0,0,0,1,0,0,0,IR,S,IR,HR,IR,HR,HR,IR,HR,LR,IR,LR,L
R,HR,S,S,S,1,0,0,0,0,0,0,0,0,0,1,S,cluster5
48,M,14002,54,0,BF,0,0,0,0,0,0,0,0,0,0,0,0,0,0,0,0,0,0,0,0,0,0,0
,0,0,0,0,1,0,0,0,1,0,0,0,0,S,S,S,S,S,S,S,S,HR,IR,LR,LR,LR,HR,LR,
HR,LR,1,0,0,1,0,0,0,0,0,0,0,HR,cluster0
56,M,17484,75,1,BB,0,0,0,0,0,0,1,0,1,1,0,0,0,1,0,1,0,0,1,0,1,0,0
,0,1,0,0,1,1,0,0,0,0,1,0,0,HR,LR,HR,HR,IR,HR,HR,IR,HR,HR,HR,HR,H
R,HR,IR,HR,LR,1,0,0,0,0,0,0,0,0,0,1,HR,cluster2
48,F,97393,99,1,BB,0,0,1,1,0,0,0,1,0,0,0,0,1,0,0,1,0,0,0,1,0,0,0
,0,0,0,0,1,1,0,0,0,0,0,0,0,HR,S,IR,IR,IR,HR,LR,IR,HR,HR,HR,HR,HR
,HR,IR,S,S,1,0,0,0,0,0,0,1,0,0,1,LR,cluster2
56,M,23442,266,1,BB,0,0,1,0,0,0,1,1,1,1,1,0,0,1,0,1,0,0,1,0,0,0,
0,0,1,0,0,1,0,0,0,1,1,0,1,0,HR,LR,HR,HR,HR,HR,HR,IR,HR,HR,HR,HR,
HR,HR,IR,HR,LR,1,0,0,0,0,0,0,1,0,0,1,HR,cluster2
29,F,119121,192,1,BB,0,0,0,0,0,0,0,0,0,0,0,1,0,0,0,0,0,0,0,0,0,0,0
,0,0,0,0,0,1,0,0,0,0,0,0,0,0,S,S,S,S,S,S,S,S,HR,LR,S,S,S,HR,S,S,
S,1,0,0,0,1,0,0,1,0,0,0,S,cluster1
62,M,6553,426,0,BB,1,0,0,1,0,0,1,0,0,0,1,1,0,1,1,0,0,0,1,0,1,0,0
,0,0,0,0,1,1,0,0,0,0,1,0,0,IR,S,IR,HR,IR,HR,IR,IR,HR,IR,HR,IR,IR
,HR,LR,S,S,1,0,0,0,0,0,0,1,0,0,1,S,cluster5
59,M,12440,186,0,BB,0,0,0,0,0,0,0,0,0,0,0,1,0,0,0,0,0,0,1,0,1,0,
0,0,0,0,0,0,1,0,0,0,0,1,0,0,S,S,S,S,S,S,S,S,S,LR,HR,HR,LR,S,LR,S
,S,0,0,0,1,0,1,0,0,0,0,0,S,cluster0

38,M,19376,63,1,BF,1,0,0,0,1,0,1,1,0,0,1,0,0,1,0,1,0,0,1,0,0,0,0
,0,1,0,0,1,0,0,0,1,0,0,0,0,HR,LR,IR,HR,IR,HR,IR,LR,HR,HR,HR,HR,H
R,HR,IR,IR,LR,1,0,1,0,0,0,0,0,0,0,1,HR,cluster2
61,F,17261,689,?,BB,0,0,0,0,0,0,0,0,0,0,0,0,0,0,1,0,0,0,0,0,0,
0,0,1,0,0,1,0,0,0,1,0,0,0,0,S,S,S,S,S,S,S,S,HR,HR,HR,HR,IR,HR,IR
,HR,LR,1,0,0,0,1,0,0,0,0,0,0,HR,cluster0
46,M,2577,103,1,BB,0,0,0,0,0,0,0,0,0,0,0,0,0,0,0,0,0,0,0,0,0,0,0
,0,0,0,0,1,0,0,0,0,0,0,1,0,S,S,S,S,S,S,S,S,HR,LR,S,S,S,HR,S,HR,L
R,1,0,0,1,0,0,0,0,0,0,0,HR,cluster1
41,M,3802,205,0,BB,0,0,0,0,0,0,0,0,0,0,0,0,0,0,0,0,0,0,0,0,0,0,0
,0,0,0,0,1,0,0,0,0,0,0,0,0,S,S,S,S,S,S,S,S,HR,LR,S,S,S,HR,S,HR,L
R,1,0,0,1,0,0,0,0,1,0,0,HR,cluster1
39,M,27428,133,1,BB,0,0,0,0,0,0,1,1,0,1,1,0,0,1,0,0,0,0,0,1,1,0,
0,0,0,0,0,1,0,0,0,0,0,0,0,0,IR,S,IR,HR,IR,HR,HR,LR,HR,LR,LR,LR,L
R,HR,S,S,S,1,0,0,0,1,0,0,0,0,0,0,S,cluster5
31,F,196529,123,0,BB,0,0,0,0,0,0,0,0,0,0,0,0,0,0,0,0,0,0,0,1,1,0
,0,0,0,0,0,1,0,0,0,0,0,0,1,0,S,S,S,S,S,S,S,S,HR,LR,LR,LR,LR,HR,L
R,HR,HR,1,0,0,1,0,0,0,0,0,0,0,HR,cluster1
59,M,12707,498,1,BB,0,0,0,0,1,0,1,1,0,1,0,1,0,1,0,0,0,0,1,0,1,0,
0,0,1,0,0,1,1,0,0,0,1,0,0,0,HR,S,IR,HR,HR,HR,HR,IR,HR,IR,HR,IR,I
R,HR,LR,HR,IR,1,0,0,1,0,1,0,1,0,0,0,HR,cluster2
39,M,6630,106,1,BB,0,0,0,0,0,0,0,0,0,0,1,1,0,0,0,0,0,0,1,0,1,0,0
,0,0,0,0,1,0,1,0,0,1,0,0,0,S,S,S,S,S,S,S,S,HR,IR,HR,IR,LR,HR,LR,
HR,IR,1,0,0,1,0,0,0,0,0,0,0,HR,cluster0
44,F,60770,87,1,BB,0,0,0,0,1,0,0,1,0,0,0,1,0,1,0,0,0,0,0,0,0,0,0
,0,0,0,0,1,0,0,0,0,0,0,1,0,HR,S,LR,IR,IR,HR,LR,S,HR,LR,S,S,S,HR,
S,HR,LR,1,1,0,0,0,0,0,1,0,0,1,HR,cluster1
65,M,3090,130,0,BB,0,0,1,0,0,0,0,1,0,0,0,1,0,1,0,1,0,0,1,0,1,0,0
,0,0,0,0,1,1,0,0,0,1,0,0,0,HR,S,IR,HR,IR,HR,HR,IR,HR,IR,HR,HR,IR
,HR,LR,S,S,1,0,0,1,0,0,0,1,0,0,1,LR,cluster2
56,F,3167,88,1,BB,0,0,0,1,0,1,0,1,0,1,1,0,0,1,0,0,0,0,1,1,1,0,0,
0,0,0,0,1,1,0,0,0,1,0,0,0,HR,LR,HR,HR,HR,HR,HR,S,HR,IR,HR,HR,IR,
HR,LR,HR,HR,1,0,0,0,0,0,0,0,0,0,1,HR,cluster2
57,M,197720,46,1,BB,0,0,0,0,0,0,0,0,0,0,0,0,0,0,0,0,0,0,0,0,0,0,
0,0,0,0,0,0,0,0,0,0,0,0,0,0,S,S,S,S,S,S,S,S,S,S,S,S,S,S,S,S,S,1,
0,0,1,0,0,0,1,0,0,1,S,cluster4
43,M,5871,387,0,BB,0,0,0,0,0,0,0,0,0,0,0,0,0,0,0,0,0,0,0,0,0,0,0
,0,0,0,0,1,0,0,0,0,0,0,1,0,S,S,S,S,S,S,S,S,HR,LR,S,S,S,HR,S,HR,L
R,1,0,0,1,0,0,0,0,0,0,0,HR,cluster1
38,F,2864,432,0,BB,0,0,0,0,0,0,1,1,0,1,1,0,0,1,0,1,0,0,0,0,0,0,0
,0,0,0,0,1,0,0,0,1,0,0,0,0,HR,S,IR,HR,IR,HR,HR,LR,HR,IR,IR,IR,IR
,HR,LR,HR,LR,1,0,0,1,0,0,0,0,1,0,0,HR,cluster5
37,M,5300,819,0,BB,0,0,0,0,0,0,0,0,0,0,1,1,0,0,0,0,0,0,0,0,0,0,0
,0,0,0,0,1,0,0,0,0,0,0,1,0,S,S,S,S,S,S,S,S,HR,LR,S,S,S,HR,S,HR,L
R,1,0,0,1,0,0,0,0,0,0,0,HR,cluster1
43,M,7792,246,0,BB,0,0,0,0,0,0,0,0,0,0,0,0,1,0,0,1,0,1,0,0,1,0,0
,0,0,0,0,1,0,0,0,1,0,1,0,0,S,S,S,S,S,S,S,S,HR,HR,HR,HR,HR,HR,IR,
HR,LR,1,0,0,1,0,0,0,0,0,0,0,HR,cluster0
43,F,5004,437,0,F1,0,0,0,0,0,0,0,0,0,0,0,0,0,0,0,0,0,0,1,0,1,0,0
,0,0,0,0,1,0,0,0,0,0,1,0,0,HR,S,S,S,S,IR,S,S,HR,LR,IR,LR,LR,HR,S
,HR,IR,1,0,0,1,0,0,0,0,0,0,0,HR,cluster0
38,F,135745,239,1,BB,0,1,1,0,0,1,0,1,0,0,0,0,1,0,0,1,0,0,1,0,0,0
,0,0,0,0,0,1,0,0,0,1,0,0,0,0,HR,IR,HR,HR,HR,HR,IR,IR,HR,HR,HR,HR
,IR,HR,IR,HR,IR,1,0,0,0,0,0,0,1,0,0,1,HR,cluster2

40,F,11276,576,1,F1,0,0,0,0,0,0,0,0,0,1,0,1,0,1,0,1,0,1,0,0,1,0,
0,0,0,0,0,1,1,0,0,0,1,0,0,0,HR,S,LR,IR,LR,HR,IR,S,HR,HR,HR,HR,HR
,HR,LR,IR,LR,1,1,0,0,0,0,0,0,0,0,1,HR,cluster3
42,M,3048,439,1,BB,0,0,0,0,0,0,0,0,0,0,0,0,0,0,0,1,0,0,0,1,0,0,0
,0,0,0,0,1,0,0,0,1,0,0,0,0,S,S,S,S,S,S,S,S,HR,HR,HR,HR,HR,HR,IR,
HR,LR,1,0,0,0,0,0,0,0,0,0,1,HR,cluster0
45,M,10920,242,1,BB,1,0,1,1,0,0,1,1,0,0,1,0,0,1,0,1,0,0,1,1,1,0,
0,0,0,0,0,1,1,0,0,0,1,0,0,0,HR,S,IR,HR,IR,HR,IR,IR,HR,HR,HR,HR,H
R,HR,IR,S,S,1,0,1,1,0,0,0,0,0,0,1,S,cluster2

**Supplementary File 8** – Multifasta sequences with protease and transcriptase genes concatenated, aligned and edited for phylogenetic tree inference.

>1_O

actctttggcaacgacccgttgtcacagtaagaatagagggacagttaaaagaagcactg

ttagatacaggagcagatgacacagtgctagaagaaatagaattgggaggtagatggaaa

ccaaaaatgatagggggaattggaggtttcataaaagtaagacaatatgataatgttaca

atagacatatgtggaaaaagagcagtaggtacagttttagtaggacccacacctgtaaat

atcataggaaggaatattttgactcaaattggatgcactttaaatttttgtacagagatg

gaaaaggaagggaaaatttcaaaaattgggcctgaaaatccatacaatactccagtattt

gccataaagaaaaaggacagtactaaatggagaaaattactagatttcagagaacttaat

aagagaactcaagacttctgggaagttcaattaggaataccacatcccgcagggttaaaa

aagaaaaaatcagtaacagtactggatgtgggtgatgcatatttttcagttcccttagat

gaagacttcaggaaatatactgcatttaccatacctagtataaacaatgagacaccaggg

attagatatcagtacaatgtgcttccacagggatggaaaggatcaccagcaatattccaa

agtagcatgacaaaaatcttagagccttttagagaacaaaatccagacatagttatctat

caatacatggatgatttgtatgtaggatctgacttagaaatagggcagcatagaacaaaa

atagaggagctgagacaacatctgttgaggtggggatttaccacaccagacaaaaaacat

cagaaagaacctccattcctttggatgggttatgaactccatcctgataaatggacagta

cagcctatagtgctgcca

>2_MI

ACTCTTTGGCAACGACCCATCGTCACAATAAAGATAGGGGGGCAGTTAAAGGAAGCTCTA

ATAGAYACAGGAGCAGATGATACAGTATTyGAAGAAMTGGAGTTGCCAGGAAGATGGACA

CCAAAAATCATAGGGGGACTTGGRGGTTTTGTCAGAGTAAGACAGTATGATCAGATACTT

GTAGAAATTTGTGGACATAAAGTCGTGGGTACAGTATTAGTAGGACCTACACCTGCCAAC

ATAATTGGAAGAAACCTGTTGACTCAAATTGGTTGCACTTTAAATTTTTGTACAGAATTG

GAAAAGGAAGGGAAAATCTCAAAAATTGGGCCTGAAAATCCATACAATACCCCAATATTT

GCTATAAAGAAAAAGAACAGTAmTAGATGGAGGAAATTAATGGATCTCAGAGAACTTAAT

AAGAGAACTCAAGACTTCTGGGAAGTTCAATTAGGAATACCACATCCCGCAGGGTTAAAA

AAGAAAAAATCAGTAACAGTACTGGATGTGGGTGATGCATATTTTTCAGTTCCTTTATAT

GAAGATTTTAGGAAGTACACTGCATTTACcATACCTAGTCTAAATAATGAGACACCAGGG

ATTAGATACCAGTACAATGTGCTACCACAGGGATGGAAAGGATCAcCAGCAATATTCCAA

AGTAGCATGACAAAAATCTTAGAGCCTTTTAGAAAACAAAATCCAGAAATArTTATCTAT

CAATACGTGGATGATCTGTATGTAGGATCTGACTTAGAAATAGGGCAGCATAGAACAAAA

ATAGAGGAACTGAGAGAACATCTGTTGAGGTGGGGATTTTTCACACCAGACGAAAAACAT

CAGAAAGAGCCGCCATTCTTrTGGATGGGTTATGAACTCCATCCTGATAAATGGACAGTA

CAgCCTATAGAGCTACCA

>3_MI

ACTCTTTGGCAACGACCCTTAGTCACAATAAAAGTAGGGGGGCAACTAAAGGAAGCTCTA

TTAGATACAGGAGCAGATGATACAGTATTAGAAGACATAAATTTGCCAGGAAAATGGAAA

CCAAAAATGATAGGGGGAATTGGAGGTTTtATyAAAGTAAGACAGTATGATCAGGTACTY

RTAGAAATCTGTGGTCATAAAGCTATAGGTACAGTATTAGTAGGRCCTACACCTGTCAAC

ATAATTGGAAGAAATCTGTTGACTCArATTGGCTGYACYYTAAATTTTTGTACAGAACTG

GAAAACGAAGGAAAAATTTCAAAAATTGGGCCTGAAAATCCATACAATACTCCAGTATTT

GCCATAAAGAAAAAGGACAGTAATAAATGGAGAAAATTAATGGATCTCAGAGAACTTAAT

AAGAGAACTCAAGACTTCTGGGAAGTCCAATTAGGAATACCACATCCCGGAGGGTTAAAA

AAGAACAAATCAGTAACAATACTAGATGTGGGTGATGCATATTTTTCAGTTCCTTTAGAC

AAAGAATTCAGGAAGTACACgGCATTTACcATACCTAGTCTAAATAATGAGACACCAGGG

ATTAGATATCAGTACAATGTGCTTCCACAGGGAtgGAAAGGRTCACCAGCAATATTCCAA

AGTAGCATGACAAAAATCTTAGAGCCTTTTAGAAAACAAAATCCAGACATAGTTATCTAC

CAATACGTGGATGATTTGTATRTAGGGTCTGACTTAGAAATAGGGCAGCACAGAGCAAAA

ATCAAGGAGTTAAGAGAACATCTACTGAAATGGGGATTTTATACACCAGACAAAAAACAT

CAGGAAGAACCTCCATTCCGTTGGATGGGGTATGAACTCCATCCTGATACATGGACAGTG

CAGCCTATACAACTGCCA

>4_MI

ACTCTTTGGCAACGACCCGTAGTCACAGTAAAAGTAGGGGGACAGCTAAGGGAAGCTTTA

TTAGATACAGGAGCAGATGATACAGTATTAGAAGACATAACTTTGCCAGGAAAATGGAAA

CCAAgAATGATAGGGGGACTTGGAGGTTTTATCAAAGTAAAACAGTATGATAAyATACCC

ATAGAAATTTGTGGACACAAGGTTATARGTACAGTGTTGGTAGGACCCACGCCTGTCAAC

ATAATTGGAAGAAATACGATGACTCAGATTGGCTGTACTTTAAATTTYTGTACAGAATTG
GAAAAAGAAGGAAAAATTTCAAAAATTGGGCCTGAAAATCCATACAATACTCCAATATTT
GCCATAAAGAAAAAGGGCGGTACTAGATGGAGAAAAATAGTAGATTTCAGAGAACTTAAT
AAAAGAACTCAAGATTTTTGGGAGGTTCAATTAGGAATACCACATCCTGGAGGGTTAAAr
AAGAAAAAATCAGTAACAATACTGGATGTGGGGGATGCATATTTTTCAGTTCCYTTAGAT
RAGGAkTTCAGGAAGTAYACKGCATTcACCATACCTAGTATCAACAATGAGACACCAGGA
ATTAGGTACCAGTACAATGTRCTTCcACAAGGATGGAAAGGgTCACCAGCAATATTCCAA
TGTAGCATGACAAAAATYYTAGAACCCTTTAGAGCAAAAAATCCAGAMATAGTTATCTAC
CAATACGTGGATGATTTGTATGTAGCATCTGACTTAGAAATAGGGCAGCACAGAGCAAAA
ATTGATGAGTTAAGASAWCATCTAYTGAAATGGGGATTTTTTACACCAGATGAAAAACAT
CAAAAGGAACCCCCATTCCTTTGGATGGGGTATGAACTCCAYCCWGATAAATGGACAGTG
CAGCCTATACAATTGCCA
>5_MI
ACTCTtTgGcaACGACCATATGTCACAGTAAAGGTAGGGGGGcaAGTAAGGGAAGCTCTA
TTAGATACAGGAGCAGATGATACAATmTTTGAAGACATAGAGTTGCCAGGAAGATGGCAG
CCAAAAATGGTAGGGGGAATTGGAGGTTTTCTCAAAGTAAAGCAGTATGATCAGGTACCC
ATAGAAATCTGCGGACATAAAACTACAACTACAGTATTRGTAGGGCCTACACCTGCCAAC
ATAATTGGAAGAAAyYTGTTGACTCAGATTGGCTGCACTTTAAATTTTTGTACAGAATTG
GAAAAGGACGGAAAAATTTCAAAAATTGGGCCTGAGAATCCATACAATACTCCAGTATTT
GCCATAAAGAAAAAGAACAGTACTAAATGGAGAAAATTAGTAGATTTCAGAGAACTTAAY
AAGAGAACTCAAGACTTCTGkGAARTTCAATTAGGAATACCACATCCYTCAGGRTTAGWA
AAGAAAAAATCAGTAACAGTACTAGATATAGGTGATGCCTATTTTTCAGTrCCCTTAGAC
GAAGACTTCAGGAAGTACACTGCATTTACcATACCTAGTAYAAACAATGCGACACCAGGG
ACTAgATATCAGTaCAATGTGCTTCCACArGGAtgGAAAGGAtCaCCAGCAATATTCCAA
TGTAGCATGACAAAAATCTTAGATCCTTTTAGAMAACAAAATCCAGACATGATTATCTAT
CAATATGTRGATGATTTGTATGTAGCATCTGACTTAGAAATAGArCAGCATAGAACAAAA
ATAGAGGAGyTGAGACARTATCTGTGGAAGTGGGGRTTTTACACACCAGACAAAAAACAT
CAGAAGGAACCTCCATTCCTTTGGATGGGTTATGAACTCCATCCTGATAAATGGACAGTA
CAGCCTaTAGAGCTGCCA
>6_MI
ACTCTTTGGCAACGACCCATGGTCACAATAAGAGTRGAGGGACAGCTAACGGAAGCTCTA
TTAGATACAGGAGCAGATGATACAGTATTAGAAGAYATAAATTTGACAGGAAAATGGAAA
CCAAAAATGATAGGGGGACTTGGAGGTTTTATCAAAGTAAAACAGTATGATAACATAACA

ATAGACATTTGTGGACACAAGGCTACAGGTGCAGTATTGGTAGGACCTACACCTGTCAAC
ATAATTGGAAGGAGTATGTTGACTCAGATTGGTTGTACTTTAAATTTTTGTCTAGAAATG
GAAARAGAAGGAAAAATTTCAAAAATTGGGCCAGAAAATCCATACAATACTCCARTATTT
GCCATAAAGAAAAAARACAGTACTARATGGAGAAAATTAGTAGATTTCAGAGAACTTAAT
AAAAGAACTCAAGATTTTTGGGAAGTTCAATTAGGAATACCACATCCTGCAGGGTTACCA
AAGARCAAGTCAGTAACAGTACTGGATGTGGGGGATGCATATTTTTCAGTTCCCTTAGAT
AAAGAATTCAGGAAGTACACTGCATTCACYATACCTAGTTGCAACAATGAGACACCAGGA
ATTAGGTACCAATACAATGTGCTTCCACAAGGATGgaAAGGAtcaCCAGCGATATTCCAA
TmTAGYATGACAAAAATCTTAGATCCCTTTAGAGCAAAAAATCCAGAAATAGTTATCTAC
CAATACGTAGATGATTTGTAyGTAGGGTCTGACTTAGAAATAGGGCAGCATAGAGCAAAA
ATAGAAGAGTTrAGAGACCATCTCTTGAAATGGGGATTTACTACACCAGACmAAAAGCAT
CAAAArGAACCCCCATTCCTTTGGATGGGGTATGAACTCCATCCKGATAAATGGACAGTG
CAGCCTATACAATTGCCA
>7_MI
ACTCTTTGGCAACGACCCCTCGTCCCAATAAAAATaGGGGGGCAAGTAAAAGAAGCTcTA
TTAGATACAGGAGCAGATGAYACAGTATTAGAAGAAATGAATTTACCAGGAAAATGGATA
CCAAAAATGATAGGGGGAATTGGAGGTTTTATCAArGTAAGACAGTATGATCAGATAACC
ATAGAAATCTGTGGACATAARACTAYAGGTACAGTATTAATAGGACCTACACCTGTCAAC
ATAATTGGGAGAAATCTGTTGACGCAGCTTGGTTGCACTTTAAATTTTTGTACAGAAYTG
GAAAAGGAMGGGAAAATTTCAAAAATTGGGCCTGAAAATCCATACAATACTCCAGTATTT
GCCATAAAGAAAAAAGGTGGCGAKAGATGGAGAAAAwTAGTAGATTTCAGAGAACTTAAT
AAGAGAACTCAAGATTTCTGGGARGTTCAATTAGGAATACCACATCCCGCAGGATTAAAA
AAGAAmAARTCAGTAACAGTACTGGATGTGGGTGATGCATACTTCTCAGTTCCCTTAGAT
AAAGAATTCAGGAAGTATACTGCATTTACTATACCTAGTACAAACAATGAGACACCAGGA
ATTAGATATCAGTACAATGTGCTTCCACAGGGATGGAAgGGATCACCgGCAATATTCCAA
TATAGCATGACAAAAATCTTAGAGCCGTTTAGAAAACAAAATCCAGACATAGTyATCTAT
CAATACGTGGATGATTTGTATGTAGGATCTGACTTAGAAATAGGACAGCATAGAATAAAA
ATAGAGGAACTAAGACAACATCTGCTGCAGTGGGGGTTTTACACACCAGAACAAAAACAT
CAGAAGGAACCTCCATTCCTTTGGATGGGTTATGAACTCCAYCCTGATAAATGGACAGTA
CAGCCTATAGTGCTGCCA
>8_MI
ACTCTTTGGCAACGACCArTAGTCRCAATAAAGGTAGGGGGACAACTRAAGGAaGCCCTA
TTAGATACAGGAGCAGATGATACAGTATTAGAAGAAATGAATTTGCCAGGAAAATGGAAA

CCAAARATGATAGGGGGAATTGGAGGTTTTATCAAAGTAAGACAGTATGATCAGATAGyA
GTAGACATCTGTGGACATAAAGCTATAGGTACAGTATTAGTAGGACCTACACCTGTCAAC
ATAATTGGAAGAAATCTGTTGACACAGCTTGGTTGCACTTTAAaTTTTTGTACAGAAATG
GAAAAGGAAGGGAAAATTTCAAAAATTGGGCCTGAAAATCCATACAATACTCCAATATTT
GCCATAAAGAAAAAGGACAGTACAAAATGGAGAAAATTAGTAGATTTCAGAGAACTTAAT
AAGAGAACTCAAGACTTCTGGGAARTTCAATTAGGAATACCACATCCCGCAGGGyTAAAA
mAGAACAAATCAGTAACAGTACTAGATGTGGGTGATGCATATTTTTCAGTTCCCTTAGAT
AAAGACTTCAGGAAGTACACTGCATTTACCATACCTAGTATGAACAAyGAGAMACCAGGG
ATTAGATATCAGTACAATGTGCTGCCACAGGGrTGGAAAGGATCACCAGCAATATTCCAA
AGCAGCATGACAAAAATCTTAGAGCCTTTTAGAAAACAAAATCCAGACATAGTTATCTAT
CAATACGTGGATGATTTGTATGTAGGATCTGACTTAGAAATAGGGCAGCACAGAGCAAAA
ATAGAGGAGCTAAGAGAACATCTGTTGAGGTGGGGAtTTmCCACACCAGACAAAAAACAT
CAGAAAGAACATCCATTCCTTTGGATGGGTTATGAGCTCCATCCTGATAAATGGACAGTA
CAGCCTATAGTGCTGCCA

>9_MI

ACTCTTTGGCAACGACCCATCGTCACAATAAAAATAGGAGGGCAACTAAAGGAAGCTCTA
TTAGATACAGGAGCAGATGATACAGTATTAGAAGAAATGARTTTGCCAGGAAGATGGAAA
CCAAGAATGATAGGGGGAATTGGAGGTTTTGTCAAAGTAAGACARTATGATCAGrTACCC
ATrGAAATCTGTGGACAGAAGGTTATArGTACAGTATTAATAGGACCTACACCTGCCAAC
ATAATTGGAAGAAATCTGATGACACAGCTTGGTTkCACTTTAAATTTTTgTrCAGAAMTG
GAAAAGGAAGGGAAAATTTCAAAAATTGGGCCTGAAAATCCATACAATACTCCAGTATTT
GCYATAAAGAAAAAAGACAGTACTAAATGGAGAAAATTAGTAGATTTCAGAGAACTYAAC
AAGAGAACTCAAGACTTTTGGGAAGTTCAATTAGGAATACCACATCCCGSAGGGCTAAAG
AAGAACAAATCAGTAACAGTTCTGGATGTGGGTGATGCATATTTyTCAGTTCCCTTAGAT
GAAGACTTYAGGAAGTATACTGCATTCACCATACCTAGyMYAAACAATGAGACACCAGGA
GTAAGATATCAGTACAATGTGCTTCCACAGGGATGGAAAGGATCACcAGCAATATTCCAA
AGTAGCATGACAAAAATCTTAGAGCCTTTTAGAAAACAAAATCCAGACATAGtTATCTAT
CAATACGTGGATGATTTGTATGTAGGATCAGATTTAGAAATAGGGCAGCATAGAGCAAAA
ATAGAGGAACTGAGACAACATCTGTTGAGGTGGGGGTTTTACACACCAGACAAAAAACAT
CAGAAAGAACCTCCTTTCmwTTGGATGGGYTATGAGCTCCATCCTGATAMATGGACAGTA
CAGCCTATAGTGTTGCCA

>10_MI

ACTCTTTGGCARCGACCCCTCGTCACAGTAAAGATAGGGGGGCAACTAAAAGAAGCTCTC

yTAGATACAGGAGCAGATGATACAGTATTrGAAGAAATGTCTTTGCCAGGAAGATGGAAA
CCAAAAATGATAGGGGGAATTGGAGGTTTTATCAAAGTAAGACAGTATGAACAAATACCC
ATAGAAATATGTGGAAAGACAGCTATAGGTACAGTATTAATAGGACCCACACCTGTCAAC
ATAATTGGAAGAAAYCTGTTGACTCAGATTGGWTGCACTTTAAATTTTTGTAcAGAAATG
GAAAAGGAAGGRAAAATTTCAAAAATTGGGCCTGAAAATCCATATAATACTCCAGTATTT
GCCATAAAGAAAAAAGACAGTACTAAATGGAGAAAGTTAGTAGATTTCAGAGAACTTAAT
AAGAAAACTCAAGATTTCTGGGAAGTTCAATTAGGAATACCACACCCCGCAGGGTTAAAA
AAGAARAAATCAGTAACAGTACTAGATGTGGGTGATGCATATTTTTCAGTTCCYTTAGAT
AAAGAATTCAGGAAGTATACTGCATTYACmATACCTAGTATAAACAATGAGACACCAGGr
ATTAGATATCAGTACAATGTGCTTCCACAGGGATgGAAAGGcTCACCAGCAATATTCCAA
GCCAGCATGACAAAAATCTTAGARCCTTTTAGAAAACAAAACYCArAmwTAGTTATCTAT
CAATACATGGATGATTTGTATGTAGGATCTGACTTAGAAATAGGGCAGCAYAGAACAAAA
GTAGAGGAACTGAGACAACATCTGTTrAArTGGGGGTTTACCACACCAGACAArAAACAT
CAGAAAGAACCTCCATTCCTTTGGATGGGTTATGAACTCCATCCTGATAARTGGACAGTA
CAGCCTATAGTGCTGCCA
>11_MI
ACTCTTTGGcaACGACCAATAGTCACAGCAAAGATAGGGGGGcaACTAGTTGAAGTTTTA
TTAGATACAGGAGCAGATGATACAGTATTAGAAAAcATAAATTTGCCAGGAACATGGAAA
CCAAAAATmATAGGAGGAATTGGAGGTTTTCTCAGAGTAAGACArTATGAGCAAGTACCT
ATAGAACTCTGYGGGCATAAGGTTAGAGGTACAGTATTAGTAGGACCTACACCTGCCAAC
ATAATTGGAAGAAATGTGATGACTAAACTTGGCTGCACTTTRAATTTcTGTACAGAAATG
GAAAAGGAAGGAAAAATTTCAAAAATTGGGCCTGAAAATCCATACAACACTCCAGTATTT
GCCATAAAGAAAAAGAACAGTAATAGATGGAGAAAATTAGTAGATTTCAGAGAACTTAAT
AARAGAACTCAGGACTTCTGGGAAGTTCAATTAGGAATACCACATCCCKCAGGGTTAGAA
AAGAAAAAATCAGTAACAGTACTAGATGTGGGTGATGCATATTTTTCAGTTCCCTTAGAT
GAAGACTTCAGGAAGTATACTGCATTtACCATACCTAGTACAAACAATGAGACACCAGGA
ATTAGATATCAGTACAATGTGCTTCCACAAGGAtGgAAGGgAtCACCAGCAATATTCCAA
AGTAGCATGACAAAAATCTTAGAGCCTTTTAGAAAACAAAATCCAGACATAGTTATCTGT
CAATACGTGGATGATTTGTATGTAGCATCTGACTTAGAAATAGGGCAACATAGAATAAAA
GTAGAGGAACTGAGAAACCATCTGCTrAGGTGGGGGTTTTTTACACCAGACGAAAAACAT
CAGAAAGARCCTCCATTCCATTGGATGGGKTATGAACTCCATCCTGATAAATGGACAGTR
CAGGCTATAAAGCTGCCA
>12_MI

ACTCTTTGGCAACGACCCGTCGTCACAATAAGGATAGGGGGGcaAMTAAAGGAAGCTCTA
TTAGATACAGGAGCAGATGATACAGTATTyGAAGArCTGAATTTGCCAGGAAAGTGGAAA
CCAAAAATGATAGTGGGAATTGGAGGTTTTACCAAAGTAAGASAGTATGAGrACRTACCC
ATAGAAATTTGCGGACATAAAGTTAGAGGTACAGTATTAGTAGGACCTACACCCGCCAAC
ATAATTGGAAGAAATCTGTTGACTCAGCTTGGCTGCACTTTAAATTTTTGTACAGAATTG
GAAAAAGAAGGAAAAATTTCAAAAATTGGGCCTGAAAATCCATACAATACTCCAATATTT
GCAATAAAGAAAAAAAACAGTACCAGGTGGAGGAAATTAGTAGATTTCAGAGAACTTAAT
AAAAGAACTCAAGAtTTTTGGGAAGTTCAATTAGGAATACCACATCCCGGAGGGTTAAAG
AAGAAAAAATCAGTAACAGTACTAGATGTGGGTGATGCATATTTTTCAGTTCCCTTAGAy
AAAGACTTCAGGAAGTATACTGCATTTACcATACCTAGTCTAAACAATGAAACACCAGGG
ATTAGATACCAGTACAATGTGCTTCCACAAGGATGGAAgGGATCACCAGCAATATTCCAA
AGTAGCATGACAAAAATCCTAGAGCCTTTTAGGAAACAAAATCCAGACATAATAATTTAT
CAATACGTGGATGATTTATATGTAGGATCTGACTTAGAAATAGGGCAGCATAGAACAAAA
ATAGAGGAACTGAGACAACATCTGTTGAGGTGGGGGTTTTTCACACCAGACGAAAAACAT
CAGAAAGAGCCTCCATTCCTTTGGATGGGTTATGAACTCCATCCAGATAAATGGACAGTA
CAGCCTATCATACTGCCA

>13_MI

ACtCTTTGGCAACGACCAGTAGTCACAATAAAGGTAGAGGGGCAACYAATGGAAGCTCTA
yTAGATACAGGAGCAGATGATACAGTATTAGAAGAYATAGAGTTGCCAGGAAGATGGACA
CCAAAACTGATAGGGGGAATTGGAGGTTTTGTCAAAGTAAGACARTATGATCAGrTACTA
ATAGAAATATGTGGAYrCAGARTTAGARGTACAGTGTTrGTAGGACCTACACCTGCcAAC
ATAATTGGAAGRAATCTRATGACACAGCTTGGTTGCACTTTAAACTTTTGTACAGAAATG
GAAAAGGAAGGGAAAATCTCAAAAATTGGGCCTGAgAACCCATACAATACTCCAATATTT
GCTATAAAGAAAAAGAACAGTACTAGATGGAGAAAATTAGTAGATTTTAGAGAACTTAAT
AAGAGAACTCAAGATTTTTGGGAAGTTCAATTAGGACTACCACATCCTGCAGGGTTAAAA
CAGAACAAATCAGTAACAGTACTAGATGTAGGTGATGCATATTTTTCAATTCCCTTAGAC
AAAGACTTCAGGAAGTATACTGCATTCACCATACCTAGTATAAACAATGAGACACCAGGG
ATTAGATATCAGTACAATGTGCTGCCACAGGGATGGAAAGGATCACCAGCAATATTCCAA
AGTAGCATGACAAAAATCTTAGAGCCTTTTAGAAAACAAAATCCAGACATAGTTATCTAT
CAATACGTGGATGATTTGCTTGTAGGATCTGACTTAGAAATAGGGCAGCATARAACAAAA
ATAGAAGAACTGAGACAACATCTGTTGAGGTGGGGATTATTCACACCAGATGAAAAACAT
CAGAAAGAACCTCCATTCCTTTGGATGGGTTATGAGCTCCATCCTGATAARTGGACAGTA
CAGCCTATAATGCTGCCA

>14_MI

ACTCTTTGGCAACGAcCACTCGTCACAGTAAAAGTAGGGGGGCAATTAAAGGAAGCTCTA

TTAGATACAGGAGCAGATGATACAGTATTAGAAGACATGGAGTTGCCAGGAAGATGGAAA

CCAAAAATGATAGGGGGAATTGGAGGTTTTATTAAAGTAAGACAGTATGATCARATACTC

ATAGAAATCTGTGGACATAAAGCCATAGGTACAGTGTTAATAGGGCCTACACCTATCAAC

ATAATTGGAAGAAATATGTTGACTCAGATTGGCTGCACTTTAAATTTTTGTACAGAAATG

GAAAAGGAAGGAAAAATTTCAAAGATTGGACCTGAAAATCCATATAATACTCCAGTATTT

GCTATAAAGAAAAAGGACAGTACTAAATGGAGAAAAYTAGTGGATTTCAGAGAACTTAAT

AAGAGAACTCAAGACTTCTGGGAAGTGCAATTAGGAATACCACATCCCGCAGGGCTACAA

ATGAATAAATCAGTAACAGTACTGGATGTGGGTGATGCATATTTTTCAGTTCCCTTAGAC

AAAGACTTCAGGAAGTATACTGCATTtACcATACCTAGTATAAACAATGAAACACCAGGG

atTAGATATCAGTACAATGTGCTTCCACAGGGATGGAAAGGATCACCAGCAATATTCCAA

AGTAGCATGACAAAAATCTTAGAGCCTTTTAGAAAGCAAAATCCAGACATAGTTATCTAT

CAATACGTGGATGATCTGTACGTAGGATCTGACTTACMAATAGAGCAGCATAGAACAAAG

ATAGAGGAGCTGAGACAACATCTGTTGAGGTGGGGATTTACCACACCAGACAAAAAACAT

CAGAAAGAACCTCCATTCCTGTGGATGGGTTATGAACTCCATCCTGATAAATGGACAGTA

CAGCCTATAAYGTTACCA

>15_MI

ACTCTTTGGCAACGACCCATAATCACAATAAAGGTAGGGGGGCAAsyAATGGAAGCTCTA

ATAGATACAGGAGCAGATGATACAGTATTAGAAAACATAAATTTACCAGGCAAATGGAAA

CCAAAATTGATAGGGGGAMTTGGAGGTyTTGTCAgAGTTAAACAGTATGATAACGTACCC

ATAGAAATTTGTGGACAcAAGrTTACAGGTACAGTGCTGGTAGGACCTACACCTGCcAAc

ATAATTGGAAGAAATvTGTTGACTAAGATTGGCTGCACTTTAAATTTTTGTAAAGAATTG

GAAGAGGACGGAAAGATTTCAAARATTGGGCCTGAAAATCCATACAATACTCCAATATTT

GCTATAAAGAAAAAGAACAGTACTAAATGGAGAAAATTAATGGATTTCAGAGAACTTAAT

AAGAGAACTCAAGACTTCTGGGAAGTTCAATTAGGAATACCGCATCCCGGGGGGTTAAwA

RAGAAAAAATCAATAACAGTACTGGATGTGGGTGATGCATATTTTTCAATTCCCTTASAT

GAAGAYTTCAGGAAGTATACTGCATTTACYATACCTAGTACAAACAATGCGACACCAGGG

rTTAGGTATCAGTACAATGTGCTTCCACAGGGATGGAAAGGATCACCAGCAATATTYCAA

AGTAGCATGACAAAAATCTTAGAGCCTTTTARAAAACAAAATCCAGACATAGTTATCTAT

CAATATGTGGATGATTTGTATGTAGCATCTGACTTAGAAATAGGGCAGCATAGAACAAAA

ATAAAGGAGCTGAGAGAATWTCTGTGGAAGTGGGGATTTTACACCCCAGACAGAAAACAT

CAGACAGAACCTCCGTTCCTTTGGATGGGTTATGAACTCCATCCTGATAAATGGACAGTA

CAGCCTATAGTGCTGCCA

>16_MI

ACTCTTTGGCAGCGACCCrTCGTTACAATAAAGATAGGGGGGCAGCTAARGGAAGCTCTA

ATAGATACAGGAGCAGATGATACAGTATTAGAGGATATGCATTTGCCAGGAAAATGGAAA

CCAAAAATAATAGGTGGAATTGGAGGTTTTGTCAArGTAAGACAGTATGATCAGGTAGAT

GTAGAAATCTGTGGACATAAAGTTACAGGTTCAGTATTAATAGGACCTACACCTAGCAAC

ATAATTGGAAGGAATCTGTTGWCTCAGCTTGGCTGCACTTTAAATTTTTGTACAgAAATG

GaAAAGGAAGGAAAAATTTCAAGAATTGGGCCTGAAAATCCATACAATACTCCAGTATTT

GCAATAAAGAAAAAAAACAGTACTAGATGGAGAAAATTAGTAGATTTCAGAGAACTAAAT

AAGAGAACTCAAGACTTCTGGGAAGTTCAGTTAGGGATACCACATCCAGCAGGGTTAAAA

AAGAAAAAATCAGTAACAGTRTTGGATGTGGGTGATGCATATTTTTCAGTTCCCTTACAT

GAAGACTTCAGGAAGTATACTGCCTTTACcATACCTAGTACAAACAATGAAACACCAGGG

GTTAGATATCAATACAATGTGCTTCCACAGGGATGGAAAGGATCACCAGCAATATTTCAA

AGTAGTATGACAMAAATCCTAAAGCCTTTTAGAGAACAAAATCCAGACATGGTTATCTAT

CAATATGTGGATGATCTGTATGTAGGATCTGACTTAGAAATAGGGCAACATAGAGCAAAA

ATAGAGGAACTAAGACAACATCTGTTrAAGTGGGGGTTTTTCACACCAGAGCAAAAACAT

CAAAAAGAACCTCCATTTCTTTGGATGGGGTATGAACTCCATCCTGATAAATGGACAGTA

CAGCCTATAACGCTGCCA

>17_MI

ACTCTTTGGCAACGACCCATCGTCACAATAAAgATAgGGGGGcaACTAAAGGAAGCTCTA

TTAGATACAGGAGCAGATGATAcAGTATTTCAAGAAATGAATTTGCCAGGAAGATGGACA

CCAAAAATgATAATRGGAATTGGAGGTTTGGTCAAAGTAAGACAATATGATGAGGTACCC

CTAGAAATCTGTGGACATAAAGTTATAGGTACAGTATTAGTAGGACCTACACCTGCCAAC

GTAATTGGAAGGAATCTGATGACTCAGCTTGGCTGCACTTTAAATTTTTGTACAGAATTG

GAAAAGGAAGGAAAAATTTCAAAAATTGGGCCTGAAAACCCATACAATACCCCAGTATTT

GCTATAAAGAAAAAGAACAGTACTAAATGGAGAAAATTACTAGATTTCAGAGAACTTAAT

AAGAGAACTCAAGACTTCTGGGAAGTTCAATTAGGAATACCACACCCTGCAGGGTTAAAA

AAGAACAAATCAGTAACAGTACTGGATGTGGGTGATGCATATTTCTCAATTCCTTTAGAT

AAAGACTTCAGGAAGTATACTGCATTTACCATAcCTAGTACAAACAATGAAACACCAGGA

ATTAGATATCARTACAATGTGCTTCCACAAGGATGGAAAGGATCGCCAGCAATATTCCAA

GATAGCATGACACAAATCTTAGAGCCTTTTAGAAAACAAAATCCAGACATAGTTATCTAT

CAATACGTGGATGACTTGTATGTAGGATCTGACTTAGAAATAGAACAGCATAGAACAAAA

GTAGAGGAACTGAGACAACATCTGTGGAAGTGGGGrTTTACACACCAGACGAAAAACAT

CAGAAAGAACATCCATTCCTTTGGATGGGTTATGAACTCCATCCTGACAAATGGACAGTA
CAGCCTATACAGCTACCA
>18_MI
ACTCTTTGGCAACGACCATTCGTCTCAATAAAGGTAGGGGGGCAAATAACAGAaGCTCTA
TTAGATACAGGAGCAGATGATACAGTATTmGAAGGAATAGATTTGCCAGGAAGATGGAAA
CCAAAAATAATAGGGGGACTTGGAGGTTTTATMAAAGTAARAGAGTATGATCAGATACCC
ATAGAAATTTGCGGACATAAAACTATAGGTACAGTATTAGTAGGGCCTACACCTGCCAAC
ATAATTGGAAGAAATCTGTTGACTCAGATTGGCTGCACTTTAAATTTTTGCACAGAATTG
GAAGAGGAgGGaAAgatTTCAAAAATTGGGCCTGAAAATCCATACAATACTCCAGTATTT
GCCATAAAGAAAAAAGACAGTAmTAAATGGAGAAAATTAATGGATTTCAGAGAACTTAAT
AARAGAACTCAAGACTTCTGGGAAGTKCAATTAGGAATACCACATCCCGGAGGmTTAAAA
AAGAAAAAATCAGTrACAGTACTAGAYGTGGGTGATGCATATTTTTCAGTTCCATTAGAC
AAAGAGTTCAGAAAGTAcACTGCATTtACcATACCTAGTACAAACAATGAGACACCAGGG
ATTAGATATCAGTACAATGTACTGCCACAGGGATGGAAAGGAtcaCcAGCAATATTCCAA
AGTAGCATGACAAAAATCTTAGAGCCTTTTAGAAAACAAAATCCAGAAATAGTTATCTAT
CAATACGTGGATGATTTGTATGTAGGATCTGACTTAGAAATAGGGCAGCATAGAACAAAA
ATAGAGGAGCTAAGACAATAtCTGTGGAAGTGGGGATTTTtTACACCAGACAAAAAACAT
CAGAAAGAACCTCCATTCCTTTGGATGGGTTATGAACTCCATCCTGATAAATGGACAGTR
CAGCCTATAGAGCTGCCA
>19_MI
ACTCTTTGGCAACGACCTATAGTCACAGTAAAGATAGGGGGaCAGCTAATAGAAGCTCTA
TTAGATACAGGAGCAGATGATACAGTATTMGAAGACATAAATTTGCCAGGAAAATGGAAA
CCAAAAATGATAGGGGGAATTGGAGGTTTTGTCAAAGTAaAACAGTATGACAACATACTC
ATAGACATTTGTGGACACAARGCTAYAGGTACAGTGTTAGTAGGACCTACACCTGCCAAC
ATAATTGGAAGAAATATGATGACTCAGATTGGTTGTACTTTAAATTTTTGTACAGAAaTG
GAAAAGgaAgGaAAAATTTCAAAAATTGGGCCTGAAAATCCATATAATACTCCAGTATTT
GCTATAAAGAAAAAAAATAGTACTAGATGGAGAAAATTAGTAGATTTCAGAGAACTTAAT
AAGAGAACTCAAGACTTCTGGGAAGTTCAATTAGGAATACCACATCCCTCAGGGTTAAAA
CAGAAAAAGTCAGTAACAGTACTGGATGTGGGTGATGCATATTTTTCAGTTCCCTTAGAT
AAAGAAtTCAGGAAATAYACTGCATTTACTATAcCTAGTATAaACAATGAGACACCAGGG
ATTAGATATCAGTACAATGTGCTYCCACAGGGATGGAAAGGATCACCAGCAATATTCCAA
AGCAGCATGACAAAAATCTTAGAGCCCTTTAGAAAACAAAATCCAGACATAGTTATCTAT
CAATACGTGGATGATTTGTATGTAGGATCTGACTTAGAAATAGGGCAGCATAGAACAAAA

ATAGAGGAACTGAGACAACATTTGTTGAGGTGGGGAyTTACCACACCAGACGAAAAGCAT

CAGAAAGAACCTCCATTCCTTTGGATGGGTTATGAACTCCATCCTGATAAATGGACAGTA

CAGCCTATAGTGCTGCCA

>20_MI

ACTCTttGgcAACGACCCCGTGTcACAGTAAAGATAGGGGGGCAACTAAAGGAAGCTCTC

tTAGATACAGGAGCAGATGATACAGTATTTGAAGAAATGAATTTACCAGGAAGATGGAAA

CCAAACwTGATAGGGGGACTTGGAGGTTTTATCAAAGTGAGACAGTATGATCAGATACCC

ATAGAAATTTGCGGACATAAAGCTGTAGGTACAGTrTTAATAGGACCTACACCTGCCAAC

ATAATTGGAAGAAATCTGTTGACTCAGATTGGCTGCACTTTAAATTTTTGTGCAGAAATG

GAAAAGGAAGGaMAAATTTCAAAAATTGGGCCTGAAAATCCATACAATACTCCAGTGTTT

GCCATAAAGAAAAAGRACAGTACTARATGGAGAAAATTAKTAGATTTCAGAGAACTTAAT

AAGAGAACTCAAGACTTCTGGGAAGTTCAATTAGGAATACCACATCCCGCAGGGTTAAAA

AAGAACAAATCwGTAACAGTACTAGATGTGGGTGATGCATATTTCTCAGTTCCCTTATAT

GAAGACTTCAGGAAGTATACTGCATTCACCATACCTAGTACAAACAATGCGACACCAGGG

GTTAGATATCAGTACAATGTGCTTCCACAGGGATgGAAAGGATCACCAGCAATaTTCCAA

TGTAGCATGACAAAAATCTTAGAGCCTTTTMGAAARCAAAATCCAGACATAGTTATCTAT

CAATAyGTGGATGATTTGTATGTmGGATCTGACTTAGAAATAGGGCAGCATAGAGAAAAA

ATAGAGGAACTGAGACAACATCTGTTGAAGTGGGGATTCTTCACACCAGACAAAAAACAT

CAGAAAGAACATCCATTCAATTGGCTGGGTTATGAACTCCATCCTGATAAATGGACAGTR

CAGCctatagtgctgcca

>21_MI

ACTCTTTGGCAACGACCCCTTGTCACAATAAAGATAGAGGGGcaACTAAAGGAAGCTCTA

TTAGATACAGGAGCAGATGATACAGTrTtAGAAGAMATRACTTTGTCAGGAAAATGGAAR

CCAAAAATGATAGGGGGAATtGGAGGTTTTATCAAAGTAAGACAGTATGATCAGGTACCC

ATAGAAATCTGTGGACATAAAGTTATAGGTACAGTATTAGTAGGACCTACACCTGTCAAC

ATAATTGGAAGAAATCTGTTGACTCAGCTTGGTTGCACATTAAaTTTTTGTACAGAAATG

GAAAAGGAAGGRAAAATTTCAAAAATTGGGCCTGAAAATCCATACAATACTCCAATATTT

GCCATAAAGAAAAAGAACAGTACTAGATGGAGAAAAATAGTAGATTTCAGAGAACTTAAT

AAGAGAACTCAAGACTTCTGGGAAGTTCAATTAGGGATACCACATCCYGCAGGGTTAAAR

TTGAAAAAATCAGTAACAGTACTGGATGTGGGTGATGCATATTTTTCAGTKCCCTTAGAT

AAAGACTTCAGGAAGTATACTGCATTTACCATACCTAGTACAAATAATGARACACCAGGG

ATTAGATATCARTACAATGTGCTCCCACAGGGATGgAAaGgATCACCAGCAATATTyCAA

AGTAGCATGACAAAAATCCTAGAGCCTTTTAGAAAACAAAATCCAAACATAGTTATCTAt

CAATAyGTGGATGACTTGTATGTAGCATCTGACTTAGAAATAGAGCAGCATAGAATAAAA
ATAGAGGAACTGAGARCACTCCTGTTAARATGGGGATTTACCACACCAGARCAAAAACAT
CAGAAAGAACCTCCATTCCTTTGGATGGGTTATGAACTTCATCCAGACAAATGGACAGTA
CAGCCGATAGTGCTGCCA
>22_MI
ACTCTTTGGCAACGACCAsTmGTCACAATAARGGTAGGGGGGCAACTAAAGGAAGCTCTA
TTAGATACAGGAGCAGATGATACAGTATTAGAAGAAATAGAGTTGCCAGGAAGATGGAAA
CCAAAAATGATAGGGGGAATTGGAGGTTTTRTCAAAGTAAAACAGTATGAWCAGGTACCC
ATAGACATTTGCGGGCATAArGTTGTAGGTwCAGTATTAGTAGGACCTACACCTGCCAAC
ATAATTGGAAGAAATCTGTTGACTCAACTTGGCTGTACTTTAAaTTTTTGTACAGaAMTg
GAAAAGGAAGGRAAAATTTCAAAAATTGGGCCTGAAAATCCATACAATACTCCAGTATTT
GCCATAAAGAAAAAGGAyGGTACTAAATGGAGAAAATTAGTAGATTTCAGAGAACTTAAT
AAGAGAACTCAAGACTTCTGGGAAGTTCAATTAGGGATACCACATCCCGCRGGATTAAAA
AAGAAAAAATCAGTAACAGTACTAGATGTGGGTGATGCATATTTTTCAGTTCCCTTAkAT
GAAGACTTTAGGAAGTATACTGCATTTACCATACCTAgTACAAACAATGAGACACCAGGG
ATTAGATATCAGTACAATGTGCTTCCRCAGGGAtgGAAAGGATCACCAGCAATATTCCAA
GCTAGTATGACAAAAATCTTAGAGCCKTTTAGAAAACAAAATCCAGAAATAGTTATCTAT
CAATACGTGGATGATTTGTATGTAGGATCTGACTTAGAAATAGGGCAGCATAGAACAAAA
ATAGAGGAGCTGAGAGAACATCTGTTGAAGTGGGGATTTTACACACCAGACAAAAAACAT
CAGAAAGAACCCCCATTCCTTTGGATGGGTTATGAACTCCATCCYGATAAATGGACAGTA
CAGCCTATAgTRCTGCCA
>23_MI
ACTCTTTGGCAACGACCCwwTGTCACAATAAAAATAGGGGATCAACTAAmAGAGGCTCTA
ATAGATACAGGAGCAGATGATACAGTATTAGAAGAAATGAATTTGCCAGGAAGATGGAMA
CCAAAAATCATAGGGGGACTTGGAGGTCTTATTAGAGTAAGACAGTATGAGCAGATATTA
GTAGAATTCTGCGGACATAAAGTTATAGGTACAGTATTAATAGGGCCTACACCTGCCAAC
ATAATTGGAAGAAATCTGTTGACTCAAATTGGCTGCACTTTAAaTTTTTGYACAGAAAtg
GAAAAGGAAGGAAAAATTTCAAAAATTGGGCCTGAAAATCCATACAATACTCCwGTATTT
GTCATAAAGAGAAAAGACAATACTAAATGGAGAAAATTAGTAGATTTCAGAGAACTTAAT
AAAAGAACTCAAGACTTCTGGGAAGTTCAATTAGGAATACCACATCCyGCAGGGTTAAAA
AAGAAGAAGTCAGTAACAGTACTGGATGTGGGTGATGCATATTTTTCAGTTCCTTTAGAT
GAAGAATTCAGGAAGTAYACTGCATTYaCcATACCTAGTATAAACAATGAGACACCAGGG
ATTAGATATCAGTACAATGTGCTTCCACAGGGAtgGAAAGGATCGCCAGCAATATTTCAA

AGTAGCATGACAAAAATCTTAGAGCCTTTTAGAAAACAAAATCCAGAAATAGTTATCTAT

CAGTACGTGGATGACTTRTATGTAGGATCTGATTTGGAAATAGGACAGCATAGAACAAAA

ATAGAGGAACTGAGACAACATCTGTTGAGGTGGGGGTTAACCACACCAGACCAAAAACAT

CAGAAAGAACCCCCATTCCTTTGGATGGGTTATGAACTACATCCTGATAAATGGACAGTA

CAGCCTATAACGCTGCCA

>24_MI

ACTCTTTGGCAACGACCCMTAGTCACAATAAawGTAGGGGGACAGCTAACGGAAGCTATA

TTAGATACAGGAGCAGATGATACAGTATTAsAAGATATAAATTTACCAGGAAAATGGAAA

CCAAGAATGATAGGGGGAATTGGAGGTCTTATCAAAGTAAGAcAGTATGATCAGRTACCC

ATAGAARTCTGTGGACATAAAMTTACAAGTACAGTATTAATAGGGCCTACACCKGTCAAC

ATAATTGGAAGAAATTTGATGACTCAGCTTGGCTGCACTTTAAaTTTTTGTACAGAAATG

GAAAAGGAAGGAAAAATTTCAAAAATTGGGCCTGAAAATCCATACAATACTCCAGTATTT

GCTATAAAGAAAAAGAACAGTACTAGATGGAGAAAATTAGTAGATTTCAGAGAACTTAAT

AAGAGAACTCAAGACTTCTGGGAAGTTCAATTAGGAATACCACACCCCGCAGGGTTAAAA

AAGAAAAAGTCAGTAACAGTACTGGATGTGGGTGATGCATATTTTTCAGTTCCCTTAGAT

AAAGAATTCAGGAAGTACACTGCATTTACCATACCTAGCATAAAYAATGAGACACCAGGG

ATTAGATATCAGTACAATGTGCTTCCACAGGGAtgGAAgGGATCACCAgCAATATTCCAA

AGTAGTATGACAAAAATCTTAGAGCCTTTTAGAAAGCAAAATCCAGAGATAGTTATCTRT

CAATACGTGGATGATTTGTATGTAGGATCTGACTTAGAGATAGGGCAACATAGAACAAAA

ATAGAAGAACTGAGAGAAYATCTGTTGAGGTGGGGATTTTACACACCAGATCAAAAACAT

CAGAAAGAGCCTCCATTCCATTGGATGGGTTATGAACTCCATCCTGATAAATGGACAGTA

CAGCCTATAAYACTGCCA

>25_MI

ACTCTTTGGCAACGACCACTcGTCACAGTAArGWTAGGGGGGcaWMTAAAGGAAGCTCTA

TTAGATACAGGAGCAGATGATACAGTATTAGAAGAAATGGAGTTGCCAGGAAGATGGAAA

CCAAAAATGATAGGGGGAATTGGAGGTTTTATCAAAGTAAGACAGTATGATCAGATACCC

ATAGAAATCTGTGGACATArARCAATAGGTACAGTATTARTAGGGCCTACACCTGTCAAC

ATAATTGGAAGrAATCTGTTGACTCAGATTGGCTGCACTCTAAaTTTTTGTRCAGAAwTR

GAAAAGGAAGGAAAAATTTCAAAAATTGGGCCTGAAAATCCATACAATACTCCrATATTT

GCCATAAAGAAAAAGAACAGTACTAAATGGAGAAARyTAGTAGAYTTyAGAGAACTTAAT

AAGAGAACTCAAGACTTCTGGGAAGTTCAATTAGGAATACCACATCCCGCAGGGTTAAAA

AAGAAAAAATCAGTAACAGTACTGGATGTGGGTGATGCATATTTYTCAGTTCCCTTACAT

GAAGAMTTCAGGAAGTACACTGCATTTACtATACCTAGTACAAACAATGAGACACCAGGG

ATTAGATATCAGTACAATGTGCTTCCACAGGGAtGGaAaGGtTCACCAGCAATATTCCAA
AGTAGCATGACAAAAATCTTAGAACCTTTTAGAAAACAAAATYCAGACATAGTTATCTAT
CAATATATGGATGATTTGTATGTAGGATCTGACTTAGAAATAGAGCAGCATAGARCAAAA
RTAGAAGAGCTGAGACAACATCTGTTGAGGTGGGGATTYTTCACACCAGAYCAAAAACAT
CAGAAAGAACCTCCATTCCTCTGGATGGGTTATGAACTCCATCCTGATAAATGGACAGTA
CAGCCTATAGTGCTGCCA
>26_MI
ACTcTTTGGCAACGACCCCTAGTCGAAATAAAGGTAGGGGGGCAACAAAAGGAAGCTCTA
TTAGAYACAGGAGCAGATGATACAGTATTAGAAGAAATGAATTTGCCAGGAAGATGGAAA
CCAAAAATGATAGGGGGAATTGGAGGTTTTATCAAAGTAAAACARTATGATrAyATACTy
ATAGAAATTTGTGGAAAGAAAACAATAGGTACAGTGTTAGTAGGACCTACACCTGTCAAC
ATAATTGGAAGGAATCTGTTGACTCAGMTTGGYTGCACTTTAAATTTTTGTACAGAAATG
GAAAAGGAAGGAAAAATTTCAAAAATTGGGCCTGAAAATCCATACAATACTCCAGTATTT
GCyATAAAGAAAAAGGACAGTACtAAATGGAGAAAATTAGTRGATTTCAGAGAACTTAAT
AAGAGAACTCAAGACTTTTGGGAAGTYCAATTAGGAATACCACATCCCGCAGGGTTAAAR
CAGAAAAAATCAGTGACAGTACTAGATGTGGGTGATGCATATTTyTCAGTTCCCTTAGAT
AArGACTTCAGGAAGTATACTGCATTcACCATACCTAGTATAAACAATGAGACACCAGGG
aTTAGATATCAGTACAATGTACTTCCACARGGATGGAAAGGATCACCAGCAATATTCCAA
TGCAGCATGACAAAAATYTTAGAGCCTTTTAGAAAACAAAATCCAGACATAGTTATCTAy
CAATACATGGATGATTTGTATGTAGGATCTGACTTAGAAATAGGGCARCATAGAACAAAA
ATAGAGGAACTGAGACAACATCTGTTGAGGTGGGGRTTTACCACACCAGACAAAAAACAT
CAGAAGGAACCTCCATTCCTTTGGATGGGTTATGAACTCCATCCTGATAAATGGACAGTA
CAGCCTATAGTGCTGCCA
>27_MI
ACTCTTTGGCAACGACCMGTAGTCATRGCAAAAATAGGGGGACAGCTaAcGGaAGYTCTA
TTAGATACAGGAGCAgATAATACTRTATTAGaAaAcATArATTTGCCAGGAAAATGGAAA
CCAAGAATAGTAGGGGGAATTGgAGGTTTWSTCAArGTAAAACAGTATGATAACATATGC
ATAGAAATTTGTGGACAYAAGGTTATAGGTTCAGTAWTGGTAGGACCTACRCCTGTTAAC
ATAATTGGAAGAGATAcATTGACTCGGATTGGCTGTACTTTAAATTTTTGTACAGAAATG
GAAAAGGAAGGAAAAATTTCAAAAATTGGGCCTGAAAATCCATACAATACTCCAATATTT
GCCATAAGGAAAAAGAAYAGTAATAGATGGAGAAAATTAGTAGATTTCAGAGAACTTAAT
AAGAGAACTCAAGACTTCTGGGAAGTCCAGTTAGGAATACCACATCCCGCAGGATTAAAA
AAGAACAAATCAGTAACAGTAYTGGATGTGGGTGATGCATATTTTTCAATTCCCTTAGAT

GAAGATTTCAGGAArTATACTGCATTtACCATACCTAGTATAAACAATGAGACACCAGGG
ATTAGATATCAGTAcAATGTGCTTCCACAGGGATGGAAAGGATCACCAGCAATATTCCAA
AGTAGCATGACAAAAATCTTAGAGCCTTTTAGAAAACAGAATCCAGACATAGTTATCTAT
CAATAYGTGGATGATTTGTATGTAGGATCTGACTTAGAAATAGGGCAACATAGAACAAAA
ATAGAGGAACTGAGACAACATCTGTTGAGGTGGGGACTTTTCACACCAGATGAAAAACAT
CAGAAAGAACCTCCATTCCTTTGGATGGGTTATGAACTCCAYCCTGATAAATGGACAGTA
CAGCCTaTAGWGCTGCCA
>28_MI
ACTCTTTGGCAACGACCCCTAGTCAACATAAAGGTAGGGGGacaAATAAAGGaAGCTCTA
TTAGATAcAGGAGCAGATGATACAGTATTAGAAGACATAAATTTGTCAGGAAAATGGAAA
CCAAAAATGATAGGGGGAATTGGAGGTTTTATCAaAGTAAAACAGTATGATGACATACCC
ATAGAAATTTGTGGcCacaAgGCTGTAGGTACAGTTTTGGTAGgaCCCACGCCTGTCAAC
ATAATTGGAAGAAATTTGTTGACTCAAATTGGGTGTACGTTAAATTTTTGTACAGAATTG
GAAAAGGAAGGGAAAATTTCAAAAATTGGGCCTGAAAATCCATATAATACTCCAGTATTT
GCAATAAAGAAAAAAGACAGTACTAAATGGAGAAAATTAGTAGATTTCAGAGAACTTAAT
AAGARAACTCAAGACTTCTGGGAAGTTCAATTAGGAATACCACACCCCKCwGGGTTAAAA
AAGAATAAATCAGTAACAGTACTAGATGTGGGTGATGCATATTTTTCAGTTCCCTTAGAT
AAAGAATTCAGGAAGTATACTGCATTtACcATACCTAGTACAAACAATGAGACACCAGGG
ATTAGATATCAGTACAATGTGCTCCCGCAgGGATGGAAAGGATCACCAGCAATATTCCAA
AGCAGCATGACAAAAATCTTAGAGCCTTTTAGAAAAAGAAATCCAGACATAGTTATCTAT
CAATAYGTGGATGATTTGTATGTAGGATCTGATTTAGAAATAGAACAGCATAGAACAAAA
ATAGAGGAATTAAGACAACATCTGTTGAAGTGGGGATTTTwCACACCAGACAAAAAACAT
CAGAAAGAACATCCATTCCTTTGGATGGGTTATGAACTCCATCCTGACAAATGGACAGTA
CAGCCTATAGTGCTGCCA
>29_MI
ACTCTTTGGCAACGACCCTTAGTCACAATAARGATAGGGGGACAGYTAAAGGAAGCTCTA
TTAGATACAGGAGCAGATGATACAGTATTAGAAGACATGARTTTRTCAGGAAAATGGAAA
CCAAAAATGATAGGGGGAATTGGAGGYTTTATCAAAGTAARACARTATGATrACRTAWCC
RTAGACATTTGTGGACACAAGGCTATAGGTACAGTATTAGTAGGACCTACACCTGTCAAC
ATAATTGGAAGAAATCTGTTGACTCAGATTGGcTGCACTTTAAATTTTTGTACAGAAAtG
GAAAAGGAAGGGaaAATTTCaagAATTGGGCCTGAAaACCCATATaATACTCCAGTATTT
GCYATAAAGaaaAAAGATAGTACTAArTGGAGAAARTTAGTAGATTTCAGAGAACTTAAT
AAGAGAACTCAAGACTTCTGGGAAGTTCAATTAGGAATACCACATCCCgCAGGGTTaAAA

rAGaaAAAATCAGTAACAGTAcTGGATGTrGGTGATGCATATTTtTcAGTTCCCTTAGAT

AAAGACTTCAGGAAGTATACTGCATTYACYATACCTAGTACAAATAATGAAACACCAGGG

gTTAGGTATCAGTACAATGTGCTTCCCCAGGGATGGAAAGgaTcACCAGCAATATTCCAA

ArTAGCATGACAAAAATCTTAGAGCCTTTTAGAAAACAAAATCCAGACATAGTTATCTAT

CAATATGTGGATGATTTGTTGGTAGGATCTGACTTAGAAATAGGGCAGCATAGAACAAAA

ATAGAGGAATTGAGACAACAYCTGTTGAAGTGGGGRTTTACCACACCAGACAAAAARCAC

CAGAAAGAACCTCCATTCCTTTGGATGGGTTATGAACTCCATCCTGATAgATGGACAGTA

CAGCCTATAGTGCTGCCA

>30_MI

ACTCTTTGGCAACGACCAATAGTCACAATAAARRTAGAGGGACAGCTAAGGGAAGCTTTA

TTAGATACAGGAGCAGATGATACAGTATTAGAAGACATAAATTTGCCAGGAAAATGGAAA

CCAAAAATARTAGGGGGAATTGGAGGTTTTRTCAAAGTAAAACAGTATGATAACATACTC

ATAGAAATTTGTGGGCACAAGGTTAGAGGTACAGTGGTGGTAGGACCTACGCCTGTCAAC

GTAATTGGAAGAAATATGTTGACTCAGCTTGGTTGTACTTTAAATTTTTGTACAgAAATG

GAGAAGGAAGGGAAAATTTCAAAAATTGGGCCTGAAAATCCATATAATACTCCAGTGTTT

GCCATAAAGAAGAAAAACAGTACTARATGGAGAAAATTGGTAGAyTTTAGAGAACTTAAT

AAGAGAACTCAAGACTTCTGGGAAGTTCAGCTAGGAATACCACATCCAGCAGGGTTRGAA

AAGAAAAAGTCAGTAACAGTACTRGATGTGGGTGATGCATATTTTTCAGTTCCCTTAGAT

AAAGAGTTCAGGAAATATACTGCATTYACCATACCTAGTATAAACAATGGGACACCAGGG

ATTAGATATCAGTACAATGTGCTTCCACAGGGATGGAAAGGATCACCAGCAATATTCCAA

AGCAGCATGACAAAAATCTTAGAGCCTTTTAGAAAACAAAACCCTGACATGATTATCTAT

CAATATGTGGATGATTTATATGTAGCATCTGACCTAGAAATAGGACAGCATAGAACAAAA

ATAGAGGAACTGAGGAAACATCTGTTGGCGTGGGGATTTTACACACCAGACGAAAAACAT

CAGAAAGAACCTCCATTCCTTTGGATGGGTTATGAACTCCATCCTGACAAATGGACAGTA

CAGCCTATAGTGCTGCCA

>31_MI

ACTCTTTGGCAACGACCCtTCGTCGACATGAAGATAGGGGGacaAATAATAGAAGCTCTA

ATAGATACAGGAGCAGATGATACAGTACTAGAAGAAATARATTTGCCAGGAAGATGGAAA

CCAAAAATAATAGGGGGAATTGGAGGTTTTCTCAAAGTAAGACAGTATGATCAGATACCC

GTAGAAATTTGTGGAAAGAAAGCTGTAGGTACAGTAGTAGTAGGACCTACACCTTgtaAC

ATAATCGGAAGAAATCTGTTGACTCAGATTGGTTGCACTTTAAATTTtTGTACAGAATTG

GAAAATGAAGGGAAAATTTCAAAAATTGGGCCTGAAAATCCATATAACACTCCAGTATTT

GCCATAAAGAAAAAAGACAGCACTAAATGGAGAAAAATAGTAGATTTTAGAGAACTTAAT

AAGAGAACTCAAGACTTCTGGGAAGTCCAGTTAGGAATACCACATCCCGCAGGGATAAAA
AAGAACAAATCAGTaACAGTACTGGATGTGGGTGATGCATATTTTTCAGTTCCCTTAGAT
AAAGACTTCAGGAAGTATACTGCcTTtACCATACCTAGTATAAACAATGAGACACCAGGG
ATTAGGTACCAGTACAATGTGCTTCCACAGGGATGGAAAGGATCACCAGCAATATTCCAA
AGTAGCATGACAAAGATCTTAGAGCCTTTTAGAAAACAGAATCCAGACATAATTATCTAT
CAATACGTGGATGATTTGTATGTAGGATCTGACTTAGAAATAGGGCAGCATAGArAAAAA
ATAGAGGAACTAAGACAACATCTGTGGAGGTGGGGATTTTACACACCAGACAAAAAACAT
CAGAAAGAACCTCCATTCCTTTGGATGGGGTATGAACTCCACCCTGATAAATGGACAGTA
CAgCCTATAGTGCTGCCA

>32_MI

ACTCTTTGGCAACGACCAATAGTCACAATAAAGGTAgGGGGGcaACTAACGGAAGCTATA
TTAGATACAGGAGCAGATGATACAGTATTAGAAGACATAGAGTTGTCAGGAAGATGGAAA
CCAAAAATgATAGGGGGAATTGGAGGTTTTCTCAAAGTAAAAGAGTATGATCAGGTACCC
ATAGAAATTTGTGGACAAAAGGTTATAGGTACaGTATTAGTAGGGCCTACGCCTGTCAAC
ATAATTGGAAGAAATCTGWTGACTCAGCTTGGCTGCACTTTAAATTTtTGTACAGAAyTG
GAAAAGGAAGGAAAAATTTCAAAAATTGGGCCTGAAAATCCATACAATACTCCAGTATTT
GCCATAAAGAAAAAGAACAGTAMTAGATGGAGAAAATTAGTAGATTTCAGAGAACTTAAT
AAGAGAACTCAAGACTTCTGGGAAGTTCAATTAGGAATACCACATCCCGCAGGGTTAAAA
CAGAAAAAATCAGTAACAGTACTAGATGTGGGAGATGCATATTTTTCAGTTCCCTTAGAT
rAAGACTTCAGGAAGTAcACTGCATTtACCATACCTAGTATAAACAATGAGACACCAGGG
ATTAGATATCAGTACAATGTGCTTCCACAGGGAtgGAAAGGAtCaCCAgCAATATTCCAA
AGTAGCATGACAAAAATcTTAGAGCCTTTTAGAAAACAAAATCCAGACATGATAATYTAT
CAATACGTGGATGATTTGTATGTAGGwTCTGACTTAGAAATAGGGCAACATAGAGCAAAA
ATAGAGGAACTGAGACAAyATCTGTGGAAGTGGGGGTTTTACACACCAGACGAAAAACAT
CAGAAAGAACCTCCATTCCTTTGGATGGGTTATGAACTTCATCCTGATAAATGGACAGTA
CAGCCGATCAAGCTGCCA

>33_MI

actcTTtGGCAACGACCAGTAGTCACAATAaAAATAGGGGGACAGCTAAGGGAAGCTrTA
TTAGATACAGGAGCAGATGATACAGTATTAGAAGACATAACTTTGCCAGGAAAATGGAAA
CCAAAAATgATAGGGGGAATTGGAGGTCTkGTCAAAGTAAAACAGTATGArGAyGTACTC
GTAGAAATTTGTGGACATAAAGYTAGAGGTACAGTATTGATAGGACCTACGCCTGTCAAy
ATAATTGGAAGAAATATGATGACTCAGCTTGGTTKTACTTTAAATTTTTGTGCAGATATG
GAAAAAGAAGGAAAAATTTCAAGRATTGGGCCTGAAAATCCATACAATACTCCAGTATTT

GCCATAAAGAAAAAGGrCAGTACTGAATGGAGAAAATTAGTAGATTTTAGAGAACTTAAT

AAAAGAACTCAAGATTTTTGGGAGGTTCAATTAGGAATACCGCATCCTGCRGGGTTAAAA

AAGAAAAAGTCAGTAACAGTACTRGATGTGGGGGATGCATAYTTCTCAGTTCCTTTAGAT

AAGGAGTTCAGRAAGTACACTGCgTTCACCATACCTAGTCTCAAYAATGAGACACCAGGA

ATCAGGTACCAGTACAATGTGCTTCCACAAGGATGGAAAGGATCACCAGCAATATTCCAA

TGTAGCATGACAAAAATCCTAGATCCATTTAGAGCAAAAAATCCAGAATTAGTTATCTAT

CAATAYGTrGATGATTTGTATGTAGGGTCTGACTTAGAAATAGGGCAGCATAGAGCAAAA

ATAGAGGAATTAAGAAAYCATCTACTGAAATGGGGATTtACTACACCAGAYArAAAACAT

CAAAARGAACCCCCATTCCTTTGGATGGGGTATGAACTCCATCCTGATAAATGGACAGTG

CAGCCTATAcaattacca

>34_MI

ACTCTTTGGCAACGACCCATCGTCACAATGAGGGTCGGGGGGcaACTAATAGAAGCTCTA

ATAGATACAGGAGCAGATGATACAGTATTAGAAgACATAAATTTGCCAGGAAAATGGAAA

CCAAAAATAATAGGGGGAATTGGAGGTTTTGTCAGAGTAAGAGAGTATGAGsAAGTACCC

ATAGAAATTTGTGGACATAAAGCtATAGGTTCAGTATTrGTAGGACCTACACCTGCCAAC

ATAATTGGAAGAAAyTTGTTGACTCARATTGGTTGCACTTTAAATTTTTGTRCAGAtATG

GaAAAAGAAGGAAAAATTTCAAAAATTGGGCCTGAAAACCCATACAATACTCCARTATTT

GCCATAAAGAAAAAGAATAGTAATAGATGGAGAAAATTAGTAGATTTCAGAGAACTTAAT

AAAAGGACTCAAGACTTCTGGGAAGTTCAATTAGGGATACCACATCCCGGAGGGTTAAAA

AAGAAAAAATCAGTAACAGTACTGGATGTGGGTGATGCATATTTTTCAATTCCCTTAGAT

GAAGACTTCAGGAAGTATACTGCATTTACCATACCTAGTACAAACAATGAAACACCAGGG

ATTAGATATCAGTACAATGTGCTTCCACAGGGAtgGAAAGGRTCACCAGCAATATTCCAA

AGTAGCATGACAAAAATCTTAGAGCCTTTTAGGAAACAAAATCCAGACATArTCATCTAT

CAATACGTGGATGATTTGTATGTAGGATCTGACCTAGAAATAGGGCAGCATAGAACAAAA

ATAGAGGAATTGAGGCAACATCTGTTGACATGGGGACTTTTCACACCAGACCAAAAACAT

CAGAAAGAACCTCCATTCCTTTGGATGGGGTATGAACTCCAtCCTGATAAATGGACAGTA

CAGCCTATAGTGCTGCCA

>35_MI

ACTCTTTGGCAACGACCCTACGTCACAATAAAGGTAGGGGGACAGCTAACGGAAGCTCTA

TTAGATACAGGAGCAGATAATACAGTAWTAGAArAcatAGATTTGCCAGGAAAATGGAAA

CCAAAAATGATAGGGGGAATTGGAGGTTTTATCAAAGTAAAACAGTATGATAATGTACTC

ATAGAAATTTGTGGACACAAGACTATAGGTTCAGTGTTAATAGGACCTACACCTGTCAAC

ATAGTTGGAAGAGATACrATGACTmRGATTGGTTGTACTTTAAATTTTTGTACAGAATTA

GAAAAAGAAGGRAAAATTTCAAAAATTGGGCCTGAAAATCCATATAATACTCCAGTGTTT
GCCATAAAGAARAAAGACRGTACTAAATGGAGAAAAWTAGTAGATTTCAGAGAACTTAAT
AAGAGGACCCAGGATTTCTGGGAAGTTCAATTAGGAATACCACATCCTkCAGGGTTGAAA
AAGAAAAAGTCAGTAACAGTACTGGATGTGGGTGATGCATATTTTTCAGTTCCCTTAGAT
GAAGAATTCAGGAAGTATACTGcATTTACCATACCTAGTGTAAACAATGAGACACCAGGG
aTTAGATATCAGTACAATGTGCTACCACaGGGATGGAAAGGATCACCAGCAATATTCCAA
AGCAGCATGACAAGAATCTTAGAGCCTTTTAGAAAACAAAATCCAGACATAGTTATCTAT
CAATACGTRGATGATTTATATGTAGGATCTGACTTAGAGATAGAACAGCATAGAACAAAA
ATAGAGGAACTGAGACAACATCTGTGGAGGTGGGGGTTTACACACCAGACCAAAAACAT
CAGAAAGAACCTCCATTCCTTTGGATGGGGTATGAACTCCATCCTGACAAATGGACAGTA
CAGCCTATAGTGCTGCCA
>36_MI
ACTCTTTGGCAACGACCCCTAGTCACAGTAAAAGTAGGGGGaCARYTaAAGGAAGCTCTA
TTAGATACAGGAgcAGATGATACAGTATTAGAAGATATAAATTTGCcAgGaAAATGGAAA
CCAAAAATGATAGGGGGAATTGgAGGTTTTATCAAAGTAAAAcAGTATGATaACATAGTC
ATAGAAATWTGTGGrCACAAgGCTATAGGTACAGTGTTGGTAGGACCTACTCCTGTCAAC
ATAATTGGAAGAAATCTGTTGACTCAAATTGGyTGTACTTTAAATTTTTGTACAGAAATG
GAAAAAGAAGGRAAAATTTCAAGAATAGGRCCTGAAAATCCATACAATACTCCAGTATTT
GCCATAAAGAAAAAAGACAGTACAAAATGGAGGAAATTAGTAGATTTCAGAGAACTWAAT
AAGAGAACTCAAGATTTTTGGGAGGTTCAATTAGGAATACCGCACCCwGCAGGGTTAAAA
AAGAAYAAGTCAGTRACRGTACTGGATGTGGGGGATGCATATTTTTCAGTTCCCTTAGAT
GAGGATTTCAGGAAGTACACTGCRTTcACCATACCcAGTACCAACAATGAGACACCAGGA
ATyAGGTAyCAGTACAATGTGCTTCCACAAGGATGGAAgGGATCACCAGCAATATTCCAA
TGCAGCATGACAAAAATCTTAGACCCYTATAGAGCAAAAAATCCAGACCTRGTTATCTAC
CARTACATGGATGATTTGTATGTAGGGTCTGACTTAGAAATAGGGCAGCATAGAGCAAAA
ATAGAGGAATTAAGAGAACATCTACTGAGGTGGGGATTTACCACACCAGACAAAAAACAT
CAGAAAGAACCTCCATTCCTTTGGATGGGGTATGAACTCCATCCTGATAAATGGACAGTA
CAGCCTATAGTGCTGCCA
>37_MI
ACTCTTTGGCAACGACCctaTGTCACAGTAAAGATAGGGGGGCAGCTAAGGGAAGCTCTA
TTAGATACAGGAGCAGATAAyACAGTAATAGAAGACATAACTTTGCCAGGAAGATGGAAA
CCACAAATGATAGGGGGAATTGGAGGTTTTGTCAAAGTAAGACAGTATGATCAGGTACCT
GTAGAAATTTGTGGACATAAAGCTATAGGTACAGTGTTAGTAGGACCTACACCTGTCAAC

ATAATTGGAAGAGATCTGTTgACTCGGATTGGTTGCACTTTAAATTTTTGTACAGAAyTG
GAAAAGGAAGGAAAAATTTCAAAAATTGGGCCTGAAAATCCATACAATACTCCAGTATTT
GCCATAAAGAAAAAAGGTGGTACTAGATGGAGAAAATTAGTAGATTTCAGGGAACTTAAT
AAGAAAACTCAAGACTTCTGGGAAGTTCAATTAGGAATACCACATCCTGCAGGGTTAAAA
AAGAAGAAATCAGTAACAGTACTGGATGTGGGTGATGCATATTTTTCAGTTCCCTTAGAT
AAAGACTTCAGGAAGTATACTGCATTtACcATACCTAGTATTAATAATGAAACACCAGGG
ATTAGATATCAGTAYAATGTGCTGCCACAGGGATGGAAAGGATCACCAGCAATATTCCAA
AGTAGCATGACAAAAATCTTAGARCCTTTTAGAAAACGAAATCCAGACATAATTATCTAT
CAATACGTRGATGATTTGTATGTAGGATCTGACTTAGAAATAGGGCAGCAYAGAACGAAA
GTAGAGGAACTGAGACAACATCTGTTGARGTGGGGGTTTTACACACCAGATGAAAAACAT
CAGAAAGAACCyCCATTCCTTTGGATGGGTTATGAACTCCATCCTGATACATGGACAGTG
CAGCCGATAGTGCTGCCA
>38_MI
ACTCTTTGGCAACGACCAATAGTCACAATAAAGrTaGGGGGAGAGCTAAGGGAAGCTCTA
TTAGATACAGGAGCAGATGATAcAATAATAGArAACATAAATTTGCCAGGaaAATGGAAA
CCAAAAATAATAGGGGGAATTGGAGGGTTTGTCAAGGTAAAACAGTATGATGACATACCC
ATAGAAATTTATGAATACAAAGTTAGAAGTACAGTGTTAGTAGGACCTACACCTGTTGAC
ATAATTGGAAGAAATCTRATGACTCAGATTGGCTGCACTTTAAATTTTTGYACAGAATTA
GAACAGGAAGGAAAAATTTCAAAAATTGGGCCTGAAAATCCATATAATACTCCAGTATTT
GCCATAAAGAAAAAGAACAGTACTAAATGGAGAAAATTGGTAGATTTCAGAGAACTTAAT
AAGAGAACTCAAGACTTCTGTGAAGTTCAATTAGGAATACCACATCCTGCAGGGTTAAAA
AAGAAAAAGTCAGTAACAGTACTGGATGTGGGTGATGCATATTTTTCAGTTCCCTTAGAT
GAAGAATTCAGGAAGTACACTGCATTCACcATACCTaGTATAAACAATGAAACACCAGGG
ATTAGATATCAGTACAATGTGCTTCCACAGGGATGgAAaGGATCACCAGCAATATTTCAA
AGCAGCATGACAAAAGTCTTAGATCCTTTTAGAAAACAAAATCCAGACCTAGTTATCTAT
CAATACGTGGATGATTTATATGTAGGATCTGACTTAGAAATAGGGCAACATAGARCAAAA
ATAGAGGAACTGAGACAACATCTGTGGAGGTGGGGGTTTTACACACCAGATAAAAAACAT
CAGAAAGAACCGCCATTCCTTTGGATGGGTTATGAACTCCATCCTGATAAATGGACAGTA
CAGCCTATAAAGCTGCCA
>39_MI
ACTCTTTGGCAACGACCCCTCGTCACAGTAAAGATAGAAGGACAACTAAARGAAGCTCTA
TTAGATACAGGAGCAGATGATACAGTATTAGAAGAACTARCGTTGCCAGGAAGATGGAAG
CCAAAAATGATAGGGGGAATTGGAGGTTTTATCAAAGTAAAACAGTATGATCAGATACTA

GTAGAAATCTGTGgACATAAAGCTrTAGGTACAGTATTAGTAGGACCTACACCTGTCAAC
ATAATTGGAAGAAATTTGTTGACTCAGATTGGTTGCACTTTAAATTTTTGTACAGAAATG
GAAAAGGAAGGAAAAATTTCAAAAATTGGGCCTGAAAATCCATACAATACTCCAGTATTT
GCCATAAAGAAAAAGGACAGTACTAAATGGAGAAAACTAGTAGACTTyAGAGAACTTAAT
AAGAGAACTCAAGACTTCTGGGAAGTTCAATTAGGAATACCACATCCCGCAGGGTtAAAA
AAGAAYAAATCAGTGACAGTACTAGATGTAGGTGATGCATATTTTTCAGTTCCyTTAGAT
GAAGACTTCAGGAAGTACACTGCATTtACcATACCTAGTACAAACAATGAGACACCAGGG
ATTAGATATCAGTACAATGTGCTKcCACAGGGaTGGAAAGGATCACCAGCAATATTCCAA
AGCAGCATGACAAAAATCTTAGAGCCTTTCAGAAAACAAAATCCAGACATGGTYATATAT
CAATACATGGATGATTTATATGTRGGATCTGACTTAGAAATAGGGCARCATAGAACAAAA
ATAGAGGAGCTRAGaCARCATCTGTTGARGTGGGGATTTACCACACCAGACAArAARCAT
CAGAAAGAACmTCCRTTCCTTTGGATGGGGTATGAACTCCATCCAGATAAATGGACAGTA
CAGCCTATAATGCTGCCA

>40_MI

WCTCTTTGGCAACGACCCATCGTCAACATAAAGATAGGRGGGCAAATAATAGAAGCTCTG
TTAGATACAGGAGCAGATGATACAGTATTAACAGAAATAAATTTACCAGGAAGATGGAAA
CCAAAAATAATAGGGGGAATTGGAGGTTTTGTCAGAGTAAAACAGTATGATCAGRTACCY
ATAGAAATCTGTGGACAAAAAGTTrTAACTACAGTATTAGTAGGACCTACACCTGCCAAC
ATAATTGGAAGAAATCTGATGACTCAGCTTGGCTGTACTTTAAATTTTTGTaCAGAAATG
GAAAAGGAAGGAAAAATTTCAAAAATTGGGCCAGAAAATCCATACAATACTCCAGTATTT
GCCATAAARAAGAAAAACAGCAATAGATGGAGAAAAwTAGTAGATTTCAGAGAACTTAAT
AAGAGAACTCAAGATTTCTGGGAAGTyCAATTAGGAATACCACATCCTGCAGGGTTAGAA
AAGAAAAAATCAGTAACAGTACTGGATGTGGGTGATGCATATTTTTCAGTTCCCTTAGAT
GAAGAyTTCAGGAAGTATACTGCcTTtACcATACCTAGCACAAACAATGAGACACCAGGT
ATTAGATATCAGTACAATGTGCTTCCACAGGGATGGAAgGGATCACCAGCAATATTCCAA
AGTAGCATGACAAGAATCTTRGAGCCTTTTAGAAAACAAAATCCAGACATAGTTATCTGy
CAATATGTGGATGATTTGTATGTAGCATCTGACTTAGAAATAGGGCAACATAGAACAAAA
GTAGAGGAACTGAGACAACAyCTGTTRAGGTGGGGATTTTTCACACCAGArCAAAAACAT
CAAAAGGAACCTCCATTCCATTGGATGGGTTATGAACTCCATCCTGATAAATGGACAGTA
CAGCCTATAGAATTACCA

>41_MI

ACTCTTTGGCARCGACCCGTMRTCGAAATAAAGGTAGGGGGGCAACYAATAGAAGCTCTA
TTAGATACAGGAGCAGATGATACAGTATTAGAAGACATTAATTTGCCAGGAAAATGGAAA

CCAAAAATGATAGGGGGAATTGGAGGTTATCTCAAAGTAAGACAGTATGAyCAGGTACCC
ATAGAAATTTGTGGACATAAAgTTATAAGTACAGTATTAGTAGGACCTACACCTGTTAAC
ATAATTGGAAGAAGTCTGATGACTCAGCTTGGCTGCACTTTAAATTTTTGTACAGAAATG
GAAAAGGAAGGAAAGATTTCAAAAATTGGGCCCGAAAATCCATACAATACTCCAGTATTT
GCTATAAAGAAAAARGGCAGTGATAGATGGAGAAAATTAGTAGATTTCAGAGAACTTAAC
AAAAGAACTCAAGACTTCTGGGAAGTTCAATTAGGAATACCACACCCCGCAGGGTTAAAR
AAGAAMAAATCAGTAACAGTACTGGATGTGGGTGATGCATATTTTTCAATWCCCTTAGAT
RAGGACTTCAGAAAGTACACTGCATTtACcATACCcAGTACRAACAATGAAACACCAGGA
ATYAGATATCAGTATAATGTGCTTCCACAGGGATGGAAAGGATCACCAGCAATATTCCAA
AGTAGCATGACAAAAATCTTAGAGCCTTTTAGAAAACRAAATCCAGRrATAATTATCTAy
CAATACGTAGATGATTTGTATGTAGGATCTGACTTAGAAATAGGRCAGCATAGAGCAAAA
ATAGAGGAACTGAGAGAACATCTGCTGAGGTGGGGGTTGACCACACCAGACCAAAAACAT
CAGAAAGAACCACCATTCCTTTGGATGGGTTATGAACTCCATCCTGATAAGTGGACAGTR
CAGCCTRTAGTGCTGCCA

>42_MI

ACTCTTTGGCAACGACCAGTAGTCATAGCAAAAATAGGGGGACAGCTAAAAGAAGTTCTA
TTAGATACAGGAGCAGATGATACAGTAATAGAACATATAARTTTGCCAGGAAAATGGMAA
CCAAAAATGATAATGGGAATTGGAGGTTTGGTCAAAGTGAAACAGTATGATAACGTACCC
ATAGAAATTTGTGGATAcAAGGTTACAGGTACAGTGTTGGTAGGACCTACACCTGYCGAC
ATAATTGGAAGAAATATAATGACTCAACTTGGTTGCACCTTAAATTTTTGTACAGAAAtG
GAGAAGGAAGGAAAAATTTCAAAAATTGGGCCTGAAAATCCATACAATACCCCAATATTT
GCCATAAGGAAAAAAGACGGTACTAAATGGAGRAAAGTAGTAGATTTCAGAGAACTTAAT
AAGAGAACTCAAGATTTTTGGGAGGTTCAATTAGGAATACCACATCCTGCAGGGwTAAAA
AAGAACAARTCAgTRACAGTACTAGATGTGGGGGATGCATATTTTTCAGTTCCCTTAGAT
GAGGACTTCAGGAAGTACACTGCATTcACcATACCTAGTGTCAACAATGAAACACCAGGA
ATYAGGTACCAGTACAATGTGCTCCCACAAGGATGGAAAGGATCACCAGCAATATTCCAA
AGTAGCATGACAAAAATCCTAGAGCCCTTTAGAGCAAAAAATCCAGACATAGTTATCTGC
CAATACGTAGATGATTTGTATGTAGGGTCTGAYTTAGAAATAGGGCARCACAGAGCAAAA
ATAGAyGAGCTGAGAGAACATCTACTGAAATGGGGATTyACTACACCAGACAAGAAATAT
CAAAAAGAACCCCCATTYCTTTGGATGGGGTATGAACTCCATCCTGATAAATGGACAGTG
CAGCCTATACAATTGCCA

>43_MI

ACTCTTTGGCAACGACCCATCGTCGAAGTAAAGATAGGGGGGCAAATAARAGAAGCTATA

TTAGATACAGGAGCAGATGATACAGTATTCGAAGACCTAACTTTGCCAGGAAGATGGACA
CCAAAAATGATAGGGGGAATTGGAGGTTTTGTCAAAGTAAGACAGTATGATCAGGTAcCC
ATAGAAATCTGTGGAAAGAAAATTATAAGTACAGTATTAGTAGGACCTACACCTGCCAAC
ATAATTGGAAGAAAYCTGATGACTCAACTTGGTTGCACTTTAAATTTTtGtACAGAAwTG
GAAAAGGAAGGGAAAATTTCAAAAATTGGGCCTGAAAATCCATATAATACTCCArTATTT
GCCATAAAGAAAAAGAACAGTAATAGATGGAGAAARTTAGTAGATTTCAGAGAACTTAAT
AAGAGAACGCAAGACTTCTGGGAAGTTCAGTTAGGAATACCACATCCCGGwGGGTTAAAA
AAGAAAAAATCAGTAACArTACTGGATGTGGGTGATGCATAyTTTTCAATwCCCTTAGAT
CCAGAATTCAGGAAGTATACTGCATTTACCATACCTAGTATAAACAATGAGACACCAGGG
ATTAGATATCAGTACAATGTGCTACCACaGGGATGGAAaGGATCACCAGCAATATTCCAA
AGYAGCATGACAAAAATCTTAGAGCCTTTTAGAAAACAAAATCCAGAMATAATTATCTAT
CAATACGTGGATGATTTGTATGTAGGATCTGACTTAGAAATAGGRCAGCATAGAATRAAA
ATAGAGGAACTGAGACAACATCTGYTGAAGTGGGGACTTTTCACACCAGACGAAAAACAT
CAGAAAGAACCTCCATTCCTTTGGATGGGTTATGAACTCCATCCTGATAAATGGACAGTA
CAGCCTATAGTGCTGCCA

>44_MI

ACTCTTTGGCAACGACCAATAGTCACAGTAAAGGTAGGGGGGCAmATAACGGAAGCTCTA
ATAGATACAGGAGCAGATGATACAGTATTYGAaGrcATAGATTTACCAGGAAGATGGACA
CCAAAATTGATAGGGGGAATTGGAGGTTTTATGAGAGTAAAACAGTATGAGCAGATACCC
ATAGAAATTTGTGGACATAAAGYCAYAGGTACAGTATTAGTAGGACCTACACCTGCCAAC
ATAATTGGAAGAAATCTGTTGACTCAGATTGGCTGCACTTTAAATTTYTGTGCAGAATTG
GAAAAGGATGGAAAAATTTCAAAAATTGGGCCTGAAAATCCATACAATACTCCARTATTT
GCCATAAAGAArAag---AGTGGyAGATGGAGAAAAWTAGTRGATTTCAGAGAACTTAAT
AAGAGAACTCAAGACTTCTGGGAAGTTCAATTAGGAATACCACATCCCGCAGGGTTACAC
AAGAAAAAATCAGTAACAGTACTAGATGTGGGTGATGCATATTTTTCAGTTCCCTTAGAT
GAAGACTTCAGGAAGTATACTGCATTYACCATACCTAGTAyAAAYAATGCGACACCGGGG
GTTAGATATCAGTACAATGTGCTTCCACARGGAtgGAAAGGATCACCAGCAATATTCCAA
ArTAGCATGACAAAAATCTTAGAGCCyTTTAGAACAMArAATCCAGACATAGTTATCTAy
CAATACGTGGATGACTTGTATGTArGCTCTGACTTAGAAATAGGGCAGCATAGRAArAAG
ATAGAGGAGCTAAGACAACATCTRTGGAGGTGGGGATTTTACACACCAGACGArAAACAT
CAGAAAGAACCTCCATTCCTTTGGATGGGTTATGAACTCCATCCTGAYAAATGGACAGTA
CAGCCTATAGTACTGCCA

>45_MI

ACTCTTTGGCAACGACCCATAGTCAAAGTAAGAGTAGGGGGGCAGCTAACAGAAGCTCTA
tTAGATACAGGAGCAGATGATACAGTGTTYGAAGACCTAAATTTRCCAGGACCATGGAAA
CCAAAAATGATAATGGGAATTGGAGGTTTAGTCArAGTAAAACARTATGATAAyGTACCC
ATAGAAATTTGTGGACACAAGATTRTAGGTCCAGTGTTGATAGGACCTACGCCTGCCAAC
ATAATTGGAAGAAATACKATGACTCAGATTGGKTGTACTTTAAATTTTTGTGCAGAATTG
GAAAAAGAAGGAAAAATyTCAAAAATTGGGCCTGAAAATCCATACAATACTCCAGTRTTT
GCCATAAARAAGAAAAAyAGCACTAGATGGAGAAAAATAGTAGATTTTAGAGARCTTAAT
AAGAGAACTCAAGACTTCAGTGAAGTCCAATTAGGAATACCACATCCYGGAGGATTAAAr
AAGAAaAAATCAGTAACAGTACTGGATGTGGGTGATGCATATTTTTCAATTCCCTTAGAT
GAAGATTTYAGGAAGTATACTGCATtTACYATAcCtAgtAcAAACAATGAGACACCAGGG
ATTAGATATCAGTACAATGTTCTTCCACAGGGATGGAAAGGATCACCAGCAATATTTCAA
ARTAGCATGACAAAAATCTTAGAGCCTTTTAGAAAACAAAATCCAGACATAGTTATCTAT
CAGTACGTGGATGATTTGTATGTAGGATCTGACTTAGAAATAGGGCAACATAGArCAAAA
ATAGATGAACTAAGACAACATCTGTKGAAGTGGGGACTTTTCACACCAGAACAAAAACAT
CAGAAAGAACCTCCATTTCTTTGGATGGGTTATGAACTCCATCCTGATAARTGGACAGTA
CAGCCTATAAAGCTGCCA

>46_MI

ACTCTTTGGCAACGACCAGTAATCGAAGTAAAAGTAGGGGGGCAAATAAGGGAAGCTCTA
CTAGATACAGGAGCAGATGATACArTATTCGAAGACATAGAGTTGCCAGGAAGATGGAAA
CCAAAAATGATAGGGGGAATTGGGGGTTTTCTCARAGTAAAAGAGTATGATCAGGTACCC
ATAGAAATTTGTGGACACGAAATtATAGGTACAGTGTTAGTAGGACCTACACCTGTCAAC
GTAATTGGAAGAAATCTGATGACTCRGCTTGGCTGCACTCTAAATTTTTGTGCAGAAATG
GAAAAGGAAGGAAAAATTTCAAAAATTGGGCCTGAAAATCCATACAATACTCCAGTATTT
GCCATAAAGAAAAAGAACAGTGATAGATGGAGAAAATTAGTAGATTTTAGAGAACTTAAT
AAGAGAACTCAAGACTTCTGGGAAGTACAATTAGGAATACCACATCCCGCRGGGTTACCA
ARGAAAAAATCAGTAACAGTACTAGATGTGGGTGATGCATATTTTTCAGTGCCCTTAGAT
GARAATTTCAGGAAGTATACTGCATTTACCATACCTAGTACAAACAAYGAGACACCAGGG
ATtAGATATCAGTACAATGTGCTTCCACAGGGATGGAAAGGAtcACCAGCAATATTTCAA
AGCAGCATGACAARGATCTTAGAACCTTTTAGAAAACAAAATCCAGRCATAATTATCTAT
CAATACGTGGATGATTTATATGTAGCATCAGACCTAGAAATAGGGCAGCATAGAACAAAA
ATAGAGGAGCTRAGACAACACCTGTTGAGGTGGGGATTCTTCACACCAGACCAAAAACAT
CAGAAAGAACCTCCATTCCTTTGGATGGGTTATGAACTTCATCCTGATAAATGGACAGTA
CAGCCTATAATGCTGCCA

>47_MI

ACTCTTTGGCAACGACCAGTCrTCACARTAAGGGTAGGAGGGCAGCTAAAGGAAGCTCTA

TTAGATACAGGAGCAGATGATACAATATTCGAAGAAyTGGATTTGCCAGGAAGATGGAAA

CCAAAAATAATAGGGGGAATTGgAGGTTTTATCAAAGTAAGACAGTATGATCAGATACCC

ATAGAAATCTTTGGACAGAAAGCTATAAGTACAGTATTAGTGGGACCTACACCTGTCAAC

ATAATTGGAAGAAATCTgATGACTAAGATTGGCTGCACTTTAAATTTTTGTGCAGAACTG

GAAAAGGAGGGAAAAATTTCAAAAATTGGGCCTGAAAAYCCATACAATACTCCAGTATTT

GTCATAAAGAAAAAAGACAGTACTAAATGGAGAAAATTAGTAGATTTTAGGGAACTTAAT

AAAAGAACTCAAGACTTCTGGGAGGTCCAGTTAGGGATACCACATCCCGCGGGGTTAAAA

AAGAAAAAATCAGTAACAGTrCTGGATGTAGGTGATGCATATTTTTCAGTGCCCTTAGAT

GAARACTTYAGGAAGTATACTGCATTcACcATACCTAGTACAAACAATGAGACACCAGGa

ATTAGATATCAGTACAATGTGCTTCCACAGGGATGGAAAGGATCACCAGCAATATTCCAA

TGTAGCATGACAAAAATCTTAGAGCCTTTTAGAAAACAAAATCCAGACATAGTTATCTAT

CARTATGTAGATGACCTGTATGTAGGATCTGACTTAGAAATAGGGCAACATAGArCAAAA

GTAGAAGAACTAAGAGAACATCTGTGGAAGTGGGGATTTTACACACCAGACAAAAAACAT

CAGAAAGAACCTCCATTTCGTTGGATGGGCTATGAACTCCATCCTGATAAATGgACAGTA

CAGCCTATAATGCTGCCA

>48_MI

ACTCTTTGGCAACGACCCCTCGTCACAATAAAGGTAGGGGGGCAACAAAAGGAAGCTCTR

TTAGATACAGGAgCAGATGATACAGTATTAGAAGAaATGAATTTGCcAGGaAGATGGAAA

CCAAAAATGATAGGGGGAATTGgAGGTTTTATTAAAGTAAgAcAGTATGATCAAATACTC

ATAGAAATTTGTGGACATAAAGCAATAGGTACAGTATTAATAGGGCCTACACCTGTCAAC

ATAATTGGAAGAAATCTGTTGACTCAGATTGGTTGCACTTTAAATTTTTGTACAGAAATG

GAAAAGGAAGGGAAAATTTCAAAAATTGGGCCTGAGAATCCATATAATACTCCAGTATTT

GCCATAAAGAAAAAGAACAGTAmTAGATGGAGAAAATTAGTGGATTTCAGAGAGCTTAAC

AAGAGAACTCAAGACTTCTGGGAAGTTCAATTAGGAATCCCACATCCCTCAGGGCTACCA

AAGAAAAAATCAGTAACAGTACTGGATGTGGGTGATGCATATTTTTCAATTCCCTTAGAC

AAAGAGTTCAGGAAGTATACTGCATTtACcATACCTAGTATAAACAATGAGACACCAGGA

CTtAGATATCAGTATAATGTGCTTCCACAGGGGTGGAAAGGATCACCAGCAATATTCCAA

AgTAGCATGACAAGAATCTTAGAGCCTTTTAGAAAACAAAATCCAGAATTTATTATCTAT

CAATACGTGGATGATTTGTATGTGGCATCTGACTTAGAAATAGGACAGCATAGAGCAAAA

ATAGAGGAACTGAGACAGCATCTGTTrAgATGGGGATTATTCACACCAGACGAAAAACAT

CAGAAAGAACCTCCCTTCCTTTGGATGGGTTACGAACTCCATCCTGATAAATGGACAGTA

CAGCCTATAGTACTGCCA

>49_MI

ACTCTTtGGCAACGACCCCTAGTCACAATAAAGGTAGGGGGacAGCTAAAGGAAGCTCTA

TTAGATACAGGAGCAgATGATACAGTATTAGAAGAcATAAATTTGCCAGGAAAATGGAAA

CCAAAAATGATAGGGGGAATTGGAGGTTTTATCAaAGTAAAACAGTATGAGGACATACTC

ATAGAAATTTGTGGACACAAGGCTATAGGTACAGTGTTGRTAGGACCTACACCTGTCAAT

ATAATTGGAAGAAATATGTTGACTCAGATTGGCTGTACTTTAAATTTTTGTACAGAATTG

GAAAAGGACGGAAAAATCTCAAGAATTGGGCCTGAAAATCCATATAATACTCCAGTGTTT

GCtATAAAGAAAAAAGACAGTACTAAATGGAGAAAAATAGTAGATTTCAGAGAACTTAAT

AAGAAAACTCAAGACTTCTGGGAAGTTCAATTAGGAATACCACATCCCGCrGGGTTAAAA

AAGAACAAGTCAGTAACAGTACTGGATGTGGGYGATGCATATTTTTCAGTTCCCTTAGAT

AAAGAATTCAGGAAGTATACTGCATTtACCATACCTAGYACAAACAATGAGACACCAGGG

ATTAGGTATCAGTACAATGTGCTTCCACAAGGATGGAAgGGgTCACCAGCAATATTTCAA

AGCAGCATGACAAAAATCTTAAAGCCTTTTAGAGAACAAAATCCAGACATAGTTATCTAT

CAATAYGTGGATGATTTGTATGTAGGATCTGACTTAGAAATAGAACAGCATAGAACAAAA

ATAGAGGAATTGAGACAACATCTGTGGGCGTGGGGATTTTACACACCAGACAAGAAACAT

CAGAAAGAACATCCATTCCrTTGGATGGGTTATGAACTCCATCCTGATAAATGGACAGTG

CAGCCTGTAACACTGCCA

>50_MI

ACTCTTTGGCAACGACCCTTCGTCACAATAAAGGTAGCGGGGCAWGTAatAGAAGCTCTA

TTAGATACAGGAGCAGATGATACAGTATTAGAAGAAATAGAGTTGCCAGGAAGATGGAAA

CCAAAAATAATAGGGGGAATTGGAGGTTTTGTCAArGTAAGACAGTATGATCACATACCC

ATAGAAATTTGCGGACATmGAGTCATGAGTACAGTGTTGGTAGGGCCTACACCTTCCAAC

ATAATTGGAAGAAATTTrATGACTCAGATTGGmTGTACTCTAAATTTTTGTACAGARTTG

GAASAGGCAGGAAARATTTCAAAAATTGGGCCTGAGAATCCATACAATACTCCAGTRTTT

GCCATAAAGAAAAAGAACAGTGATAAATGGAGAAAATTAGTAGATTTCAGAGAACTTAAT

AAGAGAACTCAAGACTTCTGGGAAATTCAATTAGGAATACCACATCCAGCAGGGTTAAAA

AATAACAAATCAGTAACAGTACTGGATGTGGGTGATGCATATTTTTCAATTCCCTTAGAC

GAAGACTTCAGGAAGTATACtgCATTTACCATACCTAGTACAAACAATGAGACACCAGGG

ATCAGATATCAGTACAATGTGCTTCCACaGGGATGGAAAGGATCACCAGCAATATTCCAA

TAYAGCATGACAAAAATTTTAGAGCCTTTCAGAAAGCAAAATCCAGACATAGAGATCTGT

CAATACGTGGATGATTTGTaCGTAGGATCTGATTTACCAATAGAGCAGCATAGAGCAAAA

GTAGAGGAACTGAGACAACATCTRTGGAAGTGGGGATTTTACACACCAGATAAyAAACAT

CAGAArGAACCCCCATTCCTTTGGATGGGTTATGAACTCCATCCTGATAAATGGACAGTA

CaGCCTATAGTGCTGCCA

>51_MI

ACTCTTTgGCAACGACCCTTCGTCAAyATAAAGATAGGGGGGCAAMTRATAGAAGCTCTA

TTAGATACAGGAGCAGATGAyACAGTATTAGAAGAAATRAATTTACCAGGAAAATGGAAA

CCAAAAATAATAGGGGGAATTGGAGGTTTTGTCAAAGTAAGACAGTATGATCAGGTACCT

ATAGAATTCTRTGGACATAAAACTACAACTACAGTATTRRTAGGGCCTACACCATGCAAC

ATAATTGGAAGAAATCTGATGACTCAGCTTGGAGTAACTCTAAATTTTTGTACAGAATTG

GAAAAGGACGGAAAAATTTCAAAAATTGGGCCCGAGAATCCATACAAYACTCCAGTATTT

GCCATAAAGAAAAAGGACAGTACTAAATGGAGAAAACTAATGGACYTKAGAGAACTTAAT

AAGAGAACTCAAGACTTCTGGGAAGTTCAATTAGGAATACCACATCCCGGAGGGCTAAAA

AAGAAAAAATCAGTAACAGTACTGGATGTGGGTGATGCATATTTTTCARTTCCCTTAGAT

GAAGAATTCAGGAAATATACTGCATTTACCATACCTAGTCTAAACAATGAGACACCAGGG

ATTAGATATCAGTACAATGTGCTTCCACAAGGATGGAAAGGATCACCAGCAATATTCCAA

TGTAGCATGACAAAAATCTTAGAGYCTTTTAGAAAACAAAATCCAGACATAGTCATCTAT

CAGTATGTGGATGATTTGTATGTAGGATCTGACTTAGAWATAGAGCAGCATAGAAYAAAA

ATAGAGGAACTGAGACAACATCTATGGGCGTGGGGGTTTTACACACCAGACAAAAAACAT

CAGCAAGAAyATCCATTCCGTTGGATGGGTTATGAACTCCATCCTGATAAATGGACAGTA

CAGCCTATAGAACTGCCA

>52_MI

ACTCTTTGGCAACGACCAGTAGTCAAAATAAARGTAGGGGGGCAAATAAGGGAAGCTCTA

TTAGATACAGGAGCAGATGATACAGTATTAGAAGACATAGAGTTGCCAGGAAGATGGAAA

CCAAAAwTRATAGGaGGAATTGGAGGTTTTGTCAAAGTAAAACAGTATGATCAGGTACGC

ATAGAAATyTGTGGACATGAAGTTATAGGTwCAGTATTAGTAGGACCTACACCTTCTAAC

ATAATTGGAAGAAATyTGCTGACTCAGATTGGCTGCACTTTAAATTTTTGTGMAGAATTg

GAGAAGGAAGGAAAAATTTCAAAAATTGGGCCTGAAAATCCATACAATACTCCAGTATTT

GCCATAAAGAAAAAGRATAGTACTAAATGGAGAAAATTAATGGATTTCAGAGAACTTAAT

AARAGAACTCAAGATTTCTGGGAAGTTCAATTAGGRATACCACATCCCGCAGGGTTAAAA

AAGAACAGATCAGTAACAGTACTAGATGTGGGAGATGCATATTTTTCArTTCCCTTAGAT

GAAGACTTCAGRAAGTACACAGCATTTACCATACCTAGTAyAAACAATGAGACACCAGGG

ATTAGATATCAGTACAATGTGCTGCCACAGGGatGGAAAGGATCACCAGCAATATTTmAA

AGYAGCATGACAAAAATYTTAGAGCCTTTTAGAAAACAAAATCCAGACATAGTTATCTAT

CAGTAyATGGATGATTTGTATGTAGGATCTGACTTrGAAATAGGACArCACAGAGCAAAA

ATAGAGGAGCTAAGACAACATCTGTTArArTGGGGATTTWACACACCAGACAAAAAACAT
CAGAAAGARCCTCCATTCCTTTGGATGGGTTATGAACTCCATCCTGATAAatGGACAGTA
CAGCCTATAGTGCTGCCA
>53_MI
ACTCTTTGGCAGCGACCCCTAGTCACAATAAACATAGGGGGGCAACAAACGGAAGCTmTA
CTAGATACAGGAGCAGATGATACAGTATTCArmGACATAACTTTACCAGGAAGATGGAAA
CCAAAAATGATAGGGGGACTTGGAGGTTTTATCAAAGTAAAAGAGTATGATAACrTACCC
ATAGAAATTTGTGGACACAGGGCAATArGTACAGTGTTGATAGGACCTACACCTGTCAAC
ATAATTGGAAGAAATCTGWTGACTCAACTTGGCTGTACTTTAAATTTTTGTACAGAAATG
GAAAAGGAAGGAAAAATTTCAAAAATTGGGCCTGAAAATCCATACAATACTCCAATATTT
GCCATAAAGAAAAAGGACAGTACTAAGTGGAGAAAATTAGTAGATTTCAGAGARCTTAAT
AGRAGAACTCAAGACTTCTGGGAAGTTCAATTAGGGATACCACATCCCGCAGGGTTAAAA
AAGAAAAAATCAGTAACAGTATTGGATGTGGGTGATGCCTACTTTTCrGTTCCCTTAGAT
AAAGAATTCAGGAAGTATACTGCATTtACcATACCKAGTACAAACAATGCGACACCAGGG
GTTAGATATCAGTACAATGTGCTTCCACAGGGATGGAAAGGATCACCAGCAATATTCCAA
AGTAGCATGACAAAAATCTTAGAGCCTTTTAGAAAACAAAATCCAGACATAGTTATCTAT
CAATATGTGGATGATTTGTATGTAGGATCTGACTTAGAAATAGGGCAGCATAGAACAAAA
GTAGAGGAGCTGAGACAGCATCTGTTGAAGTGGGGATTTACCACACCAGACAAAAAACAT
CAGAAAGAACCTCCATTCCTTTGGATGGGTTATGAACTCCATCCTGATAAATGGACAGTA
CAGCCTATAACACTGCCA
>54_MI
ACTCTTTGGCAACGACCCCTCGTCAAAATAAAGATAGGGGGGCAACTAAGGGAaGCTCTA
TTrGATACAGGAGCAGATGATACAGTrTTAGAAGAAATAAATTTRCCAGGAAAATGGAAA
CCAAAAATGATAGGRGGAATTGGAGGTTTTATCAAAGTAAGACAGTATGAyCAGGTACTT
GTAGAAATYTGYGGACATAAAGCTATAGGTACAGTrTTAGTAGGGCCTACACCTGTCAAC
ATAATTGGRAGAAATCTGTTGACTCAGATTGGCTGYACTTTAAATTTTTGTACAGAAATG
GAAAAGGAAGGAAAAATTTCAAAAATTGGGCCTGAAAATCCTTACAATACTCCAGTATTT
GCCATAAAGAAAAAGGACAGTACTAAATGGAGAAAAYTAGTGGATTTCAGAGAACTTAAT
AAGAGAACTCArGACTTCTGGGAAGTTCAATTAGGAATACCACATCCYGCAGGGTTAAAA
AAGAAAAAATCAGTTACAGTACTGGATGTGGGTGATGCATATTTTTCAGTrCCCTTAGAT
GAAGACTTCAGGAAATATaCTGCATTTACCATACCTAGTATAAAYAATGARACACCAGGR
ATTAGATATCAGTACAATGTRCTTCCACAGGGATGGAARGGATCACCAGCAATATTCCAA
AGYAGCATGACAAGAATCTTAGAGCCTTTTAGAGAGCAAAATCCAGACATAGTTATCTAT

CAATACATGGATGACTTGTATGTAGGATCTGACTTAGAAATAGGGCAGCATAGRRCAAAA
ATAGARGAGCTGAGACAACATCTGTTGAGGTGGGGATTTACCACACCAGACAAAAARCAT
CAGAAAGATCCTCCATTCCTTTGGATGGGTTATGAACTCcATCCTGATAAATGGACAGTA
CAGCCTATAGTACTGCCA
>55_MI
ACTCTTtGGCAACGACCCtTcGTCCCARTAARAGTAGGGGGGCAACAAATAGAAGCTCTA
TTAGATACAGGAGCAGATGATACAGTATTACAAGAYATAAATTTACCAGGAAGATGGAAA
CCAAAAATAATAGGGGGAATTGGAGGTTTTGCCAGAGTAAGAsAGTATGATCAGGTAWCT
GTAGATATCTGTGGACATAAAGCTATAGGTTCAGTATTAGTAGGACCTACACCTGCCAAC
ATAGTTGGAAGAAATCTGTTGTCTCAGATTGGCTGTACTTTAAATTTTTGTGCAGAATTG
GAAAAGGACGGGAAAATTTCAAAAATTGGGCCTGAAAATCCTTACAATACTCCAGTATTT
GCCATAAAGAAAAAGAACAGTGATAAATGGAGAAAATTAGTAGATTTCAGAGAACTTAAT
AAGAGAACTCAAGATTTCTGGGAAGTTCAATTAGGAATACCACATCCCGCAGGGTTAARM
AAGAAAAAATCAGTAACARTACTGGATGTGGGTGATGCATATTTCTCAGTTCCCTTAGAT
AAAGAATTCAGGAAGTATACTGCATTcACCATACCTAGTACAAACAATGAGACACCAGGG
ATAAGATATCAATACAATGTACTTCCACAGGGATGGAAAGGATCACCAGCAATATTCCAA
AGTAGCATGACAAAAATCTTAGAGCCTTTTAGAAAACAAAATCCAGAAATAGTTATCTGT
CAATACGTGGATGATTTRTATRTAGGATCTGACTTAGAAATAGGGCAGCATAGAACAAAA
ATAGAGGAACTGAGACAAYATCTGTGGAAGTGGGGGTTTTACACACCAGACAAAAAACAT
CAGAAAGAACCTCCATTTCTTTGGATGGGYTATGAGCTCCATCCTGATAAATGGACAGTA
CAGCCTATAATGCTGCCA
>56_MI
ACTCTTTGGaAACGACCCATCATCACAATAAAGATAGGAGGGCAACAAATAGAAGYTCTA
TTAGATACAGGAGCAGATGATACAGTAATAGAAsACCTAGATTTACCAGGAAGATGGACA
CCAAAAATAATAGGGGGAATTGGAGGTTTTGTAAGAGTAAAACAATATGAGCAAGTACCC
ATAGAAATCTGTGGGCATAAAGTGTTAAGTACAGTATTAGTAGGACCTACACCTGCCAAC
ATAGTTGGAAGAaAyTTRATGACTCARATTGGCTGCACTTTAAATTTTTGTGCAGAATTG
GAAAAGGAYGGAAAAATWTCAAAAATTGGGCCTGAAAACCCATACAATACTCCAGTATTT
GCTATAAAGAAGAAAAATAGTGATAAATGGAGACAATTATCAGATTTCAGAGAACTTAAT
AAGAGAACTCAAGACTTCTGGGAAGTCCAGTTAGGAATACCACATCCTGCAGGGTTAGAG
AAGAAAAAATCAGTAACAGTACTGGATGTGGGTGATGCATATTTTTCAATTCCCTTAGAT
GAAAACTTCAGGAAGTATACTGCATTYACCATACCTAGTAGAAACAATGARACACCAGGG
ATTAGATATCAGTACAATGTGCTTCCACAGGGATGGAAAGGATCACCAGCAATATTCCAA

GATAGCATGACAAAAATTTTAGAGCCTTTTAGAAAACAAAATCCAGACATAAYTATTTGT

CAATATGTGGATGATTTGTATATAGCATCTGATTTAGAAATAGGRCAGCACAGAGCAAAA

GTAGCRGAACTRAGACAGTTTCTGTGGAAGTGGGGATTTTACACACCAGACAAcAARCAT

CAGACAGAACCYCCATTCCATTGGATGGGTTATGAACTCCATCCTGATAAATGGACAGTA

CAGCCTATAGTGCTGCCA

>57_MI

ACTCTTTGGCAACGACCCCTCGTCACAATAAAGATAGGGGGGCAACTAAAGGAAGCTCTA

TTAGAYACAGGAGCAGATGATACAGTATTAGAAGAAATGAATTTGCCAGGAAGATGGAAA

CCAAAAATGATAGGGGGAATTGGAGGTTTTATCAAAGTAAGRCAGTATGATCAGRTACCC

ATAGAAATCTGTGGGCATAAAGCTATAGGTACAGTATTARTAGGACCTACACCTGTCAAC

ATAATTGGAAGAAATTTGTTGACTCAGATTGGCTGCACCTTAAATTTTTGTGCAGAATTG

GAAAAGGAAGGAAAGATTTCAAAAATTGGGCCTGAAAATCCATACAATACTCCAGTATTT

GCCATAAAGAAAAAACACAATAATAGATGGAGAAAAwTAGTAGATTTCAGAGAACTTAAT

AAGAGGACTCAAGACTTCTGGGAAGTCCAATTAGGAATACCACACCCAGCAGGATTGAAA

AAATACAAATCAGTAACAGTACTAGATGTGGGTGATGCATATTTCTCARTTCCCTTAGAT

AAAGACTTCAGAAAGTATACTGCATTYACcATACCTAGTACAAACAATGAGACACCAGGG

ATTAGATATCAGTACAATGTRCTTCCACAGGGATGGAAgGGATCAgCAGCRATATTCCAA

AGYAGCATGACAAAAATCTTAGAGCCTTTTAGAAAACAAAATCCAGrCATArTTATCTAY

CAGTACACGGATGATTTGTATGTAGGATCTGACTTAGAAATAGGGCARCATAGAACAAAA

ATAGAGGAACTGAGGCAACACTTGTTrAAGTGGGGGTTATTCACACCAGACsAAAAACAT

CAGAAAGAACCTCCATTCCTTTGGATGGGGTATGAACTCCATCCTGATAAATGGACAGTA

CAACCTATAAAGCTGCCc

>58_MI

ACTCTTTGGCARCGACCCATAGTCACAATAAAAGTAgGGGGacAgATAAGrGAAGCTCTA

TTAGAyACAGGAgcAGATGATACAGTATTAGAAGACATAAATTTRCCAGGAaAATGGAAA

CCAAAAATgATAGGGGGAATTGgAGGTTTTGTCAAAGTAaAACAgTATGATAACATACCC

ATAGAAATTTGTGGACayaARGTTATAGGTTCAGTGTTGGTAGGACCCACGCCTGccAAC

ATAATTGGAAGAAATCTRATGACTCAGCTTGGATGTACTTTAAATTTTTGTTTAGATTTG

GAAAAGGAAGGAAAAATTTCAAAAATTGGGCCTGAAAATCCATACAATACTCCAATATTT

GCtATAAAAAAGAAAAACAGTGATAGATGGAGAAAAGTAGTAGATTTCAGAGAACTTAAT

AAAAGAACTCAAGATTTTTGGGAAGTTCAATTAGGAATACCGCATCCGGCAGGGTTAGAA

AAGAAAAAGTCAGCAACGGTAMTAGATGTGGGGGATGCATATTTTTCAATtCCCYTAGAT

GAAGATTTCAGGAAGTACACTGCATTcACcATACCTAGTACCAACAATGCAACACCAGGA

ATTAGGTACCAGTACAATGTGCTCCCACAAGGATGGAAAGGATCACCAGCAATATTCCAA
AGTAGCATGACAAAAATCTTAGATCCCTTTAGRRCAAAAAATCCAGACATARTTATCTAC
CAATACGTGGATGATTTGTATGTAGTrTCTGACTTAGAAATAGGACAGCATAGAACAAAA
ATAGAAGAGTTAAGAGARCATTTACTrAAATGGGGATTATTTACACCAGAMCAAAAGCAT
CAAAAAGAACCCCCATTCCATTGGATGGGGTATGAACTCCATCCTGATAAATGGACAGTG
CAACCTATACAaTTGCCA
>59_MI
ACTCTTtGGCAACGACCCCTCGTCrCAATAAGAGTAGGdGGACAAyTAAAGGAAGCTCTA
TTAgATACAGGAGCAGATGATACAGTATTAGAAGACATAAATTTGCCAGGAAAATGGAAA
CCAAAAATGATAGGGGGAATTGGAGGYTTTATCAAAGTAARACARTATGATCAGATACTC
GTAGAAATCTGTGGACAbAAAGCTATAGrTACAGTRTTAGTAGGACCYACACCTGTCAAC
ATAATTGGAAGAAATCTGTTGACrCAAATyGGkTGCACTCTAAAYTTTTGTgcAGAATTG
GAAGAsGATGGAAAAATTTCAAAAATTGGGCCTGAAAATCCATACAATACTCCAGTATTT
GCCATAAAGAAAAAGAACAGTACTAAATGGAGAAAAATAGTAGATTTCAGAGAACTTAAT
AAAAGAACCCAAGATTTTTGGGAGGTTCAATTAGGAATACCRCATCCKGGAGGGTTAAAA
MAGAAAAAATCAGTAACAGTyYTGGATGTGGGAGAyGCATACTTTTCAATTCCCTTAGAT
AAGGAGTTCAGGAAGTACACTGCATTcACCATACCTAGTCTCAATAATGAGACACCAGga
gTTAGGTACCAATACAATGTGCTTCCACAAGGATGGAAAGGgTCACCAGCAATATTCCAr
AATAGCATGACAAGAATCTTAGAGCCTTTTAGAAAACAAAATCCAGACTTTrTTATCTAT
CAATACGTGGATGATTTATATGTAGCATCAGACTTAGAAATAGGGCAGCACAGAGCAAAA
ATCGATGAGTTAAGAGAATATCTATGGAAATGGGGATTTTACACACCAGACAAAAAAYAT
CAGAAAGAACCTCCATTYCTTTGGATGGGTTATGAACTCCATCCTGATAAATGGACAGTA
CAGCCTATCACGCTGCCA
>60_MI
ACTCTTTGGCAACGACCCTTAGTCACAATAAAGATAGGagGGcaACTAAAGGAAGCTCTA
TTAGATACAGGAGCAGATGATACAGTATTAGAAGATTTagagTtrCcaGGAAAATGGAAA
CCAAAAATGATAGGGGGRATTGGAGGTTTTATCAAAGTAAAACAGTATGAGAACATACCC
ATAGAAATTGAGGGACACAGGGCTATAGGTACAGTATTAGTAGGGCCTACACCTGTCAAC
ATAATTGGAAGAAATCTGTTGACTCAGATTGGCTGCACTTTAAATTTTTGTACAGAAATG
GAAAAGGAAGGAAAAATTTCAAAAATTGGGCCTGAAAATCCATACAATACTCCAATATTT
gCCATAARGAAAAAGGACAGTACTMAATGGAGAAAATTAGTAGATTTCAGAGAACTTAAT
AAAAGAACTCAAGACTTCTGGGAAGTTCAATTAGGAATACCACATCCCGCAGGGTTAAAr
AAGAArAAATCAGTAACAGTACTAGATGTGGGTGATGCATATTTTTCAGTTCCCTTAGAT

AAGGACTTTAGGAAGTACACTGCATTtACCATACCTAGTACAAACAATGAGACACCAGGG
gTTAGGTATCAGTACAATGTGCTTCCACAAGGATGGAAAGGATCACCAGCAATATTCCAA
AGTAGCATGACAAAAATCTTAGAGCCTTTTAGAAAACAAAATCCAGACATAGTAATCTAT
CAATACGTGGATGATTTGTATGTAGGATCTGACTTAGAAATAGAGAAGCACAGAACAAAA
ATAGAGGAGCTAAGACAACATCTGTYGRAGTGGGGGTTATTTACACCAGACAAAAAGCAT
CAGAAAGAACCTCCATTCCTTTGGATGGGTTATGAACTCCATCCTGATAAATGGACAGTA
CAGCCTATAGTGCTGCCA
>61_MI
ACTCTTTGGCAACGACCAGTAGTCACAGTAAAGGtAGGGGGACAGCTAARGGAAGCTCTA
TTAGATACAGGAGCAGATGATACAGTATTAGAAGAYATAAATTTGCCAGGAARATGGARA
CCAAAAATGATAGGGGRAATTGGAGGTTTTATCAAAGTAAAACAGTATGATGACATACTC
ATAGAAATTTGTGGACACAAGGCTATAGGTACAGTATTGGTAGGACCCACACCTGTCAAC
ATAATTGGAAGAAATATGTTAACTCAGATTGGTTGTACTTTAAATTTTTGTACAGAAATG
GaAAAGGAAGGGAAGATYTCAAAAATTGGGCCTGAAAATCCATACAATACTCCAATATTT
GCCATAAAGAAAAAAGACAGTACTAAATGGAGAAAATTAGTRGATTTCAGAGARCTTAAT
AArAGAACTCAAGACTTCTGGGAAGTTCAATTAGGAATACCACATCCTGSAGGGTTAAAA
AAGAACAAGTCWGTAACARTACTGGATGTRGGTGATGCATATTTTTCAGTTCCCTTAGAT
GAAGAATTTAGGAAGTATACTGCATTTACcATACCTAGTAYAAACAATGAGACACCAGGG
GTTAGaTAYCAGTACAATGTGCTTCCACArGGATGgaAAGGATCaCCAGCAATATTCCAA
AGTAGCATGACAAAAATCTTAGAGCCTTTTAGAAAACAAAATCCAGAAATAGTTATCTAT
CAATACGTGGATGATTTGTATGTAGGATCTGACTTGGAAATAGGGCAGCATAGAGCAAAA
ATAGAGGAACTGAGAcAACATCTGTTGGCGTGGGGATTTACCACACCAGACAAAAAACAT
CAGAAAGAACACCCATTCCTTTGGATGGGTTATGAACTCCATCCTGACAAATGGACAGTA
CAGCCTATAAAGCTGCCA
>62_MI
ACTcTtTGGCAACGACCCCGCGTCACAGTAAAGATAGGAGGGCAGCTAARGGAAGCTCTA
TTAGACACAGGAGCAGATGATACAATATTYGAAGACTTAAATTTGCCAGGAAGATGGAAA
CCAAAAGTGGTAGGGGGAATTGGAGGTTTTATCAAAGTAAGACAGTATGATYACATACCC
ATAGAAATCTGTGRACATAAAACTATAGGTACAGTATTAGTAGGACCTACACCTGTYGAC
GTAATTGGAAGAAATGTGTTGTCTCAGATTGGTTGCACTTTAAATTTTTGTGCAGAACTA
GAAGAGGCAGGGAAAATTTCAAGAATTGGGCCGGATAATCCATACAATACTCCAATATTT
GCCATAAAGAAGAAAAACAGTACTAAATGGAGAAAATTAGTAGATTTTAGAGAACTTAAT
AAGAGAACTCAAGACTTCTGGGAAGTTCAGTTAGGAATACCACATCCYGCAGGGTTAAAA

AAGAAAAAATCAGTAACAGTACTGGATGTAGGTGATGCATATTTTTCAATTCCYTTAGAT

GAAGACTTCAGGAAGTATACTgCATTCACaATACCTAGYATAAACAAYGAAACACCAGGG

ATtAGATATCAGtACAATGTGCTGCCACAGGGATGGAAAGGATCACCAGCAATATTCCAA

GATAGCATGACAAAAATCTTAGAGCCTTTTAGAAAyAAGAATCCAGACATGGTTATCTAT

CAGTACATGGATGATTTGTATGTAGGATCTGACTTAGAAATAGAGCAACATAGAGCAAAA

ATAGAGGAACTGAGAGAACATCTGTGGAGGTGGGGGTTTTACACACCAGACArAAAACAT

CAGAAAGAACCTCCATTCCTTTGGATGGGTTATGAACTtCATCCAGATAAATGGACAGTg

CAGCCTATAGTGCTGCCA

>63_MI

WCTCTTTGGCAACGACcaGTARTTAAAATAAAGGTAGGGGGGCAAMTGACAGaAGCTCTA

TTAGATACAGGAGCAGATGATACAGTATTCGAAAACATAGAGTTGCCAGGAAARTGGAGA

CCAAAAATWATAGGGGGAATTGGAGGTTTTGTCAAAGTAAAACAGTATGATCACGTACCC

ATAGAAATCTGTGGACATAAAACTTTAACTACAGTATTAGTAGGGCCTACACCTTGCAAC

ATAGTTGGAAGAAATCTGATGACCCAGATTGGCTGCACTTTAAATTTTTGYACAGAAYTG

GAAAAGGAAGGAAAAATTTCAAAAATTGGGCCTGAAAATCCATACAATACTCCAATATTT

GCAATAAAGAAAAAGAACAGTAMTAGATGGAGAAAATTAATGGATTTCAGAGAACTTAAC

AAAAGAACTCAAGACTTCTGGGAAGTTCAATTAGGAMTACCACATCCCGCAGGGYTAaAA

AAGAAMAAATCAGTAACAGTACTAGATGTGGGTGATGCATATTTTTCAATTCCCTTAGAT

RAAGAATTCAGGAAATAcACTGCATTTACCATACCTAGTATAAACAATGAGACACCAGGG

ATTAGATATCAGTACAATGTACTTCCACArGGATGGAAAGGATCACCAGCAATATTCCAG

AGTAGCATGCTAAAAATCTTAGAGCCTTTTAGAARRCAAAATCCAAACATAGTGATCTAy

CARTACGTGGATGATTTGTATGTAGGATCTGACTTAGAAATAGGGCAGCATAGAATAAAA

RTAGAGGAGCTGAGACAACATCTGTTGAAATGGGGATTGTTTACACCAGACGAAAAACAT

CAGAAAGAGCCCCCATTCCATTGGATGGGGTATGAACTCCATCCTGATAAATGGACAGTA

CAGCCTATACAATTGCCg

>64_MI

ACTCTttGGCAACGACCCATAGTCACAGTAAAGATAGGGGGACAGCTAAGGGAAGCTCTA

TTAGATACAGGAGCAGATGATACAGTAATAGAAGACATAAATTTGCCAGGAAAATGGAAA

CCAAAATTGATAGTGGGAATTGGAGGTTTTGTCAAAGTAAAACAGTATGATAACATATCC

ATAGACATTTGTGGTCACAAGGCTATAGGTACAGTATTGGTAGGRCCTACGCCTTCCAAC

ATAATTGGAAGAAATATGTTGACTCAGATTGGTTGTACTTTAAATTTTTGTACAGAAATG

GAAAAGGAAGGAAAAAtTTcAAAGATTGGGCCTGAAAATCCATATAATACTCCAGTATTT

GCCATAAAGAAAAAAMATAGTACTAGATGGAGGAAATTAGTAGATTTCAGAGAACTAAAT

AAGAAAACTCAAGATTTTTGGGAGGTTCAATTAGGaATACCGCATCCTGCAGGGTTAAAA
AAGAAAAAGTCAGTAACTGTACTGGATGTAGGTGATGCATATTTTTCAGTTCCCTTGGAT
AAAGAATTCAGGAAGTACACagCATTcACCATACCTAgTACAAACAATGAGACACCAGGG
ATTAGATATcAGTACAATGTGCTTCCACAAGGATGGAAgGGatCACCAGCAATATTTCAA
TATAGCATGACAAAAATCTTAGAACCCTTCAGAACAAAAAACCCAGACATAGTTATCTAT
CAATACGTGGATGATTTGTATGTAGGGTCTGACTTAGAAATAGGGCAACATAGAGCAAAA
ATAGAGGAGTTAAGAAATCATCTGCTGAAATGGGGATTTATTACACCAGACGAGAAGCAT
CAAAAGGAACCCCCATTCCTTTGGATGGGGTATGAACTCCATCCTGATAAATGGACAGTG
CAGCCTATACAATTGCCA
>65_MI
ACTCTTTGGcAACGACCCATTGTCCCAATAAAGATAGGGGGGCAACTAATGGAAGCTCTA
ATAGATACAGGAGCAGATGATACAGTATTTGAAGAAATGAATTTGCCTGGAAAATGGAAG
CCAAAATTGATAGGGGGAATTGGAGGTTTTSTCAGAGTAAGACAGTATGAACAGATACCA
ATAGAAATCTGCGGACATAAAGTKATAGGTACAGTATTAGTAGGACCTACACCTGCCAAC
GTAATTGGAAGAAAtcTGTTGACTCAGCTTGGCTGCACTTTAAATTTTTGCACAGAATTR
GAAGAGGACGGAAAAATTTCAAAAATTGGGCCTGAAAATCCATACAATACTCCAATATTT
GCTATAAAGAAGAARRATAGTACTAAATGGAGAAAATTAGTAGATTTTAGAGAACTTAAT
AAGAGAACTCAAGACTTCTGGGAAGTTCAATTRGGAATACCACATCCCGCAGGGTTRAAA
AGGAGCAAATCAGTAACAGTATTGGATRTAGGTGATGCATATTTCTCAGTTCCCTTAGAC
AAAGACTTCAGGAAGTATACTGCATTTACCATACCTAGTATAAACAATGAGACACCAGGA
ATTAGATATCAGTACAATGTGCTTCCACAAGGATGGAAGGGATCACCAGCAATTTTCCAA
AGTAGCATGACAAAAATCTTAGAGCCTTTTAGAAAACAGAATCCAGGAATAATTATTTAy
CAATACGTGGATGACTTGTATGTAGCATCTGACTTAGAAATAGAGCAACATAGAACAAAA
ATAGAGGAACTGAgACAACATCTGTYRAAGTGGGGATTTTACACACCAGACAAAAAGCAT
CAGAAAGARCCCCCATTCCTYTGGATGGGTTATGAACTYCATCCTGAKAAATGGACAGTR
CAGCCTATAGTGCTGCCA
>66_MI
ACTCTTTGGCAACGACCTMTAGTtRCTATAAAAATAGGGGGASAGMTAARGGAAGCTCTA
TTAGATACAGGAGCAGATGATACAgTaTTAGAAGACATAAATTTGCCAGGAARATGGAAA
CCAAAAATGATAGGGGGAATTGGAGGTTTTRTCAAAGTAARAGAGTATGAGAACATAMCC
aTAgAAATTTGCGGACACAAGGCTATAGGTACAGTGTTAATAGGACCTACGCCTGTCAAC
RTAATTGGAAGAAATATGTTGACTCArATTGGTTGTACCTTAAATTTTTGTACAGAAATG
GAAAAAGAAGGAAAAATTTCAARAATTGGGCCTGAAAATCCATACAATACTCCAGTATTT

GCTATAAAGAAAAARGACAGTACTAAATGGAGGAAATTAGTAGATTTCAGAGAACTTAAT
AAAAGAACTCAAGATTTTTGGGAGGTTCAATTAGGAATACCACAYCCTGCAGGGTTAAAA
AAGAACAAATCAGTAACAGTRCTGGATGTGGGGGATGCATATTTCTCAGTTCCCTTAGAT
AAGGAWTTCAGGAAGTACACTGCATTCACCATACCTAGTGTCAACAATGAGACACCAGGA
ATCAGGTACCAGTACAATGTGCTTCcACAAGGATGGAAAGGATCACCAGCAATATTTCAA
TGTAGYATGACAAARATCTTAGAGCCYTTCAGAGCAAAAAATCCAGAATTAGTTATCTAC
CAGTACGTGGATGATTTGTATGTAGGGTCTGACTTAGAAATAGAGCAGCATAGAGCAAAA
ATAGATGAGYTAAGAAATCATCTACTGAAATGGGGATTTTAYACACCAGACAAAAAACAT
CAAAAGGAACCCCCATTCCTTTGGATGGGGTATGAACTYCATCCTGATAAATGGACAGTG
CAGCCTATACAATTGCCA
>67_MI
ACTCTTTGGCAACGACCAGTAGTCAAAGTCAAGGTAGGGGGGCAGATGATrGAAGCTCTA
TTAGATACAgGaGCAGATAATACAgTCTtAGAAGGAATAGAGTTGCCAGGAAGATGGAAA
CCAAAATTGGCAGGGGGAATTGGAGGTTTTATCAAAGTAAGACAGTATGATCAGGTACCC
ATAGAAATCTGTGGACATGAAGCTATAGGTACAGTATTAGTAGGACCTACACCTGTCAAC
ATAATTGGAAGAGATCTGTTGACTCAGATTGGMTGCACTTTAAaTTTTTGTRCAGAACTa
GAAAAGGAAGGaAAAATTTCAAAAATTGGGCCTGARaATCCATACAATACTCCAGTATTT
GCCATAAAGAAAAAGAACAGTAMTAGATGGAGAAAATTAGTAGATTTCAGAGAACTTAAT
AAAAGAACTCAAGACTTCTGGGAAGTTCAATTAGGRATACCACATCCATCAGGGTTrAAA
CAGAAAAGATCAGTAACAGTACTAGATGTGGGTGATGCATATTTTTCAGTTCCCTTAGAT
GAAGACTTYAGGAAATACACTgCATTCACCATACCTAGTACAAACAATGAGACACCAGGA
ATTAGATATCAGTATAATGTGCTTCCACAAGGATGGAAAGgATCACCAGCAATATTCCAA
AGCAGCATGACAAAAATCTTAGAGCCTTTTAGAAAGCAAAATCCAGGCATAATTATATAT
CAATACGTGGATGATTTGTATGTAGGCTCTGACCTAGAAATAGGACAGCATAGAGCAAAA
ATAGAAGAGCTAAGACAACATTTGTGGARGTGGGGRTTTTAyACACCAGAYGAAAAACAT
CAGAAAGAACCTCCATTCCTTTGGATGGGTTATGAACTCCATCCTGATAAATGGACAGTA
CAGCCTATAATGCTGCCA
>68_MI
ACTCTTTGGCAACGACCCATCGTCACAATAAAGGTRGGGGGACAARTAAGRGAAGCTCTA
TTAGATACAGGAGCAGATGATACAGTATTAGAAGAAATAAATTTGCCAGGGAGATGGACG
CCAAAAATAATAGGGGGAATTGGAGGTTTTGTCAAAGTAAGACAGTATGATCAArTACCC
ATAGAAATYTGYGGACATAGAGTGATGGGTACAGTATTAGTAGGACCTACACCTAGCAAY
GTAATTGGAAGAAATGTGTTGACTAAGATTGGCTGCACTTTAAATTTTTGTGCAGACCTG

GAAAAGGAAGGAAAAATTTCAAAAATTGGGCCTGAGAATCCATACAATACTCCAGTATTT
GCCATAAAGAAAAAGAACAGTACTAGGTGGAGAAAAGTrGTAGACTTCAGAGAACTTAAY
AARAGAACTCAAGACTTTTGGGAAGTTCAATTAGGAATACCACATCCYGCWGGAATAAAA
AAGAAcAAATCAGTAACAGTACTGGATGTGGGTGATGCATATTTTTCAATTCCtTTAGAT
GAAGACTTCAGGAAGTACACTGCATTTACCATACCTAGTACAAACAATGAGACACCAGGG
ATTAGATATCAGTACAATGTGCTTCCACAGGGRTGGAAgGGATCACCAGCTATATTCCAA
TGTAGCATGACAAAAATCTTAGAGCCTTTTAGAAAACAAAATCCARACATAGTCATCTAT
CArTACGTGGATGATTTRTATGTAGGaTCTGACTTAGAAATAGGGCAGCATAGGGCAAAA
ATAGATGAACTGAGACAATATCTGTGGAAGTGGGGATTCTACACACCAGAAGAAAAACAT
CAGAAAGASCCTCCRTTCCATTGGATGGGRTATGAACTCCATCCTGATAAATGGACAGTA
CAGCCTATAGTGTTGcCA
>69_MI
ACTCTTTGGCAACGACCCATCGTCACAGTAAAAATAGGGGGGCAAGTAacAGAAGCCCTA
TTAGATACGGGAGCAGATGATACAGTATTAGAGGAAATGAATTTGCCAGGAAGATGGAAA
CCAAAATTAATAGGGGGAATTGGAGGTTTTATCAAAGTAAGAGARTATGATCAGGTACCC
ATAGAAATCTGTGGACATAAAGCTGTAACTACAGTRTTAATAGGACCTACCCCTGTCAAC
ATAATTGGAAGAAATGTGATGACTCAGATTGGGTGCACTTTAAATTTTTGTACAGAATTG
GAAAWGGAMGGGAAAATTACAAAAATTGGGCCTGAAAATCCATATaATACTCCAGTATTT
GCCATAAAGAAAAAAGATAGTACTAAATGGAGAAAAWTAGTAGATTTCAGAGAACTTAAC
AAGAGAACTCAAGACTTCTGGGAAATCCAATTAGGAATACCACATCCCGCAGGGTTAGAA
AAGAAAAAATCAATAACAGTACTGGATGTGGGTGATGCATATTTTTCAGTTCCCTTAGAT
GAAGACTTCAGGrAATATACTGCATTTACCATACCcaGYATAAAYAaTGAGACACCAGGG
ATYAGATATCAGTACAATGTGCTTCcacaGgGATGGAAAGGATCACCAGCAATATTCCAr
TAYAGCATGACAAGGATCTTAGAGCCTTTTAGAAAACARAATCCAAACATAAKTATCTGT
CAATAYGTGGATgATTTGTATGTAGCATCTgACTTAGAAATAGGGCAGCATAGGGCAAAA
ATAGAGGAACTGAGACAACATCTGTGGAGGTGGGgaTTTTACACACCAGACAAAAAACAT
CAGAARGAaCCCCCATTCCTTTGGATGGGTTATGAACTCCATCCTGAYAAATGGACAGTA
CAGCCTATAGTGCTGcCA
>70_MI
ACTCTTTGGCAACGACCCtTCGTCACARTAAAGATAGGRGGGCAGCTAAGGGAAGCYTTA
TTAGATACAGGAGCAGATGATACAGTATTTGAAGATATAGATTTGCCAGGAAAWTGGAMA
CCAAAAATAATAGGAGGACTTGGAGGTTTTATCAAAGTAAGACAGTATGATCAGGTAGmC
ATAGAAATCTGTGGACAYAAARYTATARGTACAGTGTTAGTAGGACCTACACCTGCCAAC

ATAATTGGAAGAAACCTGWTGACTCAGATTGGCTGCACTTTAAATTTTTGTACAGAAATG
GAAAAGGAaGGAAAAATTTCAAgAaTTGGGcCTGAAAATCCATACAATACTCCAGTATTT
GCYATAAAGAAAAAAGaCAGTACTAAATGGAGAAAATTAGTAGATTTCAGAGAACTTAAT
AAGAGAACTCAAGACTTCTGGGAAGTTCAATTAGGAATaCCACATCCAGCAGGGTTAAAA
AAGAAAAAATCAGTgACAGTACTGGATGTGGGTRATGCATATtTCTCAGTTCCCTtAGAT
GAAGACTTCAGGaAGTATACTGCATTcACCATACCTrGTATAAACAATGAGACACCAGGG
ATTAGATaTcAGTACAATGTGCTcCCACAGGGATGGAAAGGATCACCAGCAATATtCCAA
AGTAGCATGACAAAARTTTTAGAGCCTTTTAGAAAACAAAATCcAGACATAATTATCATT
CAATACGTGGATGATTTGTACGTRGGATCTGATYTAGAAATAGGGCAACATAGAGCAAAA
ATAGAGGAACTGAGACAACATCTGTTGAGGTGGGGATTWTTTACACCAGACAAAAAACAT
CAGAAAGAACCTCCATTCAATTGGATGGGTTATGAACTyCATCCTGATAAATGGACAGTA
CAGCCTATAATGCTGCCA
>71_MI
ACTCTTTGGCAACGrcCCTTCGTYgCAGTAAAGrTAGGGGGGCAACTAAAGGAAGCTCTA
YTAGATACAGGRGCAGATGATACARTATTCGAAGAAATGAATTTACCAGGAAGATGGAAA
CCAAAAATRATAGGGGGAATTGGAGGTTTTATCAAAGTAAGASAGTATRAYCAGATACYC
RTAGAAATCTGTGGACATAAAGYTRTAGGTACAGTATTAATAGGACCTACACCTGYCAAy
RTAATTGGAAGAAATCTGTTRACTCAGATTGGCTGCACTTTAAATTTTTGTGCAGAATTG
GAAAAGGAAGGAAAAATTTCAAAAATTGGGCCTGAAAATCCATATaATACCCCAGTATTT
GCCATAAAGAAAAAAGACAGTACTAAATGGAGAAAATTAGTAGATTTTAGAGAACTTAAT
AAGAGAACTCAAGACTTCTGGGAAGTTCAATTAGGAATACCACATCCAGCAGGGTTACCR
AAGAACAAATCrGTAACAGTACTGGATGTGGGGGATGCATATTTTTCAGTTCCACTAGAT
GAAGACTTCAGGAAGTATACTGCATTtACCATACCTAGTACAAACAATGAAACGCCAGGG
ATTAGGTATCAGTACAATGTGCTTCCACAGGGATGGAAAGGATCACCAGCAATATTCCAA
AGTAGCATGACAAAAATYTTAGATCCTTTTAGAAAACAAAATCCAGACATARTTATCTAT
CAATACGTGGATGATTTGTATGTAGGATCTGATTTAGAAATAGGGCAGCATAGARCAAAA
ATAGAGGAACTGAGACAACATCTGTKGAGGTGGGGGTTTTACACACCAGACAAAAAACAT
CAGAAAGAACCACCATTCCTTTGGATGGGTTATGAACTCCATCCTGATAAATGGACAGTA
CAGCCTATAGTGCTGCCA
>72_MI
ACTCTTTGGCAACGACCCTTCGTCACAGTAAAGATAGGGGGGCAGCTAAGGGAAGCTCTA
TTAGATACAGGAGCAGATAATACAGTATTAGAAGACATAAATTTGCCAGGGAGATGGAAA
CCAARAATGATAGGGGGAATtGGAGGKTTTATCAAAGTAAAACAGTATGATGATATACTT

GTAGAYATTTGTGGACACAAGGCTACAGGTACAGTGTTGGTAGGACCTACRCCTGTCAAC
ATAATTGGAAGAGATCTGTTGACTCAGATTGGCTGCACTTTAAATTTTTGTAcAGAAATG
GAAAAAGAaRGgAAAATCTCAAArATAGGACCTGAAAATCCATACAATACTCCAATATTT
GCCATAAAGAAAAAGGACAGTAATAGATGGAGGAAATTAGTAGATTTCAGAGAACTTAAT
AAAAGRACTCAAGATTTTTGGGAGATTCAATTAGGAATACCGCATCCTGCTGGGATAAAA
AAGAACAAGTCAGTAACAGTGTTGGATGTGGGGGATGCATATTTTTCAGTTCCCTTRGAT
AARGACTTTAGGAAGTATACTGCATTCACTATACCTAGTACCAACAATGAGACACCAGGA
ATTAGGTATCAGTACAATGTGCTTCCACAAggaTGGAAaGGGTCACCAGCAATATTCCAA
TATAGTATGACAAAAATCTTAGAKCCCTTTAGAACAAAGAATCCAGGAATARTTATCTAT
CAATACGTGGATGATTTGTATGTAGGATCTGATTTAGAAATAGAGCAGCATAGAGCAAAA
ATAGAGGAGCTGAGACAACATCTGTTRAGGTGGGGGTTAACCACACCAGACCAAAAACAT
CAGAAAGAACCACCATTCCGTTGGATGGGTTATGAACTCCATCCTGATAAATGGACAGTA
CAGCCTATAGTGCTGCCA
>73_MI
ACTCTTTGGcAACgACCCGTCGTCACAGTAARGGTAGGAGGGCAACTAARGGAAGCTCTA
TTAGATACAGGAGCAGATAATACAATATTYGAAGATCTGAGTTTACCAGGAAAATGGAAA
CCAAGAYTGGTAGGGGGACTTGGAGGTTTTATCAACGTAAGACAGTATGATCAGATACCC
ATAGAAATCTGTGGACATAAAwCTATAGGTACAGTATTAGTAGGACCTACACCTGTCAAC
ATAATTGGAAGAGATCTGTTGACTSAGATTGGCTGCACTTTAAATTTTtgtACAGAAATG
GAAAAGGAAGGAAAAATTACAAAAATtGGGCCCGAAAATCCATACAATACTCCAGTATTT
GTCATAAAGAGAAAAGAcRGTAYTAAATGGAGAAAATTAATAGATCTCAGAGAACTTAAT
AAGAGAACTCAAGACTTCTGGGAAGTCCAATTAGGAATACCACATCCCGGAGGGTTACAG
AAGAAAAAATCAGTAACAATACTGGATGTGGGTGATGCATATTATTCAGTTCCCTTAGAT
GAAGAYTTCAGGAAGTATACTGCATTTACMATACCtAGTCTAAACAATGAGACGcCAGGA
ATTAGATATCAGTACAATGTGCTTCCAATGGGATGGAAAGGATCGCCAGCAATATTCCAA
ASTAGCATGACAAAAATCCTAGAACCTTTTAGAAAGAAAAATCCAAACATAGTTATCTGT
CAGTACGTGGATGATTTGTATGTAGCATCTGACTTAGAAATAGGGCAGCATAGAACAAAA
ATAGAGGAACTGAGACAACATCTGTTGAGGTGGGGGCTTACCACACCAGACGAAAAACAT
CAGAAAGAACCACCATTCCTTTGGATGGGTTATGAACTCCATCCTGATAAATGGACAGTA
CAGCCTATAGTGCTGCCA
>74_MI
ACTCTTTGGCAGCGACCCTTCGTCCCAGTAAAGATAGGGGGGCAACAAACGGAAGCTCTA
ATAGATACAGGAGCAGATGATACAGTATTAGAAGACATGAATTTGCCAGGAAGATGGACA

CCAAAAATAATAGGGGGAATTGGAGGTTTTGTCAAAGTAAGGCAGTATGATCAGATACCC
GTAGAAATCTGTGGGCATAAGACTATAGGTACAGTATTAGTAGGACCTACACCTGCCAAC
ATAATTGGAAGAAATCTGTTGTCCCAGATTGGCTGTACTTTAAATTTTTGTACAGAAATG
GaAAAGGAAGGaAAAATTTCAAAAATTGGGCCTGAAAATCCATACAATACTCCAGTATTT
GCCATAAAGAAGAAAAACAGTACTAGATGGAGAAAATTAGTAGATTTCAGAGAACTTAAT
AAGAGAACTCAAGACTTCTGGGAAGTCCAATTAGGAATACCACATCCCGCAGGATTAAAA
AAGAAAAAATCAGTAACAGTACTGGATGTGGGTGATGCATATTTTTCAGTTCCTTTAGAT
GAAAACTTTAGGAAGTATACTGCATTTACCATACCTAGTACAAACAATGAGACACCAGGG
GTTAGATATCAATACAATGTGCtTCCACAAGGATGGAAAGGATCACCAGCAATATTCCAA
AGTAGCATGACAAAAATTTTAGAGCCTTTTAGAAAACAAAATCCAGACATAGTTATTTAT
CAATACGTGGATGATTTATATGTAGGATCTGACTTAGAAATAGGGCAACATAGAACAAAA
GTARAGGARCTGAGACAGCATCTRTTGAAGTGGGGACTTTTCACACCAGACCAAAAACAT
CAAAAAGAACCTCCATTCCTTTGGATGGGTTATGaACTCCATCCTGATAAATGGACAGTA
CAGCCyATAGTGCTGCCA
>75_MI
ACTCTTTGGcAACGACCCGTCGTCACAGTAAAGATAGGGGGGCAACTAAAGGAAGCTCTA
TTAGATACAGGAGCAGATGATACAATATTTAGAGAAATGCATTTGCCAGGAAATTGGAAA
CCAAAAATAGTAGGGGGAATTGGAGGtTTTATGAGAGTaAGAGAATATGAGCAGGTACCT
GTAGAAATTTGYGGACATAAAACTRTAGGTACAGTATTAATAGGACCTACACCTGCCAAC
ATAATTGGAAGGAATCTRATGACTCARCTTGGCTGCACTTTAAATTTTtGTACAGAACTG
GAAAAGGAAGGAAAAATTTCAAGAGTTGGGCCTGAAAATCCATACAATACTCCAGTATTT
GCCATATACAAAAAGAACAGTACTAGATGGAGAAAGATAACAGATTTCAGAGAACTTAAT
AAGAGAACTCAAGACTTCTGGGAAGTTCAATTAGGAATACCACATCCCGCAGGGTTACAA
AAGAGAAAATCAGTAACARTACTGGATGTGGGTGATGCTTACTTTTCAATTCCCTTAGAT
CCAGAATTCAGAAAGTATACAGCGTTTACAATACCTAGTAAAAACAATGAGACACCAGGG
ATTAGATATCAGTACAATGTGCTTCCACAGGGATGGAAAGGATCACCAGCAATATTCCAA
GACAGCATGACAAAAATCTTAGATCCTTTTAGAAAACAAAATCCAGACATAGAAATCTGT
CAATACGTGGATGATCTATATGTAGCATCTGACTTAGAAATAGGGCAACATAGAGCAAAA
ATAGAGGAACTGAGACAACATTTATTGAAGTGGGGACTTTTCACACCAGAACAAAAACAT
CAGAAAGAACCRCCATTCCATTGGATGGGTTATGAACTCCATCCTGATAAATGGACAGTA
CAGCCTATAAAGCTGCCA
>76_MI
ACTCTTTGGCAACGACCACTAGTCACAATAAAGGTAgGGGGGcaACTAAAGGAAGCTCTA

YTAGATACAGGAGCAGATGATACAGTATTAGAAGAAATAGAGTTGCCAGGAAGATGGAAA
CCAAAAATGATAGGGGGAATTGGAGGTTTTATCAAAGTAAGACAGTATGATCAGATACYC
ATAGAAATTTGTGGACATAAAGCTATAGGTACAGTATTAGTAGGACCTACACCTGTTAAC
ATAATTGGAAGAAATCTGWTGACTCAgATTGGATGCACTTTAAATTTTTGtacAgAAATG
GAAAAGGAAGGAAAAATTTCAAAAATTGGGCCTGAAAATCCATACAATACTCCARTATTT
GTCATAaAGAAAAAAGATASTACTAAATGGAGAAAATTAATAGATTTCAGAGAACTTAAT
AAGAGaACTCAAGACTTCTGGGAAGTTCAATTrGGAATACCACATCCCKCAGGGTTAAAG
AAGAATAAATCAGTGACAATACTAgATATaGgTGATGCATATTWTTCAGTTCCCTTAGAT
AAAGAATTCAGGAAGTATACTGCATTCACCATACCTAGTATAAACAATGAGACACCAGGG
ATtAGATATCARTACAATGTGCTGCCCATGGGATGGaAAgGATCACCAGCAATATtCCAG
AGTAGCATGACAAAAATCCTAGAGCCTTTTAGAAAACAAAATCCAGACCTARTYATCTAT
CAATACATGGATGATTTATATGTAGGaTCTGACTTAGAGATAGGGCAGCATAGAACAAAA
ATAGAAGAACTGAGACAATATCTGTTGARGTGGGGRTTTACCACACCAGACAAAAAACAT
CAGAAAGAACCTCCRTtCCTTTGGATGGGTTATGAGCTCCATCCTGAYACATGGACAGTA
CAGCCTGTAAAGTTGCCA
>77_CO
ACTCTTTGGCAACGGCCCrTCGTCACAGTAAAGATAGAGGGGCAACTaACGGAAGCTCTA
ATAGATACAGGAGCAGATGATACAGTrTTCGAAGAAATAAATTTGCCAGGAARATGGAAA
CCAAAATTrATAGGAGGAATTGGAGGTTTTCTcAAAGTAAGACAGTATGATCAAGTAACC
CTAGAAATCTGTGGAAAGAAAGCTGTAGGTTCAGTATTAGTAGGACCTACACCTGCCAAC
GTAATTGGAAGAAATATGTTGACTCAGATTGGTTGCACTTTAAATTTTTGTACAGAAATG
GAAAAGGAAGGAAAAATTTCAAAAATTGGGCCTGAAAATCCATATAATACTCCAGTATTT
GCYATAArGAAAAAAGACAGTACWAGATGGAGAAAATTAGTAGATTTYAGAGAACTTAAT
AARAGAACTCARGACTTYTGGGAAGTTCAATTAGGAATACCACATCCTGCAGGGTTAAAA
AAGAACAAATCAGTAACAGTACTGGATGTGGGTGATGCATATTTTTCAGTKCCCTTGGAT
GAAAACTTCAGGAAGTATACTGCATTtACYATACCTAGTATAAACAATGAGACACCAGGG
ATTAGATATCAGTACaATGTGCTTCCACAGGGATGGAAAGGATCACCAGCAATATTTCAA
AGTAGCATGACAAAAATCTTAGAGCCTTTCAGAAAACAAAATCCArACATAATTATCTAT
CAATATGTGGATGAyTTGTATGTAGGATCTGACTTAGAAATAGGGCAGCATAGAACAAAA
ATAGArGAACTGAGACAGCATCTGTTGAAGTGGGGACTTACCACACCAGACCAGAAACAy
CAGAAAGAACACCCATTCCATTGGATGGGGTATGAACTCCATCCTGATAAATGGACAGTA
CAGACTATAAAGCTGCCA
>78_CO

ACTCTTTGGCAACGACCCCTAGTTACAATAAGAgTAGGGGGACAGCTAAAAGAAGCTTTA
TTAGATACAGGAGCAGATGATACAgTATTAGAAGACATAAATTTGCAAGGGAAATGGAAA
CCAAAAATGATAGGGGGAATYGGAGGTTTTATCAAAGTaAaACAGTaTgAtAATATACTC
AtaGaaATTtGTGGACACAAAGCTATAGGTACAGTATTGGTAGGACCTACACCTGTCAAC
ATAATTGGAAGAAATATGTTGACTCAGATTGGTTGTACTTTAAATTTTtGTCTAGaAATG
GaAAAGGAAGGAAAAATTTCAAAAATTGGGCCTGAAAATCCATACAATACTCCAGTATTT
GCCATAAAGAAAAAAGACAGTACTAAATGGAGAAAATTAGTAGATTTCAGAGAACTTAAT
AAAAGAACTCAAGATTTTTGGGAGGTTCAATTAGGAATCCCGCACCCTGCAGGGCTAAAA
AAGAAAAAGTCAGTAACAGTACTGGATGTGGGGGATGCATATTTTTCAGTTCCCTTAGAT
GAGGATTTCAGGAAGTACACTGCATTCACcATACCTAGTACCAACAATGAGACACCAGGA
ATTAGGTATCAGTACAaTGTGCTTCCACAAGGATGGAAgGGATCACCAGCAATATTCCAA
TGTAGCATGACAAAAATCTTAGATCCCTTTAGAGCAAAAAATCCAGACATAGTtATTTAC
CAATACATGGATGATTTGTATGTAGGATCTGACTtAGAAATAGGACAGCATAGAGCAAAA
ATAGAGGAGTTAAGaGCACATCTGCTGAAAYGGGGATTTACTACGCCAGACAAAAAACAT
CAAAAAGAACCCCCATTCCTTTGGATGGGGTATGAACTCCATCCTGATAAATGGACAGTG
CAGCCTATACAATTGCCA

>79_CO

ACtCTtTGGCAACGACCCCtCGTCCCAATaAGGATAGGRGGGCAACTAAAGgAAGCTCTA
TtAgATACAGGAGCAGATGATACAGTwTTAGAAGAAATGAGTTTGCCAGGAAGATGGAAA
CCAAAAATkATAGGGGGAATTGGAGGtTTTATCAAAGTAAGRCAGTATGAYCAGATACCK
GTAGAAATYTATGGACATAAGGCTATAGGTACAGTATTAGTAGGACCYACACCTGTCAAC
ATAATTGGAAGAAATCTGTTGACACARATTGGTTGCACTTTAAaTTTTTGTACAGAAATG
GAAAAAGAAGGAAAAATCTCRAAAATTGGGCCTGAAAATCCATACAATACTCCAGTATTT
GCCATAAAGAAAAARGACAGTACTAAATGGAGAAAATTAGTAgATTTCAGAGAACTTAAT
AAGAGAACTcAAgATTTTTGGGAAGTTCAATTAGGAATACCACATCCCGCAGGGTTRAAA
AAGAAAAAATCAGTaacAgTACTAGATGTGGGTGATGCATATTTTTCAGTTCCCTTGGAT
AAAGAATTCAGGAAGTaCACTGCATTTaCCAtACCTaGTACAAACAATgAAACACCAGGG
RtTAGATatcAGTaTDATGTGCTTccACAGGGGTGGAAAGGATCACCAGCAATATTCCAA
tgTAGCATGACAAAGATTTTAGARCCTTTTAGAAAACAAAAYCCAGAGATAGTTATCTAT
CAATACATGGAYgacTTGTATGTAGGATCTGACTTAgAAATAGGGCAGCATAGAATaAAA
ATAGAGGAGCTGAGACAACATCTGTTGAGGTGGGGATTTACCACACCAGACAAAAAACAY
CAGAAAGAACCTCCATTyCTTTGGATGGGTTATGAACTCCATCCTGATAAATGGACAGTA
CAGCCTATAACRCTGCCA

>80_CO

ACTCTTTGGCAACGACCCCTCGTCACAATAAGAATAGGGGGGCAACTAAAGGAAGCyCTA

TTAGATACAGGAGCAGATGATACAGTATTAGAAGAAATGGAGTTGCCAGGAAGATGGAAA

CCAAAAATGATAGGGGGAATTGGAGGTTTTATCAAAGTAAGACAGTATGATCAGGTACAC

TTAGAAATCTGYGGACAYAAAGCTATAGGTACAGTATTAATAGGGCCTACACCKGTCAAC

ATAATTGGAAGAAACCTGTTGACTCAACTTGGCTGCACTTTAAATTTTTGTACAGAAATG

GAGAAGGAAGGAAAAATTTCAAAAATTGGGCCTGAAAATCCATACAATACTCCARTATTT

GCCATAAAGAAAAAGGACAGyAMTARATGGAGAAAACTAGTGGATTTCAGAGAACTTAAT

AAAAGAACTCAAGACTTCTGGGAAGTkCAATTAGGAATACCACATCCMGCAGGrtTAAAA

AaGAAMAAATCAGTGACAGTACTGGATGTGGGAGACGCATATTTTTCAGTTCCCTTACAT

GAAGACTTTAGGAAGTATACTGCATTTACCATACCTAGTACAAACAATGAGACACCAGGG

RTGAGGTATCAATACAATGTGCTTCCACAGGGATGGAAAGGATCACCAGCAATATTCCAA

AGTAGCATGACAAAAATCTTAGAGCCTTTTAGAAAACAAAATCCAGAAATAgTTATCTAT

CAATACRTGGATGATTTGTATGTAGGATCTGACTTGGAAATAGGGCAGCATAGAACAAAA

GTAGAGGAGCTAAGACAACATCTGTTGARGTGGGGGTTkACYACACCAGACMAAAArYAT

CAGAAAGAACCyCCATTCCKTTGGATGGGTTATGAACTCCATCCTGATAAATGGACAGTA

CAGCCTATAAYGCTGCCA

>81_CO

ACTCTTTGGCAACGACCCCTCGTCGTCATAAAGGTAGGGGGGCAACTAAAAgAAGCTCTA

TTAGATACAGGAGCAGATGATACAGTATTAGAAGAAATGGAKTTGCCAGGAAGaTGGAAA

CCAAAAATgATAGGGGGAATTGGAGGTTTTATCAAAGTAAAACAGTATGATCAAATACCC

ATAGAAATCTGTGGACATAAAGCTrTAGGTACAGTATTAGTAGGAcCKACACCKGTCAAC

ATAATTGGAAGAAATCTGTTGACTCAGATTGGCTGCACTTTAAATTTTTGTACAGAAATG

GAAAAGGAAGGAAAAATATCAAAAATTGGGCCTGAAAATCCATACAATACTCCAGTATTT

GCMATAAAGAAAAARGATAGTACTAAATGGAGAAAATTAGTAGATTTcAGAGAACTTAAT

AAGAGAACTCAAGACTTCTGGGAAgTYCAATTAGGAATACCACATCCbGCRGGGTTARAG

AAGAAAAARTCArTAACAGTACTGGATGTGGGTGATGCATATTTYTCAGTTCCMTTAGAT

GAAGACTTCAGGAAGTAYaCTGCATTYACcATACCTAGTAYAAACAATGAAAGACCAGGG

ATTAGATATCAGTACaATGTGCTTCCACAGGGATGGAAgGGATCACCAGCARTATTCCAA

aGTAGCATGAYAARrATYTTAGARCCTTTTAGAAAACAAAATCCAGACATAGTTATCTGT

CAATACGTGGATGACTTGTATGTAGSATCTGACTTAGAAATAGGGCAGCATAGAGCAAAA

ATAGAGGAATTGAGACAGCATTTGTTAAGGTGGGGAYTTACCACACCAGATAAAAAACAC

CAGAAAGAACCTCCATTCCTTTGGATGGGTTATGAACTCCATCCTGATAAATGGACAGTA

CAGCCTATAGTGCTGCCA

>82_CO

ACTCTTTGGCAACGaCCCCTCGTcRCAGTAaRAATAGGGGGACAGCTAAAgGAAGCTCTA

TtAgATACAGGAGCAGATGATACAGTATTAGaAGAAATGAGTTTGCCAGGAAAATGGAAA

CCAAAAATGATAGGGGGAATTGGAGGCTTTATCAAAGTAAGACARTATGATCAGATACTT

GTAGAAATTTGTGGGCATAAAGCTATAGGTACAGTGTTAATAGGACCTACACCTGTCAAC

ATAATTGGAAGAAATCTGTTGACTCAGATTGGCTGyACTTTAAaTTTTTGtACAGAAATG

GAAAAGGAAGGAAAAATTTCAAAAATTGGGCCCGAAAATCCATACAATACTCCaGTATTT

GCTATAAAGAAAAAAGAYAGTACTAAATGGAGAAAATTAGTAGATTTCAGAGAACTYAAT

AAGAGAACTCAAGACTTTTGGGAAGTTCAATTAGGAATACCACATCCCTcAGGGTTAAAG

AAGAAAAAATCAGTAACAGTACTGGATGTRGGTgATGCATATTTTTCAGTTCCCYTAGAT

RAAGACTTCAGGAAGTATACTGCATTYACcATACCTaGtACAAACAATGAAACRCCAGgA

ATTAGATATCAGTAYaATGTGCTTCCACAgGGATGGAAAGGATCACCAGCAATATTCCAA

AGTAGCATGACAAAAATCTTAGAGCCTTTTAGAAAACAAAATCCAGACATAGTTATCTAT

CAATAYATGGATGATTTRTATGTAGGATCTgACTTAGAAATAGGGCTGCATAGAACAAAA

ATAGAGGAACTGAGACAACATCTGyTGAGGTGGGGGTTTACCACACCAGACAAGAAACAT

CAGAAAGAACCACCATTYCTTTGGATGGGGTATGAACTCCATCCTGATAAATGGACAGTR

CAGCCTATAGTGTTGCCA

>83_CO

ACTCTTTgGCAACGACCCCTCGTcaCCGtAAAGGTAGGGGGGCAACTaMTAGAAGCTtTA

TTAgATACAGGAGCAGATGATACAGTaTtAGAAGAAATAAATTTGCCAGGAAGATGGAAA

CCAAAAATAATAGGAGGAATTGGAGGTTTTGTCAAAGTAAGACARTATGATCAAATAcCT

ATAGAAATATGTGGAAAGAAAGCTCTAGGTACAGTATTAGTAGGACCTACACCTGTAAAC

ATAATTGGAAGAAATCTGATGACTcAAATtGGYTGCACTTTAAaTTTTTGTACASAAATG

GAAAAGGAAGGAAAAATTTCAAAAATTGGACCTGAAAATCCATATWATACTCCAGTATTT

GTCATAAAGAAAAARGACGGTAAAACATGGAGAAAATTAATAGATTTCAGAGAACTTAAT

AAGAGAACTCAAGACTTCTGGGAAGTTCAATTGGGAATACCCCATCCCGCAGGA-TAAAR

AAGAACAAATCAGTAACAATACTAGAtgTGGGTGATGCATATTATTCArTTCCCTTAGAT

GAAGAATTCAGGAAGTaTACTGCATTTaCCATaCCTgGTACAAACAATGAGACACCAGGG

ATTAGATATCAGTACMaTGtGCTTccaAtgGGATGGaAAGGATcACCAGCAATATTCCAA

AGCAGCATGACAAAAATCTTAGAGCCTTTTAGAAAACAAAACCCAGACATAGTTATcTAT

CAATACATGGATGATTTGTATGTAGGCTCTgATTTAGAAATAGGRCAGCATAGARCAAAA

ATAGAGGAACTRAGACAACATCTGTTAARGTGGGGACTGACCACACCAGACAAAAAACAC

CAGAAAGAACCTCCCTTCCATTGGATGGGTTATGAACTCCACCCGGATAAATGGACAGTA
CAGCCTATAGAGCTGCCA
>84_CO
ACTCTTTGGCAACGACCCCTCGTCACAATAAAgATAGGGGGGCAACTAAAGgAAGCTCTa
TtAgATACGGGAGCAGATGATACAGTaTTAGAAGAAATGAATTTGCCAGGAAGATGGAAA
CCAAAAATgATAGGGGGAATTGGAGGTTTTATCAAAGTaAGACAGTATGATCAAATACTA
GTAGAAATTTGTGGRCATAAAGCTATCGGTACAGTATTAGTAGGACCTACACCTGTCAAC
ATAATTGGAAGAAATCTGTTGACTcAGATTGGTTGCACTTTAAATTTTTGTACAGAAATG
GAAAAGGAAGGAAAAATTTCAAGAATTGGGCCTGAAAATCCATACAATACTCCAGTATTT
GCCATAAAGAAAAAAGACAGTACTAAATGGAGAAAATTAGTAGATTTCAGAGAACTTAAC
AAGAGAACTCAAGACTTCTGGGAAGTTCAATTAGGRATACCACATCCCTcAGGRTTAAAA
AAGAAAAAATCAGTAACAGTACTGGATGTGGGSGATGCATATTTTTCAGTTCCCTTAGAT
AAAGACTTCAGGAAGTaTACTGCATTTACCATACCTAGTACAAACAATGAaACACCAGGG
ATTAGATATCAGTACAATGTGCTtCcACAGGGATGGAAaGGATCACCAGCAATATtCCAG
AGTAGCATGATAAAAATCTTAGAGCCTTTTAGAAAACAAAACCCAGATATAGTAATCTAT
CAATACATGGATGATTTGTACGTAGGGTCTGACTTAGAAATAGGACAGCATAGAGCAAAA
ATAGAGGAGCTAAGACAACATCTGTTGAGGTGGGGATTTACCACACCAGACAAAAAACAT
CAGAAAGAACCTCCATTCCTTTGGATGGGTTATGAACTCCATCCTGATAAATGGACAGTA
CAGCCYATAATGCTgCYA
>85_CO
ACTCTTTGGcAACGACCCATCGTCACAgTAAaAATAGGGGGGcaAcmAAGGGAAGCTCTA
YtAGATACAGGAGCAGATGATACAATAATAGAAGAAATAAATTTACCAGGAAAATGGAGA
CCAAAAATAATAGGGGGAATTGGAGGTTTTGTCAAAGTAAGACArTATGATCAAGTACAG
ATAGAAATCTGCGGACATAAAGTTGTAGGTACAGTATTGGTAGGACCTACACCTGCCAAC
ATAATTGGAAGAAATGTATTAACTCGGATTGGCTGTACTTTAAATTTTTGTGCAGAATTA
GAAAAGGAAGGAAAAATTTCAAAAATTGGGCCTGAAAATCCATACAATACTCCAGTATTT
GCTATAAAGAAAAAGAAyGGTACYAAATGGAGAAAATTAGTGGATTTCAGAGAACTTAAT
AAGAGAACTCAAGATTTCTGGGAAGTTCAATTAGGAATACCACATCCTGCAGGGTTAAAA
AAGAAAAAATCAGTAACAGTACTGGATGTGGGTGATGCATATTTTTCAGTTCCCTTAGAT
AAARAMTTCAGGAAGTACACTGCMTTtACcATACCTaGTATAAACAATGAGACACCAGGG
ATtAGATAtCAGTACAATGTGCTTCCACAGGGATGGAAAGGgTCACCAGCAAtAtTCCaA
AGTAGCATGACAAAAATCTTAGAGCCTTTTAGAAAACAAAATCCAGAGATARTTATCTAT
CAATATGTGGATGATTTGTATGTAGGATCTGAYTTAGAAATAGGGCAGCATAGAGCAAAA

ATAGAGGAACTGAGACAACATCTATGGAArTGGGGGTTTTACACACCAGACGAAAAACAT
CAGAAAGAACATCCATTCCTTTGGATGGGTTATGAACTCCATCCTGATAAATGGACAGTA
CAGCCTATAGTGCTGCCA
>86_CO
ACTCTTTgGCAACGACCACTAGTCAATATAAAGGTAGGGGGGCAACTAAAGGAAGCTCTA
TTAgATACAGGAGCAGATGATAcAGTattAGAAGAAATAGAGTTGCCAGGAAGATGGAAA
CCAAAAATgATAGGGGGAATTGGAGGTTTTATCAAAGTAAGACAGTATGATCAGATAcTC
ATAGAAATTTGTGGACATAAAGCTATAGGTACAGTATTAGTAGGACCTACACCTGTCAAC
ATAATTGGAAGAaATTTGTTGACTCAGATTGGATGCACTTTAAATTTTWGTACAGAAATG
GAAAAGGAAGGAAAAATTTCAAAAATTGGGCCTGAAAATCCATACAATACTCCAGTATTT
GCCATAAAGAAAAARGACAGTACTAAATGGAGAAAATTAGTAGACTTCAGAGAACTTAAT
AAGAGAACTCAAGAcTTCTgGGAAGTTCAATTAGGAATACcACATCCCGCAGGGTTAAAA
AAGAAGAAATCAGTGACAGTACTAGATGTGGGTGATGcATATTTTTCAGTTCCCTTAGAT
AAAGACTTCAGGAAGTATACTGCATTCACYATACCTaGTACAAACAATgAGACACCAGGG
ATTAGATATCAGTACAATGTACTTCCACAGGGATGGAAAGGATCACCAGCAATATTCCAG
AGTAGCATGACAAAAATCCTAGAGCCTTTTAGAAAACAAAATCCAGACATAGTTATCTAT
CAATACATGGATGATTTATATGTAGGATCTGACTTAGAAATAGGGCAGCATAGAACAAAA
ATAGAAGAACTGAGACAACATCTGTTGAGGTGGGGATTTACCACACCAGACAAAAAACAT
CAGAaAGAACCTCCATTCCTTTGGATGGGTTATGAGCTCCATCCTGATAAATGGACAGTA
CAGCCTGTAATGTTGCCA
>87_CO
ACTCTtTGGCAACGACCCgtCGTCACAGTAAAGGTAGAGGGGCAACTAAAGGAAGCTCTA
TTAgATACAGGAGCAGATGATACAGTAttAGAAGAAATGGAGTTACCWGGAAGATGGAAA
CCMAAAAtgATAGGGGGAATTGGAGGATTTATTAAAGTAAGACAGTATGATCARRTAAcC
ATAGaTATCTGTGGACATAAAGCTACAGGTACAGTATTAGTAGGACCTACACCTGTCAac
aTaATYGGAAGAAATCTGTTGACTCAGATTGGCTGCACTTTAAATTTTTGTACAGAAATG
GAAAAGGAAGGAAAAATTTCAAAAATTGGGCCTGAAAATCCATATAATACTCCAGTATTT
GCAATAAAGAAAAARGACAGTACTAAATGGAGRaAATTAGTAGATTTcAGAGAACTTAAT
AAAAgAaCTCAAGACTTCTGGGAArTCCAATTAGGCATACCACATCCTGCAGGGATAAAA
AAGAATAAATCAGTAACAGTACTAGATGTRGGTGATGCATATTTTTCAGTTCCCTTAGAT
AAAGAATTCAGGAAGTATACTGCATTTACCATACCTAGTACAAACAATGAgACACcaGGG
AtTAGATATCAGTACAATGTGCTTCCACAGGGATGGAArGGATCACCAGCAATATTCCAA
AGTAGCATGACAAGAATCTTAGAGCCTTTTAGAAAACAAAATCCAGACCTAGTTATCTAT

CAATACGTGGATGACCTGTACGTAGGATCTGACTTAGAAATAGGACAGCATAGAGCAAAA
ATAGAGGAACTGAGACAACATCTGyTGAGGTGGGGACTTACCACACCAGACAAAAAATAT
CAGAAAGAACCTCCATTCCTTTGGATGGGTTATGARCTCCATCCTGATAAATGGACAGTA
CAGCCTATAGTGCTGCCA
>88_CO
ACTCTtTGGCAACGACCCCTCGTCGTCATAAAGGTAGGRGGGCAACTAAAArAAGCTCTA
TWAgATACAGGAGCAGATGATACAGTaTTAGAAGAAATGGATTTGCCAGGAAgATGGAAA
CCAAAAATGATAGGGGGAATTGGAGGTTTTATCAAAGTRAAACAGTATGATCAAATACCC
ATAGAAATCTGTGgRCATAAAGCTATAGGTACAGTATTAGTAGGACCKACACCTGTCAAC
ATaATTGGAAGAAATCTGTTGACTCAGATTGGCTGCACTTtAAaTTTTTGTACAGAAATG
GAAAAGGAAGGAAAAATATCAAAAATTGGGCCTGAAAATCCATACAATACTCCARTATTT
GCCATAAAGAAAAAGGATAGTACTAAATGGAGAAAATTAGTAGATTTCAGAGAACTTAAT
AAGAGAACTCAAGACTTCTGGGAAGTTCAATTAGGAATACCACATCCGGCGGGGtTAGAG
AAGAAAAAATCAGTAACAGTACTGGATGTGGGTGATGCATATTTCTCAGTTCCCTTAGAT
GAAGACTTCAGGAAGTATACTGCATTtACcATACCTAGTACAAACAATGAAAGACCAGGG
ATTaGATATcAGTACAATGTGCTTCCaCAGGGATGGAAgGGATCACCAGCAATATTCCAA
AGTAGCATGACAAGAATTTTAGAGCCTTTTAGAAAACAAAATCCAGACATAGTTATCTGT
CAATACGTGGATGACTTGTATRTAGCATCTGACTTAGAAATAGGGCAGCATAGAGCAAAA
ATAGAGGAATTGAGACAGCATTTGTTAAGGTGGGGACTTACCACACCAGATAAAAAACAC
CAGAAAGAACCTCCATTCCTTTGGATGGGTTATGAACTCCATCCTGATAAATGGACAGTA
CAGCCTATAGTGCTGCCA
>89_CO
ACTCTTTgGCaACGACCCCTCGTCAACATAAAGGTAGGGGGGCAAATAAAGGAAGCTCTA
TTAgAtACAGGAGCAGATGATACAGTATTAGAAGAAATAAATTTRCCAGGAAGATGGAAA
CCAAAAATGATAGGGGGAATTGGRGGTTTTATCAAAGTAAGACAGTATGATCAGGTACtt
ATAGAAATCTGTGgATATAAAGCTATAGGtACAGTATTAGTAGGACCTACACCTGTCAAC
ATAATTGGAAgAAATCTGTTGACTCAGATTGGCTGCACYTTAAATTTTTGTACAGAAATG
GAAAAGGAAGGAAAAATCTCAAAAATTGGGCCCGAAAATCCATACAATACTCCAGTATTT
GCCATAAAGAAAAAAGACAGTACTAGATGGAGAAAATTAGTAGATTTCAGAGAACTTAAT
AAGAGAACTCAAGACTTCTGGGAAGTTCAATTAGGAATACCACATCCCGCAGGGTTAAAG
AAGAAAAAATCAGTAACAGTACTGGAT-TGGGT-ATG-ATATTTTTCAGTTCC-TTAGAC
AAAGACTTCAGGAAGTATACTGCATTTACcATACCTAGTACAAACAATGAAACACCAGGa
ATtAGATATCAGTACaATGtGCTGCCACAGGGGTGGAAAGGATCACCAGCAATATTCCAA

TGTAGCATGACAAAAATCTTAGATCCTTTTAGAAAACAAAATCCAGACATAATTATCTAT
CAATACGTGGATGATTTGTATGTAGCATCTGACTTAGAAATAGGGCAGCATAGAACAAAA
ATAAAGGAACTGAGACAACATCTGTTGAGGTGGGGTTTACCACACCAGACAAAAAACAT
CAGAAAGAACCACCATTTCTTTGGATGGGCTATGAACTCCATCCTRATACATGGACAGTA
CAGCCTATAGTGCTGCCA
>90_CO
ACTCTTTGGCAACGACCCtTAGTCATAATAAAGATAGAGGGACAGCTAAAGGAAGCTCTA
tTAGATACAGGAGCAGATGATACAGTATTAGAAGATATAAATTTGTcAGGAARATGGAAA
CCAAAAATGATAGGGGGAATTGGAGGTTTTATCAAAGTAAAACAGTATGATAACATACTC
ATAGAAATTTGTGGACACAAGGCTATAGGTACAGTGTtAATAGGACCTACGCCTGTCAAC
ATAATTGGAAGAAATATGTTGACTCAGATTGGTTGTACTTTAAATTTTTGcACAGAGATG
GAAAAGGAAGGAAAAATTTCAAAAATTGGGCCTGAAAATCCATACAATACTCCAGTATTT
GCCATAAAGAAAAAAGACAGTACTAAATGGAGAAAATTAGTAGATTTCAGAGAACTTAAT
AAGAGAACTCAAGACTTCTGGGAAGTTCAATTAGGAATACCACATCCCTCAGGGTTGAAA
AAGAAAAAGTCAGTAACAGTACTGGATGTGGGTGATGCATATTTTTCAGTTCCCTTAGAT
GAAGACTTCAGGAAGTATACTGCATTtACcATACCTAGTAYAAACAATGAGACACCAGGG
ATTAGATATCAGTACAATGTGCTACcaCAGGGATGGAAAGGATCACCAGCAATTTTTCAA
AGCAGTATGACAAAAATCTTAGAGCCTTTTAGAAAACAAAATCCAGACATAGTTATCTAT
CAATACGTGGATGACTTGTATGTAGGATCTGACTTAGAAATAGAGCAGCATAGAACAAAA
GTAGAGGAATTGAGACAGCATCTGTTGAGGTGGGGATTTACCACACCAGACAAAAAACAT
CAGAAAGAACCTCCATTCCTTTGGATGGGGTATGAACTCCATCCTGATAAGTGGACAGTA
CAACCTATAGTGCTGCCA
>91_CO
ACTCTTTGGCAACGACCCcTCGTCTCAATAAAGATAGGGGGGCAAATAAAAGAAGCTCTA
TTAgATACAGGAGCAGATGATACAGTGTtAGAAGAAATGAGTTtACCAGGAAGATGGAAA
CCAAAAATgATAGGGGGAATTGGAGGTTTTATCAAAGTAAGACAGTATGATCAAATACTt
ATAGAAATCTGTGGCCATAAAGCTATAGGTACGGTATTAGTAGGACCTACACCAGTCAAC
ATAATTGGAAGAAATCtGTTGACTCARATTGGATGCACTTTAAATTTTTGTaCAGAaATG
GAAAAGGAAGGAAAAATTTCAAAaATCGGGCCTGAAAATCCATACAATACTCCAGTATTT
GCTATAAAGAAAAAAGACAGTACTAAATGGAGAAAAGTAGTAGACTTCAGAGAACTTAAT
AAAAGAACTCAAGACTTCTGGGAGGTTCAATTAGGAATACCACAYCCCGCAGGGATAAAA
AAGARAAAATCAATAACAGTGTTGGATGTGGGTGATGCATATTTTTCAGTTCCCTTAGAT
AAAGAATTCAGGAAGtATACKGCcTTtACcATACCTAGTACAAACAATGArACACCrGGG

ATTAGATATCAGTAYAATGTGCTTCCAcAGGGATGGAAAGGATCACCAGCAATATTCCAG

AGTTGCATGACAAAAATCTTAGAGCCTTTTAGAAAACAAAATCCAGACATAGATATCTAT

CAATACGTGGATGATTTGTATGTAGGGTCGGACTTAGAAATAGGACAACATAGAACAAAA

ATAGAGGAACTRAGACAACACCTGTTAAGGTGGGGATTTACCACACCAGACGAAAAACAT

CAGAAAAAACCTCCATTCCTTTGGCTGGGTTATGAACTCCATCCTGATAAATGGACAGTA

CAGCCTATAATGCTGCCA

>92_CO

ACTCTTTGGCAACGACCCCTCGTCRCAATAAaGATAGGGGGGCAACTAAAGGAAGCTTTA

TTAgATACAGGAGCAGATGATACAGTATtAGAGGAAGTRAATTTACCAGGAAGATGGAAA

CCAAAAATgATAGGGGGAATTGGAGGTTTTATYAAAGTAaRaCAGTATGATCArATACCC

ATAGAAATCTGTGGACATAARGCTATAGGTACAGTATTAGTAGGGCCTACACCTGTCAAC

ATAATTGGAAGGAATCTGTTGACTCAGATTGGCTGTACTTTAAATTTTTGTACAGAAATG

GAAAAGGAAGGAAAAATTTCAAAAATTGGGCCTGAAAATCCATACAATACTCCAGTaTTT

GCCATAAAGAAAAAGGACAGTACTAAATGGAGAAAATTAGTAGATTTCAGAGAACTTAAT

AAGAGAACTCAAGACTTCTGGGAAGTTCAATTAGGAATACCACATCCCGCAGGGTTAAAA

AAGAAYAAATCAGTAACAGTACTGGATGTGGGTGATGCATATTTTTCAGTTCCCTtAGAT

AAAGACTTCAGAAAGTATACTGCATTCACCATACCcAGTAYAAACAATGAgACACCAGGG

ATTAGATATCAGTATAATGTGCTTCcACAGGGATGGAAAGGATCACCAGCAATATTCCAA

AGTAGCATGACAARAATCTTAGAGCCTTTTAGAMAACAAAATCCAGAAATRGTTATCTaT

CAATACGTGGATGAYTTGTATGTRGGMTCAGACTTAGAAATAGGGCAACATAGAACAAAA

ATAGAGGARCTRAGACAACATCTGTTGAGGTGGGGATTTACCACACCAGACAAGAAACAC

CAGAAGGAACCTCCATTCCTTTGGATGGGTTATGAACTCCATCCTGATAAATGGACAGTA

CAGCCTATAGTGCTGCCA

>93_CO

ACTCTTTgGcAACGACCCATCGTCACAATaAAagtagGGGGGcAACTAAAGGAAGCTTTA

TKGGATACAGGAGCAGATGATACAGTATTAGAAGAAATGAATTTACCAGGAAGATGGAAR

CCAAAAATGATAGGrGGAATTGGAGGTTTTRTCAAAGTAAGACAGTaTGATCAGRTAcCC

ATAGAAATCTGTGGACAGAAAACTGTAGGTACAGTATTAGTAGGACCTACACCTGyCAAC

ATAATTGGAAGAAATCTGATGACTCAAATTGGTTGTACTTTAAATTTTTGTACAGAAATG

GAAAAGGAAGGAAAAATTTCAAAAATTGGGCCTGAAAATCCATACAATACTCCAGTaTTT

GCyATAAAGAAAAAAGGCgGTACTAgATGGAGAAAATTAGTAGATTTCAGAGAACTTAAT

AAGAGAACTCAAGACTTTTGGGAAGTTCAATTAGGAATACCACATCCCTCAGGGTTACAC

AAGAACAAATCAGTAACAGTACTGgaTGTGGGTGATGCATATTTCTCAGTtCCtTTAGAT

GAAGAcTTCAGGAAGTATACTGCATTTACcATACCYaGTAYAAACAATGAGACACCAGGG
ATTAGATatCAGTACAaTGTGCTCCCACAAGGATGGAAAGGATCACCAGCARtATTCCAA
TGcAGCATGACAAAAATCTTAGAGCCTTTTAGAAAACAAAATCCAgACATAGTTATCTAT
CAATATGTRGATGATTTGTATGTAGCATCTGACTTAGAAATAGGGCaACATAGAGCAAAA
GTAGAGGAACTGAgACAACATYTGTTAAGGTGGGGGTTTWyWACACCAGACrAAAAACAT
CAGAAAGAACCTCCATTCCTTTGGATGGGGTATGAatcCcaTCCTGACAAATGGACAGTA
CAGCCTATAGTGCTGCCA
>94_CO
ACTCTTTGGCAACGACCACTAGTCAAmATAAAGGTAGGGGGGCAACTAAGGGAAGCTCTA
TTAGATACAGGAGCAGATGATACAGTATTAGAAGAAATAGAGTTGCCAGGAAGATGGAAA
CCAAAAWTGATAGGGGGAATTGGAGGTTTTATCAAAGTAAGACAGTATGATCAGATACYC
ATAGAAATTTGTGgACATAAAGCTATAGGTACAGTATTAGTAGGGCCTACACCTGCCAAC
ATAATTGGAAGAAATTTGTTGACTCAGATTGGATGCACTTTAAATTTTTGTACAGAAATG
GAAAAGGAAGGAAARATTTCAAAAATTGGGCCTGAAAATCCATACAATACTCCAGTATTT
GCCATAAAGAAAAAGGACAGTACTAAATGGAGAAAATTAGTAGACTTCAGAGAACTTAAT
AAGAGAACTCAAGACTTCTGGGAAGTTCAATTAGGAATACCACATCCCGCAGGGTTAAAA
AAGAAGAAATCAGTGACAGTACTAGATGTGGGTGATGCATATTTTTCAGTTCCCTTAGAY
AARGAMTTCAGGAARTATaCTGCATTcACcATACCTAGTACAAACAATGAGACACCAGGG
ATTAGATATCAGTACAATGTRCTTCCACAGGGATGGAAAGGATCACCAGCAATATTYCAr
AGTAGCATGACAAAAATCCTAGAGCCTTTTAGAAAACAAAATCCAGACATAGTTATCTAT
CAATACGTGGATGATTTGTATGTAGGATCTGACTTAGAAATAGGGCAGCATAGARCAAAA
ATAGAAGAACTGAGACAACATCTGTTGAGGTGGGGATTTACCACACCAGACAAAAAACAT
CAGAAAGAACCTCCATTCCTTTGGATGGGTTATGAGCTCCATCCTGATAAATGGACAGTA
CAGCCTATARTGTTGCCA
>95_CO
ACTCTTTgGCAACGACCCCTCGTCrCAATAARGGTAGAGGGRCAAYTAAAGGAAGCTCTA
TTAgATACAGGAGCAGATGATACAGTATTAGAAGAAATGAATTTGTCAGGAAGATGGAAG
CCAAAAATGATAGGGGGAATtGGAGGYTTTATCAAAGTAAGACAgTATGATCAGATAcTc
ATAGAAATCTGTGGmCATAAAGCTrTrGGTACAGTATTARTAGGACCTACACCTGTCAAC
ATAATTGGRAGAAATCTGTTGACTCArATTGGCTGCACTTtAAaTTTTTGTACAGAAATG
GAAAAGGAAGGaAAAATTTCAAAAATTGGGCCYGAAAATCCATACAATACTCCAGTATTT
GCYATAAAGAAAAARGACAGTACtAAATGGAGAAAATTAGTAGATTTTAGAGAACTTaAT
AAGAGAACTCAAGACTTCTGGGAAGTTCAAyTAGGAATACCACATCChGCAgGGtTAAAG

AAGAAAAAATCAGTAACAGTACTrgATGTGGGTGATGCATAYTTTTCAGTTcCCTTAGAT
RAGGACTTYAGGAAGTATACTGCATTTaCcATACCTAGTACAAACAATGAGACRCCAGga
AtTAgATATCAGTACaATGTACTyCCgCAGGgATGGAAAGGATCACCAGCAATATTCCAA
AGTAGCATGACAAARATCTTAGAACCTTTTAGRAAACAAAATCCAGACATAGTTATCTAT
CAATATATGGATgacTTGTATGTAGGATCTGACTTAGAAATAGGGCAGCATAGAACAAAA
GTAGAAGAACTAAGACAACACCTGCTGAARTGGGGGTTAACCACACCAGACAArAAACAT
CAGAAAGAACCAcCATTCCTTTGGATGGGTTATGaACTCCATCCTGATAAATGGACAGTA
CAGCCTATAGTGCTrCCA
>96_CO
ACTCTTTGGCAACGaCCAATAGTCAAATTAAAGGTAGGGGGGCAACTAAAGgAAGCTCTA
TTAGATACAGGAGCAGATGATACAGTaTTAGAAGACATGgAATTACCAGGAAGATGGAAA
CCAAAAATGATAGGGGGAATTGGAGGTTTTATCAAAGTAAGACAATATGATCAAATATCC
AtAGAAATCTGTGGAACGAAAGCTATAGGTACAGTATTAGTAGGACCTACACCTGTCAAC
ATAATTGGAAGAAATCTGTTGACTCAGATAGGTTGCACTTTAAATTTTtgTACAGAAATG
GAAAAGGAAGGAAAAATTTCAAAAATTGGGCCTGAAAATCCATACAATACCCCAGTATTT
GCCATAAAGAAAAAGGATAGTACTAAATGGAGAAAATTAGTGGATTTCAGAGAACTTAAT
AAAAGAACTCAAGACTTTTGGGAAGTTCAATTAGGAATACCACATCCCGCAGGGTTAAAA
AAGAAAAAGTCAGTGACAGTACTGGATGTGGGTGATGCATATTTTTCAGTTCCCTTAGAT
AAAGACTTTAGGAAGTATACTGCATTtaCCATACCTAGTACAAACAATgAGACACcaGGA
aTTAGATATCAGTACAATGTGCTTCCACAGGGATGGAAAGGATCACCAGCAATATTCCAA
AGCAGCATGACkAAAATCTTAGAGCCTTTTAGAAAACaAAATCCAGACATGGTTATCTAT
CAATACATGGATGATCTGTATGTAGGATCTGACTTAGAAATAGGGCAGCATAGAACAAAA
ATAGAGGAACTGAGACAACATCTGTTGAGGTGGGGGTTTACCACACCAGACAAAAAACAT
CAGAAAGAACCGCCATTCCTTTGGATGGGTTATGAACTCCATCCTGATAAATGGACAGTA
CAGCCTATAATACTCCCA
>97_CO
acTTTTgGCAAaCGACCcTTAgTCACAAtAaAagtAGGGGGGCAGCTAAGGGAAGCTcTa
TTAGATACAGGAGCAGATGATACAGTATTAGAAGATATAAaTTTGYCAGGAAAATGGAAA
CCAAAAATgATAGGGGGAATtGGAGGTTTTATCAAAGTaAAACAGTATgATGAYGTATGC
ATAGACATTTGTGGACACAAGGCTACGGGTACAGTRTTAGTAGGACCTACACCTGTCAAC
ATAATTGGAAGAAATCTGTTGACTMAGMTTGGTTGCACTTTAAATTTTTGTACAGAaATG
GAAAAGGAAGGaAAAATTTCAAAAATYGGGCCTGAAAATCCATATAACACTCCAGTATTT
GCTATAAAGAaAAAAGACAGTACTAAATGGAGAAAATTAGTAGATTTCAGAGAACTTAAT

AAAAGAACTCAAGACTTTTGGGAGGTTCAATTAGGAATACCGCACCCTGCAGGGYTAAAA
AAGAAAAAGTCAGTRACAGTACTAGATGTGGGAGATGCATATTTTTCAGTGCCCTTAGAT
AAGGAATTCAGGAARTACACTGCATTCACCATACCTAGTGTCAACAATGAGACACCAGGA
ATTAGGTACCAGTAYAATGTGCTTCacaAgGGATGGaAAGGATCACCAGCAATATtTCAA
TGTAGCATGACAAAaATCTTAGAGCCTTTTAGAAAACAAAATCCAGACATAGTGATTTAC
CAATACATGGATgATTTGtATGTAGGATCTGACTTAGAAATAGGACAGCATAGAACAAAA
ATAGAAGAATTAAGAGAACACCTRCTaAaatGGGRaTTTACTACACCAGACAAGAAACAT
CAAAAGGAACCYCCATTTCTTtGGATGGGGTATGAACTCCATCCTGATAAATGGACAGTG
CAGCCTAtACTATTGcCA
>98_CO
ACTCTTTGGCAaCGACCCCTTGTCACAAtAaagAtAGAGGGACAGTTAAAGGAGGCTCTC
TTAGACACAGGAGCAGATGATACAGTATtAGAAGAAATRAAATTGCCAGGAAATTGGAAA
CCAAAAATGATAGGAGGAATTGGAGGtTTTATCAAAGTRAGACAATATGATCAAATACTT
ATAGAAATaTGTGGAAAAaAGGCTATAGGTACAGTACTAGTAGGACCTACACCTGTCAAY
ATAATTGGAAGGAACMTGTTGACTCAGCTaGGATGCACACTAAATTTTTGTGATGAAATG
GAGAGGGAAGGAAAAATTACAAAAATTGGGCCTGAAAATCCATATAACACTCCAGTATTT
GCCATAAAAAAGAAGGACAGTACYAAGTGGAGRAAATTAGTAGATTTCAGGGAACTCAAT
AAAAGGACTCAAGACTTTTGGGAAGTTCAATTAGGGATACCACACCCAGCAGGGTTAAAA
AAGAAAAAATCAGTGACAGTACTGGATGTGGGGGATGCATATTTTTCAGTacCtTTAGAT
GAAGACTTTAGAAAATATACTGCATTcACcATACCTaGTACAAACAATGMAACACCAGGG
ATTAGATATCAATATaATGTGCTTCCACAGGgATGGAAAGGATCACCAGCAATATtCCAG
TGTAGTATGACAAAaATCTTAGAGCCCTTTAGAGCACAAAATCCAGAaATAGTTATCTAT
CAATATATGGATgAYTTGTATGTAGGATCTgACTTAGAAATAGGGCAACATAGAGCAAAA
ATAGAGGAGTTAAGAGAACATCTAtTRAAGTGGGGATTCACCACACCAGACAAGAARCAT
CAgAAAGAACCCCCATTTCTTTGGATGGGGTATGAACTCCATCCTGACAAATGGACAGTa
CAGCCtATACAGCTGCCA
>99_CO
ACTCTTTGGCAACGACCACTAGTCAAcATAAAGGTAGGgGGGCAAATAAAAGAAGCTCTA
TTAGATACAGGAGCAGATGATACAGTATTAGAAGACATAGAGTTGCCAGGAAGATGGAAA
CCAAAAATGATAGGGGGAATTGGAGGTTTTATCAAAGTAAAACAGTATGATCAGATAACC
ATAGAAATCTGTGGCCATAAAGCTATAGGTACAGTATTAGTAGGACCTACACCTGYCAAC
ATAATTGGAAGAAATCTGTTGACTCAGATTGGCTGCACTTTAAATTTTTGTACAGAAATG
GAAAAGGAAGGaAAAATTTCAAARATTGGGCCTGAAAATCCATACAATACTCCAGTATTT

GCCATAAAGAAAAAGGATGGTACTAAATGGAGAAARTTAGTAGATTTCAGAGAACTCAAT

AAGAGAACTCAAGACTTCTGGGAAGTTCAATTAGGAATACCACATCCCGCAGGGCTGAAA

CAGAAAAAATCAGTAACAGTgCTGGATGTgGGTGATGCATATTTTTCAGTTCCCTTAGAT

GAAGACTTCAGGAAGTATACTGCATTTACcATACCTAGTACAAACAATGAGACACCAGGG

ATTAGATATCAGTACAATGTGCTTCCACAGGGATGGAAAGGATCACcAGCaATATtYCAA

AGCAGTATGACAAGAATCTTAGAGCCTTTYAGAAAACAAAATCCAGACMTAGTTATCTAT

CAATACATGGATGATTTGTATGTAGGATCTGACTTAGAAATAGAACAGCATAGAACAAAA

ATAGAGGAACTGAGACARCATCTGTTGAAGTGGGGATTTACCACACCAGACAAAAAACAT

CAgAAagAaCCtCCATTCCTTTGGATGGGTTATGAACTCCATCCTGATAAATGGACAGTA

CAGCCCATAATGCTGCCA

>100_CO

aCACTTTTCgGACGRCCCGyTGTCACaATAAAaGTAgGGGGACAGcTAAAGGAGGCTCTC

TTAgacACAGGAGCAGATGATACAGTATTAgAAGAAACAAAATTGCCAGGAAATTGGAAA

CCAAAAATGATAGGAGGAATTGGGGGTTTTATCAaAGTAAGACAGTATGATCAAATAACT

ATAGAAATTTGTGGRAAAAAGGCTATAGGTACAGTATTAGTAGGACCTACACCTGTCAAC

ATAATTGGGAGAAACATRTTGACTCAGCTTGGATGCACACTAAATTTTTGTGATGAaATG

GaGAaGGaAGGAAAAATTACAAAAATTGGGCCTGAAAATCCATATAACACTCCAGTATTT

GCCATAAAGAAAAAAGACAGTACTAAATGGAGAAAATTAGTAGATTTTAGAGAACTTAAT

AAGAGAACTCAAGACTTCTGGGAGGTTCAATTAGGAATACCACATCCCGCAGGGTTRAAA

AAGAAAAAGTCAGTAACAGTACTGGATGTGGGTGATGCATATTTTTCAGTTCCCTTATAT

GAAGATTTYAGAAAGTATACTGCATTTACcATACCTAGTATAAAYAATGAGACACCAGGG

GTTAGATATCAGTAcAATGTGCTTCCACAAGGATGGAAAGGATCACCAGCaATATtCCAa

AGTAGCATGACAAAAATCTTAGAGCCTTTTAGAAAACAAAATCCAGACtTAATTATCTAT

CAATATATGGATGACTTGTATGTAGGATCTGACTTAGAAATAGGGCAACATAGAGCAAAA

ATAGAGGAGTTAAGAAAACATCTATTGCAGTGGGGATTCACTACACCAGACAAgAAACAT

CAGAAAGAACCCCCATTTCTTTGGATGGGGTATGAACTCcATCCTGACAAATGGACAgTA

CAgcCTATACAGCTGCca

>101_CO

actctTTgGCAACGaCCCCTCGTcACAATAAGGGTAGAGGGGcaAcTAAAAGAAGCTCTA

TTAGATACAGGAGCAGATGATACAGTATTAGAAGACATGGATTTGCCAGGGAGATGGAAG

CCAAAAATGATAGGGGGAATTGGAGGTTTTATCAAAGTAAGACAGTATGATCAAATACTA

GTAGAAATCTGTGGACATAAAGCTATAGGTACAGTATTAGTAGGGCCTACACCTGTCAAC

ATAATTGGAAGAAATCTGTTGACTMRGATTGGCTGCACTTTAAATTTTTGTACAgAAATG

GAAAAGGAAgGAaaAATTTCAAAAATTGGGCCTGAAAATCCATACAATACTCCAGTATTY
GCCATAAAGAAAAAGGACAGTACTAAATGGAGAAAATTAGTAGATTTCAGAGAACTTAAT
AAGAGAACTCAAGACTTCTGGGAAGTTCAATTAGGAATACCACATCCCGcAGGATTAAAA
AAGAAAAAATCAGTAACAGTACTGGATGTGGGTGATGCATATTTCTCAGTTCCTTTAGAT
AAAGACTTCAGGAAGTACACTGCATTTACCATACCTAGTATAAACAATGAGACACCAGGG
ATTAGATaTCAGTACAATGTGCTTCCACAAGGATGGAaAGGATCACCAGCAATATTYCAA
TGTAGCATGACAAAAATCTTAGATCCTTTTAGAAAACAAAATCCAGAYATAGTCATCTAT
CAATACATGGATGATTTGtATGTAGGATCTGACTTAGAAATAGGGCAGCATAGAACAAAR
ATAGATGAACTGAGAAACCATCTGTTGAAGTGGGGGTTCACCACCCCAGACAAAAAGCAT
CAgAAAGAACCTCCATTCCTGTGGATGGGTTATGAACTCCATCCTGATAAATGGACAGTA
CAGCCTAtAATGTTGCca
>102_CO
ACTTCTTTGCAACGACCCCTCGTcacaAtAAAgGtAGGgGGGcAAATTAAGGAAGCTcTa
tTAGATACAGGAGCAGATGAtACAGTATTAGAAGAAATAAATTTACCAGGAAAATGGAAA
CCAAAAATGATAGGGGGAATAGGAGGTTTTATCAAAGTAAGACAGTATGATCATATACTC
ATAGAAATCTGTGGACACAAAGCTATAGGTACAGTATTAGTAGGACCTACACCTGTCAAC
ATAATTGGgAGaAATTTGTTGACTCAAATTGGCTGCACTTtAAATTTTTGTACAgaAATG
GAAAgGGgAgGGAAAAATTCAAAAATTGGGcCTGAAAaTcCATACAATAcTCCAGtaTTT
GCCATAAAGAAAAAAGACAGTACTAAATGGAGAAAATTAGTAGATTTCAGAGAACTTAAT
AAGAGAACTCAAGACTTCTGGGAAGTTCAATTAGGAATACCGCATCCCGcAGGGTTAAAA
AAGAAAAAATCAGTAACAGTACTGGATGTGGGTGACGCATATTTTTCAATTCCCTtAGAT
aAAGAGTTCAGGAAGTATACTGCATTCACCATACCTAGTACaAACAATGAGACACCAGGG
ATTAGATATCAGTaCAATGTGCTCCCaCAGGGATGGaAAGGATCACcAGCaATATtTCAA
AGTAGCATGACAAAAATCTTAGAGCCTTTTAGAAAACAAAATCCAGAAATAGTTATCTAT
CAATACATGgATgAtTtGtATGTAGgATCTGACTTAGAAATAGGGCAACATAGGACAAAA
ATAGAAGAACTGAGACAACATCTGTTGAGGTGGGGACTTACCAcACCAGACAAGAAACAT
CAggAAgaAcCTCCATTCCTTTGGATGGGTTATGAACTCCATCCTGATAAATGGACAGTA
CAGCCTATAgTGCTGCCA
>103_CO
actctttgGCAACGACCCatCGTCACAATAAAAGTAGGGGGGCAACTAAAGGAAGCCcTT
tTAgATACAGGAGCAGATGATACAGTATTAGAGGACATAAATTTGCCAGGGAGATGGAAA
CCAAAAATGATAGGGGgAATTGGAGGTTTTATCAAAGTAAGACAATATGATCAGGTAGCC
ATAGAAATCTGTGGACATAAGGCTATAGGTACAGTATTAGTAGGACCTACACCTGTCAAT

ATAATTGGAAGaAATCTGTTGACTCAACtTgGTTGCACTCTAAaTTTTTGTACAGaAATg

GAAAAGGAAGGgAAAaTTTCAAAAATTGGGCCTGaAAATCCATACAatacTCCAGTATTT

GCCATAAAGAAgAAAGACAGTACTAAATGGAGAAAATTAGTAGATTTTAGAGAACTTAAT

AAGAGAACTCAAGATTTCTGGGAAGTCCAGTTAGGAATACCTCATCCATCAGGGTTAAAA

CAGAAGAAATCAGTAACAGTACTGGATGTGGGTGATGCCTATTTTTCAGTTCCTTTAGAT

AAgGaATTTAGGAAGTAcACTGCATTTACCATACCTaGTATAAACaaTgAGACACCAGGG

ATTaGaTATcAGTACAATGTRCTTCcAcAGGgATGGAAAGGATCACCAGCAATATTCCAA

TGTAGCATGACAAAgATCTTAGATCCTTtTAGGAaGAAAAATCCAGACATAGTGATCTAT

CAATACATGGATGATTTGtATGTAGGATCTGACTTAGAAATAGGGCAGCATAGAgaaAAA

ATAGAAGAACTGAGAAAACATCTGTTGGCGTGGGGATTTACCAcACCAGACAAgAaACAT

CAGAAAGAACCTCCATTCCTTTGGATGGGTTATGAACTCCATCCTGATAACtGGACAGTA

CAaCCtaTAGTGCTGCCA

>104_CO

ACTCTTTGGCAACGACCAGTAGTCACAATAAAGGTAGGGGGGCAATTAAAgGAAGCTCTA

TTAGATACAGGAGCAGATGATACAGTATTAGAAGAAATGAATTTGCCAGGAAAATGGAAA

CCAAAAATGATAGGGGGAATTGGAGGTTTTATCAAAGTAAGAcAGTATGACCAGATACTT

GTAGAAATCTGTGgCCATAAAGCTATAGGTACAGTATTAGTAGGGCCTACGCCTGTCAAC

ATaATTGGAAGAAATCTGTTGAcTCAGCTTGGctgcACTTTAAATTTttGTACAGAATTG

GAAAAGGAAGGaAAGATTTCAAAAATTGGGCCTGACAATCCATACAATACTCCAGTATTT

GCCATAAAGAAAAAGGACAGTACTAAATGGAGAAAATTAGTAGATTTCAGAGAACTTAAT

AAGAGAACTCAAGACTTCTGGGAAGTTCAATTAGGAATACCACATCCCGcAGGGTTAAAA

CAGAAAAAATCAGTAACAGTACTGGATGTGGGTGATGCATATTTTTCAGTTCCCTTAGAC

GAGGACTTCAGGAAGTATACTGCATTtACCATACCTAGTATAAACAATGAGACACCAGGG

ATTAGATACCAGTACAATGTGCTtcCACAAGGATGGaaRGGATCACCAGCAATATtCCAA

TGTAGCATGACAAAAATCTTAGAGCCCTTTAGAAAACAAAATCCAGAAATAGTTATCTAT

CAATACATGGATGATTTGtATGTAGGATCTGACTTAGAAATAGGGCAGCATAGAgCaAAA

ATAGAGGAACTGAGaCAACACCTGTTGAAGTGGGGgTTTgAGAcacCAGACaAAAAACAT

CAgAAAGAACCTCCATTtCTTTGGATGGGTTATGAACTCCATCCTGATAAATGGACAGTA

CAGCCTATAAaTCTGCCA

>105_CO

ACTCTTTGGCAGCGaCCCCTTGTCACaATAAAAATAGagGGaCAGTTAAARGAGGCTCTC

TTAGACACAGGAGCAGATGATACAGTATtAGAAGAAATGAAATTGCCAGGAAATtGGAAA

CCAAAAATGATAGGAGGAATTGGAGGtTTTATCAAAGTGAGACAATATGATCAAATAcYT

ATAGAAATYTGTGGAAAAAAGGCTATAGGTACAGTACTAGTAGGACCTACACcTGTCAAC
ATAATWGGAAGGAACCTGTTGACTCAGCTrGGATGCACACTAAaTTTTTGTGATGARATG
GAgAgGGAAGGAAAAATTACAAAAATTGGGCCTGAAAATCCATATaAcACTCCaGTaTTT
GCCATAAAAAAgaAGGACAgTaCTAaGTGGagAAAAtTaGTaGATTTcaGGGAACTCAAT
AAAAgGACTCAAGAcTTTTGGGAagTTcAaTtAGGGaTaCcAcaCCCaGcAGGGTtAAAA
AAGrAAAAATCAGTGACAgTACTGGATgTgGGGGATGCATATTytTCAGTacCtTTAGAT
GAAGAATTTAGAAAATATACTGCATTcACCATACCTaGTACAAACAATGArACACCAGGa
ATtAgATATCAATATaATGTGCTTCCaCAGGGATGGaAAGGATCACCAGCaATATTCCAG
TGTAGTATGACAaAaATCTTAGAGCCCTtTAGAGCACAAAATCCAGAAATAGTTATCTAT
CAATATATGGATgAYTTGtATGTAGgATCyGACTtAGAAATAGGGCAACATAGAGCAAAA
RTAGagGaRtTaAGAGAaCaTCTaTTGAaGTGGGGATTCACYAcACCAGACAAGAAACAT
CAGAAAGAACCCCCATTTCTTTGGATGGGGTATGAACTCCaTCCTGACAAATGGACAGTA
CAGCCTATACAGCTGCCA
>106_CO
ACTCTTTGGCAACGACCCCTCGTCACAATAAAGATAGGGGGGCAATTAAAGGAAGCTCTA
TTAGATACAGGAGCAGATGATACAGTATTAGAAGAAATGAATTTGCCAGGGAGATGGAAA
CCAAGAATGATAGGGGGAATTGGAGGCTTTATCAAAGTAAGACAATATGACCAGATATCC
TTAGAAATCTGTGGACATAAAGCTATAGGTACAGTATTAATAGGACCTACACCTGTCAAC
ATAATTGGAAGAAATTTGTTGACTCAGATTGGCTGCACTTTAAATTTTtGTACAGAAATG
GAAAAGGAaGGaAAAATTTCAaAAATTGGGCCTGAAAACCCATACAATACTCCAGTATTT
GCCATAAAGAAAAAAGACAGTACTAAATGGAGAAAATTAGTAGATTTCAGAGAACTTAAT
AAAAGAACTCAAGACTTTTGGGAAGTTCAATTAGGAATACCACATCCCGCAGGGTTAAAG
AAGAAAAAATCAGTAACAGTACTGGATGTGGGTGATGCATATTTTTCAGTTCCCTTAGAT
AAAGACTTTAGGAAGTATACTGCATTTaCcATACCTAGTATAAACAATGAAACACCAGGA
ATTAGATATCAGTACAaTGTGCTtCCACAGGGATGGAAgGGATCACCAGCAATATTCCAA
AGTAGCATGATAAAAATTTTAGAGCCTtTTAGGAAGGAAAACCCAGACATAGTCATCTAT
CAATACATGGATGATTTGTATGTAGGATCTGATTTAGAAATAGGGCAGCATAGAGCAAAA
GTAGAGGAACTAAGGCAACATCTGCTGAAGTGGGGGTTTACTACACCAGACaAAAAACAT
CAGAaAGAACCACCATTCCtTTGGATGGGTTATGAACTCCATCCTGATAAATGGACAGTA
CAGCCTATAGTGCTgCCA
>107_CO
ACTCTTTGGCAACGACCCCTCGTCTCAATAAAGGTAGGGGGGCAACAAAAGGAAGCTCTa
TtAGACACAGGAGCAGATGATACAGTATTAGAAGAAATGAATTtACcAGGAAAATGGAAA

CCAAAAATgATAGGGGGAATTGGAGGTTTTATCAAAGTAAGACAGTATGATCAGATACCC

ATAGAAATCTGTGGACATAAAGCTATAGGTACAGTATTAGTAGGgCCCACACCTGTCAAC

ATaATTGGAAgAAATCTGTTGACTCAGATTGGCTGCACTTTAAATTTTTGTACAGAAAtG

GAAAAGGAAGGAAAAATTTCAAAAATTGGGCCTGAAAATCCATACAATACTCCAGTATTT

GCCATAAAGAAAAAAGACAGTACTAAATGGAGAAAATTAGTAGATTTTAGGGAACTTAAT

AAGAGAACTCAAGACTTCTGGGAAGTTCAATTAGGAATACCACATCCCTcAGGGTTAAAA

AAgAAAAAATCAGTAACAGTACTGGATGTAGGTGATGCATATTTTTCAGTTCCCTTAGAT

AAAGAATTCAGGAAGTATACTGcATTTACAATACCTAGTACAAACAATGAGACACCAGGG

ATTAGATATCAGTATAATGTgCTTCCACAAGGATGGAAAGGATCACCAGCAATATTCCAA

AGTAGCATGACAAAAATCTTAGAGCCTTTTAGAAAAcAAAATCCAGAAATAGTCATTTAT

CAATACATGGATGATTTGTATGTAGGATCTGACTTAGAAATAGAGCAGCATAGAACAAAG

ATAGAGGAAcTGAGACAACATCTGTTGAGGTGGGGATTTACCACaCCAGACaaaaaACAT

CAgaaAgAACCTCCATTCCTTTGGATGGGGTATGAACTCCATCCTGATAAATGGACAGTA

CAGcCTATAaCGCTGCCA
